# Multivalent Histone and DNA Engagement by a PHD/BRD/PWWP Triple Reader Cassette Recruits ZMYND8 to K14ac-Rich Chromatin

**DOI:** 10.1016/j.celrep.2016.11.014

**Published:** 2016-12-06

**Authors:** Pavel Savitsky, Tobias Krojer, Takao Fujisawa, Jean-Philippe Lambert, Sarah Picaud, Chen-Yi Wang, Erin K. Shanle, Krzysztof Krajewski, Hans Friedrichsen, Alexander Kanapin, Colin Goding, Matthieu Schapira, Anastasia Samsonova, Brian D. Strahl, Anne-Claude Gingras, Panagis Filippakopoulos

**Affiliations:** 1 Structural Genomics Consortium, Nuffield Department of Clinical Medicine, University of Oxford, Old Road Campus Research Building, Roosevelt Drive, Oxford OX3 7DQ, UK; 2 Ludwig Institute for Cancer Research, Nuffield Department of Clinical Medicine, University of Oxford, Old Road Campus Research Building Roosevelt Drive, Oxford OX3 7DQ, UK; 3 Lunenfeld-Tanenbaum Research Institute at Mount Sinai Hospital, 600 University Ave., Toronto, ON M5G 1X5, Canada; 4 Department of Biochemistry and Biophysics, University of North Carolina, Chapel Hill, NC 27599, USA; 5 Department of Oncology, University of Oxford, Old Road Campus Research Building, Roosevelt Drive, Oxford OX3 7DQ, UK; 6 Structural Genomics Consortium, MaRS Centre, South Tower, 101 College St., Suite 700, Toronto, ON M5G 1L7 Canada; 7 Department of Pharmacology and Toxicology, University of Toronto, Toronto, ON M5S 1A8, Canada; 8 Lineberger Comprehensive Cancer Center, University of North Carolina, Chapel Hill, NC 27599, USA; 9 Department of Molecular Genetics, University of Toronto, Toronto, ON M5S 1A8, Canada

**Keywords:** chromatin binding, multivalency, structural rigidity, plasticity, histone marks, masking of chromatin binding, DNA damage, protein network assembly

## Abstract

Elucidation of interactions involving DNA and histone post-translational-modifications (PTMs) is essential for providing insights into complex biological functions. Reader assemblies connected by flexible linkages facilitate avidity and increase affinity; however, little is known about the contribution to the recognition process of multiple PTMs because of rigidity in the absence of conformational flexibility. Here, we resolve the crystal structure of the triple reader module (PHD-BRD-PWWP) of ZMYND8, which forms a stable unit capable of simultaneously recognizing multiple histone PTMs while presenting a charged platform for association with DNA. Single domain disruptions destroy the functional network of interactions initiated by ZMYND8, impairing recruitment to sites of DNA damage. Our data establish a proof of principle that rigidity can be compensated by concomitant DNA and histone PTM interactions, maintaining multivalent engagement of transient chromatin states. Thus, our findings demonstrate an important role for rigid multivalent reader modules in nucleosome binding and chromatin function.

## Highlights

• The ZMYND8 PHD/BRD/PWW cassette forms a stable structural reader ensemble
• The reader ensemble binds chromatin via simultaneous engagement of histones and DNA
• Each module of the ZMYND8 reader ensemble contributes to chromatin interactions
• Genome-wide distribution of ZMYND8 at enhancers is mediated by K14ac interactions

## Graphical Abstract

**Figure:**
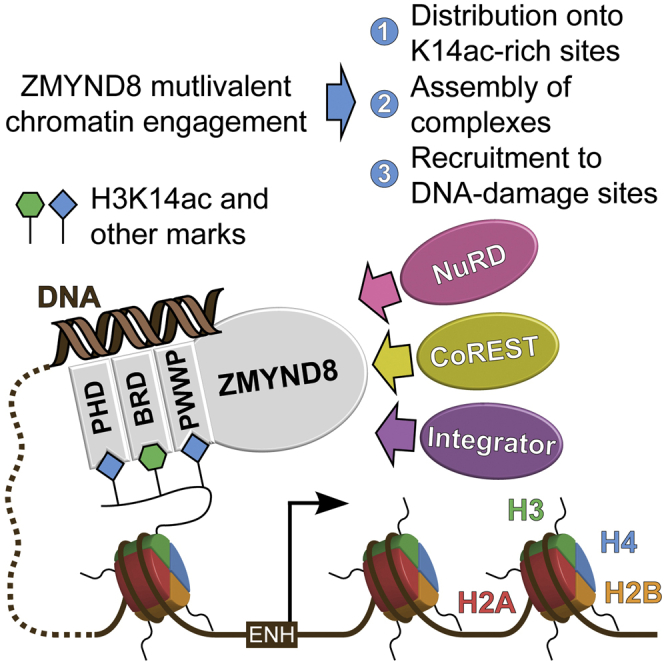

## Teaser

Savitsky et al. resolve the crystal structure of the PHD/BRD/PWWP cassette of ZMYND8 and demonstrate that tethering to K14ac-decorated chromatin is mediated through simultaneous engagement of histones and DNA by this triple reader ensemble. Such multivalent interactions control ZMYND8 participation in transcriptional complexes and recruitment to damaged DNA sites.

## Introduction

Post-translational modifications (PTMs) on histones and DNA are critical regulators of chromatin stability, structure, and gene expression (Schübeler, 2015, Tessarz and Kouzarides, 2014). Combinations of histone PTMs have been recognized to constitute a cellular language, involving deposition, interpretation, and removal of PTMs, referred to as the “histone code” (Strahl and Allis, 2000). Different classes of protein interaction modules (“readers”) have evolved to recognize and bind to specific PTMs, including lysine methylation and acetylation, among many known PTMs (Choudhary et al., 2009, Tan et al., 2011). Acetylated lysines (Kac) are recognized by bromodomain modules (BRDs) with affinities ranging in the micromolar range (Filippakopoulos et al., 2012); a single BRD can also bind two adjacent acetylation marks (Filippakopoulos et al., 2012, Morinière et al., 2009), thus enhancing affinity. Recognition of multiple histone PTMs by employing several effector modules present within a given protein confers avidity and increases specificity/affinity as a result of multivalency (Ruthenburg et al., 2007).

Multivalency has been studied in BRD-containing proteins involving two effector modules, often another BRD or a plant homeodomain (PHD). For example, in the case of the transcription initiation factor TFIID subunit 1 (TAF1), two BRD modules exist in a rigidly confined topology, allowing for optimal recognition of multiple acetylated lysines on histone H4 with low micromolar affinity (Jacobson et al., 2000). Multi-domain or paired arrangements including a PHD/BRD cassette provide a platform for combinatorial recognition of lysine methylation and acetylation, introducing higher specificity. For example, the chromatin regulator tripartite motif 24 (TRIM24) binds to the N-terminal H3 tail, simultaneously engaging lysine 4 (H3K4), via a PHD finger, whereas an adjacent BRD module linked via a flexible loop binds to acetylated lysine 23 (H3K23ac) (Tsai et al., 2010). The relative topology between two effector modules affects the type of signals interpreted as a function of the linker connecting the modules that acts as a molecular ruler; for instance, the PHD/BRD cassette found in BPTF can recognize and bind to histone 3 dimethyl-lysine 4 (H3K4me_2_) and histone 3 trimethyl-lysine 4 (H3K4me_3_) (Li et al., 2006) via the PHD domain, facilitating the adjacent BRD to gain specificity for histone 4 lysine 16 acetyl (H4K16ac) found in *trans* within a single nucleosome over other H4 acetylations (Ruthenburg et al., 2011). Therefore, combination of effector modules not only introduces conformational plasticity, leading to specificity, but also introduces avidity, resulting in higher affinities. However, it is not clear whether this is an additive effect so that introduction of additional modules will result in similar enhancements offering specificity toward developing landscapes of PTMs.

To better understand how multivalent interactions involving more than two reader domains impact recognition of PTMs, affecting protein function, we studied the topology of the N-terminal triple reader domain architecture found in the zinc-finger MYND domain-containing protein 8 (ZMYND8), which contains, in addition to a PHD/BRD arrangement, a Pro-Trp-Trp-Pro (PWWP) domain within a PHD/BRD/PWWP cassette. ZMYND8 has been previously found to participate in transcriptional regulation complexes (Eberl et al., 2013, Havugimana et al., 2012, Kloet et al., 2015, Malovannaya et al., 2011), is implicated in gene silencing (Poleshko et al., 2010), acts as a DNA damage response element (Gong et al., 2015), and has recently been proposed to control, together with KDM5C, enhancer activity (Shen et al., 2016). Here we describe the high-resolution crystal structure of the ZMYND8 N-terminal triple reader PHD/BRD/PWWP module and show how contributions from multivalent, simultaneous recognition of DNA and histone PTMs drive ZMYND8 function, affecting recruitment to DNA-damaged sites.

## Results

### ZMYND8 Triple Reader PHD/BRD/PWWP Modules Form a Stable Unit

**Figure 1:**
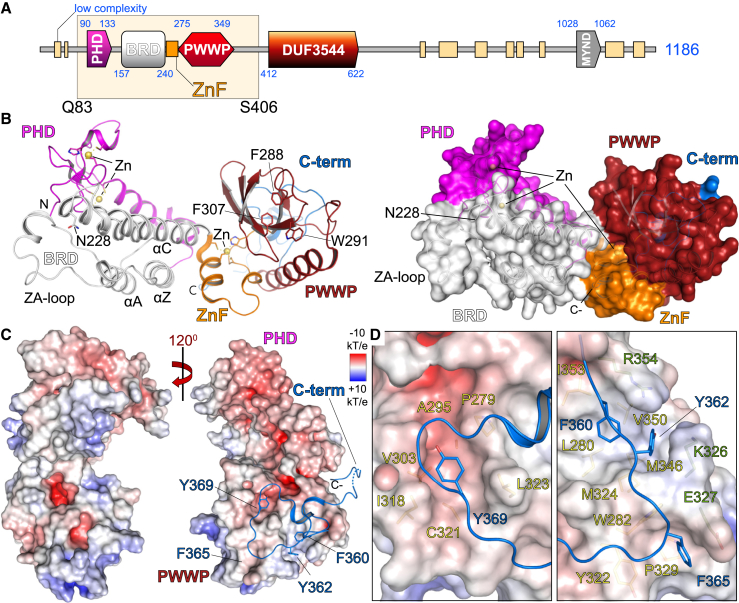
Structure of the ZMYND8 Triple Reader Module (A) ZMYND8 has a modular architecture, including an N-terminal PHD/BRD/PWWP reader cassette and a C-terminal MYND interaction domain. A recombinant construct expressing residues Q83–S406 was structurally characterized. (B) The crystal structure of the ZMYND8 reader modules is shown, with major secondary structural elements highlighted on the ribbon (left) and surface (right) models. The conserved Kac peptide docking residue (N228) of the BRD and the aromatic cage of the PWWP domain (F288, W291, and F307) are highlighted. The domain topology is highlighted on the right, with domains individually colored. (C) Electrostatic distribution of charge displaying the highly charged surface of the protein. Rotation reveals the insertion of key aromatic residues (F360, Y362, F365, and Y369), found on the C-terminal portion of the construct, into the back of the PWWP domain, stabilizing this module. (D) Detail of the C-terminal tail packing onto the surface of the PWWP domain, revealing three large hydrophobic pockets that accommodate the C-terminal peptide. The first pocket (P279, A285, V303, I318, C321, and L323) accommodates Y369 (left). The second pocket (I353, L280, V350, M324, and M346) is capped by R354 and K326 and accommodates F360 and Y362 (right). The third pocket (W282, Y322, and P329) is capped by E327 and accommodates F365 (right).

Guided by sequence similarity to the recently structurally characterized BRD/PWWP tandem modules of ZMYND11 (Wang et al., 2014, Wen et al., 2014) and by testing expression constructs of varying lengths, we expressed and crystallized the PHD/BRD/PWWP cassette of ZMYND8. The 1.7-Å crystal structure showed that the PHD domain packs next to the BRD domain, whereas a zinc-finger motif linked the PHD/BRD and PWWP modules (Figures 1A and 1B). ZMYND8 contains the conserved asparagine responsible for Kac peptide docking onto BRD modules (Filippakopoulos et al., 2012), a feature missing in the ZMYND11 BRD, which, instead, contains a tyrosine residue and was not found to recognize Kac (Wang et al., 2014, Wen et al., 2014; Figure S1A). The distribution of charge on the surface of the ZMYND8 structure revealed highly charged patches as well as a large hydrophobic area on the back of the PWWP domain (Figure 1C). A C-terminal extension necessary for expression of the three protein modules packed onto this hydrophobic area of the PWWP domain, inserting bulky hydrophobic residues (F360, Y362, F365, and Y369) into pockets formed on the PWWP surface (Figure 1D). The interactions of the C-terminal extension with the PWWP domain explain the necessity for its presence, conferring stability to the triple reader modules. Structural and sequence alignment with ZMYND11 did not reveal any similarities in this region of the protein, suggesting that the observed C-terminal interactions are unique to ZMYND8.

### The ZMYND8 Reader Ensemble Provides Structural Clues for Histone Peptide Binding

The similar orientation between the BRD/PWWP modules of ZMYND8 and ZMYND11 as well as the existence of a number of similar residues previously associated with histone isoform-specific interactions of ZMYND11 (Figures S1B and S1C) prompted us to examine whether the reader modules of ZMYND8 provide a structural template for histone peptide recognition. PHD domains recognize and bind to Kme_x_ sequences found on histones; in the presence of a full aromatic cage, H3K4me_3_ inserts within the cage while a bulky residue separates the modified lysine from the adjacent arginine (R2), as exemplified in the case of the PHD finger of BPTF bound to a histone H3 K4me_3_ peptide (Li et al., 2006; Figure S2A). A partial aromatic cage can also accommodate K4me_3_, as exemplified in the ING2 PHD finger complex with a histone H3 K4me_3_ peptide (Peña et al., 2006 Figure S2B). In the absence of such a cage, unmodified lysine is accommodated in a shallow groove of the PHD finger formed by a small hydrophobic core flanked by charged residues, as exemplified in the case of the TRIM24 complex with an H3K4 peptide (Tsai et al., 2010; Figure S2C). The architecture of the surface found on the ZMYND8 PHD finger resembles TRIM24, with a partial aromatic wall present (formed by F89) (Figures S2D and S2E). We therefore hypothesized that unmodified or, possibly, singly methylated lysines may be recognized by the ZMYND8 PHD module.

Structural alignment of the ZMYND8 PHD domain with the TRIM33 PHD/BRD tandem module in complex with a methylated histone H3 peptide (histone 3 trimethyl-lysine 3 [H3K9me_3_]) (Xi et al., 2011) revealed a different orientation of the PHD/BRD modules, suggesting simultaneous engagement of histone peptides with both modules (Figure S2F). In addition, although the TRIM33 topology was shown to be responsible for concurrent engagement of K9me_3_ and histone 3 lysine 18 acetyl (K18ac) on histone H3, the ZMYND8 topology orients the BRD module closer to histone 3 lysine 14 acetyl (H3K14ac). We therefore hypothesized that simultaneous engagement of histone peptides by the PHD and BRD modules may be possible and that histone 3 lysine 14 acetyl (K14ac) may be a target site of the ZMYND8 BRD module.

Structural overlay of the PWWP domain to the mouse ZMYND11 structure in complex with histone 3 isoform 3 trimethyl lysine 36 (H3.3K36me_3_) (Wen et al., 2014) revealed a similar arrangement of the BRD/PWWP modules, which are linked by a zinc finger in both proteins (Figure S2G). In addition, an aromatic cage (residues F288, W291, and F307) similar to ZMYND11 was found to be present, suggesting potential binding to methylated peptides. Intriguingly, the C-terminal tail of a crystallographic neighbor was found to be inserted into the cavity of the PWWP cage (Figure S3A). Superimposition of the mouse ZMYND11/histone 3 trimethyl lysine 36 (H3K36me_3_) complex established that the C-terminal tail residue K396 of ZMYND8 mimics H3K36me_3_ insertion within the cage, suggesting that ZMYND8 may be able to bind to unmodified lysine residues (Figure S3A).

Our structural analysis supports the notion that the triple reader assembly of ZMYND8 may be able to engage histone peptides. A schematic of such an possible interaction is shown in Figure S3B.

### ZMYND8 Binds to Histone Peptides In Vitro

**Figure 2:**
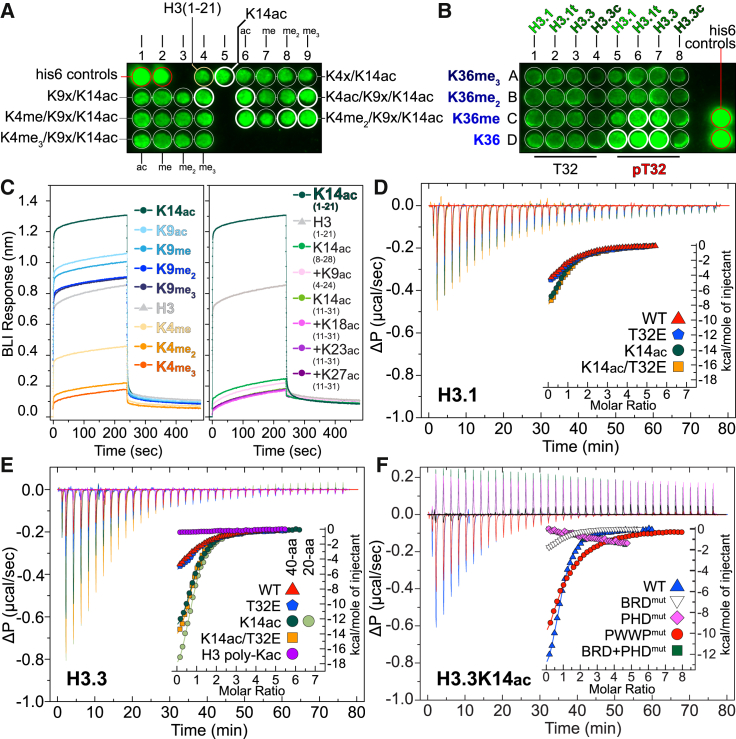
ZMYND8 Binds to Histone H3 In Vitro (A) Focused peptide SPOT array spanning lysine acetylation/methylation combinations within the first 21 residues of N-terminal histone H3 peptides. The presence of K14ac increases binding intensity (thick white circles). (B) Focused peptide SPOT array spanning residues 22–42 of all histone H3.x variants, carrying different methyl states of K36, in the absence or presence of pT32. (C) BLI of the recombinant triple reader ensemble profiled against 20-aa-long N-terminal histone H3 peptides carrying single PTMs as indicated in the inset (left). Unmodified H3 peptides bind to the triple reader modules (shown in gray). K9 modifications are tolerated, whereas K4 methylations break the interaction, and K14 acetylation greatly enhances it. Removal of the N-terminal sequence from peptides results in loss of binding irrespective of additional modifications (K9ac, K18ac, K23ac, and K27ac) present together with the central K14ac mark (right), suggesting that the N-terminal portion of H3, which engages the PHD domain, is essential for binding to ZMYND8. Experiments were carried out twice (n = 2) for each condition/peptide. (D) In-solution evaluation of histone H3.1 binding by ITC. Raw injection heats for titrations of modified peptides (carrying specific modifications as indicated in the inset) into a solution of ZMYND8 are shown. The inset shows the normalized binding enthalpies corrected for the heat of peptide dilution as a function of binding site saturation (symbols as indicated in the figure). Solid lines represent a nonlinear least-squares fit using a single-site binding model. Histone H3.1 lacking any modifications binds weakly (red triangle, K_D_ = 19.0 μM), whereas the addition of the phosphomimetic T32E has little effect (blue pentagon, K_D_ = 20.7 μM). Binding to K14ac is also weak (dark green circles, K_D_ = 15.1 μM), and the co-existence of the phosphomimetic T32E shows little effect (orange square, K_D_ = 17.5 μM). ITC titrations were carried out in triplicate (n = 3), and representative curves are shown. (E) In solution evaluation of histone H3.3. Data are presented as in (D). Histone H3.3 peptides lacking any modifications bind weakly (red triangle, K_D_ = 19.6 μM), whereas the addition of the phosphomimetic T32E has little effect on binding (blue pentagon, K_D_ = 17.4 μM). Binding is mainly driven by K14ac (dark and light green circles, K_D_ = 6.8 μM or 6.6 μM 40-mer and 20-mer, respectively), and the co-existence of the phosphomimetic T32E shows little effect (orange square, K_D_ = 6.2 μM). (F) In-solution evaluation of ZMYND8 carrying specific domain mutations to histone H3.3 binding by ITC. Data are presented as in (D). Mutation of the conserved asparagine (N228) on the bromodomain abolishes the interaction, as do mutations of conserved PHD residues (N87A, E104A, and D124A). Combination of PHD/BRD mutations has the same effect. Mutation of the conserved residues forming the PWWP cage (F288A and W291A) result in loss of affinity (WT ZMYND8, 6.8 μM; PWWP mutant, 23 μM).

To establish ZMYND8 interactions with histones, we next tested binding to histone peptides in vitro. Based on our structural analysis, we expected K14ac to engage the BRD module and K36 to engage the PWWP module. The positively charged surface lining the PWWP site toward the zinc finger (ZnF) and BRD modules was shown previously to bind to a phosphate ion in the ZMYND11 structure, in close proximity to T32 of histone H3 (Figure S3A). Threonine 32 has been found to be phosphorylated in a proteomic screen (Kettenbach et al., 2011); however, its biological role remains elusive. We interrogated binding by employing SPOT peptide arrays (Picaud and Filippakopoulos, 2015). Using 20-amino acid (aa)-long histone H3 peptides, we observed binding to most combinations containing a central K14ac modification (Figure 2A; Table S1). Histone H3 peptides spanning residues 22–42 also showed binding to histone H3.3K36 as well as K36me in the presence of pT32 (Figure 2B; Table S1), in agreement with our structural observations. We next systematically profiled histones H2A, H2B, H3, and H4, including their variant isoforms, employing 20-aa-long peptides carrying combinations of Kac, Kme_x_ (x = 1,2,3), pS, and pT (Figure S4A; Table S2). We observed strong binding to most peptides in the presence of multiple Kac modifications. Histone H3 isoforms as well as H4 exhibited higher binding intensities, suggesting stronger binding; however, we could not identify patterns suggestive of specificity toward certain combinations of PTMs.

To assess peptide binding in solution, we used biolayer interferometry (BLI) and profiled commercial libraries containing histone H2A, H2B, H3, and H4 N-terminally biotinylated peptides and found strong binding primarily to H3 and H4 peptides. Although H3K14ac exhibited the strongest signal, we also observed binding to unmodified H3 as well as to all methylation states of K9 (Figure 2C). Interestingly, although unmodified K4 peptides exhibited binding, K4 methylation decreased binding, in agreement with our structural observations (Figure S2A–S2E). Removal of the N-terminal amino acids of H3 resulted in complete loss of binding to all peptides tested irrespective of modifications incorporated (Figure 2C), suggesting that the PHD finger directly engages the N-terminal tail of the histone and is necessary for maintaining peptide interactions.

We next quantified peptide interactions employing BLI titrations and measured a dissociation constant of 27 μM against an H3K14ac peptide (Figure S4B); mutation of the conserved asparagine responsible for BRD/Kac histone interactions (Filippakopoulos et al., 2012) to a phenylalanine (N228F) weakened binding (Figure S4C). We systematically used BLI to interrogate binding to histone H3 modifications employing a set of custom peptides spanning 20 residues along the first 45 residues of each histone tail (Table S3). We observed binding in the presence of multiple Kacs, whereas combinations with Kme_x_, other than K4me_x_, systematically weakened binding (Figure S5). We also tested binding to H4 peptides by BLI and observed weak non-specific binding mainly driven by K12ac (Figure S6A–S6E). Isothermal titration calorimetry (ITC) helped establish precise thermodynamic parameters for peptide binding in solution. We prepared 40-aa-long recombinant H3.1, H3.3, and H4 peptides carrying site-specific acetylations deposited via amber codons (Neumann et al., 2009) and measured low-micromolar binding for most H3 peptides carrying K14ac, with a small advantage toward H3.3 (6.8 μM) over H3.1 (15.1 μM) sequences (Figures 2D and 2E). Histone H4 peptides showed lower affinity, driven by H4K12ac (31.6 μM) as well as poly-acetylated H4 (37.7 μM) (Figure S6F).

Taken together, our data suggest that ZMYND8 binds via the BRD module to peptides carrying Kac in vitro, whereas the PHD domain is necessary for interactions with the N-terminal tail of these peptides. In addition, ZMYND8 exhibits a slight preference toward H3.3 K14ac-containing peptides over H3.1 or H4 peptides while tolerating a number of adjacent modifications (Tables S4 and S5).

### Individual ZMYND8 Reader Domains Contribute to Histone Peptide Binding

Our structural analysis suggested that each of the three ZMYND8 reader modules may contribute to histone binding. Sequence alignments between different organisms showed high conservation of the N-terminal portion of ZMYND8 (Figure S7A). Structural comparison of each ZMYND8 domain to published peptide complexes (Peña et al., 2006, Wang et al., 2014) allowed identifying conserved residues within the PHD (N87, E104, and D124), BRD (N228), and PWWP (F288 and W291) domains, which should affect peptide binding (Figures S7A and S7B).

We tested binding to a 40-residue long histone H3 peptide carrying a K14ac epitope in solution by ITC using recombinant mutant proteins carrying the suggested mutations in each domain. Mutations in the PHD domain (Figures S7A and S7B) abolished peptide binding, as did mutation of the BRD domain (Figures S7A and S7B) or combination of PHD/BRD mutations (Figure 2F; Table S4). Interestingly, although mutation of the BRD resulted in loss of H4K12ac binding, mutation of the PHD had no effect, suggesting that engagement of the H4 tail is primarily initiated via the BRD module (Figures S6D and S6E; Table S5). Mutation of conserved aromatic residues found on the PWWP domain hydrophobic pocket (Figures S7A and S7B) resulted in a dramatic reduction in affinity toward the same peptide (Figure 2F; Table S4). Taken together, these data support a mode of ZMYND8 interaction with histone H3 peptides where all three domains simultaneously engage the first 40 N-terminal histone residues.

### ZMYND8 Engages Histones via Its Reader Ensemble in Cells

**Figure 3:**
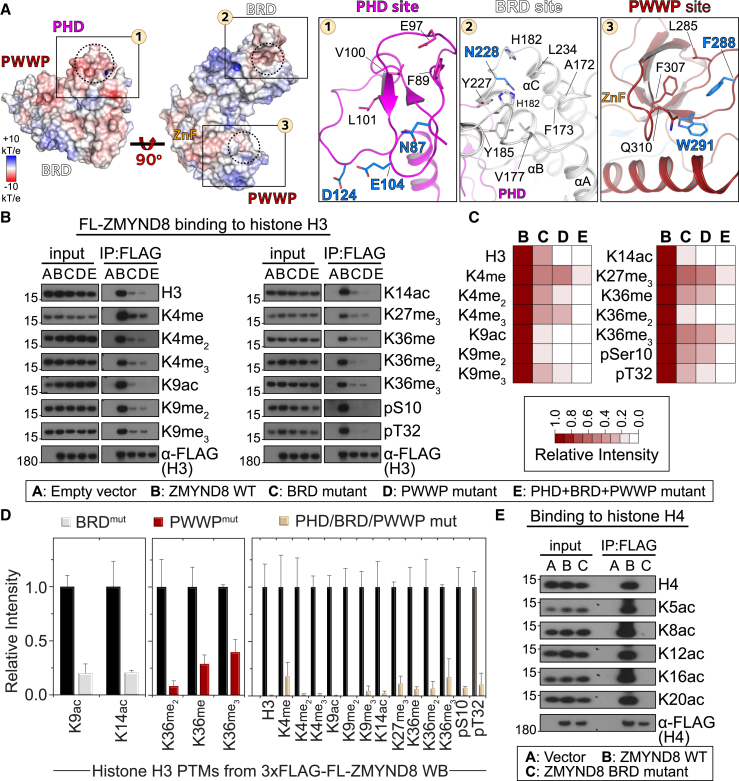
ZMYND8 Binds to Histone H3 in Cells through Its Reader Domains (A) Surface representation of the electrostatic potential distribution onto the triple reader ZMYND8 ensemble (scale as indicated in the inset), highlighting potential docking sites of histone peptides spanning the PHD, BRD, and PWWP domains (see also Figures S7A and S7B). Dotted circles highlight the cavities where histone residues would insert. Details shown on the right highlight the residues implicated in peptide binding as well as critical residues (highlighted in blue) that were further mutated to probe ZMYND8-histone interactions. (B) HEK293 cells transiently transfected with 3×FLAG-ZMYND8 WT or mutants (BRD mutant, N228F; PWWP mutant, F288A/W291A; PHD+BRD+PWWP mutant, N87A/E104A/D124A/N228F and F288A/W291A) were used to interrogate binding to histone H3. The WT protein recognizes all marks tested, whereas mutations on the interacting interface of the three modules result in gradual loss of binding. Mutated residues are annotated in (A). (C) Western blot quantification demonstrating the effect of ZMYND8 mutations on recognition of histone H3 marks from (B). Relative intensity is as annotated in the inset. (D) Quantification of the effect of BRD mutation (left), PWWP mutations (center), or PHD/BRD/PWWP mutations (right) on histone H3 marks. Although BRD mutations primarily affect recognition of acetylation at H3K9/K14ac and PWWP mutations affect H3K36me_x_ states, simultaneous mutation of all three reader modules results in more than 85% loss of histone H3 binding for all marks tested. Data represent mean ± SEM from three biological replicates of the WBs shown in (B). (E) HEK293 cells transiently transfected with 3×FLAG-ZMYND8 WT or BRD mutant (N228F) were used to interrogate binding to histone H4 marks. Acetylation of H4 can be recognized by the protein, but mutation of the BRD module results in total loss of the interaction.

Having established that ZMYND8 uses the three reader domains to engage histone H3 in vitro, we next asked whether the protein engages histone H3 in cells. We used full-length constructs of ZMYND8 WT or mutated on the BRD module (N228F), PWWP module (F288A/W291A), or all three reader modules (PHD, N87A/E104A/D124A; BRD, N228F; PWWP, F288A/W291A) (Figure 3A). We tested binding to histone H3 in HEK293 cells transfected with 3×FLAG-tagged variants (WT or mutant constructs) by performing immunoprecipitation with anti-FLAG followed by immunoblotting against specific H3 antibodies (Figure 3B). We were surprised to find all histone modifications tested to be enriched in the case of WT ZMYND8, whereas mutations in the BRD or PWWP domains strongly diminished histone binding. Importantly, when all three reader modules were mutated, binding to every histone H3 mark tested was lost. Quantification suggested that BRD mutations primarily affected K9ac/K14ac enrichment, whereas PWWP mutations affected and K36me_x_ binding (Figures 3C and 3D); mutation of all three reader domains reduced binding by more than 80% toward any H3 mark. Similar results were obtained in the case of H4 binding, where all acetylation marks were bound by the WT protein. Mutations introduced in the BRD module completely abolished H4 interactions, in agreement with our in vitro data, suggesting that H4 binding relies solely on the BRD rather than on all three reader modules of ZMYND8 (Figure 3E).

Taken together, our data suggest that ZMYND8 relies on all three reader modules to engage histone H3 in cells, whereas binding to histone H4 mainly occurs through the BRD module.

### ZMYND8 Engages DNA and Chromatin

**Figure 4:**
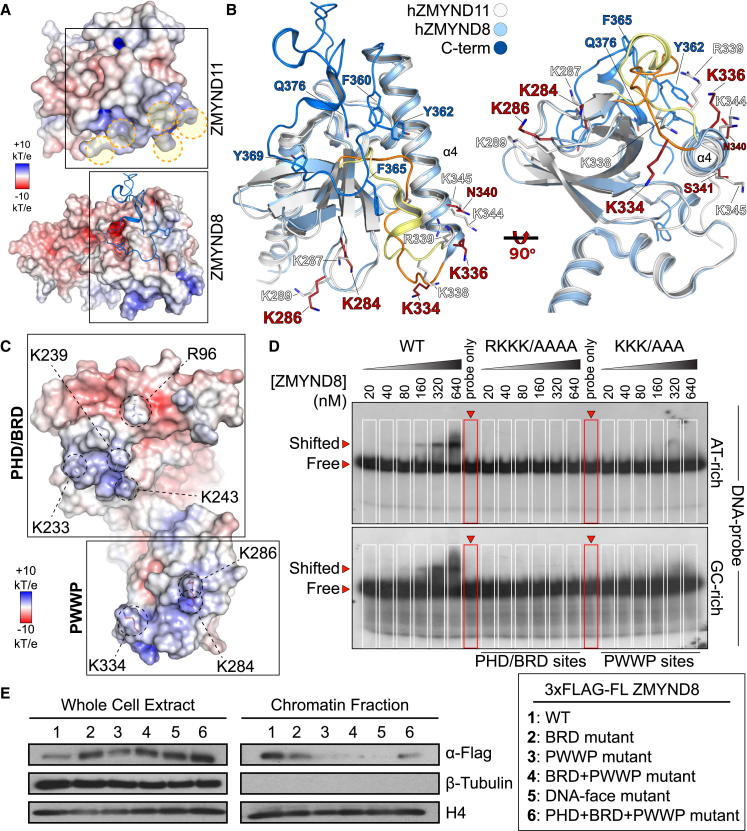
ZMYND8 Binds to DNA and Nucleosomes (A) Detail of the PWWP domains of human ZMYND11 (PDB: 4NS5) and ZMYND8, showing positively charged areas (highlighted with orange dotted circles). Structures are oriented so that the bottom of the PWWP domain is visible, with residue electrostatic potential plotted on the surface of each protein, as indicated in the inset. (B) Detail of the boxed regions shown in (A) in ribbon representation, as indicated in the inset, highlighting the structural implications of the C-terminal portion of the hZMYND8 structure. The loop region connecting β8 and α4 in the PWWP domain (hZMYND11, orange; hZMYND8, pale yellow) adopts a helical turn in hZMYND8 as a result of the C-terminus packing under the PWWP domain via aromatic residues (Y362, F365, and Y369 shown in blue), resulting in a small extension of helix α4. Repositioning of the loop between β8 and α4 results in K338 (in hZMYND8) overlaying with K334 (in hZMYND11), and the loop between β3 and β4 (K287/K289 in hZMYND8 and K284/K286 in hZMYND11) suggests a role in maintaining contacts with DNA. Rotation of the superimposed structures by 90° (right) reveals a different orientation of the positive residues in each structure, suggesting different topologies when interacting with nucleosomes. (C) DNA binding interfaces identified on the PHD/BRD modules (R96, K233, K239, and K243) and PWWP module (K284, K286, and K334) mapped onto the charged surface of the reader ensemble (scale as indicated in the inset). (D) The recombinant WT reader ensemble can shift a radioactive AT-rich (top) or GC-rich (bottom) DNA probe. Alanine mutations (guided by the model shown in A) introduced on the PHD/BRD (RKKK/AAAA) or the PWWP (KKK/AAA) interface abolish DNA interactions in EMSAs. (E) Full-length ZMYND8 associates with chromatin, whereas mutations of its readers or its DNA-binding face result in loss of binding to chromatin. Full-length 3×FLAG ZMYND8 WT or mutants (as indicated in the inset) were transiently transfected into HEK293 cells, and the whole-cell extract and chromatin-associated fraction were analyzed for 3×FLAG-ZMYND8 by western blot.

We found the triple reader ensemble of ZMYND8 engaging histone peptides in vitro and in cells, suggesting that proximity to the nucleosome core might involve interactions with DNA. Previously, ZMYND11 structural features were linked to DNA interactions (Wang et al., 2014, Wen et al., 2014), prompting us to compare the ZMYND8 triple ensemble with the BRD/PWWP ZMYND11 structure (Wang et al., 2014). We found differences in the distribution of charge on the back side of these modules, opposite the aromatic cage responsible for Kme_x_ binding (Figure 4A). Closer inspection of the two structures revealed only four positively charged patches on ZMYND8, as opposed to five on ZMYND11. A number of charged residues lining up the PWWP aromatic cage (K287, K289, K338, R339, K344, and K345) of ZMYND11 were previously implicated in nonspecific recognition of DNA (Wang et al., 2014). Although most of these residues are present in ZMYND8 (K284, K286, K334, and K336) and structurally align between proteins, the K344/K345 motif found in ZMYND11 is replaced by N340/S341 in ZMYND8 (Figure 4B; Figure S1A). In addition, the K338/R339 patch found in ZMYND11 is located in a loop region connecting β8 and α4 in the PWWP domain; however, the C-terminal PWWP loop extension of ZMYND8 forces this loop region to distort compared to ZMYND11, resulting in repositioning of K334/K336, thus forming a small extension of helix α4. This topology suggests that the α4 “face” of the human ZMYND8 PWWP domain may not be able to engage DNA. Repositioning of the loop between β8 and α4 results in K338 (ZMYND8) overlaying with K334 (ZMYND11), suggesting that this loop maintains contacts with DNA, as does the loop between β3 and β4 (K287/K289 in ZMYND8 and K284/K286 in ZMYND11).

In addition to the positively charged PWWP face, we identified positively charged patches on the surface of the BRD and PHD domains (Figure 4C). Systematic mutation of conserved residues on each interface (annotated in Figure S7A; PHD/BRD, R96A/K233A/K239A/K243A; PWWP, K284A/K286A/K334A) allowed testing the ability of recombinant constructs to interact with DNA in Electrophoretic Mobility Shift Assays (EMSA) (Garner and Revzin, 1981). Although the WT protein was able to shift AT-rich and GC-rich radioactive oligonucleotide probes, the two DNA face mutants lost their ability to interact with DNA (Figure 4D). Importantly, mutation of the two DNA-face interfaces in the full-length protein abrogated interactions with all H3 and H4 PTMs in HEK293 cells (Figures S8A and S8B); however, mutation of the histone-binding interfaces within each domain did not affect binding to DNA in EMSA assays (Figure S8C).

To verify that ZMYND8 simultaneously interacts with histones and DNA, directly engaging nucleosomes, we employed a chromatin fractionation assay (Rothbart et al., 2012a) using HEK293 cells transfected with full-length 3×FLAG-tagged ZMYND8, WT, or mutants that abolish histone or DNA interactions. Western blot (WB) analysis of the chromatin-associated fraction by anti-FLAG revealed a strong signal for the WT protein; mutations of the reader domains affecting histone binding or of the DNA-binding face of the protein resulted in dramatic reduction or loss of ZMYND8 in the chromatin-associated fraction. Taken together, our data suggest that ZMYND8 is tethered to chromatin, initiating multivalent interactions resulting in combinatorial readout of histones and DNA.

### ZMYND8 Co-localizes with H3K14ac at Transcriptional Start Sites and Enhancers

**Figure 5:**
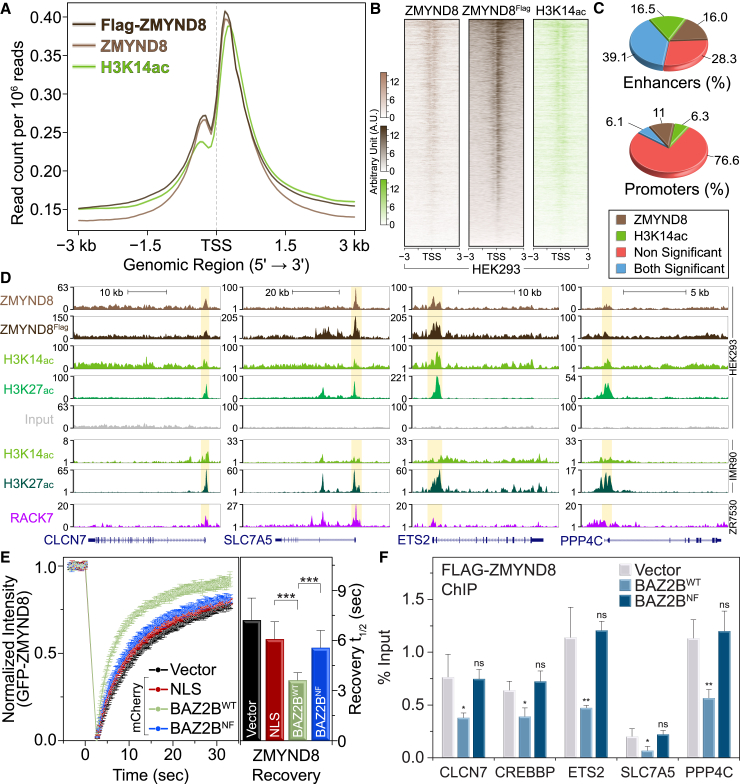
ZMYND8 Co-localizes with H3K14ac at Transcriptional Start Sites and Enhancers (A) Average profile of ZMYND8, FLAG-ZMYND8, and H3K14ac ChIP-seq signals on ± 3 kb around transcriptional start sites in HEK293 cells. Representative distributions from biological replicates (n = 2) are shown. (B) Heatmaps of ChIP-seq signals on ± 3 kb surrounding the TSS of genes bound to ZMYND8 or FLAG-ZMYND8 and H3K14ac in HEK293 cells. (C) Distribution of tag densities for ZMYND8 and K14ac around enhancer (top) and promoter (bottom) regions showing significant overlap according to p values calculated relative to local background. (D) ChIP-seq analysis of ZMYND8 and H3K14ac in HEK293 cells for selected genes. Signal enrichment is compared with the published enrichment signals for H3K14ac (GSM521883) and H3K27ac (GSM521887) in IMR90 cells as well as ZMYND8 in ZR-75-30 cells (GSE71323). (E) FRAP experiments following masking of H3K14ac in U2OS cells by transfection with mCherry-3xBAZ2B (WT or N/F mutant) constructs. Competition for H3K14ac by BAZ2B^WT^ results in faster recovery of ZMYND8, whereas the inactive BAZ2B^N/F^ mutant has no effect on ZMYND8 recovery, demonstrating direct competition for K14ac in cells. Data represent the mean ± SEM (n = 15). Right: quantitative comparison of time with half-maximal fluorescence recovery for GFP-ZMYND8. ^∗∗∗^p < 0.005. (F) ChIP-qPCR validation of FLAG-ZMYND8 over K14ac-bound loci in HEK293 cells stably expressing 3×FLAG-ZMYND8^WT^ following competition with HA-3xBAZ2B^WT^ or HA-3xBAZ2B^NF^. Masking of H3K14ac by HA-3xBAZ2B^WT^ resulted in significant reduction of ZMYND8 occupancy at these loci. Data represent mean ± SD from biological triplicates (n = 3). ^∗∗^p < 0.01, ^∗^p < 0.05. ns, not significant.

We next investigated the genome-wide distribution of ZMYND8 in HEK293 cells together with H3K14ac, which we found to be the most potent determinant of histone binding in vitro, employing chromatin immunoprecipitation followed by sequencing (ChIP-seq). We found ZMYND8 signals enriched around transcriptional start sites (TSSs) together with H3K14ac (Figures 5A and 5B), and tag density analysis identified 39.1% significant overlap with H3K14ac signals at enhancer elements (Figure 5C; Figures S9A and S9B). ZMYND8 signals also correlated well with a recently reported dataset in breast ZR-75-30 cells (Shen et al., 2016) at enhancers (Figures S9C and S9D), and ZMYND8 occupancy around TSSs was shown in Genome Browser Tracks (Figure 5D). Small interfering RNA (siRNA) depletion of histone acetyl-transferases controlling K14ac (P300, PCAF, and ADA2a) (Feller et al., 2015, Jin et al., 2011, Nagy et al., 2010) reduced protein levels of this mark (Figure S9E) and showed loss of occupancy at the TSS of genes, resulting in reduction of ZMYND8 at the same loci, albeit at variable levels (Figure S9F). However, small interference RNA targeting an histone acetyl-transferase (siHAT) also resulted in changes of methylation states of H3, making it difficult to directly assign a role for K14ac-specific recruitment of ZMYND8.

To better understand K14ac contributions to ZMYND8 localization, we engineered a “K14ac-masking” system, employing the BRD of BAZ2B, which we found previously to preferentially recognize H3K14ac in vitro (Filippakopoulos et al., 2012). Using SPOT assays, we first confirmed binding only to K14ac and measured low micromolar affinity (Table S6) in solution by ITC (Figures S10A and S10B). Mutation of the BAZ2B conserved asparagine (N2140) resulted in complete loss of binding in solution (Figures S10B and S10C). Constructs carrying three BAZ2B BRD modules were able to pull H3K14ac from HEK293 cells, whereas introduction of the N2140F mutation abolished this interaction (Figure S10C). We employed a fluorescent recovery after photobleaching (FRAP) technique to disrupt the interaction of mCherry-tagged 3×BAZ2B (WT or N/F mutant) with H3K14ac and observed displacement of the mutant protein from chromatin, as evidenced by the fast recovery after bleaching (Figures S10D and S10E), whereas the WT protein did not accumulate at the laser-damaged site (Figure S10F). Taken together, these data established the BAZ2B 3×BRD system as a mask for H3K14ac in cells. We next used this K14ac-masking tool in a U2OS Flp-In/TRex system stably expressing GFP-ZMYND8 using a FRAP assay to displace ZMYND8 from chromatin. In the presence of BAZ2B^WT^, we observed fast recovery after bleaching, suggesting removal of ZMYND8 from chromatin, whereas the mutant BAZ2B^NF^ masking did not have this effect (Figure S10G; Figure 5E), thus establishing that K14ac masking results in higher mobility of ZMYND8. We next performed ChIP assays at the loci shown previously to be affected by siHAT depletion of K14ac. In agreement with the FRAP data, BAZ2B^WT^ resulted in loss of the ZMYND8 ChIP signal, whereas BAZ2B^NF^ did not have an effect (Figure 5F). Taken together, our data suggest that H3K14ac is a key determinant for ZMYND8 recruitment to chromatin, in agreement with our in vitro and in-cell observations.

### ZMYND8 Recruitment to DNA-Damaged Sites Is Controlled by Histone and DNA Interactions

**Figure 6:**
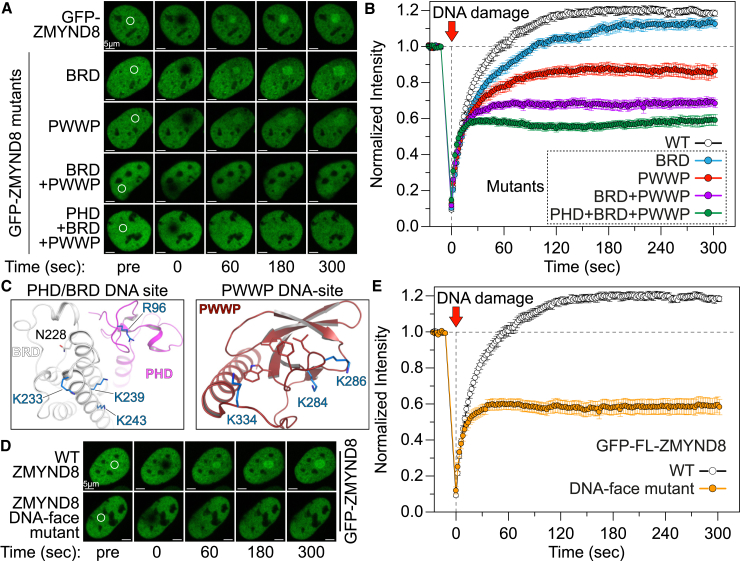
ZMYND8 Reader Interactions Control Recruitment to DNA Damage Sites (A and B) U2OS cells transfected with GFP-tagged ZMYND8 WT or mutants were photobleached (small area annotated with a white circle) in FRAP experiments (A). The WT protein is rapidly recruited to the bleached site, whereas mutations of the reader modules result in gradual loss of the protein’s ability to recruit onto the damaged site (B). (C) Detail of the PHD/BRD (left) and PWWP (right) interfaces highlighting residues that can potentially bind to DNA. (D and E) U2OS cells transfected with GFP-tagged ZMYND8 WT or DNA-face mutant (involving simultaneous mutation of the seven residues highlighted in C) were photobleached in FRAP experiments (D). The WT protein is rapidly recruited to the bleached site, whereas mutation of DNA-interacting residues results in significant loss of the proteins ability to recruit onto the damaged site (E). The data in (B) and (E) represent the mean ± SEM from multiple experiments in different cells (n = 15).

ZMYND8 has been shown to act as a DNA damage response protein, recruited to DNA-damaged sites as a function of its BRD interaction with histone H4 (Gong et al., 2015). We next tested the contribution of each reader module of ZMYND8 to recruitment onto damaged DNA sites. We employed a FRAP assay in U2OS cells transiently transfected with full-length GFP-ZMYND8^WT^ or mutants. ZMYND8^WT^ was actively recruited to a bleached nuclear region after ∼60 s; however, mutations systematically introduced to the reader ensemble gradually reduced recruitment to the DNA-damaged sites (Figures 6A and 6B), suggesting that each reader module contributes to the recruitment process. Introduction of mutations on the DNA-face sites of ZMYND8 (Figure 6C) resulted in a similar loss of recruitment onto damaged sites, suggesting that both histone- and DNA-initiated interactions are necessary for ZMYND8 interactions with these sites (Figures 6D and 6E).

### Loss of Reader Function Perturbs the ZMYND8 Interactome

**Figure 7:**
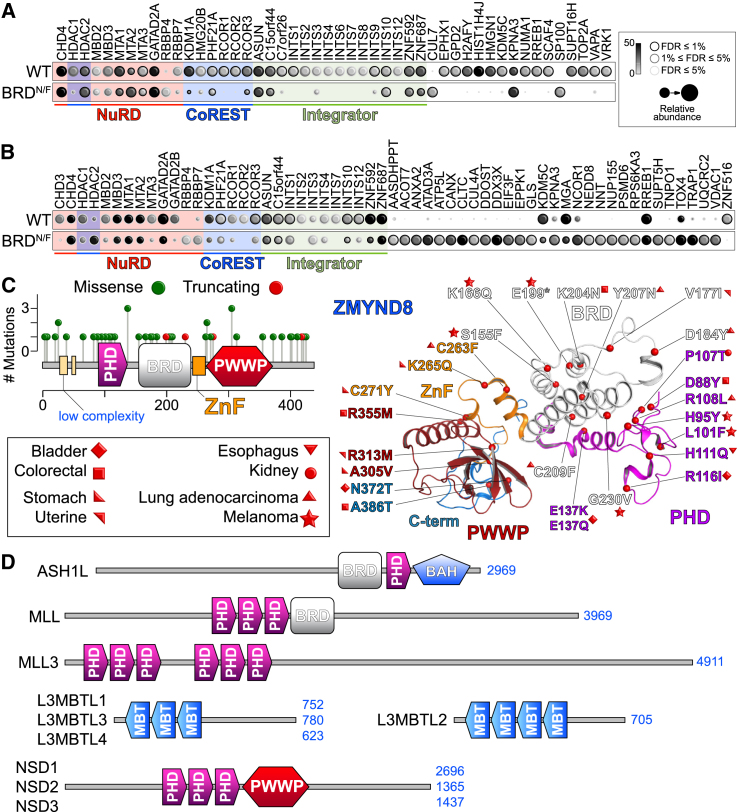
ZMYND8 Reader Interactions Control the Assembly of Transcriptional Complexes (A) AP-MS of full-length 3×FLAG WT or BRD^N/F^ ZMYND8 from HEK293 cells highlighting the changes in the ZMYND8 interactome following disruption of binding to acetylated histones by mutating the conserved BRD asparagine (N228) to a phenylalanine. The data represent the mean of biological replicate experiments (n = 2). Spectral counts of each prey proteins are displayed as a gray scale in the legend. (B) Proximity biotinylation coupled to mass spectrometry of full-length BirA^∗^-FLAG WT (top) or BRD mutant (bottom) ZMYND8 in HEK293 cells. Mutation of the BRD module results in partial loss of enrichment of many components found to interact by AP/MS as well as gain of interaction with several new proteins. The data represent the mean of biological replicate experiments (n = 2). (C) Human ZMYND8 mutations and expression in cancer. Mutations annotated by the TCGA consortium found on the triple reader modules of ZMYND8 are annotated on the structure, colored according to the domain to which they belong, and classified by cancer type. (D) Domain organization of proteins that contain multiple reader domains in serial arrangements, potentially adopting multivalent assemblies that can engage distinct histone or nucleosome states.

We next asked whether multivalent interactions are also responsible for ZMYND8 participation within protein interaction networks through chromatin associations. First, we established the broader ZMYND8 interactome using two orthogonal approaches, affinity purification (AP) and proximity biotinylation (BioID) coupled to mass spectrometry (MS) (Lambert et al., 2015). Using both AP-MS (Figure 7A) and BioID-MS (Figure 7B), we found ZMYND8 to interact with components of transcriptional complexes, including the nucleosome remodeling and de-acetylase complex (NuRD), the co-repressor of the RE1-silencing transcription factor complex (CoREST), as well as the integrator complex. Mutation of the BRD module resulted in weaker interactions with these complexes, as exemplified by the lower number of peptides identified (Figures 7A and 7B). We also observed gain of interactions (particularly with BioID) with several new components that did not seem to be functionally linked.

Functional enrichment analysis followed by annotation and clustering of the AP-MS network components confirmed a systematic loss of interactions with chromatin-associated functions upon mutation of the BRD domain for all statistically significant biological processes (Figure S11A) and cellular components (Figure S11B). Similarly, loss of enrichment was observed in biological processes enriched by the BioID set of genes (Figure S11C), whereas there was gain of function toward non-chromatin-related cellular components, as exemplified by interactions with mitochondrial proteins (Figure S11D), suggesting that disruption of the chromatin association of ZMYND8 may be affecting its sub-cellular localization and its interaction network.

## Discussion

It has been proposed that multivalent binding results in higher specificity accompanied by affinity enhancement and loss of translational/rotation freedom to gain favorable entropy (Ruthenburg et al., 2007). Interactions with modified histone termini are usually weak (100–300 μM); however, reader modules have evolved to simultaneously engage multiple PTMs, resulting in single-digit micromolar interactions (Filippakopoulos et al., 2012, Morinière et al., 2009). Combination of multiple reader modules evolved to confer avidity and further specificity, resulting in single- to double-digit micromolar affinities, as exemplified by interactions of tandem BRDs in TAF1 (Jacobson et al., 2000) or tandem PHD/BRD modules in TRIMs (Ruthenburg et al., 2011, Tsai et al., 2010, Xi et al., 2011). Direct interactions with DNA have also evolved and are usually found in larger complexes, such as the regulator of chromosome condensation (RCC1), which directly binds nucleosomal DNA (Makde et al., 2010), the RPC1 ubiquitination module, which binds directly to the core nucleosome (McGinty et al., 2014), or LSD1-CoREST, which engages the nucleosome core between two turns of the DNA superhelix (Yang et al., 2006).

Here we report the structure of the triple N-terminal reader modules found in ZMYND8, which form a stable ensemble capable of simultaneously engaging histones and DNA. Binding is mainly driven by H3K14ac in vitro, and we find ZMYND8 to be associated with this mark in cells, whereas other histone modifications seem to be tolerated, with the exception of higher lysine methylation states. Two recent studies suggested that H4K16ac is a main point of histone H4 contact based on antibody evidence (Adhikary et al., 2016, Gong et al., 2015); however, our in vitro data clearly show that H4K12ac is the main driver of this interaction, which is much weaker than H3K14ac, whereas our antibody staining showed enrichment of all H4 acetyl states tested. Masking of H3K14ac using multiple bromodomains of BAZ2B destabilized the chromatin complex of ZMYND8, as evidenced by shorter recovery times in FRAP experiments, while removing the protein from K14ac-rich loci, as evidenced by ChIP-PCR assays.

Interaction with the entire N-terminal histone tail may result in “shielding,” preventing remodeling, a topology that we find by molecular modeling to be possible (see Supplemental Information and Figure S12); we also find enzymes such as HDAC1, KDM1, and KDM5C participating in the ZMYND8 interaction network, which can potentially maintain the PTM landscape around ZMYND8 binding sites. Indeed, a recent study demonstrated that the demethylase KDM5C maintains a H3K4me1 state when recruited by ZMYND8 at its target enhancers (Shen et al., 2016). Perturbation of any of the interacting interfaces of the ZMYND8 reader ensemble does not completely dissociate the protein from chromatin, possibly allowing the system to return to an equilibrium state. However, simultaneous disruption of multiple points of contact (e.g., deacetylation of K14, methylation of K4) may result in dissociation from chromatin and destabilization of the ZMYND8 intricate network of interactions, possibly resulting in protein relocation. Indeed, in agreement with previous reports, we found ZMYND8 to be associated with tightly regulated machinery, including the CoREST and integrator complexes (Malovannaya et al., 2011) as well as the NuRD complex (Eberl et al., 2013, Havugimana et al., 2012, Kloet et al., 2015). It was reported previously that ZMYND8 is recruited to DNA damage sites through the NuRD component CHD4 (Gong et al., 2015). Here we show that perturbation of the reader functions of ZMYND8 severely reduces its ability to recruit to DNA in response to DNA damage. Interestingly, we found that, although H4 interactions are abolished when the BRD is mutated, H3 interactions are only weakened but not lost, suggesting a different mode of histone tail engagement between histones.

Intriguingly, inspection of data reported by The Cancer Genome Atlas (TCGA) consortium (extracted from cBioPortal in November 2015; Gao et al., 2013; http://www.cbioportal.org/) revealed that 25% of ZMYND8 mutations occur within the reader ensemble, with enriched sites on the PHD and PWWP modules. Mapping of the mutated sites onto the crystal structure suggests that most, if not all, affect binding to histones either by disrupting the interaction interface or by destabilizing the domain topology (Figure 7C).

Our work provides a proof of concept linking structural rigidity with simultaneous DNA and histone interactions to maintain recognition of chromatin states in the absence of conformational flexibility. The relative weak binding exhibited by ZMYND8 could result in the maintenance of a transient binding state to chromatin, allowing for early association with establishing patterns of PTMs during chromatin landscape remodeling. The conformational rigidity present in the ZMYND8 triple reader ensemble could offer protection against random changes in the same PTM landscape, resulting in deposition or removal of marks that would destabilize or alter transcriptional programs. However, several other proteins contain multi-reader organizations, potentially offering topologies similar to the ZMYND8 triple reader ensemble or allowing re-assembly in three dimensional clusters that offers similar characteristics (Figure 7D). More work is needed to define the characteristics of chromatin engagement as a function of plasticity involving multivalent protein assemblies, seeking to identify shared and distinct mechanisms that help relay integrated signaling cascades leading to specific transcriptional programs.

## Experimental Procedures

### Cloning, Mutagenesis, and Stable Cell Lines

ZMYND8 (Uniprot: Q9ULU4, isoform 13) WT, or mutant was cloned into pDONR221, pDEST 5′ Triple-FLAG-pcDNA5-FRT-TO, or pDEST 5′ BirA^∗^-FLAG-pcDNA5-FRT-TO vectors; the PHD/BRD/PWWP cassette was cloned into the pNIC-ZB vector (GenBank: GU452710). Proteins of interest were stably expressed in T-REx Flp-In HEK293 cells as described previously (Couzens et al., 2013; Supplemental Experimental Procedures).

### Protein Expression and Purification

Recombinant proteins were expressed and purified by affinity and size exclusion chromatography as described previously (Savitsky et al., 2010).

### Recombinant Histone Peptides

Recombinant histone peptides carrying specific acetylations were produced in vivo using the amber stop codon/suppressor tRNA technology (Neumann et al., 2009) with several modifications that increased overall yields from 10% to above 70% (Supplemental Experimental Procedures).

### SPOT Peptide Assays

Human histone peptides were synthesized on cellulose arrays according to the SPOT synthesis described previously (Filippakopoulos et al., 2012).

### BLI

Experiments were performed on an Octet RED384 system using commercial libraries (AltaBioSciences) or peptides synthesized as described previously (Rothbart et al., 2012b).

### ITC

Experiments were carried out on an ITC200 titration microcalorimeter, and data analysis was performed using the MicroCal Origin software as described previously (Filippakopoulos et al., 2012); data are presented in the Supplemental Information.

### Crystallization, Data Collection, and Structure Determination

Purified recombinant protein was crystallized at 4°C. Zinc single wavelength anomalous dispersion phasing (Zn-SAD) data were collected at Diamond Lightsource. Identification of three putative Zn sites in the SAD dataset allowed phasing, followed by manual model building and refinement as explained in the Supplemental Experimental Procedures.

### Chromatin Fractionation Assay

Chromatin fractionation assays were performed with HEK293T cells transfected with ZMYND8 WT or mutant plasmids as described previously (Rothbart et al., 2012a).

### Histone Immunoprecipitation

Cells were lysed, chromatin was digested, and co-immunoprecipitation was performed using antibodies described in the Supplemental Experimental Procedures.

### FRAP

FRAP experiments were performed using a protocol modified from previous studies (Filippakopoulos et al., 2010) as described in the Supplemental Experimental Procedures.

### EMSA

Oligonucleotide probes encoding AT-rich or GC-rich sequences were used together with recombinant ZMYND8 constructs (WT or mutant) to determine protein binding in electrophoresis experiments. For details, see the Supplemental Experimental Procedures.

### Mass Spectrometry

Samples were analyzed by mass spectrometry in liquid chromatography-tandem MS experiments on a TripleTOF 5600 instrument. AP-MS as well as BioID-MS were performed as described previously (Lambert et al., 2014, Lambert et al., 2015).

### RNAi

Small RNAi was used to knock down ZMYND8 or HATs in HEK293 cells expressing inducible 3×FLAG-ZMYND8. For details, see the Supplemental Experimental Procedures.

### ChIP and ChIP-Seq

Details regarding chromatin preparation, ChIP, and ChIP-PCR can be found in the Supplemental Experimental Procedures. Library construction and sequencing were performed at the Wellcome Trust Centre for Human Genetics using the PrepX Complete ILMN 32i DNA Library Kit (Illumina) and Illumina adapters. Purified DNA was analyzed by deep sequencing on the Illumina HiSeq 2500 platform.

### ChIP-Seq Data Analysis

Reads were mapped to hg19 (NCBI build 37) using BWA-MEM, allowing up to two mismatches. Genome ChIP-seq profiles were generated using HOMER (Heinz et al., 2010), and enrichment profiles were visualized using ngs.plot (Shen et al., 2014) as described in the Supplemental Experimental Procedures.

### Statistical Methods

For FRAP experiments, mean values and SEM were determined from 15 cells per condition tested over multiple experiments, and *P value*s were calculated using two-tailed Student’s t test or one-way ANOVA followed by Dunnett’s multiple comparison test. ChIP-qPCR *P value*s were determined using one-way ANOVA followed by Dunnett’s multiple comparison test on biological replicates (n = 3). In AP-MS and BioID-MS experiments, data were analyzed with SAINTexpress to calculate the probability value of each potential protein-protein interaction from background contaminants using default parameters. Controls were compressed to six samples, and a false discovery rate (FDR) of 1% or lower was required for proteins to be classified as significant interaction partners of ZMYND8.

## Author Contributions

P.S. prepared recombinant proteins, designed and cloned full-length ZMYND8 constructs and mutants, and performed crystallization trials. P.S. and S.P. designed, carried out, and analyzed biophysical experiments. T.K. performed crystallographic studies. T.F. carried out FRAP studies and immunoblotting. J.P.L established stable cell lines. J.P.L. and A.C.G. designed and performed proteomic experiments and analyzed data. E.S., B.S., and K.K. prepared recombinant peptides and performed chromatin fractionation assays. T.F., H.F., and C.G. designed and performed EMSA assays. C.Y.W. performed ChIP-seq assays. C.Y.W. and T.F. performed ChIP-qPCR assays. A.K., A.S., and P.F. analyzed ChIP-seq data. P.F. designed and supervised the study and prepared the manuscript. All authors read and commented on the manuscript.

## Accession Numbers

The accession number for the model and structure factors of the ZMYND8 PHD/BRD/PWWP cassette reported in this paper is PDB: 4COS. The accession numbers for the mass spectrometry and high-throughput sequencing data reported in this paper are MassIVE: MSV000079336 and GEO: GSE81696, respectively.

## Supplemental Information

Document S1. Supplemental Experimental Procedures, Figures S1–S12, and Tables S3–S9

Table S1. Histone Arrays of Peptide Sequences Used in the Membranes Shown in Figure 2, Related to Figure 2

Table S2. Histone Arrays of Peptide Sequences Used in the Membranes Shown in Figure 4, Related to Figure 4

Document S2. Article plus Supplemental Information
