# Supplementary material for: Multivalent Histone and DNA Engagement by a PHD/BRD/PWWP Triple Reader Cassette Recruits ZMYND8 to K14ac-Rich Chromatin
Source: Cell Rep. 2016 Dec 6;17(10):2724–37. doi: 10.1016/j.celrep.2016.11.014 (PMC5177622; doi:10.1016/j.celrep.2016.11.014)
Supplement: Table S2. Histone Arrays of Peptide Sequences Used in the Membranes Shown in Figure 4, Related to Figure 4 [file mmc3.pdf]

Histone	Peptide Length	Array Poition	Kac Mark Combinations	Sequence																			
polyHis CTRL		A1	HHHHHHHH																				
polyHis CTRL		A2	HHHHHHHH																				
		A3	Blank																				
H2A (Q6FI13)	1-20	A4	CONTROL	S	G	R	G	K	Q	G	G	K	A	R	A	K	A	K	S	R	S	S	R
		A5	pS1	pS	G	R	G	K	Q	G	G	K	A	R	A	K	A	K	S	R	S	S	R
		A6	K5me	S	G	R	G	Kme	Q	G	G	K	A	R	A	K	A	K	S	R	S	S	R
		A7	K5me2	S	G	R	G	Kme2	Q	G	G	K	A	R	A	K	A	K	S	R	S	S	R
		A8	K5me3	S	G	R	G	Kme3	Q	G	G	K	A	R	A	K	A	K	S	R	S	S	R
		A9	K5ac	S	G	R	G	Kac	Q	G	G	K	A	R	A	K	A	K	S	R	S	S	R
		A10	K9me	S	G	R	G	K	Q	G	G	Kme	A	R	A	K	A	K	S	R	S	S	R
		A11	K9me2	S	G	R	G	K	Q	G	G	Kme2	A	R	A	K	A	K	S	R	S	S	R
		A12	K9me3	S	G	R	G	K	Q	G	G	Kme3	A	R	A	K	A	K	S	R	S	S	R
		A13	K9ac	S	G	R	G	K	Q	G	G	Kac	A	R	A	K	A	K	S	R	S	S	R
		A14	K13me	S	G	R	G	K	Q	G	G	K	A	R	A	Kme	A	K	S	R	S	S	R
		A15	K13me2	S	G	R	G	K	Q	G	G	K	A	R	A	Kme2	A	K	S	R	S	S	R
		A16	K13me3	S	G	R	G	K	Q	G	G	K	A	R	A	Kme3	A	K	S	R	S	S	R
		A17	K13ac	S	G	R	G	K	Q	G	G	K	A	R	A	Kac	A	K	S	R	S	S	R
		A18	pS1/K5me	pS	G	R	G	Kme	Q	G	G	K	A	R	A	K	A	K	S	R	S	S	R
		A19	pS1/K5me2	pS	G	R	G	Kme2	Q	G	G	K	A	R	A	K	A	K	S	R	S	S	R
		A20	pS1/K5me3	pS	G	R	G	Kme3	Q	G	G	K	A	R	A	K	A	K	S	R	S	S	R
		A21	pS1/K5ac	pS	G	R	G	Kac	Q	G	G	K	A	R	A	K	A	K	S	R	S	S	R
		A22	K5me/K9me	S	G	R	G	Kme	Q	G	G	Kme	A	R	A	K	A	K	S	R	S	S	R
		A23	K5me/K9me2	S	G	R	G	Kme	Q	G	G	Kme2	A	R	A	K	A	K	S	R	S	S	R
		A24	K5me/K9me3	S	G	R	G	Kme	Q	G	G	Kme3	A	R	A	K	A	K	S	R	S	S	R
		A25	K5me/K9ac	S	G	R	G	Kme	Q	G	G	Kac	A	R	A	K	A	K	S	R	S	S	R
		A26	K5me2/K9me	S	G	R	G	Kme2	Q	G	G	Kme	A	R	A	K	A	K	S	R	S	S	R
		A27	K5me2/K9me2	S	G	R	G	Kme2	Q	G	G	Kme2	A	R	A	K	A	K	S	R	S	S	R
		A28	K5me2/K9me3	S	G	R	G	Kme2	Q	G	G	Kme3	A	R	A	K	A	K	S	R	S	S	R
		A29	K5me2/K9ac	S	G	R	G	Kme2	Q	G	G	Kac	A	R	A	K	A	K	S	R	S	S	R
		A30	K5me3/K9me	S	G	R	G	Kme3	Q	G	G	Kme	A	R	A	K	A	K	S	R	S	S	R
		A31	K5me3/K9me2	S	G	R	G	Kme3	Q	G	G	Kme2	A	R	A	K	A	K	S	R	S	S	R
		A32	K5me3/K9me3	S	G	R	G	Kme3	Q	G	G	Kme3	A	R	A	K	A	K	S	R	S	S	R
		A33	K5me3/K9ac	S	G	R	G	Kme3	Q	G	G	Kac	A	R	A	K	A	K	S	R	S	S	R
		A34	K5ac/K9me	S	G	R	G	Kac	Q	G	G	Kme	A	R	A	K	A	K	S	R	S	S	R
		A35	K5ac/K9me2	S	G	R	G	Kac	Q	G	G	Kme2	A	R	A	K	A	K	S	R	S	S	R
		A36	K5ac/K9me3	S	G	R	G	Kac	Q	G	G	Kme3	A	R	A	K	A	K	S	R	S	S	R
		A37	K5ac/K9ac	S	G	R	G	Kac	Q	G	G	Kac	A	R	A	K	A	K	S	R	S	S	R
		B1	K9me/K13me	S	G	R	G	K	Q	G	G	Kme	A	R	A	Kme	A	K	S	R	S	S	R
		B2	K9me/K13me2	S	G	R	G	K	Q	G	G	Kme	A	R	A	Kme2	A	K	S	R	S	S	R
		B3	K9me/K13me3	S	G	R	G	K	Q	G	G	Kme	A	R	A	Kme3	A	K	S	R	S	S	R
		B4	K9me/K13ac	S	G	R	G	K	Q	G	G	Kme	A	R	A	Kac	A	K	S	R	S	S	R
		B5	K9me2/K13me	S	G	R	G	K	Q	G	G	Kme2	A	R	A	Kme	A	K	S	R	S	S	R
		B6	K9me2/K13me2	S	G	R	G	K	Q	G	G	Kme2	A	R	A	Kme2	A	K	S	R	S	S	R
		B7	K9me2/K13me3	S	G	R	G	K	Q	G	G	Kme2	A	R	A	Kme3	A	K	S	R	S	S	R
		B8	K9me2/K13ac	S	G	R	G	K	Q	G	G	Kme2	A	R	A	Kac	A	K	S	R	S	S	R
		B9	K9me3/K13me	S	G	R	G	K	Q	G	G	Kme3	A	R	A	Kme	A	K	S	R	S	S	R
		B10	K9me3/K13me2	S	G	R	G	K	Q	G	G	Kme3	A	R	A	Kme2	A	K	S	R	S	S	R
		B11	K9me3/K13me3	S	G	R	G	K	Q	G	G	Kme3	A	R	A	Kme3	A	K	S	R	S	S	R
		B12	K9me3/K13ac	S	G	R	G	K	Q	G	G	Kme3	A	R	A	Kac	A	K	S	R	S	S	R
		B13	K9ac/K13me	S	G	R	G	K	Q	G	G	Kac	A	R	A	Kme	A	K	S	R	S	S	R
		B14	K9ac/K13me2	S	G	R	G	K	Q	G	G	Kac	A	R	A	Kme2	A	K	S	R	S	S	R
		B15	K9ac/K13me3	S	G	R	G	K	Q	G	G	Kac	A	R	A	Kme3	A	K	S	R	S	S	R
		B16	K9ac/K13ac	S	G	R	G	K	Q	G	G	Kac	A	R	A	Kac	A	K	S	R	S	S	R
		B17	Blank																				
	6-25	B18	CONTROL	Q	G	G	K	A	R	A	K	A	K	S	R	S	S	R	A	G	L	Q	F
		B19	K13me	Q	G	G	K	A	R	A	Kme	A	K	S	R	S	S	R	A	G	L	Q	F
		B20	K13me2	Q	G	G	K	A	R	A	Kme2	A	K	S	R	S	S	R	A	G	L	Q	F
		B21	K13me3	Q	G	G	K	A	R	A	Kme3	A	K	S	R	S	S	R	A	G	L	Q	F
		B22	K13ac	Q	G	G	K	A	R	A	Kac	A	K	S	R	S	S	R	A	G	L	Q	F
		B23	K15me	Q	G	G	K	A	R	A	K	A	Kme	S	R	S	S	R	A	G	L	Q	F
		B24	K15me2	Q	G	G	K	A	R	A	K	A	Kme2	S	R	S	S	R	A	G	L	Q	F
		B25	K15me3	Q	G	G	K	A	R	A	K	A	Kme3	S	R	S	S	R	A	G	L	Q	F
		B26	K15ac	Q	G	G	K	A	R	A	K	A	Kac	S	R	S	S	R	A	G	L	Q	F
		B27	pS16	Q	G	G	K	A	R	A	K	A	K	pS	R	S	S	R	A	G	L	Q	F
		B28	K13me/K15me	Q	G	G	K	A	R	A	Kme	A	Kme	S	R	S	S	R	A	G	L	Q	F
		B29	K13me/K15me2	Q	G	G	K	A	R	A	Kme	A	Kme2	S	R	S	S	R	A	G	L	Q	F
		B30	K13me/K15me3	Q	G	G	K	A	R	A	Kme	A	Kme3	S	R	S	S	R	A	G	L	Q	F
		B31	K13me/K15ac	Q	G	G	K	A	R	A	Kme	A	Kac	S	R	S	S	R	A	G	L	Q	F
		B32	K13me2/K15me	Q	G	G	K	A	R	A	Kme2	A	Kme	S	R	S	S	R	A	G	L	Q	F
		B33	K13me2/K15me2	Q	G	G	K	A	R	A	Kme2	A	Kme2	S	R	S	S	R	A	G	L	Q	F
		B34	K13me2/K15me3	Q	G	G	K	A	R	A	Kme2	A	Kme3	S	R	S	S	R	A	G	L	Q	F
		B35	K13me2/K15ac	Q	G	G	K	A	R	A	Kme2	A	Kac	S	R	S	S	R	A	G	L	Q	F
		B36	K13me3/K15me	Q	G	G	K	A	R	A	Kme3	A	Kme	S	R	S	S	R	A	G	L	Q	F
		B37	K13me3/K15me2	Q	G	G	K	A	R	A	Kme3	A	Kme2	S	R	S	S	R	A	G	L	Q	F
		C1	K13me3/K15me3	Q	G	G	K	A	R	A	Kme3	A	Kme3	S	R	S	S	R	A	G	L	Q	F
		C2	K13me3/K15ac	Q	G	G	K	A	R	A	Kme3	A	Kac	S	R	S	S	R	A	G	L	Q	F
		C3	K13ac/K15me	Q	G	G	K	A	R	A	Kac	A	Kme	S	R	S	S	R	A	G	L	Q	F
		C4	K13ac/K15me2	Q	G	G	K	A	R	A	Kac	A	Kme2	S	R	S	S	R	A	G	L	Q	F
		C5	K13ac/K15me3	Q	G	G	K	A	R	A	Kac	A	Kme3	S	R	S	S	R	A	G	L	Q	F
		C6	K13ac/K15ac	Q	G	G	K	A	R	A	Kac	A	Kac	S	R	S	S	R	A	G	L	Q	F
		C7	K13me/pS16	Q	G	G	K	A	R	A	Kme	A	K	pS	R	S	S	R	A	G	L	Q	F
		C8	K13me2/pS16	Q	G	G	K	A	R	A	Kme2	A	K	pS	R	S	S	R	A	G	L	Q	F
		C9	K13me3/pS16	Q	G	G	K	A	R	A	Kme3	A	K	pS	R	S	S	R	A	G	L	Q	F
		C10	K13ac/pS16	Q	G	G	K	A	R	A	Kac	A	K	pS	R	S	S	R	A	G	L	Q	F
		C11	K15me/pS16	Q	G	G	K	A	R	A	K	A	Kme	pS	R	S	S	R	A	G	L	Q	F
		C12	K15me2/pS16	Q	G	G	K	A	R	A	K	A	Kme2	pS	R	S	S	R	A	G	L	Q	F
		C13	K15me3/pS16	Q	G	G	K	A	R	A	K	A	Kme3	pS	R	S	S	R	A	G	L	Q	F
		C14	K15ac/pS16	Q	G	G	K	A	R	A	K	A	Kac	pS	R	S	S	R	A	G	L	Q	F
		C15	K13me/K15me/pS16	Q	G	G	K	A	R	A	Kme	A	Kme	pS	R	S	S	R	A	G	L	Q	F
		C16	K13me/K15me2/pS16	Q	G	G	K	A	R	A	Kme	A	Kme2	pS	R	S	S	R	A	G	L	Q	F
		C17	K13me/K15me3/pS16	Q	G	G	K	A	R	A	Kme	A	Kme3	pS	R	S	S	R	A	G	L	Q	F
		C18	K13me/K15ac/pS16	Q	G	G	K	A	R	A	Kme	A	Kac	pS	R	S	S	R	A	G	L	Q	F
		C19	K13me2/K15me/pS16	Q	G	G	K	A	R	A	Kme2	A	Kme	pS	R	S	S	R	A	G	L	Q	F
		C20	K13me2/K15me2/pS16	Q	G	G	K	A	R	A	Kme2	A	Kme2	pS	R	S	S	R	A	G	L	Q	F
		C21	K13me2/K15me3/pS16	Q	G	G	K	A	R	A	Kme2	A	Kme3	pS	R	S	S	R	A	G	L	Q	F
		C22	K13me2/K15ac/pS16	Q	G	G	K	A	R	A	Kme2	A	Kac	pS	R	S	S	R	A	G	L	Q	F
		C23	K13me3/K15me/pS16	Q	G	G	K	A	R	A	Kme3	A	Kme	pS	R	S	S	R	A	G	L	Q	F
		C24	K13me3/K15me2/pS16	Q	G	G	K	A	R	A	Kme3	A	Kme2	pS	R	S	S	R	A	G	L	Q	F
		C25	K13me3/K15me3/pS16	Q	G	G	K	A	R	A	Kme3	A	Kme3	pS	R	S	S	R	A	G	L	Q	F
		C26	K13me3/K15ac/pS16	Q	G	G	K	A	R	A	Kme3	A	Kac	pS	R	S	S	R	A	G	L	Q	F
		C27	K13ac/K15me/pS16	Q	G	G	K	A	R	A	Kac	A	Kme	pS	R	S	S	R	A	G	L	Q	F

	**C28**	K13ac/K15me2/pS16	Q	G	G	K	A	R	A	Kac	A	Kme2	pS	R	S	S	R	A	G	L	Q	F
	**C29**	K13ac/K15me3/pS16	Q	G	G	K	A	R	A	Kac	A	Kme3	pS	R	S	S	R	A	G	L	Q	F
	**C30**	K13ac/K15ac/pS16	Q	G	G	K	A	R	A	Kac	A	Kac	pS	R	S	S	R	A	G	L	Q	F
	**C31**	pS18	Q	G	G	K	A	R	A	K	A	K	S	R	pS	S	R	A	G	L	Q	F
	**C32**	K13me/pS18	Q	G	G	K	A	R	A	Kme	A	K	S	R	pS	S	R	A	G	L	Q	F
	**C33**	K13me2/pS18	Q	G	G	K	A	R	A	Kme2	A	K	S	R	pS	S	R	A	G	L	Q	F
	**C34**	K13me3/pS18	Q	G	G	K	A	R	A	Kme3	A	K	S	R	pS	S	R	A	G	L	Q	F
	**C35**	K13ac/pS18	Q	G	G	K	A	R	A	Kac	A	K	S	R	pS	S	R	A	G	L	Q	F
	**C36**	K15me/pS18	Q	G	G	K	A	R	A	K	A	Kme	S	R	pS	S	R	A	G	L	Q	F
	**C37**	K15me2/pS18	Q	G	G	K	A	R	A	K	A	Kme2	S	R	pS	S	R	A	G	L	Q	F
	**D1**	K15me3/pS18	Q	G	G	K	A	R	A	K	A	Kme3	S	R	pS	S	R	A	G	L	Q	F
	**D2**	K15ac/pS18	Q	G	G	K	A	R	A	K	A	Kac	S	R	pS	S	R	A	G	L	Q	F
	**D3**	K13me/K15me/pS18	Q	G	G	K	A	R	A	Kme	A	Kme	S	R	pS	S	R	A	G	L	Q	F
	**D4**	K13me/K15me2/pS18	Q	G	G	K	A	R	A	Kme	A	Kme2	S	R	pS	S	R	A	G	L	Q	F
	**D5**	K13me/K15me3/pS18	Q	G	G	K	A	R	A	Kme	A	Kme3	S	R	pS	S	R	A	G	L	Q	F
	**D6**	K13me/K15ac/pS18	Q	G	G	K	A	R	A	Kme	A	Kac	S	R	pS	S	R	A	G	L	Q	F
	**D7**	K13me2/K15me/pS18	Q	G	G	K	A	R	A	Kme2	A	Kme	S	R	pS	S	R	A	G	L	Q	F
	**D8**	K13me2/K15me2/pS18	Q	G	G	K	A	R	A	Kme2	A	Kme2	S	R	pS	S	R	A	G	L	Q	F
	**D9**	K13me2/K15me3/pS18	Q	G	G	K	A	R	A	Kme2	A	Kme3	S	R	pS	S	R	A	G	L	Q	F
	**D10**	K13me2/K15ac/pS18	Q	G	G	K	A	R	A	Kme2	A	Kac	S	R	pS	S	R	A	G	L	Q	F
	**D11**	K13me3/K15me/pS18	Q	G	G	K	A	R	A	Kme3	A	Kme	S	R	pS	S	R	A	G	L	Q	F
	**D12**	K13me3/K15me2/pS18	Q	G	G	K	A	R	A	Kme3	A	Kme2	S	R	pS	S	R	A	G	L	Q	F
	**D13**	K13me3/K15me3/pS18	Q	G	G	K	A	R	A	Kme3	A	Kme3	S	R	pS	S	R	A	G	L	Q	F
	**D14**	K13me3/K15ac/pS18	Q	G	G	K	A	R	A	Kme3	A	Kac	S	R	pS	S	R	A	G	L	Q	F
	**D15**	K13ac/K15me/pS18	Q	G	G	K	A	R	A	Kac	A	Kme	S	R	pS	S	R	A	G	L	Q	F
	**D16**	K13ac/K15me2/pS18	Q	G	G	K	A	R	A	Kac	A	Kme2	S	R	pS	S	R	A	G	L	Q	F
	**D17**	K13ac/K15me3/pS18	Q	G	G	K	A	R	A	Kac	A	Kme3	S	R	pS	S	R	A	G	L	Q	F
	**D18**	K13ac/K15ac/pS18	Q	G	G	K	A	R	A	Kac	A	Kac	S	R	pS	S	R	A	G	L	Q	F
	**D19**	pS19	Q	G	G	K	A	R	A	K	A	K	S	R	S	pS	R	A	G	L	Q	F
	**D20**	K13me/K15me/pS19	Q	G	G	K	A	R	A	Kme	A	Kme	S	R	S	pS	R	A	G	L	Q	F
	**D21**	K13me/K15me2/pS19	Q	G	G	K	A	R	A	Kme	A	Kme2	S	R	S	pS	R	A	G	L	Q	F
	**D22**	K13me/K15me3/pS19	Q	G	G	K	A	R	A	Kme	A	Kme3	S	R	S	pS	R	A	G	L	Q	F
	**D23**	K13me/K15ac/pS19	Q	G	G	K	A	R	A	Kme	A	Kac	S	R	S	pS	R	A	G	L	Q	F
	**D24**	K13me2/K15me/pS19	Q	G	G	K	A	R	A	Kme2	A	Kme	S	R	S	pS	R	A	G	L	Q	F
	**D25**	K13me2/K15me2/pS19	Q	G	G	K	A	R	A	Kme2	A	Kme2	S	R	S	pS	R	A	G	L	Q	F
	**D26**	K13me2/K15me3/pS19	Q	G	G	K	A	R	A	Kme2	A	Kme3	S	R	S	pS	R	A	G	L	Q	F
	**D27**	K13me2/K15ac/pS19	Q	G	G	K	A	R	A	Kme2	A	Kac	S	R	S	pS	R	A	G	L	Q	F
	**D28**	K13me3/K15me/pS19	Q	G	G	K	A	R	A	Kme3	A	Kme	S	R	S	pS	R	A	G	L	Q	F
	**D29**	K13me3/K15me2/pS19	Q	G	G	K	A	R	A	Kme3	A	Kme2	S	R	S	pS	R	A	G	L	Q	F
	**D30**	K13me3/K15me3/pS19	Q	G	G	K	A	R	A	Kme3	A	Kme3	S	R	S	pS	R	A	G	L	Q	F
	**D31**	K13me3/K15ac/pS19	Q	G	G	K	A	R	A	Kme3	A	Kac	S	R	S	pS	R	A	G	L	Q	F
	**D32**	K13ac/K15me/pS19	Q	G	G	K	A	R	A	Kac	A	Kme	S	R	S	pS	R	A	G	L	Q	F
	**D33**	K13ac/K15me2/pS19	Q	G	G	K	A	R	A	Kac	A	Kme2	S	R	S	pS	R	A	G	L	Q	F
	**D34**	K13ac/K15me3/pS19	Q	G	G	K	A	R	A	Kac	A	Kme3	S	R	S	pS	R	A	G	L	Q	F
	**D35**	K13ac/K15ac/pS19	Q	G	G	K	A	R	A	Kac	A	Kac	S	R	S	pS	R	A	G	L	Q	F
	**D36**	Blank																				
11-30	**D37**	CONTROL	R	A	K	A	K	S	R	S	**S**	R	A	G	L	Q	F	P	V	G	R	V
	**E1**	K15me	R	A	K	A	Kme	S	R	S	S	R	A	G	L	Q	F	P	V	G	R	V
	**E2**	K15me2	R	A	K	A	Kme2	S	R	S	S	R	A	G	L	Q	F	P	V	G	R	V
	**E3**	K15me3	R	A	K	A	Kme3	S	R	S	S	R	A	G	L	Q	F	P	V	G	R	V
	**E4**	K15ac	R	A	K	A	Kac	S	R	S	S	R	A	G	L	Q	F	P	V	G	R	V
	**E5**	pS16	R	A	K	A	K	pS	R	S	S	R	A	G	L	Q	F	P	V	G	R	V
	**E6**	pS18	R	A	K	A	K	S	R	pS	S	R	A	G	L	Q	F	P	V	G	R	V
	**E7**	pS19	R	A	K	A	K	S	R	S	pS	R	A	G	L	Q	F	P	V	G	R	V
	**E8**	K15me/pS16	R	A	K	A	Kme	pS	R	S	S	R	A	G	L	Q	F	P	V	G	R	V
	**E9**	K15me2/pS16	R	A	K	A	Kme2	pS	R	S	S	R	A	G	L	Q	F	P	V	G	R	V
	**E10**	K15me3/pS16	R	A	K	A	Kme3	pS	R	S	S	R	A	G	L	Q	F	P	V	G	R	V
	**E11**	K15ac/pS16	R	A	K	A	Kac	pS	R	S	S	R	A	G	L	Q	F	P	V	G	R	V
	**E12**	K15me/pS18	R	A	K	A	Kme	S	R	pS	S	R	A	G	L	Q	F	P	V	G	R	V
	**E13**	K15me2/pS18	R	A	K	A	Kme2	S	R	pS	S	R	A	G	L	Q	F	P	V	G	R	V
	**E14**	K15me3/pS18	R	A	K	A	Kme3	S	R	pS	S	R	A	G	L	Q	F	P	V	G	R	V
	**E15**	K15ac/pS18	R	A	K	A	Kac	S	R	pS	S	R	A	G	L	Q	F	P	V	G	R	V
	**E16**	K15me/pS19	R	A	K	A	Kme	S	R	S	pS	R	A	G	L	Q	F	P	V	G	R	V
	**E17**	K15me2/pS19	R	A	K	A	Kme2	S	R	S	pS	R	A	G	L	Q	F	P	V	G	R	V
	**E18**	K15me3/pS19	R	A	K	A	Kme3	S	R	S	pS	R	A	G	L	Q	F	P	V	G	R	V
	**E19**	K15ac/pS19	R	A	K	A	Kac	S	R	S	pS	R	A	G	L	Q	F	P	V	G	R	V
	**E20**	Blank																				
27-46	**E21**	CONTROL	V	G	R	V	H	R	L	L	R	K	G	N	Y	A	E	R	V	G	A	G
	**E22**	K36me	V	G	R	V	H	R	L	L	R	Kme	G	N	Y	A	E	R	V	G	A	G
	**E23**	K36me2	V	G	R	V	H	R	L	L	R	Kme2	G	N	Y	A	E	R	V	G	A	G
	**E24**	K36me3	V	G	R	V	H	R	L	L	R	Kme3	G	N	Y	A	E	R	V	G	A	G
	**E25**	K36ac	V	G	R	V	H	R	L	L	R	Kac	G	N	Y	A	E	R	V	G	A	G

Histone	Peptide Length	Array Poition	Kac Mark Combinations	Sequence																			
polyHis CTRL		A1	HHHHHHHH																				
polyHis CTRL		A2	HHHHHHHH																				
		A3	Blank																				
H2A.X (P16104)	1-20	A4	CONTROL	S	G	R	G	K	T	G	G	K	A	R	A	K	A	K	S	R	S	S	R
		A5	K5me	S	G	R	G	Kme	T	G	G	K	A	R	A	K	A	K	S	R	S	S	R
		A6	K5me2	S	G	R	G	Kme2	T	G	G	K	A	R	A	K	A	K	S	R	S	S	R
		A7	K5me3	S	G	R	G	Kme3	T	G	G	K	A	R	A	K	A	K	S	R	S	S	R
		A8	K5ac	S	G	R	G	Kac	T	G	G	K	A	R	A	K	A	K	S	R	S	S	R
		A9	pT6	S	G	R	G	K	pT	G	G	K	A	R	A	K	A	K	S	R	S	S	R
		A10	K9me	S	G	R	G	K	T	G	G	Kme	A	R	A	K	A	K	S	R	S	S	R
		A11	K9me2	S	G	R	G	K	T	G	G	Kme2	A	R	A	K	A	K	S	R	S	S	R
		A12	K9me3	S	G	R	G	K	T	G	G	Kme3	A	R	A	K	A	K	S	R	S	S	R
		A13	K9ac	S	G	R	G	K	T	G	G	Kac	A	R	A	K	A	K	S	R	S	S	R
		A14	K5me/pT6	S	G	R	G	Kme	pT	G	G	K	A	R	A	K	A	K	S	R	S	S	R
		A15	K5me2/pT6	S	G	R	G	Kme2	pT	G	G	K	A	R	A	K	A	K	S	R	S	S	R
		A16	K5me3/pT6	S	G	R	G	Kme3	pT	G	G	K	A	R	A	K	A	K	S	R	S	S	R
		A17	K5ac/pT6	S	G	R	G	Kac	pT	G	G	K	A	R	A	K	A	K	S	R	S	S	R
		A18	pT6/K9me	S	G	R	G	K	pT	G	G	Kme	A	R	A	K	A	K	S	R	S	S	R
		A19	pT6/K9me2	S	G	R	G	K	pT	G	G	Kme2	A	R	A	K	A	K	S	R	S	S	R
		A20	pT6/K9me3	S	G	R	G	K	pT	G	G	Kme3	A	R	A	K	A	K	S	R	S	S	R
		A21	pT6/K9ac	S	G	R	G	K	pT	G	G	Kac	A	R	A	K	A	K	S	R	S	S	R
		A22	K5me/pT6/K9me	S	G	R	G	Kme	pT	G	G	Kme	A	R	A	K	A	K	S	R	S	S	R
		A23	K5me/pT6/K9me2	S	G	R	G	Kme	pT	G	G	Kme2	A	R	A	K	A	K	S	R	S	S	R
		A24	K5me/pT6/K9me3	S	G	R	G	Kme	pT	G	G	Kme3	A	R	A	K	A	K	S	R	S	S	R
		A25	K5me/pT6/K9ac	S	G	R	G	Kme	pT	G	G	Kac	A	R	A	K	A	K	S	R	S	S	R
		A26	K5me2/pT6/K9me	S	G	R	G	Kme2	pT	G	G	Kme	A	R	A	K	A	K	S	R	S	S	R
		A27	K5me2/pT6/K9me2	S	G	R	G	Kme2	pT	G	G	Kme2	A	R	A	K	A	K	S	R	S	S	R
		A28	K5me2/pT6/K9me3	S	G	R	G	Kme2	pT	G	G	Kme3	A	R	A	K	A	K	S	R	S	S	R
		A29	K5me2/pT6/K9ac	S	G	R	G	Kme2	pT	G	G	Kac	A	R	A	K	A	K	S	R	S	S	R
		A30	K5me3/pT6/K9me	S	G	R	G	Kme3	pT	G	G	Kme	A	R	A	K	A	K	S	R	S	S	R
		A31	K5me3/pT6/K9me2	S	G	R	G	Kme3	pT	G	G	Kme2	A	R	A	K	A	K	S	R	S	S	R
		A32	K5me3/pT6/K9me3	S	G	R	G	Kme3	pT	G	G	Kme3	A	R	A	K	A	K	S	R	S	S	R
		A33	K5me3/pT6/K9ac	S	G	R	G	Kme3	pT	G	G	Kac	A	R	A	K	A	K	S	R	S	S	R
		A34	K5ac/pT6/K9me	S	G	R	G	Kac	pT	G	G	Kme	A	R	A	K	A	K	S	R	S	S	R
		A35	K5ac/pT6/K9me2	S	G	R	G	Kac	pT	G	G	Kme2	A	R	A	K	A	K	S	R	S	S	R
		A36	K5ac/pT6/K9me3	S	G	R	G	Kac	pT	G	G	Kme3	A	R	A	K	A	K	S	R	S	S	R
		A37	K5ac/pT6/K9ac	S	G	R	G	Kac	pT	G	G	Kac	A	R	A	K	A	K	S	R	S	S	R
		B1	Blank																				
		B2	CONTROL	T	V	G	P	K	A	P	S	G	G	K	K	A	T	Q	A	S	Q	E	Y
		B3	K127me	T	V	G	P	Kme	A	P	S	G	G	K	K	A	T	Q	A	S	Q	E	Y
		B4	K127me2	T	V	G	P	Kme2	A	P	S	G	G	K	K	A	T	Q	A	S	Q	E	Y
		B5	K127me3	T	V	G	P	Kme3	A	P	S	G	G	K	K	A	T	Q	A	S	Q	E	Y
		B6	K127ac	T	V	G	P	Kac	A	P	S	G	G	K	K	A	T	Q	A	S	Q	E	Y
		B7	pS130	T	V	G	P	K	A	P	pS	G	G	K	K	A	T	Q	A	S	Q	E	Y
		B8	K133me	T	V	G	P	K	A	P	S	G	G	Kme	K	A	T	Q	A	S	Q	E	Y
		B9	K133me2	T	V	G	P	K	A	P	S	G	G	Kme2	K	A	T	Q	A	S	Q	E	Y
		B10	K133me3	T	V	G	P	K	A	P	S	G	G	Kme3	K	A	T	Q	A	S	Q	E	Y
		B11	K133ac	T	V	G	P	K	A	P	S	G	G	Kac	K	A	T	Q	A	S	Q	E	Y
		B12	K134me	T	V	G	P	K	A	P	S	G	G	K	Kme	A	T	Q	A	S	Q	E	Y
		B13	K134me2	T	V	G	P	K	A	P	S	G	G	K	Kme2	A	T	Q	A	S	Q	E	Y
		B14	K134me3	T	V	G	P	K	A	P	S	G	G	K	Kme3	A	T	Q	A	S	Q	E	Y
		B15	K134ac	T	V	G	P	K	A	P	S	G	G	K	Kac	A	T	Q	A	S	Q	E	Y
		B16	pS136	T	V	G	P	K	A	P	S	G	G	K	K	A	pT	Q	A	S	Q	E	Y
		B17	pS139	T	V	G	P	K	A	P	S	G	G	K	K	A	T	Q	A	pS	Q	E	Y
	6-25	B18	K127me/pS130	T	V	G	P	Kme	A	P	pS	G	G	K	K	A	T	Q	A	S	Q	E	Y
		B19	K127me2/pS130	T	V	G	P	Kme2	A	P	pS	G	G	K	K	A	T	Q	A	S	Q	E	Y
		B20	K127me3/pS130	T	V	G	P	Kme3	A	P	pS	G	G	K	K	A	T	Q	A	S	Q	E	Y
		B21	K127ac/pS130	T	V	G	P	Kac	A	P	pS	G	G	K	K	A	T	Q	A	S	Q	E	Y
		B22	pS130/K133me	T	V	G	P	K	A	P	pS	G	G	Kme	K	A	T	Q	A	S	Q	E	Y
		B23	pS130/K133me2	T	V	G	P	K	A	P	pS	G	G	Kme2	K	A	T	Q	A	S	Q	E	Y
		B24	pS130/K133me3	T	V	G	P	K	A	P	pS	G	G	Kme3	K	A	T	Q	A	S	Q	E	Y
		B25	pS130/K133ac	T	V	G	P	K	A	P	pS	G	G	Kac	K	A	T	Q	A	S	Q	E	Y
		B26	pS130/K134me	T	V	G	P	K	A	P	pS	G	G	K	Kme	A	T	Q	A	S	Q	E	Y
		B27	pS130/K134me2	T	V	G	P	K	A	P	pS	G	G	K	Kme2	A	T	Q	A	S	Q	E	Y
		B28	pS130/K134me3	T	V	G	P	K	A	P	pS	G	G	K	Kme3	A	T	Q	A	S	Q	E	Y
		B29	pS130/K134ac	T	V	G	P	K	A	P	pS	G	G	K	Kac	A	T	Q	A	S	Q	E	Y
		B30	K133me/pT136	T	V	G	P	K	A	P	S	G	G	Kme	K	A	pT	Q	A	S	Q	E	Y
		B31	K133me2/pT136	T	V	G	P	K	A	P	S	G	G	Kme2	K	A	pT	Q	A	S	Q	E	Y
		B32	K133me3/pT136	T	V	G	P	K	A	P	S	G	G	Kme3	K	A	pT	Q	A	S	Q	E	Y
		B33	K133ac/pT136	T	V	G	P	K	A	P	S	G	G	Kac	K	A	pT	Q	A	S	Q	E	Y
		B34	K134me/pT136	T	V	G	P	K	A	P	S	G	G	K	Kme	A	pT	Q	A	S	Q	E	Y
		B35	K134me2/pT136	T	V	G	P	K	A	P	S	G	G	K	Kme2	A	pT	Q	A	S	Q	E	Y
		B36	K134me3/pT136	T	V	G	P	K	A	P	S	G	G	K	Kme3	A	pT	Q	A	S	Q	E	Y
		B37	K134ac/pT136	T	V	G	P	K	A	P	S	G	G	K	Kac	A	pT	Q	A	S	Q	E	Y
		C1	K133me/pS139	T	V	G	P	K	A	P	S	G	G	Kme	K	A	T	Q	A	pS	Q	E	Y
		C2	K133me2/pS139	T	V	G	P	K	A	P	S	G	G	Kme2	K	A	T	Q	A	pS	Q	E	Y
		C3	K133me3/pS139	T	V	G	P	K	A	P	S	G	G	Kme3	K	A	T	Q	A	pS	Q	E	Y
		C4	K133ac/pS139	T	V	G	P	K	A	P	S	G	G	Kac	K	A	T	Q	A	pS	Q	E	Y
		C5	K134me/pS139	T	V	G	P	K	A	P	S	G	G	K	Kme	A	T	Q	A	pS	Q	E	Y
		C6	K134me2/pS139	T	V	G	P	K	A	P	S	G	G	K	Kme2	A	T	Q	A	pS	Q	E	Y
		C7	K134me3/pS139	T	V	G	P	K	A	P	S	G	G	K	Kme3	A	T	Q	A	pS	Q	E	Y
		C8	K134ac/pS139	T	V	G	P	K	A	P	S	G	G	K	Kac	A	T	Q	A	pS	Q	E	Y
		C9	K127me/K133me	T	V	G	P	Kme	A	P	S	G	G	Kme	K	A	T	Q	A	S	Q	E	Y
		C10	K127me/K133me2	T	V	G	P	Kme	A	P	S	G	G	Kme2	K	A	T	Q	A	S	Q	E	Y
		C11	K127me/K133me3	T	V	G	P	Kme	A	P	S	G	G	Kme3	K	A	T	Q	A	S	Q	E	Y
		C12	K127me/K133ac	T	V	G	P	Kme	A	P	S	G	G	Kac	K	A	T	Q	A	S	Q	E	Y
		C13	K127me2/K133me	T	V	G	P	Kme2	A	P	S	G	G	Kme	K	A	T	Q	A	S	Q	E	Y
		C14	K127me2/K133me2	T	V	G	P	Kme2	A	P	S	G	G	Kme2	K	A	T	Q	A	S	Q	E	Y
		C15	K127me2/K133me3	T	V	G	P	Kme2	A	P	S	G	G	Kme3	K	A	T	Q	A	S	Q	E	Y
		C16	K127me2/K133ac	T	V	G	P	Kme2	A	P	S	G	G	Kac	K	A	T	Q	A	S	Q	E	Y
		C17	K127me3/K133me	T	V	G	P	Kme3	A	P	S	G	G	Kme	K	A	T	Q	A	S	Q	E	Y
		C18	K127me3/K133me2	T	V	G	P	Kme3	A	P	S	G	G	Kme2	K	A	T	Q	A	S	Q	E	Y
		C19	K127me3/K133me3	T	V	G	P	Kme3	A	P	S	G	G	Kme3	K	A	T	Q	A	S	Q	E	Y
		C20	K127me3/K133ac	T	V	G	P	Kme3	A	P	S	G	G	Kac	K	A	T	Q	A	S	Q	E	Y
		C21	K127ac/K133me	T	V	G	P	Kac	A	P	S	G	G	Kme	K	A	T	Q	A	S	Q	E	Y
		C22	K127ac/K133me2	T	V	G	P	Kac	A	P	S	G	G	Kme2	K	A	T	Q	A	S	Q	E	Y
		C23	K127ac/K133me3	T	V	G	P	Kac	A	P	S	G	G	Kme3	K	A	T	Q	A	S	Q	E	Y
		C24	K127ac/K133ac	T	V	G	P	Kac	A	P	S	G	G	Kac	K	A	T	Q	A	S	Q	E	Y
		C25	K127me/K134me	T	V	G	P	Kme	A	P	S	G	G	K	Kme	A	T	Q	A	S	Q	E	Y
		C26	K127me/K134me2	T	V	G	P	Kme	A	P	S	G	G	K	Kme2	A	T	Q	A	S	Q	E	Y
		C27	K127me/K134me3	T	V	G	P	Kme	A	P	S	G	G	K	Kme3	A	T	Q	A	S	Q	E	Y

**C28**	K127me/K134ac	T	V	G	P	Kme	A	P	S	G	G	K	Kac	A	T	Q	A	S	Q	E	Y
**C29**	K127me2/K134me	T	V	G	P	Kme2	A	P	S	G	G	K	Kme	A	T	Q	A	S	Q	E	Y
**C30**	K127me2/K134me2	T	V	G	P	Kme2	A	P	S	G	G	K	Kme2	A	T	Q	A	S	Q	E	Y
**C31**	K127me2/K134me3	T	V	G	P	Kme2	A	P	S	G	G	K	Kme3	A	T	Q	A	S	Q	E	Y
**C32**	K127me2/K134ac	T	V	G	P	Kme2	A	P	S	G	G	K	Kac	A	T	Q	A	S	Q	E	Y
**C33**	K127me3/K134me	T	V	G	P	Kme3	A	P	S	G	G	K	Kme	A	T	Q	A	S	Q	E	Y
**C34**	K127me3/K134me2	T	V	G	P	Kme3	A	P	S	G	G	K	Kme2	A	T	Q	A	S	Q	E	Y
**C35**	K127me3/K134me3	T	V	G	P	Kme3	A	P	S	G	G	K	Kme3	A	T	Q	A	S	Q	E	Y
**C36**	K127me3/K134ac	T	V	G	P	Kme3	A	P	S	G	G	K	Kac	A	T	Q	A	S	Q	E	Y
**C37**	K127ac/K134me	T	V	G	P	Kac	A	P	S	G	G	K	Kme	A	T	Q	A	S	Q	E	Y
**D1**	K127ac/K134me2	T	V	G	P	Kac	A	P	S	G	G	K	Kme2	A	T	Q	A	S	Q	E	Y
**D2**	K127ac/K134me3	T	V	G	P	Kac	A	P	S	G	G	K	Kme3	A	T	Q	A	S	Q	E	Y
**D3**	K127ac/K134ac	T	V	G	P	Kac	A	P	S	G	G	K	Kac	A	T	Q	A	S	Q	E	Y
**D4**	K127ac/K133ac/K134ac	T	V	G	P	Kac	A	P	S	G	G	Kac	Kac	A	T	Q	A	S	Q	E	Y
**D5**	K127ac/K133ac/K134ac/pS139	T	V	G	P	Kac	A	P	S	G	G	Kac	Kac	A	T	Q	A	pS	Q	E	Y

Histone	Peptide Length	Array Poition	Kac Mark Combinations	Sequence																			
polyHis CTRL		A1	HHHHHHHH																				
polyHis CTRL		A2	HHHHHHHH																				
		A3	Blank																				
H2A.Z (P0C0S5)	1-20	A4	CONTROL	A	G	G	K	A	G	K	D	S	G	K	A	K	T	K	A	V	S	R	S
		A5	K4me	A	G	G	Kme	A	G	K	D	S	G	K	A	K	T	K	A	V	S	R	S
		A6	K4me2	A	G	G	Kme2	A	G	K	D	S	G	K	A	K	T	K	A	V	S	R	S
		A7	K4me3	A	G	G	Kme3	A	G	K	D	S	G	K	A	K	T	K	A	V	S	R	S
		A8	K4ac	A	G	G	Kac	A	G	K	D	S	G	K	A	K	T	K	A	V	S	R	S
		A9	K7me	A	G	G	K	A	G	Kme	D	S	G	K	A	K	T	K	A	V	S	R	S
		A10	K7me2	A	G	G	K	A	G	Kme2	D	S	G	K	A	K	T	K	A	V	S	R	S
		A11	K7me3	A	G	G	K	A	G	Kme3	D	S	G	K	A	K	T	K	A	V	S	R	S
		A12	K7ac	A	G	G	K	A	G	Kac	D	S	G	K	A	K	T	K	A	V	S	R	S
		A13	pS9	A	G	G	K	A	G	K	D	pS	G	K	A	K	T	K	A	V	S	R	S
		A14	K11me	A	G	G	K	A	G	K	D	S	G	Kme	A	K	T	K	A	V	S	R	S
		A15	K11me2	A	G	G	K	A	G	K	D	S	G	Kme2	A	K	T	K	A	V	S	R	S
		A16	K11me3	A	G	G	K	A	G	K	D	S	G	Kme3	A	K	T	K	A	V	S	R	S
		A17	K11ac	A	G	G	K	A	G	K	D	S	G	Kac	A	K	T	K	A	V	S	R	S
		A18	K13me	A	G	G	K	A	G	K	D	S	G	K	A	Kme	T	K	A	V	S	R	S
		A19	K13me2	A	G	G	K	A	G	K	D	S	G	K	A	Kme2	T	K	A	V	S	R	S
		A20	K13me3	A	G	G	K	A	G	K	D	S	G	K	A	Kme3	T	K	A	V	S	R	S
		A21	K13ac	A	G	G	K	A	G	K	D	S	G	K	A	Kac	T	K	A	V	S	R	S
		A22	K4me/K7me	A	G	G	Kme	A	G	Kme	D	S	G	K	A	K	T	K	A	V	S	R	S
		A23	K4me/K7me2	A	G	G	Kme	A	G	Kme2	D	S	G	K	A	K	T	K	A	V	S	R	S
		A24	K4me/K7me3	A	G	G	Kme	A	G	Kme3	D	S	G	K	A	K	T	K	A	V	S	R	S
		A25	K4me/K7ac	A	G	G	Kme	A	G	Kac	D	S	G	K	A	K	T	K	A	V	S	R	S
		A26	K4me2/K7me	A	G	G	Kme2	A	G	Kme	D	S	G	K	A	K	T	K	A	V	S	R	S
		A27	K4me2/K7me2	A	G	G	Kme2	A	G	Kme2	D	S	G	K	A	K	T	K	A	V	S	R	S
		A28	K4me2/K7me3	A	G	G	Kme2	A	G	Kme3	D	S	G	K	A	K	T	K	A	V	S	R	S
		A29	K4me2/K7ac	A	G	G	Kme2	A	G	Kac	D	S	G	K	A	K	T	K	A	V	S	R	S
		A30	K4me3/K7me	A	G	G	Kme3	A	G	Kme	D	S	G	K	A	K	T	K	A	V	S	R	S
		A31	K4me3/K7me2	A	G	G	Kme3	A	G	Kme2	D	S	G	K	A	K	T	K	A	V	S	R	S
		A32	K4me3/K7me3	A	G	G	Kme3	A	G	Kme3	D	S	G	K	A	K	T	K	A	V	S	R	S
		A33	K4me3/K7ac	A	G	G	Kme3	A	G	Kac	D	S	G	K	A	K	T	K	A	V	S	R	S
		A34	K4ac/K7me	A	G	G	Kac	A	G	Kme	D	S	G	K	A	K	T	K	A	V	S	R	S
		A35	K4ac/K7me2	A	G	G	Kac	A	G	Kme2	D	S	G	K	A	K	T	K	A	V	S	R	S
		A36	K4ac/K7me3	A	G	G	Kac	A	G	Kme3	D	S	G	K	A	K	T	K	A	V	S	R	S
		A37	K4ac/K7ac	A	G	G	Kac	A	G	Kac	D	S	G	K	A	K	T	K	A	V	S	R	S
		B1	K4me/pS9	A	G	G	Kme	A	G	K	D	pS	G	K	A	K	T	K	A	V	S	R	S
		B2	K4me2/pS9	A	G	G	Kme2	A	G	K	D	pS	G	K	A	K	T	K	A	V	S	R	S
		B3	K4me3/pS9	A	G	G	Kme3	A	G	K	D	pS	G	K	A	K	T	K	A	V	S	R	S
		B4	K4ac/pS9	A	G	G	Kac	A	G	K	D	pS	G	K	A	K	T	K	A	V	S	R	S
		B5	K7me/pS9	A	G	G	K	A	G	Kme	D	pS	G	K	A	K	T	K	A	V	S	R	S
		B6	K7me2/pS9	A	G	G	K	A	G	Kme2	D	pS	G	K	A	K	T	K	A	V	S	R	S
		B7	K7me3/pS9	A	G	G	K	A	G	Kme3	D	pS	G	K	A	K	T	K	A	V	S	R	S
		B8	K7ac/pS9	A	G	G	K	A	G	Kac	D	pS	G	K	A	K	T	K	A	V	S	R	S
		B9	pS9/K11me	A	G	G	K	A	G	K	D	pS	G	Kme	A	K	T	K	A	V	S	R	S
		B10	pS9/K11me2	A	G	G	K	A	G	K	D	pS	G	Kme2	A	K	T	K	A	V	S	R	S
		B11	pS9/K11me3	A	G	G	K	A	G	K	D	pS	G	Kme3	A	K	T	K	A	V	S	R	S
		B12	pS9/K11ac	A	G	G	K	A	G	K	D	pS	G	Kac	A	K	T	K	A	V	S	R	S
		B13	K7me/K11me	A	G	G	K	A	G	Kme	D	S	G	Kme	A	K	T	K	A	V	S	R	S
		B14	K7me/K11me2	A	G	G	K	A	G	Kme	D	S	G	Kme2	A	K	T	K	A	V	S	R	S
		B15	K7me/K11me3	A	G	G	K	A	G	Kme	D	S	G	Kme3	A	K	T	K	A	V	S	R	S
		B16	K7me/K11ac	A	G	G	K	A	G	Kme	D	S	G	Kac	A	K	T	K	A	V	S	R	S
		B17	K7me2/K11me	A	G	G	K	A	G	Kme2	D	S	G	Kme	A	K	T	K	A	V	S	R	S
	6-25	B18	K7me2/K11me2	A	G	G	K	A	G	Kme2	D	S	G	Kme2	A	K	T	K	A	V	S	R	S
		B19	K7me2/K11me3	A	G	G	K	A	G	Kme2	D	S	G	Kme3	A	K	T	K	A	V	S	R	S
		B20	K7me2/K11ac	A	G	G	K	A	G	Kme2	D	S	G	Kac	A	K	T	K	A	V	S	R	S
		B21	K7me3/K11me	A	G	G	K	A	G	Kme3	D	S	G	Kme	A	K	T	K	A	V	S	R	S
		B22	K7me3/K11me2	A	G	G	K	A	G	Kme3	D	S	G	Kme2	A	K	T	K	A	V	S	R	S
		B23	K7me3/K11me3	A	G	G	K	A	G	Kme3	D	S	G	Kme3	A	K	T	K	A	V	S	R	S
		B24	K7me3/K11ac	A	G	G	K	A	G	Kme3	D	S	G	Kac	A	K	T	K	A	V	S	R	S
		B25	K7ac/K11me	A	G	G	K	A	G	Kac	D	S	G	Kme	A	K	T	K	A	V	S	R	S
		B26	K7ac/K11me2	A	G	G	K	A	G	Kac	D	S	G	Kme2	A	K	T	K	A	V	S	R	S
		B27	K7ac/K11me3	A	G	G	K	A	G	Kac	D	S	G	Kme3	A	K	T	K	A	V	S	R	S
		B28	K7ac/K11ac	A	G	G	K	A	G	Kac	D	S	G	Kac	A	K	T	K	A	V	S	R	S
		B29	K7me/pS9/K11me	A	G	G	K	A	G	Kme	D	pS	G	Kme	A	K	T	K	A	V	S	R	S
		B30	K7me/pS9/K11me2	A	G	G	K	A	G	Kme	D	pS	G	Kme2	A	K	T	K	A	V	S	R	S
		B31	K7me/pS9/K11me3	A	G	G	K	A	G	Kme	D	pS	G	Kme3	A	K	T	K	A	V	S	R	S
		B32	K7me/pS9/K11ac	A	G	G	K	A	G	Kme	D	pS	G	Kac	A	K	T	K	A	V	S	R	S
		B33	K7me2/pS9/K11me	A	G	G	K	A	G	Kme2	D	pS	G	Kme	A	K	T	K	A	V	S	R	S
		B34	K7me2/pS9/K11me2	A	G	G	K	A	G	Kme2	D	pS	G	Kme2	A	K	T	K	A	V	S	R	S
		B35	K7me2/pS9/K11me3	A	G	G	K	A	G	Kme2	D	pS	G	Kme3	A	K	T	K	A	V	S	R	S
		B36	K7me2/pS9/K11ac	A	G	G	K	A	G	Kme2	D	pS	G	Kac	A	K	T	K	A	V	S	R	S
		B37	K7me3/pS9/K11me	A	G	G	K	A	G	Kme3	D	pS	G	Kme	A	K	T	K	A	V	S	R	S
		C1	K7me3/pS9/K11me2	A	G	G	K	A	G	Kme3	D	pS	G	Kme2	A	K	T	K	A	V	S	R	S
		C2	K7me3/pS9/K11me3	A	G	G	K	A	G	Kme3	D	pS	G	Kme3	A	K	T	K	A	V	S	R	S
		C3	K7me3/pS9/K11ac	A	G	G	K	A	G	Kme3	D	pS	G	Kac	A	K	T	K	A	V	S	R	S
		C4	K7ac/pS9/K11me	A	G	G	K	A	G	Kac	D	pS	G	Kme	A	K	T	K	A	V	S	R	S
		C5	K7ac/pS9/K11me2	A	G	G	K	A	G	Kac	D	pS	G	Kme2	A	K	T	K	A	V	S	R	S
		C6	K7ac/pS9/K11me3	A	G	G	K	A	G	Kac	D	pS	G	Kme3	A	K	T	K	A	V	S	R	S
		C7	K7ac/pS9/K11ac	A	G	G	K	A	G	Kac	D	pS	G	Kac	A	K	T	K	A	V	S	R	S
		C8	K11me/K13me	A	G	G	K	A	G	K	D	S	G	Kme	A	Kme	T	K	A	V	S	R	S
		C9	K11me/K13me2	A	G	G	K	A	G	K	D	S	G	Kme	A	Kme2	T	K	A	V	S	R	S
		C10	K11me/K13me3	A	G	G	K	A	G	K	D	S	G	Kme	A	Kme3	T	K	A	V	S	R	S
		C11	K11me/K13ac	A	G	G	K	A	G	K	D	S	G	Kme	A	Kac	T	K	A	V	S	R	S
		C12	K11me2/K13me	A	G	G	K	A	G	K	D	S	G	Kme2	A	Kme	T	K	A	V	S	R	S
		C13	K11me2/K13me2	A	G	G	K	A	G	K	D	S	G	Kme2	A	Kme2	T	K	A	V	S	R	S
		C14	K11me2/K13me3	A	G	G	K	A	G	K	D	S	G	Kme2	A	Kme3	T	K	A	V	S	R	S
		C15	K11me2/K13ac	A	G	G	K	A	G	K	D	S	G	Kme2	A	Kac	T	K	A	V	S	R	S
		C16	K11me3/K13me	A	G	G	K	A	G	K	D	S	G	Kme3	A	Kme	T	K	A	V	S	R	S
		C17	K11me3/K13me2	A	G	G	K	A	G	K	D	S	G	Kme3	A	Kme2	T	K	A	V	S	R	S
		C18	K11me3/K13me3	A	G	G	K	A	G	K	D	S	G	Kme3	A	Kme3	T	K	A	V	S	R	S
		C19	K11me3/K13ac	A	G	G	K	A	G	K	D	S	G	Kme3	A	Kac	T	K	A	V	S	R	S
		C20	K11ac/K13me	A	G	G	K	A	G	K	D	S	G	Kac	A	Kme	T	K	A	V	S	R	S
		C21	K11ac/K13me2	A	G	G	K	A	G	K	D	S	G	Kac	A	Kme2	T	K	A	V	S	R	S
		C22	K11ac/K13me3	A	G	G	K	A	G	K	D	S	G	Kac	A	Kme3	T	K	A	V	S	R	S
		C23	K11ac/K13ac	A	G	G	K	A	G	K	D	S	G	Kac	A	Kac	T	K	A	V	S	R	S
		C24																					
		C25	CONTROL	G	K	D	S	G	K	A	K	T	K	A	V	S	R	S	Q	R	A	G	L
		C26	K11me	G	K	D	S	G	Kme	A	K	T	K	A	V	S	R	S	Q	R	A	G	L
		C27	K11me2	G	K	D	S	G	Kme2	A	K	T	K	A	V	S	R	S	Q	R	A	G	L
		C28	K11me3	G	K	D	S	G	Kme3	A	K	T	K	A	V	S	R	S	Q	R	A	G	L
		C29	K11ac	G	K	D	S	G	Kac	A	K	T	K	A	V	S	R	S	Q	R	A	G	L
		C30	K13me	G	K	D	S	G	K	A	Kme	T	K	A	V	S	R	S	Q	R	A	G	L
		C31	K13me2	G	K	D	S	G	K	A	Kme2	T	K	A	V	S	R	S	Q	R	A	G	L
		C32	K13me3	G	K	D	S	G	K	A	Kme3	T	K	A	V	S	R	S	Q	R	A	G	L

**C33**	K13ac	G	K	D	S	G	K	A	Kac	T	K	A	V	S	R	S	Q	R	A	G	L
**C34**	pT14	G	K	D	S	G	K	A	K	pT	K	A	V	S	R	S	Q	R	A	G	L
**C35**	K15me	G	K	D	S	G	K	A	K	T	Kme	A	V	S	R	S	Q	R	A	G	L
**C36**	K15me2	G	K	D	S	G	K	A	K	T	Kme2	A	V	S	R	S	Q	R	A	G	L
**C37**	K15me3	G	K	D	S	G	K	A	K	T	Kme3	A	V	S	R	S	Q	R	A	G	L
**D1**	K15ac	G	K	D	S	G	K	A	K	T	Kac	A	V	S	R	S	Q	R	A	G	L
**D2**	K11me/K13me	G	K	D	S	G	Kme	A	Kme	T	K	A	V	S	R	S	Q	R	A	G	L
**D3**	K11me/K13me2	G	K	D	S	G	Kme	A	Kme2	T	K	A	V	S	R	S	Q	R	A	G	L
**D4**	K11me/K13me3	G	K	D	S	G	Kme	A	Kme3	T	K	A	V	S	R	S	Q	R	A	G	L
**D5**	K11me/K13ac	G	K	D	S	G	Kme	A	Kac	T	K	A	V	S	R	S	Q	R	A	G	L
**D6**	K11me2/K13me	G	K	D	S	G	Kme2	A	Kme	T	K	A	V	S	R	S	Q	R	A	G	L
**D7**	K11me2/K13me2	G	K	D	S	G	Kme2	A	Kme2	T	K	A	V	S	R	S	Q	R	A	G	L
**D8**	K11me2/K13me3	G	K	D	S	G	Kme2	A	Kme3	T	K	A	V	S	R	S	Q	R	A	G	L
**D9**	K11me2/K13ac	G	K	D	S	G	Kme2	A	Kac	T	K	A	V	S	R	S	Q	R	A	G	L
**D10**	K11me3/K13me	G	K	D	S	G	Kme3	A	Kme	T	K	A	V	S	R	S	Q	R	A	G	L
**D11**	K11me3/K13me2	G	K	D	S	G	Kme3	A	Kme2	T	K	A	V	S	R	S	Q	R	A	G	L
**D12**	K11me3/K13me3	G	K	D	S	G	Kme3	A	Kme3	T	K	A	V	S	R	S	Q	R	A	G	L
**D13**	K11me3/K13ac	G	K	D	S	G	Kme3	A	Kac	T	K	A	V	S	R	S	Q	R	A	G	L
**D14**	K11ac/K13me	G	K	D	S	G	Kac	A	Kme	T	K	A	V	S	R	S	Q	R	A	G	L
**D15**	K11ac/K13me2	G	K	D	S	G	Kac	A	Kme2	T	K	A	V	S	R	S	Q	R	A	G	L
**D16**	K11ac/K13me3	G	K	D	S	G	Kac	A	Kme3	T	K	A	V	S	R	S	Q	R	A	G	L
**D17**	K11ac/K13ac	G	K	D	S	G	Kac	A	Kac	T	K	A	V	S	R	S	Q	R	A	G	L
**D18**	K11me/K15me	G	K	D	S	G	Kme	A	K	T	Kme	A	V	S	R	S	Q	R	A	G	L
**D19**	K11me/K15me2	G	K	D	S	G	Kme	A	K	T	Kme2	A	V	S	R	S	Q	R	A	G	L
**D20**	K11me/K15me3	G	K	D	S	G	Kme	A	K	T	Kme3	A	V	S	R	S	Q	R	A	G	L
**D21**	K11me/K15ac	G	K	D	S	G	Kme	A	K	T	Kac	A	V	S	R	S	Q	R	A	G	L
**D22**	K11me2/K15me	G	K	D	S	G	Kme2	A	K	T	Kme	A	V	S	R	S	Q	R	A	G	L
**D23**	K11me2/K15me2	G	K	D	S	G	Kme2	A	K	T	Kme2	A	V	S	R	S	Q	R	A	G	L
**D24**	K11me2/K15me3	G	K	D	S	G	Kme2	A	K	T	Kme3	A	V	S	R	S	Q	R	A	G	L
**D25**	K11me2/K15ac	G	K	D	S	G	Kme2	A	K	T	Kac	A	V	S	R	S	Q	R	A	G	L
**D26**	K11me3/K15me	G	K	D	S	G	Kme3	A	K	T	Kme	A	V	S	R	S	Q	R	A	G	L
**D27**	K11me3/K15me2	G	K	D	S	G	Kme3	A	K	T	Kme2	A	V	S	R	S	Q	R	A	G	L
**D28**	K11me3/K15me3	G	K	D	S	G	Kme3	A	K	T	Kme3	A	V	S	R	S	Q	R	A	G	L
**D29**	K11me3/K15ac	G	K	D	S	G	Kme3	A	K	T	Kac	A	V	S	R	S	Q	R	A	G	L
**D30**	K11ac/K15me	G	K	D	S	G	Kac	A	K	T	Kme	A	V	S	R	S	Q	R	A	G	L
**D31**	K11ac/K15me2	G	K	D	S	G	Kac	A	K	T	Kme2	A	V	S	R	S	Q	R	A	G	L
**D32**	K11ac/K15me3	G	K	D	S	G	Kac	A	K	T	Kme3	A	V	S	R	S	Q	R	A	G	L
**D33**	K11ac/K15ac	G	K	D	S	G	Kac	A	K	T	Kac	A	V	S	R	S	Q	R	A	G	L
**D34**	K11me/pT14	G	K	D	S	G	Kme	A	K	pT	K	A	V	S	R	S	Q	R	A	G	L
**D35**	K11me2/pT14	G	K	D	S	G	Kme2	A	K	pT	K	A	V	S	R	S	Q	R	A	G	L
**D36**	K11me3/pT14	G	K	D	S	G	Kme3	A	K	pT	K	A	V	S	R	S	Q	R	A	G	L
**D37**	K11ac/pT14	G	K	D	S	G	Kac	A	K	pT	K	A	V	S	R	S	Q	R	A	G	L
**E1**	K13me/pT14	G	K	D	S	G	K	A	Kme	pT	K	A	V	S	R	S	Q	R	A	G	L
**E2**	K13me2/pT14	G	K	D	S	G	K	A	Kme2	pT	K	A	V	S	R	S	Q	R	A	G	L
**E3**	K13me3/pT14	G	K	D	S	G	K	A	Kme3	pT	K	A	V	S	R	S	Q	R	A	G	L
**E4**	K13ac/pT14	G	K	D	S	G	K	A	Kac	pT	K	A	V	S	R	S	Q	R	A	G	L
**E5**	pT14/K15me	G	K	D	S	G	K	A	K	pT	Kme	A	V	S	R	S	Q	R	A	G	L
**E6**	pT14/K15me2	G	K	D	S	G	K	A	K	pT	Kme2	A	V	S	R	S	Q	R	A	G	L
**E7**	pT14/K15me3	G	K	D	S	G	K	A	K	pT	Kme3	A	V	S	R	S	Q	R	A	G	L
**E8**	pT14/K15ac	G	K	D	S	G	K	A	K	pT	Kac	A	V	S	R	S	Q	R	A	G	L
**E9**	K11me/K13me/pT14	G	K	D	S	G	Kme	A	Kme	pT	K	A	V	S	R	S	Q	R	A	G	L
**E10**	K11me/K13me2/pT14	G	K	D	S	G	Kme	A	Kme2	pT	K	A	V	S	R	S	Q	R	A	G	L
**E11**	K11me/K13me3/pT14	G	K	D	S	G	Kme	A	Kme3	pT	K	A	V	S	R	S	Q	R	A	G	L
**E12**	K11me/K13ac/pT14	G	K	D	S	G	Kme	A	Kac	pT	K	A	V	S	R	S	Q	R	A	G	L
**E13**	K11me2/K13me/pT14	G	K	D	S	G	Kme2	A	Kme	pT	K	A	V	S	R	S	Q	R	A	G	L
**E14**	K11me2/K13me2/pT14	G	K	D	S	G	Kme2	A	Kme2	pT	K	A	V	S	R	S	Q	R	A	G	L
**E15**	K11me2/K13me3/pT14	G	K	D	S	G	Kme2	A	Kme3	pT	K	A	V	S	R	S	Q	R	A	G	L
**E16**	K11me2/K13ac/pT14	G	K	D	S	G	Kme2	A	Kac	pT	K	A	V	S	R	S	Q	R	A	G	L
**E17**	K11me3/K13me/pT14	G	K	D	S	G	Kme3	A	Kme	pT	K	A	V	S	R	S	Q	R	A	G	L
**E18**	K11me3/K13me2/pT14	G	K	D	S	G	Kme3	A	Kme2	pT	K	A	V	S	R	S	Q	R	A	G	L
**E19**	K11me3/K13me3/pT14	G	K	D	S	G	Kme3	A	Kme3	pT	K	A	V	S	R	S	Q	R	A	G	L
**E20**	K11me3/K13ac/pT14	G	K	D	S	G	Kme3	A	Kac	pT	K	A	V	S	R	S	Q	R	A	G	L
**E21**	K11ac/K13me/pT14	G	K	D	S	G	Kac	A	Kme	pT	K	A	V	S	R	S	Q	R	A	G	L
**E22**	K11ac/K13me2/pT14	G	K	D	S	G	Kac	A	Kme2	pT	K	A	V	S	R	S	Q	R	A	G	L
**E23**	K11ac/K13me3/pT14	G	K	D	S	G	Kac	A	Kme3	pT	K	A	V	S	R	S	Q	R	A	G	L
**E24**	K11ac/K13ac/pT14	G	K	D	S	G	Kac	A	Kac	pT	K	A	V	S	R	S	Q	R	A	G	L
**E25**	K11me/pT14/K15me	G	K	D	S	G	Kme	A	K	pT	Kme	A	V	S	R	S	Q	R	A	G	L
**E26**	K11me/pT14/K15me2	G	K	D	S	G	Kme	A	K	pT	Kme2	A	V	S	R	S	Q	R	A	G	L
**E27**	K11me/pT14/K15me3	G	K	D	S	G	Kme	A	K	pT	Kme3	A	V	S	R	S	Q	R	A	G	L
**E28**	K11me/pT14/K15ac	G	K	D	S	G	Kme	A	K	pT	Kac	A	V	S	R	S	Q	R	A	G	L
**E29**	K11me2/pT14/K15me	G	K	D	S	G	Kme2	A	K	pT	Kme	A	V	S	R	S	Q	R	A	G	L
**E30**	K11me2/pT14/K15me2	G	K	D	S	G	Kme2	A	K	pT	Kme2	A	V	S	R	S	Q	R	A	G	L
**E31**	K11me2/pT14/K15me3	G	K	D	S	G	Kme2	A	K	pT	Kme3	A	V	S	R	S	Q	R	A	G	L
**E32**	K11me2/pT14/K15ac	G	K	D	S	G	Kme2	A	K	pT	Kac	A	V	S	R	S	Q	R	A	G	L
**E33**	K11me3/pT14/K15me	G	K	D	S	G	Kme3	A	K	pT	Kme	A	V	S	R	S	Q	R	A	G	L
**E34**	K11me3/pT14/K15me2	G	K	D	S	G	Kme3	A	K	pT	Kme2	A	V	S	R	S	Q	R	A	G	L
**E35**	K11me3/pT14/K15me3	G	K	D	S	G	Kme3	A	K	pT	Kme3	A	V	S	R	S	Q	R	A	G	L
**E36**	K11me3/pT14/K15ac	G	K	D	S	G	Kme3	A	K	pT	Kac	A	V	S	R	S	Q	R	A	G	L
**E37**	K11ac/pT14/K15me	G	K	D	S	G	Kac	A	K	pT	Kme	A	V	S	R	S	Q	R	A	G	L
**F1**	K11ac/pT14/K15me2	G	K	D	S	G	Kac	A	K	pT	Kme2	A	V	S	R	S	Q	R	A	G	L
**F2**	K11ac/pT14/K15me3	G	K	D	S	G	Kac	A	K	pT	Kme3	A	V	S	R	S	Q	R	A	G	L
**F3**	K11ac/pT14/K15ac	G	K	D	S	G	Kac	A	K	pT	Kac	A	V	S	R	S	Q	R	A	G	L
**F4**	K11ac/K13ac/K15ac	G	K	D	S	G	Kac	A	Kac	T	Kac	A	V	S	R	S	Q	R	A	G	L
**F5**																					
**F6**	CONTROL	K	A	K	T	K	A	V	S	R	S	Q	R	A	G	L	Q	F	P	V	G
**F7**	K15me	K	A	K	T	Kme	A	V	S	R	S	Q	R	A	G	L	Q	F	P	V	G
**F8**	K15me2	K	A	K	T	Kme2	A	V	S	R	S	Q	R	A	G	L	Q	F	P	V	G
**F9**	K15me3	K	A	K	T	Kme3	A	V	S	R	S	Q	R	A	G	L	Q	F	P	V	G
**F10**	K15ac	K	A	K	T	Kac	A	V	S	R	S	Q	R	A	G	L	Q	F	P	V	G
**F11**	pS18	K	A	K	T	K	A	V	pS	R	S	Q	R	A	G	L	Q	F	P	V	G
**F12**	pS20	K	A	K	T	K	A	V	S	R	pS	Q	R	A	G	L	Q	F	P	V	G
**F13**	K15me/pS18	K	A	K	T	Kme	A	V	pS	R	S	Q	R	A	G	L	Q	F	P	V	G
**F14**	K15me2/pS18	K	A	K	T	Kme2	A	V	pS	R	S	Q	R	A	G	L	Q	F	P	V	G
**F15**	K15me3/pS18	K	A	K	T	Kme3	A	V	pS	R	S	Q	R	A	G	L	Q	F	P	V	G
**F16**	K15ac/pS18	K	A	K	T	Kac	A	V	pS	R	S	Q	R	A	G	L	Q	F	P	V	G
**F17**	K15me/pS20	K	A	K	T	Kme	A	V	S	R	pS	Q	R	A	G	L	Q	F	P	V	G
**F18**	K15me2/pS20	K	A	K	T	Kme2	A	V	S	R	pS	Q	R	A	G	L	Q	F	P	V	G
**F19**	K15me3/pS20	K	A	K	T	Kme3	A	V	S	R	pS	Q	R	A	G	L	Q	F	P	V	G
**F20**	K15ac/pS20	K	A	K	T	Kac	A	V	S	R	pS	Q	R	A	G	L	Q	F	P	V	G
**F21**	K15me/pS18/pS20	K	A	K	T	Kme	A	V	pS	R	pS	Q	R	A	G	L	Q	F	P	V	G
**F22**	K15me2/pS18/pS20	K	A	K	T	Kme2	A	V	pS	R	pS	Q	R	A	G	L	Q	F	P	V	G
**F23**	K15me3/pS18/pS20	K	A	K	T	Kme3	A	V	pS	R	pS	Q	R	A	G	L	Q	F	P	V	G
**F24**	K15ac/pS18/pS20	K	A	K	T	Kac	A	V	pS	R	pS	Q	R	A	G	L	Q	F	P	V	G
**F25**																					
**F26**	CONTROL	Q	F	P	V	G	R	I	H	R	H	L	K	S	R	T	T	S	H	G	R
**F27**	K37me	Q	F	P	V	G	R	I	H	R	H	L	Kme	S	R	T	T	S	H	G	R
**F28**	K37me2	Q	F	P	V	G	R	I	H	R	H	L	Kme2	S	R	T	T	S	H	G	R
**F29**	K37me3	Q	F	P	V	G	R	I	H	R	H	L	Kme3	S	R	T	T	S	H	G	R
**F30**	K37ac	Q	F	P	V	G	R	I	H	R	H	L	Kac	S	R	T	T	S	H	G	R

**F31**	pS38	Q	F	P	V	G	R	I	H	R	H	L	K	**pS**	R	T	T	S	H	G	R
**F32**	K37me/pS38	Q	F	P	V	G	R	I	H	R	H	L	**Kme**	**pS**	R	T	T	S	H	G	R
**F33**	K37me2/pS38	Q	F	P	V	G	R	I	H	R	H	L	**Kme2**	**pS**	R	T	T	S	H	G	R
**F34**	K37me3/pS38	Q	F	P	V	G	R	I	H	R	H	L	**Kme3**	**pS**	R	T	T	S	H	G	R
**F35**	K37ac/pS38	Q	F	P	V	G	R	I	H	R	H	L	**Kac**	**pS**	R	T	T	S	H	G	R

Histone	Peptide Length	Array Poition	Kac Mark Combinations	Sequence																			
polyHis CTRL		A1	HHHHHHHH																				
polyHis CTRL		A2	HHHHHHHH																				
		A3	Blank																				
								5				9				13		15					
H2B (Q6FI13)	1-20	A4	CONTROL	P	E	P	A	K	S	A	P	A	P	K	K	G	S	K	K	A	V	T	K
		A5	K5me	P	E	P	A	Kme	S	A	P	A	P	K	K	G	S	K	K	A	V	T	K
		A6	K5me2	P	E	P	A	Kme2	S	A	P	A	P	K	K	G	S	K	K	A	V	T	K
		A7	K5me3	P	E	P	A	Kme3	S	A	P	A	P	K	K	G	S	K	K	A	V	T	K
		A8	K5ac	P	E	P	A	Kac	S	A	P	A	P	K	K	G	S	K	K	A	V	T	K
		A9	pS6	P	E	P	A	K	pS	A	P	A	P	K	K	G	S	K	K	A	V	T	K
		A10	K11me	P	E	P	A	K	S	A	P	A	P	Kme	K	G	S	K	K	A	V	T	K
		A11	K11me2	P	E	P	A	K	S	A	P	A	P	Kme2	K	G	S	K	K	A	V	T	K
		A12	K11me3	P	E	P	A	K	S	A	P	A	P	Kme3	K	G	S	K	K	A	V	T	K
		A13	K11ac	P	E	P	A	K	S	A	P	A	P	Kac	K	G	S	K	K	A	V	T	K
		A14	K12me	P	E	P	A	K	S	A	P	A	P	K	Kme	G	S	K	K	A	V	T	K
		A15	K12me2	P	E	P	A	K	S	A	P	A	P	K	Kme2	G	S	K	K	A	V	T	K
		A16	K12me3	P	E	P	A	K	S	A	P	A	P	K	Kme3	G	S	K	K	A	V	T	K
		A17	K12ac	P	E	P	A	K	S	A	P	A	P	K	Kac	G	S	K	K	A	V	T	K
		A18	pS14	P	E	P	A	K	S	A	P	A	P	K	K	G	pS	K	K	A	V	T	K
		A19	K5me/pS6	P	E	P	A	Kme	pS	A	P	A	P	K	K	G	S	K	K	A	V	T	K
		A20	K5me2/pS6	P	E	P	A	Kme2	pS	A	P	A	P	K	K	G	S	K	K	A	V	T	K
		A21	K5me3/pS6	P	E	P	A	Kme3	pS	A	P	A	P	K	K	G	S	K	K	A	V	T	K
		A22	K5ac/pS6	P	E	P	A	Kac	pS	A	P	A	P	K	K	G	S	K	K	A	V	T	K
		A23	K5me/K11me	P	E	P	A	Kme	S	A	P	A	P	Kme	K	G	S	K	K	A	V	T	K
		A24	K5me/K11me2	P	E	P	A	Kme	S	A	P	A	P	Kme2	K	G	S	K	K	A	V	T	K
		A25	K5me/K11me3	P	E	P	A	Kme	S	A	P	A	P	Kme3	K	G	S	K	K	A	V	T	K
		A26	K5me/K11ac	P	E	P	A	Kme	S	A	P	A	P	Kac	K	G	S	K	K	A	V	T	K
		A27	K5me2/K11me	P	E	P	A	Kme2	S	A	P	A	P	Kme	K	G	S	K	K	A	V	T	K
		A28	K5me2/K11me2	P	E	P	A	Kme2	S	A	P	A	P	Kme2	K	G	S	K	K	A	V	T	K
		A29	K5me2/K11me3	P	E	P	A	Kme2	S	A	P	A	P	Kme3	K	G	S	K	K	A	V	T	K
		A30	K5me2/K11ac	P	E	P	A	Kme2	S	A	P	A	P	Kac	K	G	S	K	K	A	V	T	K
		A31	K5me3/K11me	P	E	P	A	Kme3	S	A	P	A	P	Kme	K	G	S	K	K	A	V	T	K
		A32	K5me3/K11me2	P	E	P	A	Kme3	S	A	P	A	P	Kme2	K	G	S	K	K	A	V	T	K
		A33	K5me3/K11me3	P	E	P	A	Kme3	S	A	P	A	P	Kme3	K	G	S	K	K	A	V	T	K
		A34	K5me3/K11ac	P	E	P	A	Kme3	S	A	P	A	P	Kac	K	G	S	K	K	A	V	T	K
		A35	K5ac/K11me	P	E	P	A	Kac	S	A	P	A	P	Kme	K	G	S	K	K	A	V	T	K
		A36	K5ac/K11me2	P	E	P	A	Kac	S	A	P	A	P	Kme2	K	G	S	K	K	A	V	T	K
		A37	K5ac/K11me3	P	E	P	A	Kac	S	A	P	A	P	Kme3	K	G	S	K	K	A	V	T	K
		B1	K5ac/K11ac	P	E	P	A	Kac	S	A	P	A	P	Kac	K	G	S	K	K	A	V	T	K
		B2	K5me/pS6/K11me	P	E	P	A	Kme	pS	A	P	A	P	Kme	K	G	S	K	K	A	V	T	K
		B3	K5me/pS6/K11me2	P	E	P	A	Kme	pS	A	P	A	P	Kme2	K	G	S	K	K	A	V	T	K
		B4	K5me/pS6/K11me3	P	E	P	A	Kme	pS	A	P	A	P	Kme3	K	G	S	K	K	A	V	T	K
		B5	K5me/pS6/K11ac	P	E	P	A	Kme	pS	A	P	A	P	Kac	K	G	S	K	K	A	V	T	K
		B6	K5me2/pS6/K11me	P	E	P	A	Kme2	pS	A	P	A	P	Kme	K	G	S	K	K	A	V	T	K
		B7	K5me2/pS6/K11me2	P	E	P	A	Kme2	pS	A	P	A	P	Kme2	K	G	S	K	K	A	V	T	K
		B8	K5me2/pS6/K11me3	P	E	P	A	Kme2	pS	A	P	A	P	Kme3	K	G	S	K	K	A	V	T	K
		B9	K5me2/pS6/K11ac	P	E	P	A	Kme2	pS	A	P	A	P	Kac	K	G	S	K	K	A	V	T	K
		B10	K5me3/pS6/K11me	P	E	P	A	Kme3	pS	A	P	A	P	Kme	K	G	S	K	K	A	V	T	K
		B11	K5me3/pS6/K11me2	P	E	P	A	Kme3	pS	A	P	A	P	Kme2	K	G	S	K	K	A	V	T	K
		B12	K5me3/pS6/K11me3	P	E	P	A	Kme3	pS	A	P	A	P	Kme3	K	G	S	K	K	A	V	T	K
		B13	K5me3/pS6/K11ac	P	E	P	A	Kme3	pS	A	P	A	P	Kac	K	G	S	K	K	A	V	T	K
		B14	K5ac/pS6/K11me	P	E	P	A	Kac	pS	A	P	A	P	Kme	K	G	S	K	K	A	V	T	K
		B15	K5ac/pS6/K11me2	P	E	P	A	Kac	pS	A	P	A	P	Kme2	K	G	S	K	K	A	V	T	K
		B16	K5ac/pS6/K11me3	P	E	P	A	Kac	pS	A	P	A	P	Kme3	K	G	S	K	K	A	V	T	K
		B17	K5ac/pS6/K11ac	P	E	P	A	Kac	pS	A	P	A	P	Kac	K	G	S	K	K	A	V	T	K
	6-25	B18	K11me/K12me	P	E	P	A	K	S	A	P	A	P	Kme	Kme	G	S	K	K	A	V	T	K
		B19	K11me/K12me2	P	E	P	A	K	S	A	P	A	P	Kme	Kme2	G	S	K	K	A	V	T	K
		B20	K11me/K12me3	P	E	P	A	K	S	A	P	A	P	Kme	Kme3	G	S	K	K	A	V	T	K
		B21	K11me/K12ac	P	E	P	A	K	S	A	P	A	P	Kme	Kac	G	S	K	K	A	V	T	K
		B22	K11me2/K12me	P	E	P	A	K	S	A	P	A	P	Kme2	Kme	G	S	K	K	A	V	T	K
		B23	K11me2/K12me2	P	E	P	A	K	S	A	P	A	P	Kme2	Kme2	G	S	K	K	A	V	T	K
		B24	K11me2/K12me3	P	E	P	A	K	S	A	P	A	P	Kme2	Kme3	G	S	K	K	A	V	T	K
		B25	K11me2/K12ac	P	E	P	A	K	S	A	P	A	P	Kme2	Kac	G	S	K	K	A	V	T	K
		B26	K11me3/K12me	P	E	P	A	K	S	A	P	A	P	Kme3	Kme	G	S	K	K	A	V	T	K
		B27	K11me3/K12me2	P	E	P	A	K	S	A	P	A	P	Kme3	Kme2	G	S	K	K	A	V	T	K
		B28	K11me3/K12me3	P	E	P	A	K	S	A	P	A	P	Kme3	Kme3	G	S	K	K	A	V	T	K
		B29	K11me3/K12ac	P	E	P	A	K	S	A	P	A	P	Kme3	Kac	G	S	K	K	A	V	T	K
		B30	K11ac/K12me	P	E	P	A	K	S	A	P	A	P	Kac	Kme	G	S	K	K	A	V	T	K
		B31	K11ac/K12me2	P	E	P	A	K	S	A	P	A	P	Kac	Kme2	G	S	K	K	A	V	T	K
		B32	K11ac/K12me3	P	E	P	A	K	S	A	P	A	P	Kac	Kme3	G	S	K	K	A	V	T	K
		B33	K11ac/K12ac	P	E	P	A	K	S	A	P	A	P	Kac	Kac	G	S	K	K	A	V	T	K
		B34	K11me/K12me/pS14	P	E	P	A	K	S	A	P	A	P	Kme	Kme	G	pS	K	K	A	V	T	K
		B35	K11me/K12me2/pS14	P	E	P	A	K	S	A	P	A	P	Kme	Kme2	G	pS	K	K	A	V	T	K
		B36	K11me/K12me3/pS14	P	E	P	A	K	S	A	P	A	P	Kme	Kme3	G	pS	K	K	A	V	T	K
		B37	K11me/K12ac/pS14	P	E	P	A	K	S	A	P	A	P	Kme	Kac	G	pS	K	K	A	V	T	K
		C1	K11me2/K12me/pS14	P	E	P	A	K	S	A	P	A	P	Kme2	Kme	G	pS	K	K	A	V	T	K
		C2	K11me2/K12me2/pS14	P	E	P	A	K	S	A	P	A	P	Kme2	Kme2	G	pS	K	K	A	V	T	K
		C3	K11me2/K12me3/pS14	P	E	P	A	K	S	A	P	A	P	Kme2	Kme3	G	pS	K	K	A	V	T	K
		C4	K11me2/K12ac/pS14	P	E	P	A	K	S	A	P	A	P	Kme2	Kac	G	pS	K	K	A	V	T	K
		C5	K11me3/K12me/pS14	P	E	P	A	K	S	A	P	A	P	Kme3	Kme	G	pS	K	K	A	V	T	K
		C6	K11me3/K12me2/pS14	P	E	P	A	K	S	A	P	A	P	Kme3	Kme2	G	pS	K	K	A	V	T	K
		C7	K11me3/K12me3/pS14	P	E	P	A	K	S	A	P	A	P	Kme3	Kme3	G	pS	K	K	A	V	T	K
		C8	K11me3/K12ac/pS14	P	E	P	A	K	S	A	P	A	P	Kme3	Kac	G	pS	K	K	A	V	T	K
		C9	K11ac/K12me/pS14	P	E	P	A	K	S	A	P	A	P	Kac	Kme	G	pS	K	K	A	V	T	K
		C10	K11ac/K12me2/pS14	P	E	P	A	K	S	A	P	A	P	Kac	Kme2	G	pS	K	K	A	V	T	K
		C11	K11ac/K12me3/pS14	P	E	P	A	K	S	A	P	A	P	Kac	Kme3	G	pS	K	K	A	V	T	K
		C12	K11ac/K12ac/pS14	P	E	P	A	K	S	A	P	A	P	Kac	Kac	G	pS	K	K	A	V	T	K
		C13	blank																				
		C14	CONTROL	S	A	P	A	P	K	K	G	S	K	K	A	V	T	K	A	Q	K	K	D
		C15	K11me	S	A	P	A	P	Kme	K	G	S	K	K	A	V	T	K	A	Q	K	K	D
		C16	K11me2	S	A	P	A	P	Kme2	K	G	S	K	K	A	V	T	K	A	Q	K	K	D
		C17	K11me3	S	A	P	A	P	Kme3	K	G	S	K	K	A	V	T	K	A	Q	K	K	D
		C18	K11ac	S	A	P	A	P	Kac	K	G	S	K	K	A	V	T	K	A	Q	K	K	D
		C19	K12me	S	A	P	A	P	K	Kme	G	S	K	K	A	V	T	K	A	Q	K	K	D
		C20	K12me2	S	A	P	A	P	K	Kme2	G	S	K	K	A	V	T	K	A	Q	K	K	D
		C21	K12me3	S	A	P	A	P	K	Kme3	G	S	K	K	A	V	T	K	A	Q	K	K	D
		C22	K12ac	S	A	P	A	P	K	Kac	G	S	K	K	A	V	T	K	A	Q	K	K	D
		C23	pS14	S	A	P	A	P	K	K	G	pS	K	K	A	V	T	K	A	Q	K	K	D
		C24	K15me	S	A	P	A	P	K	K	G	S	Kme	K	A	V	T	K	A	Q	K	K	D
		C25	K15me2	S	A	P	A	P	K	K	G	S	Kme2	K	A	V	T	K	A	Q	K	K	D
		C26	K15me3	S	A	P	A	P	K	K	G	S	Kme3	K	A	V	T	K	A	Q	K	K	D
		C27	K15ac	S	A	P	A	P	K	K	G	S	Kac	K	A	V	T	K	A	Q	K	K	D
		C28	K16me	S	A	P	A	P	K	K	G	S	K	Kme	A	V	T	K	A	Q	K	K	D
		C29	K16me2	S	A	P	A	P	K	K	G	S	K	Kme2	A	V	T	K	A	Q	K	K	D
		C30	K16me3	S	A	P	A	P	K	K	G	S	K	Kme3	A	V	T	K	A	Q	K	K	D
		C31	K16ac	S	A	P	A	P	K	K	G	S	K	Kac	A	V	T	K	A	Q	K	K	D
		C32	pT19	S	A	P	A	P	K	K	G	S	K	K	A	V	pT	K	A	Q	K	K	D
		C33	K11me/K12me	S	A	P	A	P	Kme	Kme	G	S	K	K	A	V	T	K	A	Q	K	K	D
		C34	K11me/K12me2	S	A	P	A	P	Kme	Kme2	G	S	K	K	A	V	T	K	A	Q	K	K	D
		C35	K11me/K12me3	S	A	P	A	P	Kme	Kme3	G	S	K	K	A	V	T	K	A	Q	K	K	D
		C36	K11me/K12ac	S	A	P	A	P	Kme	Kac	G	S	K	K	A	V	T	K	A	Q	K	K	D
		C37	K11me2/K12me	S	A	P	A	P	Kme2	Kme	G	S	K	K	A	V	T	K	A	Q	K	K	D
		D1	K11me2/K12me2	S	A	P	A	P	Kme2	Kme2	G	S	K	K	A	V	T	K	A	Q	K	K	D

**D2**	K11me2/K12me3	S	A	P	A	P	Kme2	Kme3	G	S	K	K	A	V	T	K	A	Q	K	K	D
**D3**	K11me2/K12ac	S	A	P	A	P	Kme2	Kac	G	S	K	K	A	V	T	K	A	Q	K	K	D
**D4**	K11me3/K12me	S	A	P	A	P	Kme3	Kme	G	S	K	K	A	V	T	K	A	Q	K	K	D
**D5**	K11me3/K12me2	S	A	P	A	P	Kme3	Kme2	G	S	K	K	A	V	T	K	A	Q	K	K	D
**D6**	K11me3/K12me3	S	A	P	A	P	Kme3	Kme3	G	S	K	K	A	V	T	K	A	Q	K	K	D
**D7**	K11me3/K12ac	S	A	P	A	P	Kme3	Kac	G	S	K	K	A	V	T	K	A	Q	K	K	D
**D8**	K11ac/K12me	S	A	P	A	P	Kac	Kme	G	S	K	K	A	V	T	K	A	Q	K	K	D
**D9**	K11ac/K12me2	S	A	P	A	P	Kac	Kme2	G	S	K	K	A	V	T	K	A	Q	K	K	D
**D10**	K11ac/K12me3	S	A	P	A	P	Kac	Kme3	G	S	K	K	A	V	T	K	A	Q	K	K	D
**D11**	K11ac/K12ac	S	A	P	A	P	Kac	Kac	G	S	K	K	A	V	T	K	A	Q	K	K	D
**D12**	K11me/K12me/pS14	S	A	P	A	P	Kme	Kme	G	pS	K	K	A	V	T	K	A	Q	K	K	D
**D13**	K11me/K12me2/pS14	S	A	P	A	P	Kme	Kme2	G	pS	K	K	A	V	T	K	A	Q	K	K	D
**D14**	K11me/K12me3/pS14	S	A	P	A	P	Kme	Kme3	G	pS	K	K	A	V	T	K	A	Q	K	K	D
**D15**	K11me/K12ac/pS14	S	A	P	A	P	Kme	Kac	G	pS	K	K	A	V	T	K	A	Q	K	K	D
**D16**	K11me2/K12me/pS14	S	A	P	A	P	Kme2	Kme	G	pS	K	K	A	V	T	K	A	Q	K	K	D
**D17**	K11me2/K12me2/pS14	S	A	P	A	P	Kme2	Kme2	G	pS	K	K	A	V	T	K	A	Q	K	K	D
**D18**	K11me2/K12me3/pS14	S	A	P	A	P	Kme2	Kme3	G	pS	K	K	A	V	T	K	A	Q	K	K	D
**D19**	K11me2/K12ac/pS14	S	A	P	A	P	Kme2	Kac	G	pS	K	K	A	V	T	K	A	Q	K	K	D
**D20**	K11me3/K12me/pS14	S	A	P	A	P	Kme3	Kme	G	pS	K	K	A	V	T	K	A	Q	K	K	D
**D21**	K11me3/K12me2/pS14	S	A	P	A	P	Kme3	Kme2	G	pS	K	K	A	V	T	K	A	Q	K	K	D
**D22**	K11me3/K12me3/pS14	S	A	P	A	P	Kme3	Kme3	G	pS	K	K	A	V	T	K	A	Q	K	K	D
**D23**	K11me3/K12ac/pS14	S	A	P	A	P	Kme3	Kac	G	pS	K	K	A	V	T	K	A	Q	K	K	D
**D24**	K11ac/K12me/pS14	S	A	P	A	P	Kac	Kme	G	pS	K	K	A	V	T	K	A	Q	K	K	D
**D25**	K11ac/K12me2/pS14	S	A	P	A	P	Kac	Kme2	G	pS	K	K	A	V	T	K	A	Q	K	K	D
**D26**	K11ac/K12me3/pS14	S	A	P	A	P	Kac	Kme3	G	pS	K	K	A	V	T	K	A	Q	K	K	D
**D27**	K11ac/K12ac/pS14	S	A	P	A	P	Kac	Kac	G	pS	K	K	A	V	T	K	A	Q	K	K	D
**D28**	K15me/K16me	S	A	P	A	P	K	K	G	S	Kme	Kme	A	V	T	K	A	Q	K	K	D
**D29**	K15me/K16me2	S	A	P	A	P	K	K	G	S	Kme	Kme2	A	V	T	K	A	Q	K	K	D
**D30**	K15me/K16me3	S	A	P	A	P	K	K	G	S	Kme	Kme3	A	V	T	K	A	Q	K	K	D
**D31**	K15me/K16ac	S	A	P	A	P	K	K	G	S	Kme	Kac	A	V	T	K	A	Q	K	K	D
**D32**	K15me2/K16me	S	A	P	A	P	K	K	G	S	Kme2	Kme	A	V	T	K	A	Q	K	K	D
**D33**	K15me2/K16me2	S	A	P	A	P	K	K	G	S	Kme2	Kme2	A	V	T	K	A	Q	K	K	D
**D34**	K15me2/K16me3	S	A	P	A	P	K	K	G	S	Kme2	Kme3	A	V	T	K	A	Q	K	K	D
**D35**	K15me2/K16ac	S	A	P	A	P	K	K	G	S	Kme2	Kac	A	V	T	K	A	Q	K	K	D
**D36**	K15me3/K16me	S	A	P	A	P	K	K	G	S	Kme3	Kme	A	V	T	K	A	Q	K	K	D
**D37**	K15me3/K16me2	S	A	P	A	P	K	K	G	S	Kme3	Kme2	A	V	T	K	A	Q	K	K	D
**E1**	K15me3/K16me3	S	A	P	A	P	K	K	G	S	Kme3	Kme3	A	V	T	K	A	Q	K	K	D
**E2**	K15me3/K16ac	S	A	P	A	P	K	K	G	S	Kme3	Kac	A	V	T	K	A	Q	K	K	D
**E3**	K15ac/K16me	S	A	P	A	P	K	K	G	S	Kac	Kme	A	V	T	K	A	Q	K	K	D
**E4**	K15ac/K16me2	S	A	P	A	P	K	K	G	S	Kac	Kme2	A	V	T	K	A	Q	K	K	D
**E5**	K15ac/K16me3	S	A	P	A	P	K	K	G	S	Kac	Kme3	A	V	T	K	A	Q	K	K	D
**E6**	K15ac/K16ac	S	A	P	A	P	K	K	G	S	Kac	Kac	A	V	T	K	A	Q	K	K	D
**E7**	pS14/K15me/K16me	S	A	P	A	P	K	K	G	pS	Kme	Kme	A	V	T	K	A	Q	K	K	D
**E8**	pS14/K15me/K16me2	S	A	P	A	P	K	K	G	pS	Kme	Kme2	A	V	T	K	A	Q	K	K	D
**E9**	pS14/K15me/K16me3	S	A	P	A	P	K	K	G	pS	Kme	Kme3	A	V	T	K	A	Q	K	K	D
**E10**	pS14/K15me/K16ac	S	A	P	A	P	K	K	G	pS	Kme	Kac	A	V	T	K	A	Q	K	K	D
**E11**	pS14/K15me2/K16me	S	A	P	A	P	K	K	G	pS	Kme2	Kme	A	V	T	K	A	Q	K	K	D
**E12**	pS14/K15me2/K16me2	S	A	P	A	P	K	K	G	pS	Kme2	Kme2	A	V	T	K	A	Q	K	K	D
**E13**	pS14/K15me2/K16me3	S	A	P	A	P	K	K	G	pS	Kme2	Kme3	A	V	T	K	A	Q	K	K	D
**E14**	pS14/K15me2/K16ac	S	A	P	A	P	K	K	G	pS	Kme2	Kac	A	V	T	K	A	Q	K	K	D
**E15**	pS14/K15me3/K16me	S	A	P	A	P	K	K	G	pS	Kme3	Kme	A	V	T	K	A	Q	K	K	D
**E16**	pS14/K15me3/K16me2	S	A	P	A	P	K	K	G	pS	Kme3	Kme2	A	V	T	K	A	Q	K	K	D
**E17**	pS14/K15me3/K16me3	S	A	P	A	P	K	K	G	pS	Kme3	Kme3	A	V	T	K	A	Q	K	K	D
**E18**	pS14/K15me3/K16ac	S	A	P	A	P	K	K	G	pS	Kme3	Kac	A	V	T	K	A	Q	K	K	D
**E19**	pS14/K15ac/K16me	S	A	P	A	P	K	K	G	pS	Kac	Kme	A	V	T	K	A	Q	K	K	D
**E20**	pS14/K15ac/K16me2	S	A	P	A	P	K	K	G	pS	Kac	Kme2	A	V	T	K	A	Q	K	K	D
**E21**	pS14/K15ac/K16me3	S	A	P	A	P	K	K	G	pS	Kac	Kme3	A	V	T	K	A	Q	K	K	D
**E22**	pS14/K15ac/K16ac	S	A	P	A	P	K	K	G	pS	Kac	Kac	A	V	T	K	A	Q	K	K	D
**E23**	K15me/K16me/pT19	S	A	P	A	P	K	K	G	S	Kme	Kme	A	V	pT	K	A	Q	K	K	D
**E24**	K15me/K16me2/pT19	S	A	P	A	P	K	K	G	S	Kme	Kme2	A	V	pT	K	A	Q	K	K	D
**E25**	K15me/K16me3/pT19	S	A	P	A	P	K	K	G	S	Kme	Kme3	A	V	pT	K	A	Q	K	K	D
**E26**	K15me/K16ac/pT19	S	A	P	A	P	K	K	G	S	Kme	Kac	A	V	pT	K	A	Q	K	K	D
**E27**	K15me2/K16me/pT19	S	A	P	A	P	K	K	G	S	Kme2	Kme	A	V	pT	K	A	Q	K	K	D
**E28**	K15me2/K16me2/pT19	S	A	P	A	P	K	K	G	S	Kme2	Kme2	A	V	pT	K	A	Q	K	K	D
**E29**	K15me2/K16me3/pT19	S	A	P	A	P	K	K	G	S	Kme2	Kme3	A	V	pT	K	A	Q	K	K	D
**E30**	K15me2/K16ac/pT19	S	A	P	A	P	K	K	G	S	Kme2	Kac	A	V	pT	K	A	Q	K	K	D
**E31**	K15me3/K16me/pT19	S	A	P	A	P	K	K	G	S	Kme3	Kme	A	V	pT	K	A	Q	K	K	D
**E32**	K15me3/K16me2/pT19	S	A	P	A	P	K	K	G	S	Kme3	Kme2	A	V	pT	K	A	Q	K	K	D
**E33**	K15me3/K16me3/pT19	S	A	P	A	P	K	K	G	S	Kme3	Kme3	A	V	pT	K	A	Q	K	K	D
**E34**	K15me3/K16ac/pT19	S	A	P	A	P	K	K	G	S	Kme3	Kac	A	V	pT	K	A	Q	K	K	D
**E35**	K15ac/K16me/pT19	S	A	P	A	P	K	K	G	S	Kac	Kme	A	V	pT	K	A	Q	K	K	D
**E36**	K15ac/K16me2/pT19	S	A	P	A	P	K	K	G	S	Kac	Kme2	A	V	pT	K	A	Q	K	K	D
**E37**	K15ac/K16me3/pT19	S	A	P	A	P	K	K	G	S	Kac	Kme3	A	V	pT	K	A	Q	K	K	D
**F1**	K15ac/K16ac/pT19	S	A	P	A	P	K	K	G	S	Kac	Kac	A	V	pT	K	A	Q	K	K	D
**F2**	Blank																				
**F3**	CONTROL	K	K	G	S	K	K	A	V	T	K	A	Q	K	K	D	G	K	K	R	K
**F4**	K15me	K	K	G	S	Kme	K	A	V	T	K	A	Q	K	K	D	G	K	K	R	K
**F5**	K15me2	K	K	G	S	Kme2	K	A	V	T	K	A	Q	K	K	D	G	K	K	R	K
**F6**	K15me3	K	K	G	S	Kme3	K	A	V	T	K	A	Q	K	K	D	G	K	K	R	K
**F7**	K15ac	K	K	G	S	Kac	K	A	V	T	K	A	Q	K	K	D	G	K	K	R	K
**F8**	K16me	K	K	G	S	K	Kme	A	V	T	K	A	Q	K	K	D	G	K	K	R	K
**F9**	K16me2	K	K	G	S	K	Kme2	A	V	T	K	A	Q	K	K	D	G	K	K	R	K
**F10**	K16me3	K	K	G	S	K	Kme3	A	V	T	K	A	Q	K	K	D	G	K	K	R	K
**F11**	K16ac	K	K	G	S	K	Kac	A	V	T	K	A	Q	K	K	D	G	K	K	R	K
**F12**	pT19	K	K	G	S	K	K	A	V	pT	K	A	Q	K	K	D	G	K	K	R	K
**F13**	K20me	K	K	G	S	K	K	A	V	T	Kme	A	Q	K	K	D	G	K	K	R	K
**F14**	K20me2	K	K	G	S	K	K	A	V	T	Kme2	A	Q	K	K	D	G	K	K	R	K
**F15**	K20me3	K	K	G	S	K	K	A	V	T	Kme3	A	Q	K	K	D	G	K	K	R	K
**F16**	K20ac	K	K	G	S	K	K	A	V	T	Kac	A	Q	K	K	D	G	K	K	R	K
**F17**	K23me	K	K	G	S	K	K	A	V	T	K	A	Q	Kme	K	D	G	K	K	R	K
**F18**	K23me2	K	K	G	S	K	K	A	V	T	K	A	Q	Kme2	K	D	G	K	K	R	K
**F19**	K23me3	K	K	G	S	K	K	A	V	T	K	A	Q	Kme3	K	D	G	K	K	R	K
**F20**	K23ac	K	K	G	S	K	K	A	V	T	K	A	Q	Kac	K	D	G	K	K	R	K
**F21**	pT19/K20me	K	K	G	S	K	K	A	V	pT	Kme	A	Q	K	K	D	G	K	K	R	K
**F22**	pT19/K20me2	K	K	G	S	K	K	A	V	pT	Kme2	A	Q	K	K	D	G	K	K	R	K
**F23**	pT19/K20me3	K	K	G	S	K	K	A	V	pT	Kme3	A	Q	K	K	D	G	K	K	R	K
**F24**	pT19/K20ac	K	K	G	S	K	K	A	V	pT	Kac	A	Q	K	K	D	G	K	K	R	K
**F25**	pT19/K23me	K	K	G	S	K	K	A	V	pT	K	A	Q	Kme	K	D	G	K	K	R	K
**F26**	pT19/K23me2	K	K	G	S	K	K	A	V	pT	K	A	Q	Kme2	K	D	G	K	K	R	K
**F27**	pT19/K23me3	K	K	G	S	K	K	A	V	pT	K	A	Q	Kme3	K	D	G	K	K	R	K
**F28**	pT19/K23ac	K	K	G	S	K	K	A	V	pT	K	A	Q	Kac	K	D	G	K	K	R	K
**F29**	K20me/K23me	K	K	G	S	K	K	A	V	T	Kme	A	Q	Kme	K	D	G	K	K	R	K
**F30**	K20me/K23me2	K	K	G	S	K	K	A	V	T	Kme	A	Q	Kme2	K	D	G	K	K	R	K
**F31**	K20me/K23me3	K	K	G	S	K	K	A	V	T	Kme	A	Q	Kme3	K	D	G	K	K	R	K
**F32**	K20me/K23ac	K	K	G	S	K	K	A	V	T	Kme	A	Q	Kac	K	D	G	K	K	R	K
**F33**	K20me2/K23me	K	K	G	S	K	K	A	V	T	Kme2	A	Q	Kme	K	D	G	K	K	R	K
**F34**	K20me2/K23me2	K	K	G	S	K	K	A	V	T	Kme2	A	Q	Kme2	K	D	G	K	K	R	K
**F35**	K20me2/K23me3	K	K	G	S	K	K	A	V	T	Kme2	A	Q	Kme3	K	D	G	K	K	R	K
**F36**	K20me2/K23ac	K	K	G	S	K	K	A	V	T	Kme2	A	Q	Kac	K	D	G	K	K	R	K
**F37**	K20me3/K23me	K	K	G	S	K	K	A	V	T	Kme3	A	Q	Kme	K	D	G	K	K	R	K
**G1**	K20me3/K23me2	K	K	G	S	K	K	A	V	T	Kme3	A	Q	Kme2	K	D	G	K	K	R	K
**G2**	K20me3/K23me3	K	K	G	S	K	K	A	V	T	Kme3	A	Q	Kme3	K	D	G	K	K	R	K
**G3**	K20me3/K23ac	K	K	G	S	K	K	A	V	T	Kme3	A	Q	Kac	K	D	G	K	K	R	K
**G4**	K20ac/K23me	K	K	G	S	K	K	A	V	T	Kac	A	Q	Kme	K	D	G	K	K	R	K
**G5**	K20ac/K23me2	K	K	G	S	K	K	A	V	T	Kac	A	Q	Kme2	K	D	G	K	K	R	K

G6	K20ac/K23me3	K	K	G	S	K	K	A	V	T	Kac	A	Q	Kme3	K	D	G	K	K	R	K
G7	K20ac/K23ac	K	K	G	S	K	K	A	V	T	Kac	A	Q	Kac	K	D	G	K	K	R	K
G8	pT19/K20me/K23me	K	K	G	S	K	K	A	V	pT	Kme	A	Q	Kme	K	D	G	K	K	R	K
G9	pT19/K20me/K23me2	K	K	G	S	K	K	A	V	pT	Kme	A	Q	Kme2	K	D	G	K	K	R	K
G10	pT19/K20me/K23me3	K	K	G	S	K	K	A	V	pT	Kme	A	Q	Kme3	K	D	G	K	K	R	K
G11	pT19/K20me/K23ac	K	K	G	S	K	K	A	V	pT	Kme	A	Q	Kac	K	D	G	K	K	R	K
G12	pT19/K20me2/K23me	K	K	G	S	K	K	A	V	pT	Kme2	A	Q	Kme	K	D	G	K	K	R	K
G13	pT19/K20me2/K23me2	K	K	G	S	K	K	A	V	pT	Kme2	A	Q	Kme2	K	D	G	K	K	R	K
G14	pT19/K20me2/K23me3	K	K	G	S	K	K	A	V	pT	Kme2	A	Q	Kme3	K	D	G	K	K	R	K
G15	pT19/K20me2/K23ac	K	K	G	S	K	K	A	V	pT	Kme2	A	Q	Kac	K	D	G	K	K	R	K
G16	pT19/K20me3/K23me	K	K	G	S	K	K	A	V	pT	Kme3	A	Q	Kme	K	D	G	K	K	R	K
G17	pT19/K20me3/K23me2	K	K	G	S	K	K	A	V	pT	Kme3	A	Q	Kme2	K	D	G	K	K	R	K
G18	pT19/K20me3/K23me3	K	K	G	S	K	K	A	V	pT	Kme3	A	Q	Kme3	K	D	G	K	K	R	K
G19	pT19/K20me3/K23ac	K	K	G	S	K	K	A	V	pT	Kme3	A	Q	Kac	K	D	G	K	K	R	K
G20	pT19/K20ac/K23me	K	K	G	S	K	K	A	V	pT	Kac	A	Q	Kme	K	D	G	K	K	R	K
G21	pT19/K20ac/K23me2	K	K	G	S	K	K	A	V	pT	Kac	A	Q	Kme2	K	D	G	K	K	R	K
G22	pT19/K20ac/K23me3	K	K	G	S	K	K	A	V	pT	Kac	A	Q	Kme3	K	D	G	K	K	R	K
G23	pT19/K20ac/K23ac	K	K	G	S	K	K	A	V	pT	Kac	A	Q	Kac	K	D	G	K	K	R	K
G24	K20me/K24me	K	K	G	S	K	K	A	V	T	Kme	A	Q	K	Kme	D	G	K	K	R	K
G25	K20me/K24me2	K	K	G	S	K	K	A	V	T	Kme	A	Q	K	Kme2	D	G	K	K	R	K
G26	K20me/K24me3	K	K	G	S	K	K	A	V	T	Kme	A	Q	K	Kme3	D	G	K	K	R	K
G27	K20me/K24ac	K	K	G	S	K	K	A	V	T	Kme	A	Q	K	Kac	D	G	K	K	R	K
G28	K20me2/K24me	K	K	G	S	K	K	A	V	T	Kme2	A	Q	K	Kme	D	G	K	K	R	K
G29	K20me2/K24me2	K	K	G	S	K	K	A	V	T	Kme2	A	Q	K	Kme2	D	G	K	K	R	K
G30	K20me2/K24me3	K	K	G	S	K	K	A	V	T	Kme2	A	Q	K	Kme3	D	G	K	K	R	K
G31	K20me2/K24ac	K	K	G	S	K	K	A	V	T	Kme2	A	Q	K	Kac	D	G	K	K	R	K
G32	K20me3/K24me	K	K	G	S	K	K	A	V	T	Kme3	A	Q	K	Kme	D	G	K	K	R	K
G33	K20me3/K24me2	K	K	G	S	K	K	A	V	T	Kme3	A	Q	K	Kme2	D	G	K	K	R	K
G34	K20me3/K24me3	K	K	G	S	K	K	A	V	T	Kme3	A	Q	K	Kme3	D	G	K	K	R	K
G35	K20me3/K24ac	K	K	G	S	K	K	A	V	T	Kme3	A	Q	K	Kac	D	G	K	K	R	K
G36	K20ac/K24me	K	K	G	S	K	K	A	V	T	Kac	A	Q	K	Kme	D	G	K	K	R	K
G37	K20ac/K24me2	K	K	G	S	K	K	A	V	T	Kac	A	Q	K	Kme2	D	G	K	K	R	K
H1	K20ac/K24me3	K	K	G	S	K	K	A	V	T	Kac	A	Q	K	Kme3	D	G	K	K	R	K
H2	K20ac/K24ac	K	K	G	S	K	K	A	V	T	Kac	A	Q	K	Kac	D	G	K	K	R	K
H3	pT19/K20me/K24me	K	K	G	S	K	K	A	V	pT	Kme	A	Q	K	Kme	D	G	K	K	R	K
H4	pT19/K20me/K24me2	K	K	G	S	K	K	A	V	pT	Kme	A	Q	K	Kme2	D	G	K	K	R	K
H5	pT19/K20me/K24me3	K	K	G	S	K	K	A	V	pT	Kme	A	Q	K	Kme3	D	G	K	K	R	K
H6	pT19/K20me/K24ac	K	K	G	S	K	K	A	V	pT	Kme	A	Q	K	Kac	D	G	K	K	R	K
H7	pT19/K20me2/K24me	K	K	G	S	K	K	A	V	pT	Kme2	A	Q	K	Kme	D	G	K	K	R	K
H8	pT19/K20me2/K24me2	K	K	G	S	K	K	A	V	pT	Kme2	A	Q	K	Kme2	D	G	K	K	R	K
H9	pT19/K20me2/K24me3	K	K	G	S	K	K	A	V	pT	Kme2	A	Q	K	Kme3	D	G	K	K	R	K
H10	pT19/K20me2/K24ac	K	K	G	S	K	K	A	V	pT	Kme2	A	Q	K	Kac	D	G	K	K	R	K
H11	pT19/K20me3/K24me	K	K	G	S	K	K	A	V	pT	Kme3	A	Q	K	Kme	D	G	K	K	R	K
H12	pT19/K20me3/K24me2	K	K	G	S	K	K	A	V	pT	Kme3	A	Q	K	Kme2	D	G	K	K	R	K
H13	pT19/K20me3/K24me3	K	K	G	S	K	K	A	V	pT	Kme3	A	Q	K	Kme3	D	G	K	K	R	K
H14	pT19/K20me3/K24ac	K	K	G	S	K	K	A	V	pT	Kme3	A	Q	K	Kac	D	G	K	K	R	K
H15	pT19/K20ac/K24me	K	K	G	S	K	K	A	V	pT	Kac	A	Q	K	Kme	D	G	K	K	R	K
H16	pT19/K20ac/K24me2	K	K	G	S	K	K	A	V	pT	Kac	A	Q	K	Kme2	D	G	K	K	R	K
H17	pT19/K20ac/K24me3	K	K	G	S	K	K	A	V	pT	Kac	A	Q	K	Kme3	D	G	K	K	R	K
H18	pT19/K20ac/K24ac	K	K	G	S	K	K	A	V	pT	Kac	A	Q	K	Kac	D	G	K	K	R	K
H19																					
H20	CONTROL	K	A	V	T	K	A	Q	K	K	D	G	K	K	R	K	R	S	R	K	E
H21	K23me	K	A	V	T	K	A	Q	Kme	K	D	G	K	K	R	K	R	S	R	K	E
H22	K23me2	K	A	V	T	K	A	Q	Kme2	K	D	G	K	K	R	K	R	S	R	K	E
H23	K23me3	K	A	V	T	K	A	Q	Kme3	K	D	G	K	K	R	K	R	S	R	K	E
H24	K23ac	K	A	V	T	K	A	Q	Kac	K	D	G	K	K	R	K	R	S	R	K	E
H25	K24me	K	A	V	T	K	A	Q	K	Kme	D	G	K	K	R	K	R	S	R	K	E
H26	K24me2	K	A	V	T	K	A	Q	K	Kme2	D	G	K	K	R	K	R	S	R	K	E
H27	K24me	K	A	V	T	K	A	Q	K	Kme3	D	G	K	K	R	K	R	S	R	K	E
H28	K24ac	K	A	V	T	K	A	Q	K	Kac	D	G	K	K	R	K	R	S	R	K	E
H29	K27me	K	A	V	T	K	A	Q	K	K	D	G	Kme	K	R	K	R	S	R	K	E
H30	K27me2	K	A	V	T	K	A	Q	K	K	D	G	Kme2	K	R	K	R	S	R	K	E
H31	K27me3	K	A	V	T	K	A	Q	K	K	D	G	Kme3	K	R	K	R	S	R	K	E
H32	K27ac	K	A	V	T	K	A	Q	K	K	D	G	Kac	K	R	K	R	S	R	K	E
H33	K28me	K	A	V	T	K	A	Q	K	K	D	G	K	Kme	R	K	R	S	R	K	E
H34	K28me2	K	A	V	T	K	A	Q	K	K	D	G	K	Kme2	R	K	R	S	R	K	E
H35	K28me3	K	A	V	T	K	A	Q	K	K	D	G	K	Kme3	R	K	R	S	R	K	E
H36	K28ac	K	A	V	T	K	A	Q	K	K	D	G	K	Kac	R	K	R	S	R	K	E
H37	K23me/K24me	K	A	V	T	K	A	Q	Kme	Kme	D	G	K	K	R	K	R	S	R	K	E
I1	K23me/K24me2	K	A	V	T	K	A	Q	Kme	Kme2	D	G	K	K	R	K	R	S	R	K	E
I2	K23me/K24me3	K	A	V	T	K	A	Q	Kme	Kme3	D	G	K	K	R	K	R	S	R	K	E
I3	K23me/K24ac	K	A	V	T	K	A	Q	Kme	Kac	D	G	K	K	R	K	R	S	R	K	E
I4	K23me2/K24me	K	A	V	T	K	A	Q	Kme2	Kme	D	G	K	K	R	K	R	S	R	K	E
I5	K23me2/K24me2	K	A	V	T	K	A	Q	Kme2	Kme2	D	G	K	K	R	K	R	S	R	K	E
I6	K23me2/K24me3	K	A	V	T	K	A	Q	Kme2	Kme3	D	G	K	K	R	K	R	S	R	K	E
I7	K23me2/K24ac	K	A	V	T	K	A	Q	Kme2	Kac	D	G	K	K	R	K	R	S	R	K	E
I8	K23me3/K24me	K	A	V	T	K	A	Q	Kme3	Kme	D	G	K	K	R	K	R	S	R	K	E
I9	K23me3/K24me2	K	A	V	T	K	A	Q	Kme3	Kme2	D	G	K	K	R	K	R	S	R	K	E
I10	K23me3/K24me3	K	A	V	T	K	A	Q	Kme3	Kme3	D	G	K	K	R	K	R	S	R	K	E
I11	K23me3/K24ac	K	A	V	T	K	A	Q	Kme3	Kac	D	G	K	K	R	K	R	S	R	K	E
I12	K23ac/K24me	K	A	V	T	K	A	Q	Kac	Kme	D	G	K	K	R	K	R	S	R	K	E
I13	K23ac/K24me2	K	A	V	T	K	A	Q	Kac	Kme2	D	G	K	K	R	K	R	S	R	K	E
I14	K23ac/K24me3	K	A	V	T	K	A	Q	Kac	Kme3	D	G	K	K	R	K	R	S	R	K	E
I15	K23ac/K24ac	K	A	V	T	K	A	Q	Kac	Kac	D	G	K	K	R	K	R	S	R	K	E
I16	K23me/K27me	K	A	V	T	K	A	Q	Kme	K	D	G	Kme	K	R	K	R	S	R	K	E
I17	K23me/K27me2	K	A	V	T	K	A	Q	Kme	K	D	G	Kme2	K	R	K	R	S	R	K	E
I18	K23me/K27me3	K	A	V	T	K	A	Q	Kme	K	D	G	Kme3	K	R	K	R	S	R	K	E
I19	K23me/K27ac	K	A	V	T	K	A	Q	Kme	K	D	G	Kac	K	R	K	R	S	R	K	E
I20	K23me2/K27me	K	A	V	T	K	A	Q	Kme2	K	D	G	Kme	K	R	K	R	S	R	K	E
I21	K23me2/K27me2	K	A	V	T	K	A	Q	Kme2	K	D	G	Kme2	K	R	K	R	S	R	K	E
I22	K23me2/K27me3	K	A	V	T	K	A	Q	Kme2	K	D	G	Kme3	K	R	K	R	S	R	K	E
I23	K23me2/K27ac	K	A	V	T	K	A	Q	Kme2	K	D	G	Kac	K	R	K	R	S	R	K	E
I24	K23me3/K27me	K	A	V	T	K	A	Q	Kme3	K	D	G	Kme	K	R	K	R	S	R	K	E
I25	K23me3/K27me2	K	A	V	T	K	A	Q	Kme3	K	D	G	Kme2	K	R	K	R	S	R	K	E
I26	K23me3/K27me3	K	A	V	T	K	A	Q	Kme3	K	D	G	Kme3	K	R	K	R	S	R	K	E
I27	K23me3/K27ac	K	A	V	T	K	A	Q	Kme3	K	D	G	Kac	K	R	K	R	S	R	K	E
I28	K23ac/K27me	K	A	V	T	K	A	Q	Kac	K	D	G	Kme	K	R	K	R	S	R	K	E
I29	K23ac/K27me2	K	A	V	T	K	A	Q	Kac	K	D	G	Kme2	K	R	K	R	S	R	K	E
I30	K23ac/K27me3	K	A	V	T	K	A	Q	Kac	K	D	G	Kme3	K	R	K	R	S	R	K	E
I31	K23ac/K27ac	K	A	V	T	K	A	Q	Kac	K	D	G	Kac	K	R	K	R	S	R	K	E
I32	K23me/K28me	K	A	V	T	K	A	Q	Kme	K	D	G	K	Kme	R	K	R	S	R	K	E
I33	K23me/K28me2	K	A	V	T	K	A	Q	Kme	K	D	G	K	Kme2	R	K	R	S	R	K	E
I34	K23me/K28me3	K	A	V	T	K	A	Q	Kme	K	D	G	K	Kme3	R	K	R	S	R	K	E
I35	K23me/K28ac	K	A	V	T	K	A	Q	Kme	K	D	G	K	Kac	R	K	R	S	R	K	E
I36	K23me2/K28me	K	A	V	T	K	A	Q	Kme2	K	D	G	K	Kme	R	K	R	S	R	K	E
I37	K23me2/K28me2	K	A	V	T	K	A	Q	Kme2	K	D	G	K	Kme2	R	K	R	S	R	K	E
J1	K23me2/K28me3	K	A	V	T	K	A	Q	Kme2	K	D	G	K	Kme3	R	K	R	S	R	K	E
J2	K23me2/K28ac	K	A	V	T	K	A	Q	Kme2	K	D	G	K	Kac	R	K	R	S	R	K	E
J3	K23me3/K28me	K	A	V	T	K	A	Q	Kme3	K	D	G	K	Kme	R	K	R	S	R	K	E
J4	K23me3/K28me2	K	A	V	T	K	A	Q	Kme3	K	D	G	K	Kme2	R	K	R	S	R	K	E
J5	K23me3/K28me3	K	A	V	T	K	A	Q	Kme3	K	D	G	K	Kme3	R	K	R	S	R	K	E
J6	K23me3/K28ac	K	A	V	T	K	A	Q	Kme3	K	D	G	K	Kac	R	K	R	S	R	K	E
J7	K23ac/K28me	K	A	V	T	K	A	Q	Kac	K	D	G	K	Kme	R	K	R	S	R	K	E
J8	K23ac/K28me2	K	A	V	T	K	A	Q	Kac	K	D	G	K	Kme2	R	K	R	S	R	K	E
J9	K23ac/K28me3	K	A	V	T	K	A	Q	Kac	K	D	G	K	Kme3	R	K	R	S	R	K	E

J10	K23ac/K28ac	K	A	V	T	K	A	Q	Kac	K	D	G	K	Kac	R	K	R	S	R	K	E
J11	K23ac/K24ac/K27ac	K	A	V	T	K	A	Q	Kac	Kac	D	G	Kac	K	R	K	R	S	R	K	E
J12	K23ac/K27ac/K28ac	K	A	V	T	K	A	Q	Kac	K	D	G	Kac	Kac	R	K	R	S	R	K	E
J13	K23ac/K24ac/K28ac	K	A	V	T	K	A	Q	Kac	Kac	D	G	K	Kac	R	K	R	S	R	K	E
J14	K23ac/K24ac/K27ac/K28ac	K	A	V	T	K	A	Q	Kac	Kac	D	G	Kac	Kac	R	K	R	S	R	K	E
J15																					
J16	CONTROL	A	Q	K	K	D	G	K	K	R	K	R	S	R	K	E	S	Y	S	V	Y
J17	K27me	A	Q	K	K	D	G	Kme	K	R	K	R	S	R	K	E	S	Y	S	V	Y
J18	K27me2	A	Q	K	K	D	G	Kme2	K	R	K	R	S	R	K	E	S	Y	S	V	Y
J19	K27me3	A	Q	K	K	D	G	Kme3	K	R	K	R	S	R	K	E	S	Y	S	V	Y
J20	K27ac	A	Q	K	K	D	G	Kac	K	R	K	R	S	R	K	E	S	Y	S	V	Y
J21	K28me	A	Q	K	K	D	G	K	Kme	R	K	R	S	R	K	E	S	Y	S	V	Y
J22	K28me2	A	Q	K	K	D	G	K	Kme2	R	K	R	S	R	K	E	S	Y	S	V	Y
J23	K28me3	A	Q	K	K	D	G	K	Kme3	R	K	R	S	R	K	E	S	Y	S	V	Y
J24	K28ac	A	Q	K	K	D	G	K	Kac	R	K	R	S	R	K	E	S	Y	S	V	Y
J25	K30me	A	Q	K	K	D	G	K	K	R	Kme	R	S	R	K	E	S	Y	S	V	Y
J26	K30me2	A	Q	K	K	D	G	K	K	R	Kme2	R	S	R	K	E	S	Y	S	V	Y
J27	K30me3	A	Q	K	K	D	G	K	K	R	Kme3	R	S	R	K	E	S	Y	S	V	Y
J28	K30ac	A	Q	K	K	D	G	K	K	R	Kac	R	S	R	K	E	S	Y	S	V	Y
J29	pS32	A	Q	K	K	D	G	K	K	R	K	R	pS	R	K	E	S	Y	S	V	Y
J30	K27me/K28me	A	Q	K	K	D	G	Kme	Kme	R	K	R	S	R	K	E	S	Y	S	V	Y
J31	K27me/K28me2	A	Q	K	K	D	G	Kme	Kme2	R	K	R	S	R	K	E	S	Y	S	V	Y
J32	K27me/K28me3	A	Q	K	K	D	G	Kme	Kme3	R	K	R	S	R	K	E	S	Y	S	V	Y
J33	K27me/K28ac	A	Q	K	K	D	G	Kme	Kac	R	K	R	S	R	K	E	S	Y	S	V	Y
J34	K27me2/K28me	A	Q	K	K	D	G	Kme2	Kme	R	K	R	S	R	K	E	S	Y	S	V	Y
J35	K27me2/K28me2	A	Q	K	K	D	G	Kme2	Kme2	R	K	R	S	R	K	E	S	Y	S	V	Y
J36	K27me2/K28me3	A	Q	K	K	D	G	Kme2	Kme3	R	K	R	S	R	K	E	S	Y	S	V	Y
J37	K27me2/K28ac	A	Q	K	K	D	G	Kme2	Kac	R	K	R	S	R	K	E	S	Y	S	V	Y
K1	K27me3/K28me	A	Q	K	K	D	G	Kme3	Kme	R	K	R	S	R	K	E	S	Y	S	V	Y
K2	K27me3/K28me2	A	Q	K	K	D	G	Kme3	Kme2	R	K	R	S	R	K	E	S	Y	S	V	Y
K3	K27me3/K28me3	A	Q	K	K	D	G	Kme3	Kme3	R	K	R	S	R	K	E	S	Y	S	V	Y
K4	K27me3/K28ac	A	Q	K	K	D	G	Kme3	Kac	R	K	R	S	R	K	E	S	Y	S	V	Y
K5	K27ac/K28me	A	Q	K	K	D	G	Kac	Kme	R	K	R	S	R	K	E	S	Y	S	V	Y
K6	K27ac/K28me2	A	Q	K	K	D	G	Kac	Kme2	R	K	R	S	R	K	E	S	Y	S	V	Y
K7	K27ac/K28me3	A	Q	K	K	D	G	Kac	Kme3	R	K	R	S	R	K	E	S	Y	S	V	Y
K8	K27ac/K28ac	A	Q	K	K	D	G	Kac	Kac	R	K	R	S	R	K	E	S	Y	S	V	Y
K9	K27me/K30me	A	Q	K	K	D	G	Kme	K	R	Kme	R	S	R	K	E	S	Y	S	V	Y
K10	K27me/K30me2	A	Q	K	K	D	G	Kme	K	R	Kme2	R	S	R	K	E	S	Y	S	V	Y
K11	K27me/K30me3	A	Q	K	K	D	G	Kme	K	R	Kme3	R	S	R	K	E	S	Y	S	V	Y
K12	K27me/K30ac	A	Q	K	K	D	G	Kme	K	R	Kac	R	S	R	K	E	S	Y	S	V	Y
K13	K27me2/K30me	A	Q	K	K	D	G	Kme2	K	R	Kme	R	S	R	K	E	S	Y	S	V	Y
K14	K27me2/K30me2	A	Q	K	K	D	G	Kme2	K	R	Kme2	R	S	R	K	E	S	Y	S	V	Y
K15	K27me2/K30me3	A	Q	K	K	D	G	Kme2	K	R	Kme3	R	S	R	K	E	S	Y	S	V	Y
K16	K27me2/K30ac	A	Q	K	K	D	G	Kme2	K	R	Kac	R	S	R	K	E	S	Y	S	V	Y
K17	K27me3/K30me	A	Q	K	K	D	G	Kme3	K	R	Kme	R	S	R	K	E	S	Y	S	V	Y
K18	K27me3/K30me2	A	Q	K	K	D	G	Kme3	K	R	Kme2	R	S	R	K	E	S	Y	S	V	Y
K19	K27me3/K30me3	A	Q	K	K	D	G	Kme3	K	R	Kme3	R	S	R	K	E	S	Y	S	V	Y
K20	K27me3/K30ac	A	Q	K	K	D	G	Kme3	K	R	Kac	R	S	R	K	E	S	Y	S	V	Y
K21	K27ac/K30me	A	Q	K	K	D	G	Kac	K	R	Kme	R	S	R	K	E	S	Y	S	V	Y
K22	K27ac/K30me2	A	Q	K	K	D	G	Kac	K	R	Kme2	R	S	R	K	E	S	Y	S	V	Y
K23	K27ac/K30me3	A	Q	K	K	D	G	Kac	K	R	Kme3	R	S	R	K	E	S	Y	S	V	Y
K24	K27ac/K30ac	A	Q	K	K	D	G	Kac	K	R	Kac	R	S	R	K	E	S	Y	S	V	Y
K25	K28me/K30me	A	Q	K	K	D	G	K	Kme	R	Kme	R	S	R	K	E	S	Y	S	V	Y
K26	K28me/K30me2	A	Q	K	K	D	G	K	Kme	R	Kme2	R	S	R	K	E	S	Y	S	V	Y
K27	K28me/K30me3	A	Q	K	K	D	G	K	Kme	R	Kme3	R	S	R	K	E	S	Y	S	V	Y
K28	K28me/K30ac	A	Q	K	K	D	G	K	Kme	R	Kac	R	S	R	K	E	S	Y	S	V	Y
K29	K28me2/K30me	A	Q	K	K	D	G	K	Kme2	R	Kme	R	S	R	K	E	S	Y	S	V	Y
K30	K28me2/K30me2	A	Q	K	K	D	G	K	Kme2	R	Kme2	R	S	R	K	E	S	Y	S	V	Y
K31	K28me2/K30me3	A	Q	K	K	D	G	K	Kme2	R	Kme3	R	S	R	K	E	S	Y	S	V	Y
K32	K28me2/K30ac	A	Q	K	K	D	G	K	Kme2	R	Kac	R	S	R	K	E	S	Y	S	V	Y
K33	K28me3/K30me	A	Q	K	K	D	G	K	Kme3	R	Kme	R	S	R	K	E	S	Y	S	V	Y
K34	K28me3/K30me2	A	Q	K	K	D	G	K	Kme3	R	Kme2	R	S	R	K	E	S	Y	S	V	Y
K35	K28me3/K30me3	A	Q	K	K	D	G	K	Kme3	R	Kme3	R	S	R	K	E	S	Y	S	V	Y
K36	K28me3/K30ac	A	Q	K	K	D	G	K	Kme3	R	Kac	R	S	R	K	E	S	Y	S	V	Y
K37	K28ac/K30me	A	Q	K	K	D	G	K	Kac	R	Kme	R	S	R	K	E	S	Y	S	V	Y
L1	K28ac/K30me2	A	Q	K	K	D	G	K	Kac	R	Kme2	R	S	R	K	E	S	Y	S	V	Y
L2	K28ac/K30me3	A	Q	K	K	D	G	K	Kac	R	Kme3	R	S	R	K	E	S	Y	S	V	Y
L3	K28ac/K30ac	A	Q	K	K	D	G	K	Kac	R	Kac	R	S	R	K	E	S	Y	S	V	Y
L4	K27me/pS32	A	Q	K	K	D	G	Kme	K	R	K	R	pS	R	K	E	S	Y	S	V	Y
L5	K27me2/pS32	A	Q	K	K	D	G	Kme2	K	R	K	R	pS	R	K	E	S	Y	S	V	Y
L6	K27me3/pS32	A	Q	K	K	D	G	Kme3	K	R	K	R	pS	R	K	E	S	Y	S	V	Y
L7	K27ac/pS32	A	Q	K	K	D	G	Kac	K	R	K	R	pS	R	K	E	S	Y	S	V	Y
L8	K28me/pS32	A	Q	K	K	D	G	K	Kme	R	K	R	pS	R	K	E	S	Y	S	V	Y
L9	K28me2/pS32	A	Q	K	K	D	G	K	Kme2	R	K	R	pS	R	K	E	S	Y	S	V	Y
L10	K28me3/pS32	A	Q	K	K	D	G	K	Kme3	R	K	R	pS	R	K	E	S	Y	S	V	Y
L11	K28ac/pS32	A	Q	K	K	D	G	K	Kac	R	K	R	pS	R	K	E	S	Y	S	V	Y
L12	K30me/pS32	A	Q	K	K	D	G	K	K	R	Kme	R	pS	R	K	E	S	Y	S	V	Y
L13	K30me2/pS32	A	Q	K	K	D	G	K	K	R	Kme2	R	pS	R	K	E	S	Y	S	V	Y
L14	K30me3/pS32	A	Q	K	K	D	G	K	K	R	Kme3	R	pS	R	K	E	S	Y	S	V	Y
L15	K30ac/pS32	A	Q	K	K	D	G	K	K	R	Kac	R	pS	R	K	E	S	Y	S	V	Y
L16	K27ac/K30ac	A	Q	K	K	D	G	Kac	K	R	Kac	R	S	R	K	E	S	Y	S	V	Y
L17	K27ac/K28ac	A	Q	K	K	D	G	Kac	Kac	R	K	R	S	R	K	E	S	Y	S	V	Y
L18	K27ac/K28ac/K30ac	A	Q	K	K	D	G	Kac	Kac	R	Kac	R	S	R	K	E	S	Y	S	V	Y
L19	K27me/K30me/pS32	A	Q	K	K	D	G	Kme	K	R	Kme	R	pS	R	K	E	S	Y	S	V	Y
L20	K27me/K30me2/pS32	A	Q	K	K	D	G	Kme	K	R	Kme2	R	pS	R	K	E	S	Y	S	V	Y
L21	K27me/K30me3/pS32	A	Q	K	K	D	G	Kme	K	R	Kme3	R	pS	R	K	E	S	Y	S	V	Y
L22	K27me/K30ac/pS32	A	Q	K	K	D	G	Kme	K	R	Kac	R	pS	R	K	E	S	Y	S	V	Y
L23	K27me2/K30me/pS32	A	Q	K	K	D	G	Kme2	K	R	Kme	R	pS	R	K	E	S	Y	S	V	Y
L24	K27me2/K30me2/pS32	A	Q	K	K	D	G	Kme2	K	R	Kme2	R	pS	R	K	E	S	Y	S	V	Y
L25	K27me2/K30me3/pS32	A	Q	K	K	D	G	Kme2	K	R	Kme3	R	pS	R	K	E	S	Y	S	V	Y
L26	K27me2/K30ac/pS32	A	Q	K	K	D	G	Kme2	K	R	Kac	R	pS	R	K	E	S	Y	S	V	Y
L27	K27me3/K30me/pS32	A	Q	K	K	D	G	Kme3	K	R	Kme	R	pS	R	K	E	S	Y	S	V	Y
L28	K27me3/K30me2/pS32	A	Q	K	K	D	G	Kme3	K	R	Kme2	R	pS	R	K	E	S	Y	S	V	Y
L29	K27me3/K30me3/pS32	A	Q	K	K	D	G	Kme3	K	R	Kme3	R	pS	R	K	E	S	Y	S	V	Y
L30	K27me3/K30ac/pS32	A	Q	K	K	D	G	Kme3	K	R	Kac	R	pS	R	K	E	S	Y	S	V	Y
L31	K27ac/K30me/pS32	A	Q	K	K	D	G	Kac	K	R	Kme	R	pS	R	K	E	S	Y	S	V	Y
L32	K27ac/K30me2/pS32	A	Q	K	K	D	G	Kac	K	R	Kme2	R	pS	R	K	E	S	Y	S	V	Y
L33	K27ac/K30me3/pS32	A	Q	K	K	D	G	Kac	K	R	Kme3	R	pS	R	K	E	S	Y	S	V	Y
L34	K27ac/K30ac/pS32	A	Q	K	K	D	G	Kac	K	R	Kac	R	pS	R	K	E	S	Y	S	V	Y
L35	K28me/K30me/pS32	A	Q	K	K	D	G	K	Kme	R	Kme	R	pS	R	K	E	S	Y	S	V	Y
L36	K28me/K30me2/pS32	A	Q	K	K	D	G	K	Kme	R	Kme2	R	pS	R	K	E	S	Y	S	V	Y
L37	K28me/K30me3/pS32	A	Q	K	K	D	G	K	Kme	R	Kme3	R	pS	R	K	E	S	Y	S	V	Y
M1	K28me/K30ac/pS32	A	Q	K	K	D	G	K	Kme	R	Kac	R	pS	R	K	E	S	Y	S	V	Y
M2	K28me2/K30me/pS32	A	Q	K	K	D	G	K	Kme2	R	Kme	R	pS	R	K	E	S	Y	S	V	Y
M3	K28me2/K30me2/pS32	A	Q	K	K	D	G	K	Kme2	R	Kme2	R	pS	R	K	E	S	Y	S	V	Y
M4	K28me2/K30me3/pS32	A	Q	K	K	D	G	K	Kme2	R	Kme3	R	pS	R	K	E	S	Y	S	V	Y
M5	K28me2/K30ac/pS32	A	Q	K	K	D	G	K	Kme2	R	Kac	R	pS	R	K	E	S	Y	S	V	Y
M6	K28me3/K30me/pS32	A	Q	K	K	D	G	K	Kme3	R	Kme	R	pS	R	K	E	S	Y	S	V	Y
M7	K28me3/K30me2/pS32	A	Q	K	K	D	G	K	Kme3	R	Kme2	R	pS	R	K	E	S	Y	S	V	Y
M8	K28me3/K30me3/pS32	A	Q	K	K	D	G	K	Kme3	R	Kme3	R	pS	R	K	E	S	Y	S	V	Y
M9	K28me3/K30ac/pS32	A	Q	K	K	D	G	K	Kme3	R	Kac	R	pS	R	K	E	S	Y	S	V	Y
M10	K28ac/K30me/pS32	A	Q	K	K	D	G	K	Kac	R	Kme	R	pS	R	K	E	S	Y	S	V	Y
M11	K28ac/K30me2/pS32	A	Q	K	K	D	G	K	Kac	R	Kme2	R	pS	R	K	E	S	Y	S	V	Y
M12	K28ac/K30me3/pS32	A	Q	K	K	D	G	K	Kac	R	Kme3	R	pS	R	K	E	S	Y	S	V	Y
M13	K28ac/K30ac/pS32	A	Q	K	K	D	G	K	Kac	R	Kac	R	pS	R	K	E	S	Y	S	V	Y

**M14**																					
**M15**	CONTROL	G	**K**	**K**	R	**K**	R	**S**	R	**K**	E	**S**	Y	**S**	V	Y	V	Y	**K**	V	L
**M16**	pS32	G	K	K	R	K	R	**pS**	R	K	E	S	Y	S	V	Y	V	Y	K	V	L
**M17**	K34me	G	K	K	R	K	R	S	R	**Kme**	E	S	Y	S	V	Y	V	Y	K	V	L
**M18**	K34me2	G	K	K	R	K	R	S	R	**Kme2**	E	S	Y	S	V	Y	V	Y	K	V	L
**M19**	K34me3	G	K	K	R	K	R	S	R	**Kme3**	E	S	Y	S	V	Y	V	Y	K	V	L
**M20**	K34ac	G	K	K	R	K	R	S	R	**Kac**	E	S	Y	S	V	Y	V	Y	K	V	L
**M21**	pS36	G	K	K	R	K	R	S	R	K	E	**pS**	Y	S	V	Y	V	Y	K	V	L
**M22**	pS38	G	K	K	R	K	R	S	R	K	E	S	Y	**pS**	V	Y	V	Y	K	V	L
**M23**	pS32/K34me	G	K	K	R	K	R	**pS**	R	**Kme**	E	S	Y	S	V	Y	V	Y	K	V	L
**M24**	pS32/K34me2	G	K	K	R	K	R	**pS**	R	**Kme2**	E	S	Y	S	V	Y	V	Y	K	V	L
**M25**	pS32/K34me3	G	K	K	R	K	R	**pS**	R	**Kme3**	E	S	Y	S	V	Y	V	Y	K	V	L
**M26**	pS32/K34ac	G	K	K	R	K	R	**pS**	R	**Kac**	E	S	Y	S	V	Y	V	Y	K	V	L
**M27**	K34me/pS36	G	K	K	R	K	R	S	R	**Kme**	E	**pS**	Y	S	V	Y	V	Y	K	V	L
**M28**	K34me2/pS36	G	K	K	R	K	R	S	R	**Kme2**	E	**pS**	Y	S	V	Y	V	Y	K	V	L
**M29**	K34me3/pS36	G	K	K	R	K	R	S	R	**Kme3**	E	**pS**	Y	S	V	Y	V	Y	K	V	L
**M30**	K34ac/pS36	G	K	K	R	K	R	S	R	**Kac**	E	**pS**	Y	S	V	Y	V	Y	K	V	L
**M31**	K34me/pS38	G	K	K	R	K	R	S	R	**Kme**	E	S	Y	**pS**	V	Y	V	Y	K	V	L
**M32**	K34me2/pS38	G	K	K	R	K	R	S	R	**Kme2**	E	S	Y	**pS**	V	Y	V	Y	K	V	L
**M33**	K34me3/pS38	G	K	K	R	K	R	S	R	**Kme3**	E	S	Y	**pS**	V	Y	V	Y	K	V	L
**M34**	K34ac/pS38	G	K	K	R	K	R	S	R	**Kac**	E	S	Y	**pS**	V	Y	V	Y	K	V	L

Histone	Peptide Length	Array Poition	Kac Mark Combinations	Sequence																			
polyHis CTRL		A1	HHHHHHHHHH																				
polyHis CTRL		A2	HHHHHHHHHH																				
		A3	(blank)																				
							4					9					14				18		
H3.1 (P68431)	1-20	A4	CONTROL	A	R	T	K	Q	T	A	R	K	S	T	G	G	K	A	P	R	K	Q	L
		A5	K4me	A	R	T	Kme	Q	T	A	R	K	S	T	G	G	K	A	P	R	K	Q	L
		A6	K4me2	A	R	T	Kme2	Q	T	A	R	K	S	T	G	G	K	A	P	R	K	Q	L
		A7	K4me3	A	R	T	Kme3	Q	T	A	R	K	S	T	G	G	K	A	P	R	K	Q	L
		A8	K4ac	A	R	T	Kac	Q	T	A	R	K	S	T	G	G	K	A	P	R	K	Q	L
		A9	pT3	A	R	pT	K	Q	T	A	R	K	S	T	G	G	K	A	P	R	K	Q	L
		A10	pT3/K4ac	A	R	pT	Kme	Q	T	A	R	K	S	T	G	G	K	A	P	R	K	Q	L
		A11	pT3/K4me	A	R	pT	Kme2	Q	T	A	R	K	S	T	G	G	K	A	P	R	K	Q	L
		A12	pT3/K4me2	A	R	pT	Kme3	Q	T	A	R	K	S	T	G	G	K	A	P	R	K	Q	L
		A13	pT3/K4me3	A	R	pT	Kac	Q	T	A	R	K	S	T	G	G	K	A	P	R	K	Q	L
		A14	pT6	A	R	T	K	Q	pT	A	R	K	S	T	G	G	K	A	P	R	K	Q	L
		A15	K4me/pT6	A	R	T	Kme	Q	pT	A	R	K	S	T	G	G	K	A	P	R	K	Q	L
		A16	K4me2/pT6	A	R	T	Kme2	Q	pT	A	R	K	S	T	G	G	K	A	P	R	K	Q	L
		A17	K4me3/pT6	A	R	T	Kme3	Q	pT	A	R	K	S	T	G	G	K	A	P	R	K	Q	L
		A18	K4ac/pT6	A	R	T	Kac	Q	pT	A	R	K	S	T	G	G	K	A	P	R	K	Q	L
		B1	K9me	A	R	T	K	Q	T	A	R	Kme	S	T	G	G	K	A	P	R	K	Q	L
		B2	K9me2	A	R	T	K	Q	T	A	R	Kme2	S	T	G	G	K	A	P	R	K	Q	L
		B3	K9me3	A	R	T	K	Q	T	A	R	Kme3	S	T	G	G	K	A	P	R	K	Q	L
		B4	K9ac	A	R	T	K	Q	T	A	R	Kac	S	T	G	G	K	A	P	R	K	Q	L
		B5	pT6/K9me	A	R	T	K	Q	pT	A	R	Kme	S	T	G	G	K	A	P	R	K	Q	L
		B6	pT6/K9me2	A	R	T	K	Q	pT	A	R	Kme2	S	T	G	G	K	A	P	R	K	Q	L
		B7	pT6/K9me3	A	R	T	K	Q	pT	A	R	Kme3	S	T	G	G	K	A	P	R	K	Q	L
		B8	pT6/K9ac	A	R	T	K	Q	pT	A	R	Kac	S	T	G	G	K	A	P	R	K	Q	L
		B9	pS10	A	R	T	K	Q	T	A	R	K	pS	T	G	G	K	A	P	R	K	Q	L
		B10	K9me/pS10	A	R	T	K	Q	T	A	R	Kme	pS	T	G	G	K	A	P	R	K	Q	L
		B11	K9me2/pS10	A	R	T	K	Q	T	A	R	Kme2	pS	T	G	G	K	A	P	R	K	Q	L
		B12	K9me3/pS10	A	R	T	K	Q	T	A	R	Kme3	pS	T	G	G	K	A	P	R	K	Q	L
		B13	K9ac/pS10	A	R	T	K	Q	T	A	R	Kac	pS	T	G	G	K	A	P	R	K	Q	L
		B14	pT11	A	R	T	K	Q	T	A	R	K	S	pT	G	G	K	A	P	R	K	Q	L
		B15	K9me/pT11	A	R	T	K	Q	T	A	R	Kme	S	pT	G	G	K	A	P	R	K	Q	L
		B16	K9me2/pT11	A	R	T	K	Q	T	A	R	Kme2	S	pT	G	G	K	A	P	R	K	Q	L
		B17	K9me3/pT11	A	R	T	K	Q	T	A	R	Kme3	S	pT	G	G	K	A	P	R	K	Q	L
		B18	K9ac/pT11	A	R	T	K	Q	T	A	R	Kac	S	pT	G	G	K	A	P	R	K	Q	L
		C1	K14me	A	R	T	K	Q	T	A	R	K	S	T	G	G	Kme	A	P	R	K	Q	L
		C2	K14me2	A	R	T	K	Q	T	A	R	K	S	T	G	G	Kme2	A	P	R	K	Q	L
		C3	K14me3	A	R	T	K	Q	T	A	R	K	S	T	G	G	Kme3	A	P	R	K	Q	L
		C4	K14ac	A	R	T	K	Q	T	A	R	K	S	T	G	G	Kac	A	P	R	K	Q	L
		C5	K9me/K14ac	A	R	T	K	Q	T	A	R	Kme	S	T	G	G	Kac	A	P	R	K	Q	L
		C6	K9me2/K14ac	A	R	T	K	Q	T	A	R	Kme2	S	T	G	G	Kac	A	P	R	K	Q	L
		C7	K9me3/K14ac	A	R	T	K	Q	T	A	R	Kme3	S	T	G	G	Kac	A	P	R	K	Q	L
		C8	K9ac/K14ac	A	R	T	K	Q	T	A	R	Kac	S	T	G	G	Kac	A	P	R	K	Q	L
		C9	pS10/K14ac	A	R	T	K	Q	T	A	R	K	pS	T	G	G	Kac	A	P	R	K	Q	L
		C10	pT11/K14ac	A	R	T	K	Q	T	A	R	K	S	pT	G	G	Kac	A	P	R	K	Q	L
		C11	K9ac/pS10/K14ac	A	R	T	K	Q	T	A	R	Kac	pS	T	G	G	Kac	A	P	R	K	Q	L
		C12	K9ac/pT11/K14ac	A	R	T	K	Q	T	A	R	Kac	S	pT	G	G	Kac	A	P	R	K	Q	L
							9					14				18					23		
H3.1 (P68431)	6-25	C13	CONTROL	T	A	R	K	S	T	G	G	K	A	P	R	K	Q	L	A	T	K	A	A
		C14	K14me	T	A	R	K	S	T	G	G	Kme	A	P	R	K	Q	L	A	T	K	A	A
		C15	K14me2	T	A	R	K	S	T	G	G	Kme2	A	P	R	K	Q	L	A	T	K	A	A
		C16	K14me3	T	A	R	K	S	T	G	G	Kme3	A	P	R	K	Q	L	A	T	K	A	A
		C17	K14ac	T	A	R	K	S	T	G	G	Kac	A	P	R	K	Q	L	A	T	K	A	A
		C18	K9me/K14ac	T	A	R	Kme	S	T	G	G	Kac	A	P	R	K	Q	L	A	T	K	A	A
		D1	K9me2/K14ac	T	A	R	Kme2	S	T	G	G	Kac	A	P	R	K	Q	L	A	T	K	A	A
		D2	K9me3/K14ac	T	A	R	Kme3	S	T	G	G	Kac	A	P	R	K	Q	L	A	T	K	A	A
		D3	K9ac/K14ac	T	A	R	Kac	S	T	G	G	Kac	A	P	R	K	Q	L	A	T	K	A	A
		D4	pS10/K14ac	T	A	R	K	pS	T	G	G	K	A	P	R	K	Q	L	A	T	K	A	A
		D5	pT11/K14ac	T	A	R	K	S	pT	G	G	K	A	P	R	K	Q	L	A	T	K	A	A
		D6	K9ac/pS10/K14ac	T	A	R	Kac	pS	T	G	G	Kac	A	P	R	K	Q	L	A	T	K	A	A
		D7	K9ac/pT11/K14ac	T	A	R	Kac	S	pT	G	G	Kac	A	P	R	K	Q	L	A	T	K	A	A
		D8	K18me	T	A	R	K	S	T	G	G	K	A	P	R	Kme	Q	L	A	T	K	A	A
		D9	K18me2	T	A	R	K	S	T	G	G	K	A	P	R	Kme2	Q	L	A	T	K	A	A
		D10	K18me3	T	A	R	K	S	T	G	G	K	A	P	R	Kme3	Q	L	A	T	K	A	A
		D11	K18ac	T	A	R	K	S	T	G	G	K	A	P	R	Kac	Q	L	A	T	K	A	A
		D12	K14ac/K18me	T	A	R	K	S	T	G	G	Kac	A	P	R	Kme	Q	L	A	T	K	A	A
		D13	K14ac/K18me2	T	A	R	K	S	T	G	G	Kac	A	P	R	Kme2	Q	L	A	T	K	A	A
		D14	K14ac/K18me3	T	A	R	K	S	T	G	G	Kac	A	P	R	Kme3	Q	L	A	T	K	A	A
		D15	K14ac/K18ac	T	A	R	K	S	T	G	G	Kac	A	P	R	Kac	Q	L	A	T	K	A	A
		D16	pS10/K14ac/K18me	T	A	R	K	pS	T	G	G	Kac	A	P	R	Kme	Q	L	A	T	K	A	A
		D17	pS10/K14ac/K18me2	T	A	R	K	pS	T	G	G	Kac	A	P	R	Kme2	Q	L	A	T	K	A	A
		D18	pS10/K14ac/K18me3	T	A	R	K	pS	T	G	G	Kac	A	P	R	Kme3	Q	L	A	T	K	A	A
		E1	pS10/K14ac/K18ac	T	A	R	K	pS	T	G	G	Kac	A	P	R	Kac	Q	L	A	T	K	A	A
		E2	pT11/K14ac/K18me	T	A	R	K	S	pT	G	G	Kac	A	P	R	Kme	Q	L	A	T	K	A	A
		E3	pT11/K14ac/K18me2	T	A	R	K	S	pT	G	G	Kac	A	P	R	Kme2	Q	L	A	T	K	A	A
		E4	pT11/K14ac/K18me3	T	A	R	K	S	pT	G	G	Kac	A	P	R	Kme3	Q	L	A	T	K	A	A
		E5	pT11/K14ac/K18ac	T	A	R	K	S	pT	G	G	Kac	A	P	R	Kac	Q	L	A	T	K	A	A
							14				18				22	23				27	28		
H3.1 (P68431)	11-30	E6	CONTROL	T	G	G	K	A	P	R	K	Q	L	A	T	K	A	A	R	K	S	A	P
		E7	K18me	T	G	G	K	A	P	R	Kme	Q	L	A	T	K	A	A	R	K	S	A	P
		E8	K18me2	T	G	G	K	A	P	R	Kme2	Q	L	A	T	K	A	A	R	K	S	A	P
		E9	K18me3	T	G	G	K	A	P	R	Kme3	Q	L	A	T	K	A	A	R	K	S	A	P
		E10	K18ac	T	G	G	K	A	P	R	Kac	Q	L	A	T	K	A	A	R	K	S	A	P
		E11	K14ac/K18me	T	G	G	Kac	A	P	R	Kme	Q	L	A	T	K	A	A	R	K	S	A	P
		E12	K14ac/K18me2	T	G	G	Kac	A	P	R	Kme2	Q	L	A	T	K	A	A	R	K	S	A	P
		E13	K14ac/K18me3	T	G	G	Kac	A	P	R	Kme3	Q	L	A	T	K	A	A	R	K	S	A	P
		E14	K14ac/K18ac	T	G	G	Kac	A	P	R	Kac	Q	L	A	T	K	A	A	R	K	S	A	P
		E15	pT22	T	G	G	K	A	P	R	K	Q	L	A	pT	K	A	A	R	K	S	A	P
		E16	pT22/K18me	T	G	G	K	A	P	R	Kme	Q	L	A	pT	K	A	A	R	K	S	A	P
		E17	pT22/K18me2	T	G	G	K	A	P	R	Kme2	Q	L	A	pT	K	A	A	R	K	S	A	P
		E18	pT22/K18me3	T	G	G	K	A	P	R	Kme3	Q	L	A	pT	K	A	A	R	K	S	A	P
		F1	pT22/K18ac	T	G	G	K	A	P	R	Kac	Q	L	A	pT	K	A	A	R	K	S	A	P
		F2	K23me	T	G	G	K	A	P	R	K	Q	L	A	T	Kme	A	A	R	K	S	A	P
		F3	K23me2	T	G	G	K	A	P	R	K	Q	L	A	T	Kme2	A	A	R	K	S	A	P
		F4	K23me3	T	G	G	K	A	P	R	K	Q	L	A	T	Kme3	A	A	R	K	S	A	P
		F5	K23ac	T	G	G	K	A	P	R	K	Q	L	A	T	Kac	A	A	R	K	S	A	P
		F6	pT22/K23me	T	G	G	K	A	P	R	K	Q	L	A	pT	Kme	A	A	R	K	S	A	P

		F7	pT22/K23me2	T	G	G	K	A	P	R	K	Q	L	A	pT	Kme2	A	A	R	K	S	A	P
		F8	pT22/K23me3	T	G	G	K	A	P	R	K	Q	L	A	pT	Kme3	A	A	R	K	S	A	P
		F9	pT22/K23ac	T	G	G	K	A	P	R	K	Q	L	A	pT	Kac	A	A	R	K	S	A	P
		F10	K18me/K23me	T	G	G	K	A	P	R	Kme	Q	L	A	T	Kme	A	A	R	K	S	A	P
		F11	K18me2/K23me	T	G	G	K	A	P	R	Kme2	Q	L	A	T	Kme	A	A	R	K	S	A	P
		F12	K18me3/K23me	T	G	G	K	A	P	R	Kme3	Q	L	A	T	Kme	A	A	R	K	S	A	P
		F13	K18ac/K23me	T	G	G	K	A	P	R	Kac	Q	L	A	T	Kme	A	A	R	K	S	A	P
		F14	K18me/K23me2	T	G	G	K	A	P	R	Kme	Q	L	A	T	Kme2	A	A	R	K	S	A	P
		F15	K18me2/K23me2	T	G	G	K	A	P	R	Kme2	Q	L	A	T	Kme2	A	A	R	K	S	A	P
		F16	K18me3/K23me2	T	G	G	K	A	P	R	Kme3	Q	L	A	T	Kme2	A	A	R	K	S	A	P
		F17	K18ac/K23me2	T	G	G	K	A	P	R	Kac	Q	L	A	T	Kme2	A	A	R	K	S	A	P
		F18	K18me/K23me3	T	G	G	K	A	P	R	Kme	Q	L	A	T	Kme3	A	A	R	K	S	A	P
		G1	K18me2/K23me3	T	G	G	K	A	P	R	Kme2	Q	L	A	T	Kme3	A	A	R	K	S	A	P
		G2	K18me3/K23me3	T	G	G	K	A	P	R	Kme3	Q	L	A	T	Kme3	A	A	R	K	S	A	P
		G3	K18ac/K23me3	T	G	G	K	A	P	R	Kac	Q	L	A	T	Kme3	A	A	R	K	S	A	P
		G4	K18me/K23ac	T	G	G	K	A	P	R	Kme	Q	L	A	T	Kac	A	A	R	K	S	A	P
		G5	K18me2/K23ac	T	G	G	K	A	P	R	Kme2	Q	L	A	T	Kac	A	A	R	K	S	A	P
		G6	K18me3/K23ac	T	G	G	K	A	P	R	Kme3	Q	L	A	T	Kac	A	A	R	K	S	A	P
		G7	K18ac/K23ac	T	G	G	K	A	P	R	Kac	Q	L	A	T	Kac	A	A	R	K	S	A	P
		G8	K18me/pT22/K23me	T	G	G	K	A	P	R	Kme	Q	L	A	pT	Kme	A	A	R	K	S	A	P
		G9	K18me2/pT22/K23me	T	G	G	K	A	P	R	Kme2	Q	L	A	pT	Kme	A	A	R	K	S	A	P
		G10	K18me3/pT22/K23me	T	G	G	K	A	P	R	Kme3	Q	L	A	pT	Kme	A	A	R	K	S	A	P
		G11	K18ac/pT22/K23me	T	G	G	K	A	P	R	Kac	Q	L	A	pT	Kme	A	A	R	K	S	A	P
		G12	K18me/pT22/K23me2	T	G	G	K	A	P	R	Kme	Q	L	A	pT	Kme2	A	A	R	K	S	A	P
		G13	K18me2/pT22/K23me2	T	G	G	K	A	P	R	Kme2	Q	L	A	pT	Kme2	A	A	R	K	S	A	P
		G14	K18me3/pT22/K23me2	T	G	G	K	A	P	R	Kme3	Q	L	A	pT	Kme2	A	A	R	K	S	A	P
		G15	K18ac/pT22/K23me2	T	G	G	K	A	P	R	Kac	Q	L	A	pT	Kme2	A	A	R	K	S	A	P
		G16	K18me/pT22/K23me3	T	G	G	K	A	P	R	Kme	Q	L	A	pT	Kme3	A	A	R	K	S	A	P
		G17	K18me2/pT22/K23me3	T	G	G	K	A	P	R	Kme2	Q	L	A	pT	Kme3	A	A	R	K	S	A	P
		G18	K18me3/pT22/K23me3	T	G	G	K	A	P	R	Kme3	Q	L	A	pT	Kme3	A	A	R	K	S	A	P
		H1	K18ac/pT22/K23me3	T	G	G	K	A	P	R	Kac	Q	L	A	pT	Kme3	A	A	R	K	S	A	P
		H2	K18me/pT22/K23ac	T	G	G	K	A	P	R	Kme	Q	L	A	pT	Kac	A	A	R	K	S	A	P
		H3	K18me2/pT22/K23ac	T	G	G	K	A	P	R	Kme2	Q	L	A	pT	Kac	A	A	R	K	S	A	P
		H4	K18me3/pT22/K23ac	T	G	G	K	A	P	R	Kme3	Q	L	A	pT	Kac	A	A	R	K	S	A	P
		H5	K18ac/pT22/K23ac	T	G	G	K	A	P	R	Kac	Q	L	A	pT	Kac	A	A	R	K	S	A	P
		H6	K14ac/pT22/K18me	T	G	G	Kac	A	P	R	Kme	Q	L	A	pT	K	A	A	R	K	S	A	P
		H7	K14ac/pT22/K18me2	T	G	G	Kac	A	P	R	Kme2	Q	L	A	pT	K	A	A	R	K	S	A	P
		H8	K14ac/pT22/K18me3	T	G	G	Kac	A	P	R	Kme3	Q	L	A	pT	K	A	A	R	K	S	A	P
		H9	K14ac/pT22/K18ac	T	G	G	Kac	A	P	R	Kac	Q	L	A	pT	K	A	A	R	K	S	A	P
		H10	K14ac/K18me/K23me	T	G	G	Kac	A	P	R	Kme	Q	L	A	T	Kme	A	A	R	K	S	A	P
		H11	K14ac/K18me2/K23me	T	G	G	Kac	A	P	R	Kme2	Q	L	A	T	Kme	A	A	R	K	S	A	P
		H12	K14ac/K18me3/K23me	T	G	G	Kac	A	P	R	Kme3	Q	L	A	T	Kme	A	A	R	K	S	A	P
		H13	K14ac/K18ac/K23me	T	G	G	Kac	A	P	R	Kac	Q	L	A	T	Kme	A	A	R	K	S	A	P
		H14	K14ac/K18me/K23me2	T	G	G	Kac	A	P	R	Kme	Q	L	A	T	Kme2	A	A	R	K	S	A	P
		H15	K14ac/K18me2/K23me2	T	G	G	Kac	A	P	R	Kme2	Q	L	A	T	Kme2	A	A	R	K	S	A	P
		H16	K14ac/K18me3/K23me2	T	G	G	Kac	A	P	R	Kme3	Q	L	A	T	Kme2	A	A	R	K	S	A	P
		H17	K14ac/K18ac/K23me2	T	G	G	Kac	A	P	R	Kac	Q	L	A	T	Kme2	A	A	R	K	S	A	P
		H18	K14ac/K18me/K23me3	T	G	G	Kac	A	P	R	Kme	Q	L	A	T	Kme3	A	A	R	K	S	A	P
		I1	K14ac/K18me2/K23me3	T	G	G	Kac	A	P	R	Kme2	Q	L	A	T	Kme3	A	A	R	K	S	A	P
		I2	K14ac/K18me3/K23me3	T	G	G	Kac	A	P	R	Kme3	Q	L	A	T	Kme3	A	A	R	K	S	A	P
		I3	K14ac/K18ac/K23me3	T	G	G	Kac	A	P	R	Kac	Q	L	A	T	Kme3	A	A	R	K	S	A	P
		I4	K14ac/K18me/K23ac	T	G	G	Kac	A	P	R	Kme	Q	L	A	T	Kac	A	A	R	K	S	A	P
		I5	K14ac/K18me2/K23ac	T	G	G	Kac	A	P	R	Kme2	Q	L	A	T	Kac	A	A	R	K	S	A	P
		I6	K14ac/K18me3/K23ac	T	G	G	Kac	A	P	R	Kme3	Q	L	A	T	Kac	A	A	R	K	S	A	P
		I7	K14ac/K18ac/K23ac	T	G	G	Kac	A	P	R	Kac	Q	L	A	T	Kac	A	A	R	K	S	A	P
						18				22	23				27	28				32			
H3.1 (P68431)	16-35	I8	CONTROL	P	R	K	Q	L	A	T	K	A	A	R	K	S	A	P	A	T	G	G	V
		I9	pT22	P	R	K	Q	L	A	pT	K	A	A	R	K	S	A	P	A	T	G	G	V
		I10	K23me	P	R	K	Q	L	A	T	Kme	A	A	R	K	S	A	P	A	T	G	G	V
		I11	K23me2	P	R	K	Q	L	A	T	Kme2	A	A	R	K	S	A	P	A	T	G	G	V
		I12	K23me3	P	R	K	Q	L	A	T	Kme3	A	A	R	K	S	A	P	A	T	G	G	V
		I13	K23ac	P	R	K	Q	L	A	T	Kac	A	A	R	K	S	A	P	A	T	G	G	V
		I14	pT22/K23me	P	R	K	Q	L	A	pT	Kme	A	A	R	K	S	A	P	A	T	G	G	V
		I15	pT22/K23me2	P	R	K	Q	L	A	pT	Kme2	A	A	R	K	S	A	P	A	T	G	G	V
		I16	pT22/K23me3	P	R	K	Q	L	A	pT	Kme3	A	A	R	K	S	A	P	A	T	G	G	V
		I17	pT22/K23ac	P	R	K	Q	L	A	pT	Kac	A	A	R	K	S	A	P	A	T	G	G	V
		I18	K27me	P	R	K	Q	L	A	T	K	A	A	R	Kme	S	A	P	A	T	G	G	V
		J1	K27me2	P	R	K	Q	L	A	T	K	A	A	R	Kme2	S	A	P	A	T	G	G	V
		J2	K27me3	P	R	K	Q	L	A	T	K	A	A	R	Kme3	S	A	P	A	T	G	G	V
		J3	K27ac	P	R	K	Q	L	A	T	K	A	A	R	Kac	S	A	P	A	T	G	G	V
		J4	K23me/K27me	P	R	K	Q	L	A	T	Kme	A	A	R	Kme	S	A	P	A	T	G	G	V
		J5	K23me2/K27me	P	R	K	Q	L	A	T	Kme2	A	A	R	Kme	S	A	P	A	T	G	G	V
		J6	K23me3/K27me	P	R	K	Q	L	A	T	Kme3	A	A	R	Kme	S	A	P	A	T	G	G	V
		J7	K23ac/K27me	P	R	K	Q	L	A	T	Kac	A	A	R	Kme	S	A	P	A	T	G	G	V
		J8	K23me/K27me2	P	R	K	Q	L	A	T	Kme	A	A	R	Kme2	S	A	P	A	T	G	G	V
		J9	K23me2/K27me2	P	R	K	Q	L	A	T	Kme2	A	A	R	Kme2	S	A	P	A	T	G	G	V
		J10	K23me3/K27me2	P	R	K	Q	L	A	T	Kme3	A	A	R	Kme2	S	A	P	A	T	G	G	V
		J11	K23ac/K27me2	P	R	K	Q	L	A	T	Kac	A	A	R	Kme2	S	A	P	A	T	G	G	V
		J12	K23me/K27me3	P	R	K	Q	L	A	T	Kme	A	A	R	Kme3	S	A	P	A	T	G	G	V
		J13	K23me2/K27me3	P	R	K	Q	L	A	T	Kme2	A	A	R	Kme3	S	A	P	A	T	G	G	V
		J14	K23me3/K27me3	P	R	K	Q	L	A	T	Kme3	A	A	R	Kme3	S	A	P	A	T	G	G	V
		J15	K23ac/K27me3	P	R	K	Q	L	A	T	Kac	A	A	R	Kme3	S	A	P	A	T	G	G	V
		J16	K23me/K27ac	P	R	K	Q	L	A	T	Kme	A	A	R	Kac	S	A	P	A	T	G	G	V
		J17	K23me2/K27ac	P	R	K	Q	L	A	T	Kme2	A	A	R	Kac	S	A	P	A	T	G	G	V
		J18	K23me3/K27ac	P	R	K	Q	L	A	T	Kme3	A	A	R	Kac	S	A	P	A	T	G	G	V
		K1	K23ac/K27ac	P	R	K	Q	L	A	T	Kac	A	A	R	Kac	S	A	P	A	T	G	G	V
		K2	pT22/K23me/K27me	P	R	K	Q	L	A	pT	Kme	A	A	R	Kme	S	A	P	A	T	G	G	V
		K3	pT22/K23me2/K27me	P	R	K	Q	L	A	pT	Kme2	A	A	R	Kme	S	A	P	A	T	G	G	V
		K4	pT22/K23me3/K27me	P	R	K	Q	L	A	pT	Kme3	A	A	R	Kme	S	A	P	A	T	G	G	V
		K5	pT22/K23ac/K27me	P	R	K	Q	L	A	pT	Kac	A	A	R	Kme	S	A	P	A	T	G	G	V
		K6	pT22/K23me/K27me2	P	R	K	Q	L	A	pT	Kme	A	A	R	Kme2	S	A	P	A	T	G	G	V
		K7	pT22/K23me2/K27me2	P	R	K	Q	L	A	pT	Kme2	A	A	R	Kme2	S	A	P	A	T	G	G	V
		K8	pT22/K23me3/K27me2	P	R	K	Q	L	A	pT	Kme3	A	A	R	Kme2	S	A	P	A	T	G	G	V
		K9	pT22/K23ac/K27me2	P	R	K	Q	L	A	pT	Kac	A	A	R	Kme2	S	A	P	A	T	G	G	V
		K10	pT22/K23me/K27me3	P	R	K	Q	L	A	pT	Kme	A	A	R	Kme3	S	A	P	A	T	G	G	V
		K11	pT22/K23me2/K27me3	P	R	K	Q	L	A	pT	Kme2	A	A	R	Kme3	S	A	P	A	T	G	G	V
		K12	pT22/K23me3/K27me3	P	R	K	Q	L	A	pT	Kme3	A	A	R	Kme3	S	A	P	A	T	G	G	V
		K13	pT22/K23ac/K27me3	P	R	K	Q	L	A	pT	Kac	A	A	R	Kme3	S	A	P	A	T	G	G	V
		K14	pT22/K23me/K27ac	P	R	K	Q	L	A	pT	Kme	A	A	R	Kac	S	A	P	A	T	G	G	V
		K15	pT22/K23me2/K27ac	P	R	K	Q	L	A	pT	Kme2	A	A	R	Kac	S	A	P	A	T	G	G	V
		K16	pT22/K23me3/K27ac	P	R	K	Q	L	A	pT	Kme3	A	A	R	Kac	S	A	P	A	T	G	G	V
		K17	pT22/K23ac/K27ac	P	R	K	Q	L	A	pT	Kac	A	A	R	Kac	S	A	P	A	T	G	G	V

		K18	pS28	P	R	K	Q	L	A	T	K	A	A	R	K	pS	A	P	A	T	G	G	V
		L1	K27me/pS28	P	R	K	Q	L	A	T	K	A	A	R	Kme	pS	A	P	A	T	G	G	V
		L2	K27me2/pS28	P	R	K	Q	L	A	T	K	A	A	R	Kme2	pS	A	P	A	T	G	G	V
		L3	K27me3/pS28	P	R	K	Q	L	A	T	K	A	A	R	Kme3	pS	A	P	A	T	G	G	V
		L4	K27ac/pS28	P	R	K	Q	L	A	T	K	A	A	R	Kac	pS	A	P	A	T	G	G	V
		L5	K23me/K27me/pS28	P	R	K	Q	L	A	T	Kme	A	A	R	Kme	pS	A	P	A	T	G	G	V
		L6	K23me2/K27me/pS28	P	R	K	Q	L	A	T	Kme2	A	A	R	Kme	pS	A	P	A	T	G	G	V
		L7	K23me3/K27me/pS28	P	R	K	Q	L	A	T	Kme3	A	A	R	Kme	pS	A	P	A	T	G	G	V
		L8	K23ac/K27me/pS28	P	R	K	Q	L	A	T	Kac	A	A	R	Kme	pS	A	P	A	T	G	G	V
		L9	K23me/K27me2/pS28	P	R	K	Q	L	A	T	Kme	A	A	R	Kme2	pS	A	P	A	T	G	G	V
		L10	K23me2/K27me2/pS28	P	R	K	Q	L	A	T	Kme2	A	A	R	Kme2	pS	A	P	A	T	G	G	V
		L11	K23me3/K27me2/pS28	P	R	K	Q	L	A	T	Kme3	A	A	R	Kme2	pS	A	P	A	T	G	G	V
		L12	K23ac/K27me2/pS28	P	R	K	Q	L	A	T	Kac	A	A	R	Kme2	pS	A	P	A	T	G	G	V
		L13	K23me/K27me3/pS28	P	R	K	Q	L	A	T	Kme	A	A	R	Kme3	pS	A	P	A	T	G	G	V
		L14	K23me2/K27me3/pS28	P	R	K	Q	L	A	T	Kme2	A	A	R	Kme3	pS	A	P	A	T	G	G	V
		L15	K23me3/K27me3/pS28	P	R	K	Q	L	A	T	Kme3	A	A	R	Kme3	pS	A	P	A	T	G	G	V
		L16	K23ac/K27me3/pS28	P	R	K	Q	L	A	T	Kac	A	A	R	Kme3	pS	A	P	A	T	G	G	V
		L17	K23me/K27ac/pS28	P	R	K	Q	L	A	T	Kme	A	A	R	Kac	pS	A	P	A	T	G	G	V
		L18	K23me2/K27ac/pS28	P	R	K	Q	L	A	T	Kme2	A	A	R	Kac	pS	A	P	A	T	G	G	V
		M1	K23me3/K27ac/pS28	P	R	K	Q	L	A	T	Kme3	A	A	R	Kac	pS	A	P	A	T	G	G	V
		M2	K23ac/K27ac/pS28	P	R	K	Q	L	A	T	Kac	A	A	R	Kac	pS	A	P	A	T	G	G	V
		M3	pT22/pS28	P	R	K	Q	L	A	T	pT	A	A	R	K	pS	A	P	A	T	G	G	V
					22	23				27	28				32					36	37		
**H3.1 (P68431)**	21-40	M4	CONTROL	A	T	K	A	A	R	K	S	A	P	A	T	G	G	V	K	K	P	H	R
		M5	K27me	A	T	K	A	A	R	Kme	S	A	P	A	T	G	G	V	K	K	P	H	R
		M6	K27me2	A	T	K	A	A	R	Kme2	S	A	P	A	T	G	G	V	K	K	P	H	R
		M7	K27me3	A	T	K	A	A	R	Kme3	S	A	P	A	T	G	G	V	K	K	P	H	R
		M8	K27ac	A	T	K	A	A	R	Kac	S	A	P	A	T	G	G	V	K	K	P	H	R
		M9	pS28	A	T	K	A	A	R	K	pS	A	P	A	T	G	G	V	K	K	P	H	R
		M10	K27me/pS28	A	T	K	A	A	R	Kme	pS	A	P	A	T	G	G	V	K	K	P	H	R
		M11	K27me2/pS28	A	T	K	A	A	R	Kme2	pS	A	P	A	T	G	G	V	K	K	P	H	R
		M12	K27me3/pS28	A	T	K	A	A	R	Kme3	pS	A	P	A	T	G	G	V	K	K	P	H	R
		M13	K27ac/pS28	A	T	K	A	A	R	Kac	pS	A	P	A	T	G	G	V	K	K	P	H	R
		M14	pT32	A	T	K	A	A	R	K	S	A	P	A	pT	G	G	V	K	K	P	H	R
		M15	pS28/pT32	A	T	K	A	A	R	K	pS	A	P	A	pT	G	G	V	K	K	P	H	R
		M16	K27me/pS28/pT32	A	T	K	A	A	R	Kme	pS	A	P	A	pT	G	G	V	K	K	P	H	R
		M17	K27me2/pS28/pT32	A	T	K	A	A	R	Kme2	pS	A	P	A	pT	G	G	V	K	K	P	H	R
		M18	K27me3/pS28/pT32	A	T	K	A	A	R	Kme3	pS	A	P	A	pT	G	G	V	K	K	P	H	R
		N1	K27ac/pS28/pT32	A	T	K	A	A	R	Kac	pS	A	P	A	pT	G	G	V	K	K	P	H	R
					27	28				32				36	37								
**H3.1 (P68431)**	26-45	N2	CONTROL	R	K	S	A	P	A	T	G	G	V	K	K	P	H	R	Y	R	P	G	T
		N3	K36me	R	K	S	A	P	A	T	G	G	V	Kme	K	P	H	R	Y	R	P	G	T
		N4	K36me2	R	K	S	A	P	A	T	G	G	V	Kme2	K	P	H	R	Y	R	P	G	T
		N5	K36me3	R	K	S	A	P	A	T	G	G	V	Kme3	K	P	H	R	Y	R	P	G	T
		N6	K36ac	R	K	S	A	P	A	T	G	G	V	Kac	K	P	H	R	Y	R	P	G	T
		N7	pT32	R	K	S	A	P	A	pT	G	G	V	K	K	P	H	R	Y	R	P	G	T
		N8	pT32/K36me	R	K	S	A	P	A	pT	G	G	V	Kme	K	P	H	R	Y	R	P	G	T
		N9	pT32/K36me2	R	K	S	A	P	A	pT	G	G	V	Kme2	K	P	H	R	Y	R	P	G	T
		N10	pT32/K36me3	R	K	S	A	P	A	pT	G	G	V	Kme3	K	P	H	R	Y	R	P	G	T
		N11	pT32/K36ac	R	K	S	A	P	A	pT	G	G	V	Kac	K	P	H	R	Y	R	P	G	T
		N12	K37me	R	K	S	A	P	A	T	G	G	V	K	Kme	P	H	R	Y	R	P	G	T
		N13	K37me2	R	K	S	A	P	A	T	G	G	V	K	Kme2	P	H	R	Y	R	P	G	T
		N14	K37me3	R	K	S	A	P	A	T	G	G	V	K	Kme3	P	H	R	Y	R	P	G	T
		N15	K37ac	R	K	S	A	P	A	T	G	G	V	K	Kac	P	H	R	Y	R	P	G	T
		N16	pT32/K37me	R	K	S	A	P	A	pT	G	G	V	K	Kme	P	H	R	Y	R	P	G	T
		N17	pT32/K37me2	R	K	S	A	P	A	pT	G	G	V	K	Kme2	P	H	R	Y	R	P	G	T
		N18	pT32/K37me3	R	K	S	A	P	A	pT	G	G	V	K	Kme3	P	H	R	Y	R	P	G	T
		O1	pT32/K37ac	R	K	S	A	P	A	pT	G	G	V	K	Kac	P	H	R	Y	R	P	G	T
		O2	K36me/K37me	R	K	S	A	P	A	T	G	G	V	Kme	Kme	P	H	R	Y	R	P	G	T
		O3	K36me2/K37me	R	K	S	A	P	A	T	G	G	V	Kme2	Kme	P	H	R	Y	R	P	G	T
		O4	K36me3/K37me	R	K	S	A	P	A	T	G	G	V	Kme3	Kme	P	H	R	Y	R	P	G	T
		O5	K36ac/K37me	R	K	S	A	P	A	T	G	G	V	Kac	Kme	P	H	R	Y	R	P	G	T
		O6	K36me/K37me2	R	K	S	A	P	A	T	G	G	V	Kme	Kme2	P	H	R	Y	R	P	G	T
		O7	K36me2/K37me2	R	K	S	A	P	A	T	G	G	V	Kme2	Kme2	P	H	R	Y	R	P	G	T
		O8	K36me3/K37me2	R	K	S	A	P	A	T	G	G	V	Kme3	Kme2	P	H	R	Y	R	P	G	T
		O9	K36ac/K37me2	R	K	S	A	P	A	T	G	G	V	Kac	Kme2	P	H	R	Y	R	P	G	T
		O10	K36me/K37me3	R	K	S	A	P	A	T	G	G	V	Kme	Kme3	P	H	R	Y	R	P	G	T
		O11	K36me2/K37me3	R	K	S	A	P	A	T	G	G	V	Kme2	Kme3	P	H	R	Y	R	P	G	T
		O12	K36me3/K37me3	R	K	S	A	P	A	T	G	G	V	Kme3	Kme3	P	H	R	Y	R	P	G	T
		O13	K36ac/K37me3	R	K	S	A	P	A	T	G	G	V	Kac	Kme3	P	H	R	Y	R	P	G	T
		O14	K36me/K37ac	R	K	S	A	P	A	T	G	G	V	Kme	Kac	P	H	R	Y	R	P	G	T
		O15	K36me2/K37ac	R	K	S	A	P	A	T	G	G	V	Kme2	Kac	P	H	R	Y	R	P	G	T
		O16	K36me3/K37ac	R	K	S	A	P	A	T	G	G	V	Kme3	Kac	P	H	R	Y	R	P	G	T
		O17	K36ac/K37ac	R	K	S	A	P	A	T	G	G	V	Kac	Kac	P	H	R	Y	R	P	G	T
		O18	pT32/K36me/K37me	R	K	S	A	P	A	pT	G	G	V	Kme	Kme	P	H	R	Y	R	P	G	T
		P1	pT32/K36me2/K37me	R	K	S	A	P	A	pT	G	G	V	Kme2	Kme	P	H	R	Y	R	P	G	T
		P2	pT32/K36me3/K37me	R	K	S	A	P	A	pT	G	G	V	Kme3	Kme	P	H	R	Y	R	P	G	T
		P3	pT32/K36ac/K37me	R	K	S	A	P	A	pT	G	G	V	Kac	Kme	P	H	R	Y	R	P	G	T
		P4	pT32/K36me/K37me2	R	K	S	A	P	A	pT	G	G	V	Kme	Kme2	P	H	R	Y	R	P	G	T
		P5	pT32/K36me2/K37me2	R	K	S	A	P	A	pT	G	G	V	Kme2	Kme2	P	H	R	Y	R	P	G	T
		P6	pT32/K36me3/K37me2	R	K	S	A	P	A	pT	G	G	V	Kme3	Kme2	P	H	R	Y	R	P	G	T
		P7	pT32/K36ac/K37me2	R	K	S	A	P	A	pT	G	G	V	Kac	Kme2	P	H	R	Y	R	P	G	T
		P8	pT32/K36me/K37me3	R	K	S	A	P	A	pT	G	G	V	Kme	Kme3	P	H	R	Y	R	P	G	T
		P9	pT32/K36me2/K37me3	R	K	S	A	P	A	pT	G	G	V	Kme2	Kme3	P	H	R	Y	R	P	G	T
		P10	pT32/K36me3/K37me3	R	K	S	A	P	A	pT	G	G	V	Kme3	Kme3	P	H	R	Y	R	P	G	T
		P11	pT32/K36ac/K37me3	R	K	S	A	P	A	pT	G	G	V	Kac	Kme3	P	H	R	Y	R	P	G	T
		P12	pT32/K36me/K37ac	R	K	S	A	P	A	pT	G	G	V	Kme	Kac	P	H	R	Y	R	P	G	T
		P13	pT32/K36me2/K37ac	R	K	S	A	P	A	pT	G	G	V	Kme2	Kac	P	H	R	Y	R	P	G	T
		P14	pT32/K36me3/K37ac	R	K	S	A	P	A	pT	G	G	V	Kme3	Kac	P	H	R	Y	R	P	G	T
		P15	pT32/K36ac/K37ac	R	K	S	A	P	A	pT	G	G	V	Kac	Kac	P	H	R	Y	R	P	G	T
		P16	blank																				
polyHis CTRL		P17	HHHHHHHHHH																				
polyHis CTRL		P18	HHHHHHHHHH																				

Histone	Peptide Length	Array Poition	Kac Mark Combinations	Sequence																			
						18				22	23				27	28				32			
H3.1t (Q16695)	16-35	A1	CONTROL	P	R	K	Q	L	A	T	K	V	A	R	K	S	A	P	A	T	G	G	V
		A2	pT22	P	R	K	Q	L	A	pT	K	V	A	R	K	S	A	P	A	T	G	G	V
		A3	K23me	P	R	K	Q	L	A	T	Kme	V	A	R	K	S	A	P	A	T	G	G	V
		A4	K23me2	P	R	K	Q	L	A	T	Kme2	V	A	R	K	S	A	P	A	T	G	G	V
		A5	K23me3	P	R	K	Q	L	A	T	Kme3	V	A	R	K	S	A	P	A	T	G	G	V
		B1	K23ac	P	R	K	Q	L	A	T	Kac	V	A	R	K	S	A	P	A	T	G	G	V
		B2	pT22/K23me	P	R	K	Q	L	A	pT	Kme	V	A	R	K	S	A	P	A	T	G	G	V
		B3	pT22/K23me2	P	R	K	Q	L	A	pT	Kme2	V	A	R	K	S	A	P	A	T	G	G	V
		B4	pT22/K23me3	P	R	K	Q	L	A	pT	Kme3	V	A	R	K	S	A	P	A	T	G	G	V
		B5	pT22/K23ac	P	R	K	Q	L	A	pT	Kac	V	A	R	K	S	A	P	A	T	G	G	V
		C1	K27me	P	R	K	Q	L	A	T	K	V	A	R	Kme	S	A	P	A	T	G	G	V
		C2	K27me2	P	R	K	Q	L	A	T	K	V	A	R	Kme2	S	A	P	A	T	G	G	V
		C3	K27me3	P	R	K	Q	L	A	T	K	V	A	R	Kme3	S	A	P	A	T	G	G	V
		C4	K27ac	P	R	K	Q	L	A	T	K	V	A	R	Kac	S	A	P	A	T	G	G	V
		C5	K23me/K27me	P	R	K	Q	L	A	T	Kme	V	A	R	Kme	S	A	P	A	T	G	G	V
		D1	K23me2/K27me	P	R	K	Q	L	A	T	Kme2	V	A	R	Kme	S	A	P	A	T	G	G	V
		D2	K23me3/K27me	P	R	K	Q	L	A	T	Kme3	V	A	R	Kme	S	A	P	A	T	G	G	V
		D3	K23ac/K27me	P	R	K	Q	L	A	T	Kac	V	A	R	Kme	S	A	P	A	T	G	G	V
		D4	K23me/K27me2	P	R	K	Q	L	A	T	Kme	V	A	R	Kme2	S	A	P	A	T	G	G	V
		D5	K23me2/K27me2	P	R	K	Q	L	A	T	Kme2	V	A	R	Kme2	S	A	P	A	T	G	G	V
		E1	K23me3/K27me2	P	R	K	Q	L	A	T	Kme3	V	A	R	Kme2	S	A	P	A	T	G	G	V
		E2	K23ac/K27me2	P	R	K	Q	L	A	T	Kac	V	A	R	Kme2	S	A	P	A	T	G	G	V
		E3	K23me/K27me3	P	R	K	Q	L	A	T	Kme	V	A	R	Kme3	S	A	P	A	T	G	G	V
		E4	K23me2/K27me3	P	R	K	Q	L	A	T	Kme2	V	A	R	Kme3	S	A	P	A	T	G	G	V
		E5	K23me3/K27me3	P	R	K	Q	L	A	T	Kme3	V	A	R	Kme3	S	A	P	A	T	G	G	V
		F1	K23ac/K27me3	P	R	K	Q	L	A	T	Kac	V	A	R	Kme3	S	A	P	A	T	G	G	V
		F2	K23me/K27ac	P	R	K	Q	L	A	T	Kme	V	A	R	Kac	S	A	P	A	T	G	G	V
		F3	K23me2/K27ac	P	R	K	Q	L	A	T	Kme2	V	A	R	Kac	S	A	P	A	T	G	G	V
		F4	K23me3/K27ac	P	R	K	Q	L	A	T	Kme3	V	A	R	Kac	S	A	P	A	T	G	G	V
		F5	K23ac/K27ac	P	R	K	Q	L	A	T	Kac	V	A	R	Kac	S	A	P	A	T	G	G	V
		G1	pT22/K23me/K27me	P	R	K	Q	L	A	pT	Kme	V	A	R	Kme	S	A	P	A	T	G	G	V
		G2	pT22/K23me2/K27me	P	R	K	Q	L	A	pT	Kme2	V	A	R	Kme	S	A	P	A	T	G	G	V
		G3	pT22/K23me3/K27me	P	R	K	Q	L	A	pT	Kme3	V	A	R	Kme	S	A	P	A	T	G	G	V
		G4	pT22/K23ac/K27me	P	R	K	Q	L	A	pT	Kac	V	A	R	Kme	S	A	P	A	T	G	G	V
		G5	pT22/K23me/K27me2	P	R	K	Q	L	A	pT	Kme	V	A	R	Kme2	S	A	P	A	T	G	G	V
		H1	pT22/K23me2/K27me2	P	R	K	Q	L	A	pT	Kme2	V	A	R	Kme2	S	A	P	A	T	G	G	V
		H2	pT22/K23me3/K27me2	P	R	K	Q	L	A	pT	Kme3	V	A	R	Kme2	S	A	P	A	T	G	G	V
		H3	pT22/K23ac/K27me2	P	R	K	Q	L	A	pT	Kac	V	A	R	Kme2	S	A	P	A	T	G	G	V
		H4	pT22/K23me/K27me3	P	R	K	Q	L	A	pT	Kme	V	A	R	Kme3	S	A	P	A	T	G	G	V
		H5	pT22/K23me2/K27me3	P	R	K	Q	L	A	pT	Kme2	V	A	R	Kme3	S	A	P	A	T	G	G	V
		I1	pT22/K23me3/K27me3	P	R	K	Q	L	A	pT	Kme3	V	A	R	Kme3	S	A	P	A	T	G	G	V
		I2	pT22/K23ac/K27me3	P	R	K	Q	L	A	pT	Kac	V	A	R	Kme3	S	A	P	A	T	G	G	V
		I3	pT22/K23me/K27ac	P	R	K	Q	L	A	pT	Kme	V	A	R	Kac	S	A	P	A	T	G	G	V
		I4	pT22/K23me2/K27ac	P	R	K	Q	L	A	pT	Kme2	V	A	R	Kac	S	A	P	A	T	G	G	V
		I5	pT22/K23me3/K27ac	P	R	K	Q	L	A	pT	Kme3	V	A	R	Kac	S	A	P	A	T	G	G	V
		J1	pT22/K23ac/K27ac	P	R	K	Q	L	A	pT	Kac	V	A	R	Kac	S	A	P	A	T	G	G	V
		J2	pS28	P	R	K	Q	L	A	T	K	V	A	R	K	pS	A	P	A	T	G	G	V
		J3	K27me/pS28	P	R	K	Q	L	A	T	K	V	A	R	Kme	pS	A	P	A	T	G	G	V
		J4	K27me2/pS28	P	R	K	Q	L	A	T	K	V	A	R	Kme2	pS	A	P	A	T	G	G	V
		J5	K27me3/pS28	P	R	K	Q	L	A	T	K	V	A	R	Kme3	pS	A	P	A	T	G	G	V
		K1	K27ac/pS28	P	R	K	Q	L	A	T	K	V	A	R	Kac	pS	A	P	A	T	G	G	V
		K2	K23me/K27me/pS28	P	R	K	Q	L	A	T	Kme	V	A	R	Kme	pS	A	P	A	T	G	G	V
		K3	K23me2/K27me/pS28	P	R	K	Q	L	A	T	Kme2	V	A	R	Kme	pS	A	P	A	T	G	G	V
		K4	K23me3/K27me/pS28	P	R	K	Q	L	A	T	Kme3	V	A	R	Kme	pS	A	P	A	T	G	G	V
		K5	K23ac/K27me/pS28	P	R	K	Q	L	A	T	Kac	V	A	R	Kme	pS	A	P	A	T	G	G	V
		L1	K23me/K27me2/pS28	P	R	K	Q	L	A	T	Kme	V	A	R	Kme2	pS	A	P	A	T	G	G	V
		L2	K23me2/K27me2/pS28	P	R	K	Q	L	A	T	Kme2	V	A	R	Kme2	pS	A	P	A	T	G	G	V
		L3	K23me3/K27me2/pS28	P	R	K	Q	L	A	T	Kme3	V	A	R	Kme2	pS	A	P	A	T	G	G	V
		L4	K23ac/K27me2/pS28	P	R	K	Q	L	A	T	Kac	V	A	R	Kme2	pS	A	P	A	T	G	G	V
		L5	K23me/K27me3/pS28	P	R	K	Q	L	A	T	Kme	V	A	R	Kme3	pS	A	P	A	T	G	G	V
		M1	K23me2/K27me3/pS28	P	R	K	Q	L	A	T	Kme2	V	A	R	Kme3	pS	A	P	A	T	G	G	V
		M2	K23me3/K27me3/pS28	P	R	K	Q	L	A	T	Kme3	V	A	R	Kme3	pS	A	P	A	T	G	G	V
		M3	K23ac/K27me3/pS28	P	R	K	Q	L	A	T	Kac	V	A	R	Kme3	pS	A	P	A	T	G	G	V
		M4	K23me/K27ac/pS28	P	R	K	Q	L	A	T	Kme	V	A	R	Kac	pS	A	P	A	T	G	G	V
		M5	K23me2/K27ac/pS28	P	R	K	Q	L	A	T	Kme2	V	A	R	Kac	pS	A	P	A	T	G	G	V
		N1	K23me3/K27ac/pS28	P	R	K	Q	L	A	T	Kme3	V	A	R	Kac	pS	A	P	A	T	G	G	V
		N2	K23ac/K27ac/pS28	P	R	K	Q	L	A	T	Kac	V	A	R	Kac	pS	A	P	A	T	G	G	V
		N3	pT22/pS28	P	R	K	Q	L	A	T	pT	V	A	R	K	pS	A	P	A	T	G	G	V
					22	23				27	28				32				36	37			
H3.1t (Q16695)	21-40	N4	CONTROL	A	T	K	V	A	R	K	S	A	P	A	T	G	G	V	K	K	P	H	R
		N5	K27me	A	T	K	V	A	R	Kme	S	A	P	A	T	G	G	V	K	K	P	H	R
		O1	K27me2	A	T	K	V	A	R	Kme2	S	A	P	A	T	G	G	V	K	K	P	H	R
		O2	K27me3	A	T	K	V	A	R	Kme3	S	A	P	A	T	G	G	V	K	K	P	H	R
		O3	K27ac	A	T	K	V	A	R	Kac	S	A	P	A	T	G	G	V	K	K	P	H	R
		O4	pS28	A	T	K	V	A	R	K	pS	A	P	A	T	G	G	V	K	K	P	H	R
		O5	K27me/pS28	A	T	K	V	A	R	Kme	pS	A	P	A	T	G	G	V	K	K	P	H	R
		P1	K27me2/pS28	A	T	K	V	A	R	Kme2	pS	A	P	A	T	G	G	V	K	K	P	H	R
		P2	K27me3/pS28	A	T	K	V	A	R	Kme3	pS	A	P	A	T	G	G	V	K	K	P	H	R

	P3	K27ac/pS28	A	T	K	**V**	A	R	**Kac**	**pS**	A	P	A	T	G	G	V	K	K	P	H	R
	P4	pT32	A	T	K	**V**	A	R	K	S	A	P	A	**pT**	G	G	V	K	K	P	H	R
	P5	pS28/pT32	A	T	K	**V**	A	R	K	**pS**	A	P	A	**pT**	G	G	V	K	K	P	H	R
	Q1	K27me/pS28/pT32	A	T	K	**V**	A	R	**Kme**	**pS**	A	P	A	**pT**	G	G	V	K	K	P	H	R
	Q2	K27me2/pS28/pT32	A	T	K	**V**	A	R	**Kme2**	**pS**	A	P	A	**pT**	G	G	V	K	K	P	H	R
	Q3	K27me3/pS28/pT32	A	T	K	**V**	A	R	**Kme3**	**pS**	A	P	A	**pT**	G	G	V	K	K	P	H	R
	Q4	K27ac/pS28/pT32	A	T	K	**V**	A	R	**Kac**	**pS**	A	P	A	**pT**	G	G	V	K	K	P	H	R
polyHis CTRL	Q5	HHHHHHHHHH																				

Histone	Peptide Length	Array Poition	Kac Mark Combinations	Sequence																			
polyHis CTRL		A1	HHHHHHHHH																				
polyHis CTRL		A2	HHHHHHHHH																				
					22	23				27	28			31	32				36	37			
**H3.3 (P84243)**	21-40	A3	CONTROL	A	T	K	A	A	R	K	S	A	P	S	T	G	G	V	K	K	P	H	R
		A4	K27me	A	T	K	A	A	R	Kme	S	A	P	S	T	G	G	V	K	K	P	H	R
		A5	K27me2	A	T	K	A	A	R	Kme2	S	A	P	S	T	G	G	V	K	K	P	H	R
		A6	K27me3	A	T	K	A	A	R	Kme3	S	A	P	S	T	G	G	V	K	K	P	H	R
		B1	K27ac	A	T	K	A	A	R	Kac	S	A	P	S	T	G	G	V	K	K	P	H	R
		B2	pS28	A	T	K	A	A	R	K	pS	A	P	S	T	G	G	V	K	K	P	H	R
		B3	K27me/pS28	A	T	K	A	A	R	Kme	pS	A	P	S	T	G	G	V	K	K	P	H	R
		B4	K27me2/pS28	A	T	K	A	A	R	Kme2	pS	A	P	S	T	G	G	V	K	K	P	H	R
		B5	K27me3/pS28	A	T	K	A	A	R	Kme3	pS	A	P	S	T	G	G	V	K	K	P	H	R
		B6	K27ac/pS28	A	T	K	A	A	R	Kac	pS	A	P	S	T	G	G	V	K	K	P	H	R
		C1	pT32	A	T	K	A	A	R	K	S	A	P	S	pT	G	G	V	K	K	P	H	R
		C2	pS28/pT32	A	T	K	A	A	R	K	pS	A	P	S	pT	G	G	V	K	K	P	H	R
		C3	K27me/pS28/pT32	A	T	K	A	A	R	Kme	pS	A	P	S	pT	G	G	V	K	K	P	H	R
		C4	K27me2/pS28/pT32	A	T	K	A	A	R	Kme2	pS	A	P	S	pT	G	G	V	K	K	P	H	R
		C5	K27me3/pS28/pT32	A	T	K	A	A	R	Kme3	pS	A	P	S	pT	G	G	V	K	K	P	H	R
		C6	K27ac/pS28/pT32	A	T	K	A	A	R	Kac	pS	A	P	S	pT	G	G	V	K	K	P	H	R
		D1	pS31	A	T	K	A	A	R	K	S	A	P	pS	T	G	G	V	K	K	P	H	R
		D2	K27me/pS31	A	T	K	A	A	R	Kme	S	A	P	pS	T	G	G	V	K	K	P	H	R
		D3	K27me2/pS31	A	T	K	A	A	R	Kme2	S	A	P	pS	T	G	G	V	K	K	P	H	R
		D4	K27me3/pS31	A	T	K	A	A	R	Kme3	S	A	P	pS	T	G	G	V	K	K	P	H	R
		D5	K27ac/pS31	A	T	K	A	A	R	Kac	S	A	P	pS	T	G	G	V	K	K	P	H	R
		D6	pS31/pT32	A	T	K	A	A	R	K	S	A	P	pS	pT	G	G	V	K	K	P	H	R

Histone	Peptide Length	Array Poition	Kac Mark Combinations	Sequence																			
					27	28			31	32				36	37								
**H3.3 (P84243)**	26-45	E1	CONTROL	R	K	S	A	P	S	T	G	G	V	K	K	P	H	R	Y	R	P	G	T
		E2	pS31	R	K	S	A	P	pS	T	G	G	V	K	K	P	H	R	Y	R	P	G	T
		E3	K36me	R	K	S	A	P	S	T	G	G	V	Kme	K	P	H	R	Y	R	P	G	T
		E4	K36me2	R	K	S	A	P	S	T	G	G	V	Kme2	K	P	H	R	Y	R	P	G	T
		E5	K36me3	R	K	S	A	P	S	T	G	G	V	Kme3	K	P	H	R	Y	R	P	G	T
		E6	K36ac	R	K	S	A	P	S	T	G	G	V	Kac	K	P	H	R	Y	R	P	G	T
		F1	pS31/K36me	R	K	S	A	P	pS	T	G	G	V	Kme	K	P	H	R	Y	R	P	G	T
		F2	pS31/K36me2	R	K	S	A	P	pS	T	G	G	V	Kme2	K	P	H	R	Y	R	P	G	T
		F3	pS31/K36me3	R	K	S	A	P	pS	T	G	G	V	Kme3	K	P	H	R	Y	R	P	G	T
		F4	pS31/K36ac	R	K	S	A	P	pS	T	G	G	V	Kac	K	P	H	R	Y	R	P	G	T
		F5	pT32	R	K	S	A	P	S	pT	G	G	V	K	K	P	H	R	Y	R	P	G	T
		F6	pT32/K36me	R	K	S	A	P	S	pT	G	G	V	Kme	K	P	H	R	Y	R	P	G	T
		G1	pT32/K36me2	R	K	S	A	P	S	pT	G	G	V	Kme2	K	P	H	R	Y	R	P	G	T
		G2	pT32/K36me3	R	K	S	A	P	S	pT	G	G	V	Kme3	K	P	H	R	Y	R	P	G	T
		G3	pT32/K36ac	R	K	S	A	P	S	pT	G	G	V	Kac	K	P	H	R	Y	R	P	G	T
		G4	K37me	R	K	S	A	P	S	T	G	G	V	K	Kme	P	H	R	Y	R	P	G	T
		G5	K37me2	R	K	S	A	P	S	T	G	G	V	K	Kme2	P	H	R	Y	R	P	G	T
		G6	K37me3	R	K	S	A	P	S	T	G	G	V	K	Kme3	P	H	R	Y	R	P	G	T
		H1	K37ac	R	K	S	A	P	S	T	G	G	V	K	Kac	P	H	R	Y	R	P	G	T
		H2	pS31/K37me	R	K	S	A	P	pS	T	G	G	V	K	Kme	P	H	R	Y	R	P	G	T
		H3	pS31/K37me2	R	K	S	A	P	pS	T	G	G	V	K	Kme2	P	H	R	Y	R	P	G	T
		H4	pS31/K37me3	R	K	S	A	P	pS	T	G	G	V	K	Kme3	P	H	R	Y	R	P	G	T
		H5	pS31/K37ac	R	K	S	A	P	pS	T	G	G	V	K	Kac	P	H	R	Y	R	P	G	T
		H6	pT32/K37me	R	K	S	A	P	S	pT	G	G	V	K	Kme	P	H	R	Y	R	P	G	T
		I1	pT32/K37me2	R	K	S	A	P	S	pT	G	G	V	K	Kme2	P	H	R	Y	R	P	G	T
		I2	pT32/K37me3	R	K	S	A	P	S	pT	G	G	V	K	Kme3	P	H	R	Y	R	P	G	T
		I3	pT32/K37ac	R	K	S	A	P	S	pT	G	G	V	K	Kac	P	H	R	Y	R	P	G	T
		I4	pS31/K36me/K37me	R	K	S	A	P	pS	T	G	G	V	Kme	Kme	P	H	R	Y	R	P	G	T
		I5	pS31/K36me2/K37me	R	K	S	A	P	pS	T	G	G	V	Kme2	Kme	P	H	R	Y	R	P	G	T
		I6	pS31/K36me3/K37me	R	K	S	A	P	pS	T	G	G	V	Kme3	Kme	P	H	R	Y	R	P	G	T
		J1	pS31/K36ac/K37me	R	K	S	A	P	pS	T	G	G	V	Kac	Kme	P	H	R	Y	R	P	G	T
		J2	pS31/K36me/K37me2	R	K	S	A	P	pS	T	G	G	V	Kme	Kme2	P	H	R	Y	R	P	G	T
		J3	pS31/K36me2/K37me2	R	K	S	A	P	pS	T	G	G	V	Kme2	Kme2	P	H	R	Y	R	P	G	T
		J4	pS31/K36me3/K37me2	R	K	S	A	P	pS	T	G	G	V	Kme3	Kme2	P	H	R	Y	R	P	G	T
		J5	pS31/K36ac/K37me2	R	K	S	A	P	pS	T	G	G	V	Kac	Kme2	P	H	R	Y	R	P	G	T
		J6	pS31/K36me/K37me3	R	K	S	A	P	pS	T	G	G	V	Kme	Kme3	P	H	R	Y	R	P	G	T
		K1	pS31/K36me2/K37me3	R	K	S	A	P	pS	T	G	G	V	Kme2	Kme3	P	H	R	Y	R	P	G	T
		K2	pS31/K36me3/K37me3	R	K	S	A	P	pS	T	G	G	V	Kme3	Kme3	P	H	R	Y	R	P	G	T
		K3	pS31/K36ac/K37me3	R	K	S	A	P	pS	T	G	G	V	Kac	Kme3	P	H	R	Y	R	P	G	T
		K4	pS31/K36me/K37ac	R	K	S	A	P	pS	T	G	G	V	Kme	Kac	P	H	R	Y	R	P	G	T
		K5	pS31/K36me2/K37ac	R	K	S	A	P	pS	T	G	G	V	Kme2	Kac	P	H	R	Y	R	P	G	T
		K6	pS31/K36me3/K37ac	R	K	S	A	P	pS	T	G	G	V	Kme3	Kac	P	H	R	Y	R	P	G	T
		L1	pS31/K36ac/K37ac	R	K	S	A	P	pS	T	G	G	V	Kac	Kac	P	H	R	Y	R	P	G	T
		L2	K36me/K37me	R	K	S	A	P	S	T	G	G	V	Kme	Kme	P	H	R	Y	R	P	G	T
		L3	K36me2/K37me	R	K	S	A	P	S	T	G	G	V	Kme2	Kme	P	H	R	Y	R	P	G	T
		L4	K36me3/K37me	R	K	S	A	P	S	T	G	G	V	Kme3	Kme	P	H	R	Y	R	P	G	T
		L5	K36ac/K37me	R	K	S	A	P	S	T	G	G	V	Kac	Kme	P	H	R	Y	R	P	G	T
		L6	K36me/K37me2	R	K	S	A	P	S	T	G	G	V	Kme	Kme2	P	H	R	Y	R	P	G	T
		M1	K36me2/K37me2	R	K	S	A	P	S	T	G	G	V	Kme2	Kme2	P	H	R	Y	R	P	G	T
		M2	K36me3/K37me2	R	K	S	A	P	S	T	G	G	V	Kme3	Kme2	P	H	R	Y	R	P	G	T
		M3	K36ac/K37me2	R	K	S	A	P	S	T	G	G	V	Kac	Kme2	P	H	R	Y	R	P	G	T
		M4	K36me/K37me3	R	K	S	A	P	S	T	G	G	V	Kme	Kme3	P	H	R	Y	R	P	G	T
		M5	K36me2/K37me3	R	K	S	A	P	S	T	G	G	V	Kme2	Kme3	P	H	R	Y	R	P	G	T
		M6	K36me3/K37me3	R	K	S	A	P	S	T	G	G	V	Kme3	Kme3	P	H	R	Y	R	P	G	T
		N1	K36ac/K37me3	R	K	S	A	P	S	T	G	G	V	Kac	Kme3	P	H	R	Y	R	P	G	T
		N2	K36me/K37ac	R	K	S	A	P	S	T	G	G	V	Kme	Kac	P	H	R	Y	R	P	G	T
		N3	K36me2/K37ac	R	K	S	A	P	S	T	G	G	V	Kme2	Kac	P	H	R	Y	R	P	G	T
		N4	K36me3/K37ac	R	K	S	A	P	S	T	G	G	V	Kme3	Kac	P	H	R	Y	R	P	G	T
		N5	K36ac/K37ac	R	K	S	A	P	S	T	G	G	V	Kac	Kac	P	H	R	Y	R	P	G	T
		N6	pT32/K36me/K37me	R	K	S	A	P	S	pT	G	G	V	Kme	Kme	P	H	R	Y	R	P	G	T
		O1	pT32/K36me2/K37me	R	K	S	A	P	S	pT	G	G	V	Kme2	Kme	P	H	R	Y	R	P	G	T
		O2	pT32/K36me3/K37me	R	K	S	A	P	S	pT	G	G	V	Kme3	Kme	P	H	R	Y	R	P	G	T

	O3	pT32/K36ac/K37me	R	K	S	A	P	**S**	**pT**	G	G	V	**Kac**	**Kme**	P	H	R	Y	R	P	G	T
	O4	pT32/K36me/K37me2	R	K	S	A	P	**S**	**pT**	G	G	V	**Kme**	**Kme2**	P	H	R	Y	R	P	G	T
	O5	pT32/K36me2/K37me2	R	K	S	A	P	**S**	**pT**	G	G	V	**Kme2**	**Kme2**	P	H	R	Y	R	P	G	T
	O6	pT32/K36me3/K37me2	R	K	S	A	P	**S**	**pT**	G	G	V	**Kme3**	**Kme2**	P	H	R	Y	R	P	G	T
	P1	pT32/K36ac/K37me2	R	K	S	A	P	**S**	**pT**	G	G	V	**Kac**	**Kme2**	P	H	R	Y	R	P	G	T
	P2	pT32/K36me/K37me3	R	K	S	A	P	**S**	**pT**	G	G	V	**Kme**	**Kme3**	P	H	R	Y	R	P	G	T
	P3	pT32/K36me2/K37me3	R	K	S	A	P	**S**	**pT**	G	G	V	**Kme2**	**Kme3**	P	H	R	Y	R	P	G	T
	P4	pT32/K36me3/K37me3	R	K	S	A	P	**S**	**pT**	G	G	V	**Kme3**	**Kme3**	P	H	R	Y	R	P	G	T
	P5	pT32/K36ac/K37me3	R	K	S	A	P	**S**	**pT**	G	G	V	**Kac**	**Kme3**	P	H	R	Y	R	P	G	T
	P6	pT32/K36me/K37ac	R	K	S	A	P	**S**	**pT**	G	G	V	**Kme**	**Kac**	P	H	R	Y	R	P	G	T
	Q1	pT32/K36me2/K37ac	R	K	S	A	P	**S**	**pT**	G	G	V	**Kme2**	**Kac**	P	H	R	Y	R	P	G	T
	Q2	pT32/K36me3/K37ac	R	K	S	A	P	**S**	**pT**	G	G	V	**Kme3**	**Kac**	P	H	R	Y	R	P	G	T
	Q3	pT32/K36ac/K37ac	R	K	S	A	P	**S**	**pT**	G	G	V	**Kac**	**Kac**	P	H	R	Y	R	P	G	T
	Q4	BLANK																				
polyHis CTRL	Q5	HHHHHHHH																				
polyHis CTRL	Q6	HHHHHHHH																				

Histone	Peptide Length	Array Poition	Kac Mark Combinations	Sequence																		
polyHis CTRL		A1	HHHHHHHH																			
polyHis CTRL		A2	HHHHHHHH																			
					22	23				27	28	29		31	32				36			
H3.3C (Q6NXT2)	21-40	B3	CONTROL	A	T	K	A	A	R	K	S	T	P	S	T	C	G	V	K	P	H	R
		B4	K27me	A	T	K	A	A	R	Kme	S	T	P	S	T	C	G	V	K	P	H	R
		B5	K27me2	A	T	K	A	A	R	Kme2	S	T	P	S	T	C	G	V	K	P	H	R
		C1	K27me3	A	T	K	A	A	R	Kme3	S	T	P	S	T	C	G	V	K	P	H	R
		C2	K27ac	A	T	K	A	A	R	Kac	S	T	P	S	T	C	G	V	K	P	H	R
		C3	pS28	A	T	K	A	A	R	K	pS	T	P	S	T	C	G	V	K	P	H	R
		C4	K27me/pS28	A	T	K	A	A	R	Kme	pS	T	P	S	T	C	G	V	K	P	H	R
		C5	K27me2/pS28	A	T	K	A	A	R	Kme2	pS	T	P	S	T	C	G	V	K	P	H	R
		D1	K27me3/pS28	A	T	K	A	A	R	Kme3	pS	T	P	S	T	C	G	V	K	P	H	R
		D2	K27ac/pS28	A	T	K	A	A	R	Kac	pS	T	P	S	T	C	G	V	K	P	H	R
		D3	pT29	A	T	K	A	A	R	K	S	pT	P	S	T	C	G	V	K	P	H	R
		D4	K27me/pT29	A	T	K	A	A	R	Kme	S	pT	P	S	T	C	G	V	K	P	H	R
		D5	K27me2/pT29	A	T	K	A	A	R	Kme2	S	pT	P	S	T	C	G	V	K	P	H	R
		E1	K27me3/pT29	A	T	K	A	A	R	Kme3	S	pT	P	S	T	C	G	V	K	P	H	R
		E2	K27ac/pT29	A	T	K	A	A	R	Kac	S	pT	P	S	T	C	G	V	K	P	H	R
		E3	pS31	A	T	K	A	A	R	K	S	T	P	pS	T	C	G	V	K	P	H	R
		E4	K27me/pS31	A	T	K	A	A	R	Kme	S	T	P	pS	T	C	G	V	K	P	H	R
		E5	K27me2/pS31	A	T	K	A	A	R	Kme2	S	T	P	pS	T	C	G	V	K	P	H	R
		F1	K27me3/pS31	A	T	K	A	A	R	Kme3	S	T	P	pS	T	C	G	V	K	P	H	R
		F2	K27ac/pS31	A	T	K	A	A	R	Kac	S	T	P	pS	T	C	G	V	K	P	H	R
		F3	pT32	A	T	K	A	A	R	K	S	T	P	S	pT	C	G	V	K	P	H	R
		F4	K27me/pT32	A	T	K	A	A	R	Kme	S	T	P	S	pT	C	G	V	K	P	H	R
		F5	K27me2/pT32	A	T	K	A	A	R	Kme2	S	T	P	S	pT	C	G	V	K	P	H	R
		G1	K27me3/pT32	A	T	K	A	A	R	Kme3	S	T	P	S	pT	C	G	V	K	P	H	R
		G2	K27ac/pT32	A	T	K	A	A	R	Kac	S	T	P	S	pT	C	G	V	K	P	H	R
		G3	pS28/pT29	A	T	K	A	A	R	K	pS	pT	P	S	T	C	G	V	K	P	H	R
		G4	pS28/pS31	A	T	K	A	A	R	K	pS	T	P	pS	T	C	G	V	K	P	H	R
		G5	pS28/pT32	A	T	K	A	A	R	K	pS	T	P	S	pT	C	G	V	K	P	H	R
		H1	pT29/pS31	A	T	K	A	A	R	K	S	pT	P	pS	T	C	G	V	K	P	H	R
		H2	pT29/pT32	A	T	K	A	A	R	K	S	pT	P	S	pT	C	G	V	K	P	H	R
		H3	K27me/pS28/pS31	A	T	K	A	A	R	Kme	pS	T	P	pS	T	C	G	V	K	P	H	R
		H4	K27me2/pS28/pS31	A	T	K	A	A	R	Kme	pS	T	P	pS	T	C	G	V	K	P	H	R
		H5	K27me3/pS28/pS31	A	T	K	A	A	R	Kme	pS	T	P	pS	T	C	G	V	K	P	H	R
		I1	K27ac/pS28/pS31	A	T	K	A	A	R	Kme	pS	T	P	pS	T	C	G	V	K	P	H	R
		I2	K27me/pS28/pT32	A	T	K	A	A	R	Kme	pS	T	P	S	pT	C	G	V	K	P	H	R
		I3	K27me2/pS28/pT32	A	T	K	A	A	R	Kme2	pS	T	P	S	pT	C	G	V	K	P	H	R
		I4	K27me3/pS28/pT32	A	T	K	A	A	R	Kme3	pS	T	P	S	pT	C	G	V	K	P	H	R
		I5	K27ac/pS28/pT32	A	T	K	A	A	R	Kac	pS	T	P	S	pT	C	G	V	K	P	H	R

Histone	Peptide Length	Array Poition	Kac Mark Combinations	Sequence																		
					27	28	29		31	32				36								
H3.3C (Q6NXT2)	26-45	J1	CONTROL	R	K	S	T	P	S	T	C	G	V	K	P	H	R	Y	R	P	G	T
		J2	pT29	R	K	S	pT	P	S	T	C	G	V	K	P	H	R	Y	R	P	G	T
		J3	pT29/K36me	R	K	S	pT	P	S	T	C	G	V	Kme	P	H	R	Y	R	P	G	T
		J4	pT29/K36me2	R	K	S	pT	P	S	T	C	G	V	Kme2	P	H	R	Y	R	P	G	T
		J5	pT29/K36me3	R	K	S	pT	P	S	T	C	G	V	Kme3	P	H	R	Y	R	P	G	T
		K1	pT29/K36ac	R	K	S	pT	P	S	T	C	G	V	Kac	P	H	R	Y	R	P	G	T
		K2	pS31	R	K	S	T	P	pS	T	C	G	V	K	P	H	R	Y	R	P	G	T
		K3	pS31/K36me	R	K	S	T	P	pS	T	C	G	V	Kme	P	H	R	Y	R	P	G	T
		K4	pS31/K36me2	R	K	S	T	P	pS	T	C	G	V	Kme2	P	H	R	Y	R	P	G	T
		K5	pS31/K36me3	R	K	S	T	P	pS	T	C	G	V	Kme3	P	H	R	Y	R	P	G	T
		L1	pS31/K36ac	R	K	S	T	P	pS	T	C	G	V	Kac	P	H	R	Y	R	P	G	T
		L2	pT32	R	K	S	T	P	S	pT	C	G	V	K	P	H	R	Y	R	P	G	T
		L3	pT32/K36me	R	K	S	T	P	S	pT	C	G	V	Kme	P	H	R	Y	R	P	G	T
		L4	pT32/K36me2	R	K	S	T	P	S	pT	C	G	V	Kme2	P	H	R	Y	R	P	G	T
		L5	pT32/K36me3	R	K	S	T	P	S	pT	C	G	V	Kme3	P	H	R	Y	R	P	G	T
		M1	pT32/K36ac	R	K	S	T	P	S	pT	C	G	V	Kac	P	H	R	Y	R	P	G	T
		M2	K36me	R	K	S	T	P	S	T	C	G	V	Kme	P	H	R	Y	R	P	G	T
		M3	K36me2	R	K	S	T	P	S	T	C	G	V	Kme2	P	H	R	Y	R	P	G	T
		M4	K36me3	R	K	S	T	P	S	T	C	G	V	Kme3	P	H	R	Y	R	P	G	T
		M5	K36ac	R	K	S	T	P	S	T	C	G	V	Kac	P	H	R	Y	R	P	G	T
		N1	pT29/pS31	R	K	S	pT	P	pS	T	C	G	V	K	P	H	R	Y	R	P	G	T
		N2	pT29/pS31/K36me	R	K	S	pT	P	pS	T	C	G	V	Kme	P	H	R	Y	R	P	G	T
		N3	pT29/pS31/K36me2	R	K	S	pT	P	pS	T	C	G	V	Kme2	P	H	R	Y	R	P	G	T
		N4	pT29/pS31/K36me3	R	K	S	pT	P	pS	T	C	G	V	Kme3	P	H	R	Y	R	P	G	T
		N5	pT29/pS31/K36ac	R	K	S	pT	P	pS	T	C	G	V	Kac	P	H	R	Y	R	P	G	T
		O1	pT29/pT32	R	K	S	pT	P	S	pT	C	G	V	K	P	H	R	Y	R	P	G	T
		O2	pT29/pT32/K36me	R	K	S	pT	P	S	pT	C	G	V	Kme	P	H	R	Y	R	P	G	T
		O3	pT29/pT32/K36me2	R	K	S	pT	P	S	pT	C	G	V	Kme2	P	H	R	Y	R	P	G	T
		O4	pT29/pT32/K36me3	R	K	S	pT	P	S	pT	C	G	V	Kme3	P	H	R	Y	R	P	G	T
		O5	pT29/pT32/K36ac	R	K	S	pT	P	S	pT	C	G	V	Kac	P	H	R	Y	R	P	G	T
		P1	pS31/pT32	R	K	S	T	P	pS	pT	C	G	V	K	P	H	R	Y	R	P	G	T
		P2	pS31/pT32/K36me	R	K	S	T	P	pS	pT	C	G	V	Kme	P	H	R	Y	R	P	G	T
		P3	pS31/pT32/K36me2	R	K	S	T	P	pS	pT	C	G	V	Kme2	P	H	R	Y	R	P	G	T
		P4	pS31/pT32/K36me3	R	K	S	T	P	pS	pT	C	G	V	Kme3	P	H	R	Y	R	P	G	T
		P5	pS31/pT32/K36ac	R	K	S	T	P	pS	pT	C	G	V	Kac	P	H	R	Y	R	P	G	T
polyHis CTRL		Q1	HHHHHHHH																			
polyHis CTRL		Q2	HHHHHHHH																			

Histone	Peptide Length	Array Poition	Kac Mark Combinations	Sequence
polyHis CTRL		A1	HHHHHHHH	
polyHis CTRL		A2	HHHHHHHH	
		A3	Blank	
H4 (P62805)	1-20	A4	CONTROL	S G R G K G G K G L G K G G A K R H R K
		A5	pS1	pS G R G K G G K G L G K G G A K R H R K
		A6	K5me	S G R G X1 G G K G L G K G G A K R H R K
		A7	K5me2	S G R G X2 G G K G L G K G G A K R H R K
		A8	K5me3	S G R G X3 G G K G L G K G G A K R H R K
		A9	K5ac	S G R G X4 G G K G L G K G G A K R H R K
		A10	K8me	S G R G K G G X1 G L G K G G A K R H R K
		A11	K8me2	S G R G K G G X2 G L G K G G A K R H R K
		A12	K8me3	S G R G K G G X3 G L G K G G A K R H R K
		A13	K8ac	S G R G K G G X4 G L G K G G A K R H R K
		A14	K12me	S G R G K G G K G L G X1 G G A K R H R K
		A15	K12me2	S G R G K G G K G L G X2 G G A K R H R K
		A16	K12me3	S G R G K G G K G L G X3 G G A K R H R K
		A17	K12ac	S G R G K G G K G L G X4 G G A K R H R K
		A18	pS1/K5me	pS G R G X1 G G K G L G K G G A K R H R K
		A19	pS1/K5me2	pS G R G X2 G G K G L G K G G A K R H R K
		A20	pS1/K5me3	pS G R G X3 G G K G L G K G G A K R H R K
		A21	pS1/K5ac	pS G R G X4 G G K G L G K G G A K R H R K
		A22	pS1/K8me	pS G R G K G G X1 G L G K G G A K R H R K
		A23	pS1/K8me2	pS G R G K G G X2 G L G K G G A K R H R K
		A24	pS1/K8me3	pS G R G K G G X3 G L G K G G A K R H R K
		A25	pS1/K8ac	pS G R G K G G X4 G L G K G G A K R H R K
		A26	K5me/K8me	S G R G X1 G G X1 G L G K G G A K R H R K
		A27	K5me/K8me2	S G R G X1 G G X2 G L G K G G A K R H R K
		A28	K5me/K8me3	S G R G X1 G G X3 G L G K G G A K R H R K
		A29	K5me/K8ac	S G R G X1 G G X4 G L G K G G A K R H R K
		A30	K5me2/K8me	S G R G X2 G G X1 G L G K G G A K R H R K
		A31	K5me2/K8me2	S G R G X2 G G X2 G L G K G G A K R H R K
		A32	K5me2/K8me3	S G R G X2 G G X3 G L G K G G A K R H R K
		A33	K5me2/K8ac	S G R G X2 G G X4 G L G K G G A K R H R K
		A34	K5me3/K8me	S G R G X3 G G X1 G L G K G G A K R H R K
		A35	K5me3/K8me2	S G R G X3 G G X2 G L G K G G A K R H R K
		A36	K5me3/K8me3	S G R G X3 G G X3 G L G K G G A K R H R K
		A37	K5me3/K8ac	S G R G X3 G G X4 G L G K G G A K R H R K
		B1	K5ac/K8me	S G R G X4 G G X1 G L G K G G A K R H R K
		B2	K5ac/K8me2	S G R G X4 G G X2 G L G K G G A K R H R K
		B3	K5ac/K8me3	S G R G X4 G G X3 G L G K G G A K R H R K
		B4	K5ac/K8ac	S G R G X4 G G X4 G L G K G G A K R H R K
		B5	K8me/K12me	S G R G K G G X1 G L G X1 G G A K R H R K
		B6	K8me/K12me2	S G R G K G G X1 G L G X2 G G A K R H R K
		B7	K8me/K12me3	S G R G K G G X1 G L G X3 G G A K R H R K
		B8	K8me/K12ac	S G R G K G G X1 G L G X4 G G A K R H R K
		B9	K8me2/K12me	S G R G K G G X2 G L G X1 G G A K R H R K
		B10	K8me2/K12me2	S G R G K G G X2 G L G X2 G G A K R H R K
		B11	K8me2/K12me3	S G R G K G G X2 G L G X3 G G A K R H R K
		B12	K8me2/K12ac	S G R G K G G X2 G L G X4 G G A K R H R K
		B13	K8me3/K12me	S G R G K G G X3 G L G X1 G G A K R H R K
		B14	K8me3/K12me2	S G R G K G G X3 G L G X2 G G A K R H R K
		B15	K8me3/K12me3	S G R G K G G X3 G L G X3 G G A K R H R K
		B16	K8me3/K12ac	S G R G K G G X3 G L G X4 G G A K R H R K
		B17	K8ac/K12me	S G R G K G G X4 G L G X1 G G A K R H R K
	6-25	B18	K8ac/K12me2	S G R G K G G X4 G L G X2 G G A K R H R K
		B19	K8ac/K12me3	S G R G K G G X4 G L G X3 G G A K R H R K
		B20	K8ac/K12ac	S G R G K G G X4 G L G X4 G G A K R H R K
		B21	pS1/K5me/K8me	pS G R G X1 G G X1 G L G K G G A K R H R K
		B22	pS1/K5me/K8me2	pS G R G X1 G G X2 G L G K G G A K R H R K
		B23	pS1/K5me/K8me3	pS G R G X1 G G X3 G L G K G G A K R H R K
		B24	pS1/K5me/K8ac	pS G R G X1 G G X4 G L G K G G A K R H R K
		B25	pS1/K5me2/K8me	pS G R G X2 G G X1 G L G K G G A K R H R K
		B26	pS1/K5me2/K8me2	pS G R G X2 G G X2 G L G K G G A K R H R K
		B27	pS1/K5me2/K8me3	pS G R G X2 G G X3 G L G K G G A K R H R K
		B28	pS1/K5me2/K8ac	pS G R G X2 G G X4 G L G K G G A K R H R K
		B29	pS1/K5me3/K8me	pS G R G X3 G G X1 G L G K G G A K R H R K
		B30	pS1/K5me3/K8me2	pS G R G X3 G G X2 G L G K G G A K R H R K
		B31	pS1/K5me3/K8me3	pS G R G X3 G G X3 G L G K G G A K R H R K
		B32	pS1/K5me3/K8ac	pS G R G X3 G G X4 G L G K G G A K R H R K
		B33	pS1/K5ac/K8me	pS G R G X4 G G X1 G L G K G G A K R H R K
		B34	pS1/K5ac/K8me2	pS G R G X4 G G X2 G L G K G G A K R H R K
		B35	pS1/K5ac/K8me3	pS G R G X4 G G X3 G L G K G G A K R H R K
		B36	pS1/K5ac/K8ac	pS G R G X4 G G X4 G L G K G G A K R H R K
		B37	K5ac/K8ac/K12ac	S G R G X4 G G X4 G L G X4 G G A K R H R K
		C1		
		C2	CONTROL	G G K G L G K G G A K R H R K V L R D N
		C3	K12me	G G K G L G X1 G G A K R H R K V L R D N
		C4	K12me2	G G K G L G X2 G G A K R H R K V L R D N
		C5	K12me3	G G K G L G X3 G G A K R H R K V L R D N
		C6	K12ac	G G K G L G X4 G G A K R H R K V L R D N
		C7	K16me	G G K G L G K G G A X1 R H R K V L R D N
		C8	K16me2	G G K G L G K G G A X2 R H R K V L R D N
		C9	K16me3	G G K G L G K G G A X3 R H R K V L R D N
		C10	K16ac	G G K G L G K G G A X4 R H R K V L R D N
		C11	K20me	G G K G L G K G G A K R H R X1 V L R D N
		C12	K20me2	G G K G L G K G G A K R H R X2 V L R D N
		C13	K20me3	G G K G L G K G G A K R H R X3 V L R D N
		C14	K20ac	G G K G L G K G G A K R H R X4 V L R D N
		C15	K12me/K16me	G G K G L G X1 G G A X1 R H R K V L R D N
		C16	K12me/K16me2	G G K G L G X1 G G A X2 R H R K V L R D N
		C17	K12me/K16me3	G G K G L G X1 G G A X3 R H R K V L R D N
		C18	K12me/K16ac	G G K G L G X1 G G A X4 R H R K V L R D N
		C19	K12me2/K16me	G G K G L G X2 G G A X1 R H R K V L R D N
		C20	K12me2/K16me2	G G K G L G X2 G G A X2 R H R K V L R D N
		C21	K12me2/K16me3	G G K G L G X2 G G A X3 R H R K V L R D N
		C22	K12me2/K16ac	G G K G L G X2 G G A X4 R H R K V L R D N
		C23	K12me3/K16me	G G K G L G X3 G G A X1 R H R K V L R D N
		C24	K12me3/K16me2	G G K G L G X3 G G A X2 R H R K V L R D N
		C25	K12me3/K16me3	G G K G L G X3 G G A X3 R H R K V L R D N

**C26**	K12me3/K16ac	G	G	K	G	L	G	X3	G	G	A	X4	R	H	R	K	V	L	R	D	N
**C27**	K12ac/K16me	G	G	K	G	L	G	X4	G	G	A	X1	R	H	R	K	V	L	R	D	N
**C28**	K12ac/K16me2	G	G	K	G	L	G	X4	G	G	A	X2	R	H	R	K	V	L	R	D	N
**C29**	K12ac/K16me3	G	G	K	G	L	G	X4	G	G	A	X3	R	H	R	K	V	L	R	D	N
**C30**	K12ac/K16ac	G	G	K	G	L	G	X4	G	G	A	X4	R	H	R	K	V	L	R	D	N
**C31**	K16me/K20me	G	G	K	G	L	G	K	G	G	A	X1	R	H	R	X1	V	L	R	D	N
**C32**	K16me/K20me2	G	G	K	G	L	G	K	G	G	A	X1	R	H	R	X2	V	L	R	D	N
**C33**	K16me/K20me3	G	G	K	G	L	G	K	G	G	A	X1	R	H	R	X3	V	L	R	D	N
**C34**	K16me/K20ac	G	G	K	G	L	G	K	G	G	A	X1	R	H	R	X4	V	L	R	D	N
**C35**	K16me2/K20me	G	G	K	G	L	G	K	G	G	A	X2	R	H	R	X1	V	L	R	D	N
**C36**	K16me2/K20me2	G	G	K	G	L	G	K	G	G	A	X2	R	H	R	X2	V	L	R	D	N
**C37**	K16me2/K20me3	G	G	K	G	L	G	K	G	G	A	X2	R	H	R	X3	V	L	R	D	N
**D1**	K16me2/K20ac	G	G	K	G	L	G	K	G	G	A	X2	R	H	R	X4	V	L	R	D	N
**D2**	K16me3/K20me	G	G	K	G	L	G	K	G	G	A	X3	R	H	R	X1	V	L	R	D	N
**D3**	K16me3/K20me2	G	G	K	G	L	G	K	G	G	A	X3	R	H	R	X2	V	L	R	D	N
**D4**	K16me3/K20me3	G	G	K	G	L	G	K	G	G	A	X3	R	H	R	X3	V	L	R	D	N
**D5**	K16me3/K20ac	G	G	K	G	L	G	K	G	G	A	X3	R	H	R	X4	V	L	R	D	N
**D6**	K16ac/K20me	G	G	K	G	L	G	K	G	G	A	X4	R	H	R	X1	V	L	R	D	N
**D7**	K16ac/K20me2	G	G	K	G	L	G	K	G	G	A	X4	R	H	R	X2	V	L	R	D	N
**D8**	K16ac/K20me3	G	G	K	G	L	G	K	G	G	A	X4	R	H	R	X3	V	L	R	D	N
**D9**	K16ac/K20ac	G	G	K	G	L	G	K	G	G	A	X4	R	H	R	X4	V	L	R	D	N
**D10**	K12ac/Kl16ac/K20ac	G	G	K	G	L	G	X4	G	G	A	X4	R	H	R	X4	V	L	R	D	N
**D11**																					
**D12**	CONTROL	G	K	G	G	A	K	R	H	R	K	V	L	R	D	N	I	Q	G	I	T
**D13**	K16me	G	K	G	G	A	X1	R	H	R	K	V	L	R	D	N	I	Q	G	I	T
**D14**	K16me2	G	K	G	G	A	X2	R	H	R	K	V	L	R	D	N	I	Q	G	I	T
**D15**	K16me3	G	K	G	G	A	X3	R	H	R	K	V	L	R	D	N	I	Q	G	I	T
**D16**	K16ac	G	K	G	G	A	X4	R	H	R	K	V	L	R	D	N	I	Q	G	I	T
**D17**	K20me	G	K	G	G	A	K	R	H	R	X1	V	L	R	D	N	I	Q	G	I	T
**D18**	K20me2	G	K	G	G	A	K	R	H	R	X2	V	L	R	D	N	I	Q	G	I	T
**D19**	K20me3	G	K	G	G	A	K	R	H	R	X3	V	L	R	D	N	I	Q	G	I	T
**D20**	K20ac	G	K	G	G	A	K	R	H	R	X4	V	L	R	D	N	I	Q	G	I	T
**D21**	K16me/K20me	G	K	G	G	A	X1	R	H	R	X1	V	L	R	D	N	I	Q	G	I	T
**D22**	K16me/K20me2	G	K	G	G	A	X1	R	H	R	X2	V	L	R	D	N	I	Q	G	I	T
**D23**	K16me/K20me3	G	K	G	G	A	X1	R	H	R	X3	V	L	R	D	N	I	Q	G	I	T
**D24**	K16me/K20ac	G	K	G	G	A	X1	R	H	R	X4	V	L	R	D	N	I	Q	G	I	T
**D25**	K16me2/K20me	G	K	G	G	A	X2	R	H	R	X1	V	L	R	D	N	I	Q	G	I	T
**D26**	K16me2/K20me2	G	K	G	G	A	X2	R	H	R	X2	V	L	R	D	N	I	Q	G	I	T
**D27**	K16me2/K20me3	G	K	G	G	A	X2	R	H	R	X3	V	L	R	D	N	I	Q	G	I	T
**D28**	K16me2/K20ac	G	K	G	G	A	X2	R	H	R	X4	V	L	R	D	N	I	Q	G	I	T
**D29**	K16me3/K20me	G	K	G	G	A	X3	R	H	R	X1	V	L	R	D	N	I	Q	G	I	T
**D30**	K16me3/K20me2	G	K	G	G	A	X3	R	H	R	X2	V	L	R	D	N	I	Q	G	I	T
**D31**	K16me3/K20me3	G	K	G	G	A	X3	R	H	R	X3	V	L	R	D	N	I	Q	G	I	T
**D32**	K16me3/K20ac	G	K	G	G	A	X3	R	H	R	X4	V	L	R	D	N	I	Q	G	I	T
**D33**	K16ac/K20me	G	K	G	G	A	X4	R	H	R	X1	V	L	R	D	N	I	Q	G	I	T
**D34**	K16ac/K20me2	G	K	G	G	A	X4	R	H	R	X2	V	L	R	D	N	I	Q	G	I	T
**D35**	K16ac/K20me3	G	K	G	G	A	X4	R	H	R	X3	V	L	R	D	N	I	Q	G	I	T
**D36**	K16ac/K20ac	G	K	G	G	A	X4	R	H	R	X4	V	L	R	D	N	I	Q	G	I	T
**D37**																					
**E1**	CONTROL	K	R	H	R	K	V	L	R	D	N	I	Q	G	I	T	K	P	A	O	R
**E2**	K20me	K	R	H	R	X1	V	L	R	D	N	I	Q	G	I	T	K	P	A	O	R
**E3**	K20me2	K	R	H	R	X2	V	L	R	D	N	I	Q	G	I	T	K	P	A	O	R
**E4**	K20me3	K	R	H	R	X3	V	L	R	D	N	I	Q	G	I	T	K	P	A	O	R
**E5**	K20ac	K	R	H	R	X4	V	L	R	D	N	I	Q	G	I	T	K	P	A	O	R
**E6**																					
**E7**	CONTROL	V	L	R	D	N	I	Q	G	I	T	K	P	A	O	R	E	L	A	R	R
**E8**	pT30	V	L	R	D	N	I	Q	G	I	pT	K	P	A	O	R	E	L	A	R	R
**E9**	K31me	V	L	R	D	N	I	Q	G	I	T	X1	P	A	O	R	E	L	A	R	R
**E10**	K31me2	V	L	R	D	N	I	Q	G	I	T	X2	P	A	O	R	E	L	A	R	R
**E11**	K31me3	V	L	R	D	N	I	Q	G	I	T	X3	P	A	O	R	E	L	A	R	R
**E12**	K31ac	V	L	R	D	N	I	Q	G	I	T	X4	P	A	O	R	E	L	A	R	R
**E13**	pT30/K31me	V	L	R	D	N	I	Q	G	I	pT	X1	P	A	O	R	E	L	A	R	R
**E14**	pT30/K31me2	V	L	R	D	N	I	Q	G	I	pT	X2	P	A	O	R	E	L	A	R	R
**E15**	pT30/K31me3	V	L	R	D	N	I	Q	G	I	pT	X3	P	A	O	R	E	L	A	R	R
**E16**	pT30/K31ac	V	L	R	D	N	I	Q	G	I	pT	X4	P	A	O	R	E	L	A	R	R
