# Supplementary material for: Multivalent Histone and DNA Engagement by a PHD/BRD/PWWP Triple Reader Cassette Recruits ZMYND8 to K14ac-Rich Chromatin
Source: Cell Rep. 2016 Dec 6;17(10):2724–37. doi: 10.1016/j.celrep.2016.11.014 (PMC5177622; doi:10.1016/j.celrep.2016.11.014)
Supplement: Document S2. Article plus Supplemental Information [file mmc4.pdf]

Article

Cell Reports

Multivalent Histone and DNA Engagement by a PHD/BRD/PWWP Triple Reader Cassette Recruits ZMYND8 to K14ac-Rich Chromatin

Graphical Abstract

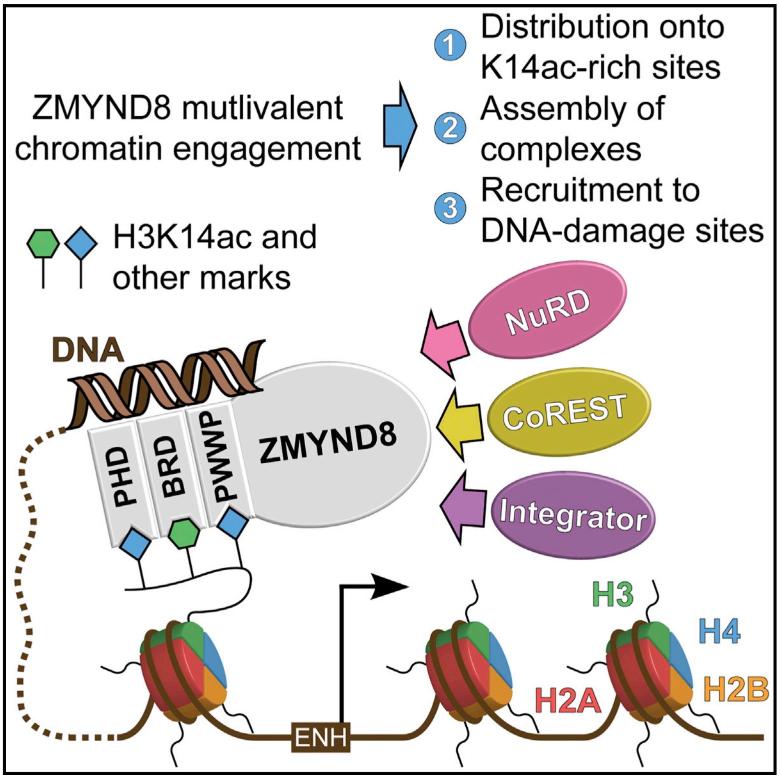

Authors

Pavel Savitsky, Tobias Krojer, Takao Fujisawa, ..., Brian D. Strahl, Anne-Claude Gingras, Panagis Filippakopoulos

Correspondence

panagis.filippakopoulos@sgc.ox.ac.uk

In Brief

Savitsky et al. resolve the crystal structure of the PHD/BRD/PWWP cassette of ZMYND8 and demonstrate that tethering to K14ac-decorated chromatin is mediated through simultaneous engagement of histones and DNA by this triple reader ensemble. Such multivalent interactions control ZMYND8 participation in transcriptional complexes and recruitment to damaged DNA sites.

Highlights

- The ZMYND8 PHD/BRD/PWW cassette forms a stable structural reader ensemble
- The reader ensemble binds chromatin via simultaneous engagement of histones and DNA
- Each module of the ZMYND8 reader ensemble contributes to chromatin interactions
- Genome-wide distribution of ZMYND8 at enhancers is mediated by K14ac interactions

Accession Numbers

4COS
GSE81696

Savitsky et al., 2016, Cell Reports *17*, 2724–2737
December 6, 2016 © 2016 The Author(s).
http://dx.doi.org/10.1016/j.celrep.2016.11.014

CellPress

OPEN ACCESS
CellPress

Cell Reports
Article

Multivalent Histone and DNA Engagement by a PHD/BRD/PWWP Triple Reader Cassette Recruits ZMYND8 to K14ac-Rich Chromatin

Pavel Savitsky,[1,10] Tobias Krojer,[1,10] Takao Fujisawa,[2,10] Jean-Philippe Lambert,[3] Sarah Picaud,[1] Chen-Yi Wang,[2] Erin K. Shanle,[4] Krzysztof Krajewski,[4] Hans Friedrichsen,[2] Alexander Kanapin,[5] Colin Goding,[2] Matthieu Schapira,[6,7] Anastasia Samsonova,[5] Brian D. Strahl,[4,8] Anne-Claude Gingras,[3,9] and Panagis Filippakopoulos[1,2,11,*]
[1]Structural Genomics Consortium, Nuffield Department of Clinical Medicine, University of Oxford, Old Road Campus Research Building, Roosevelt Drive, Oxford OX3 7DQ, UK
[2]Ludwig Institute for Cancer Research, Nuffield Department of Clinical Medicine, University of Oxford, Old Road Campus Research Building Roosevelt Drive, Oxford OX3 7DQ, UK
[3]Lunenfeld-Tanenbaum Research Institute at Mount Sinai Hospital, 600 University Ave., Toronto, ON M5G 1X5, Canada
[4]Department of Biochemistry and Biophysics, University of North Carolina, Chapel Hill, NC 27599, USA
[5]Department of Oncology, University of Oxford, Old Road Campus Research Building, Roosevelt Drive, Oxford OX3 7DQ, UK
[6]Structural Genomics Consortium, MaRS Centre, South Tower, 101 College St., Suite 700, Toronto, ON M5G 1L7 Canada
[7]Department of Pharmacology and Toxicology, University of Toronto, Toronto, ON M5S 1A8, Canada
[8]Lineberger Comprehensive Cancer Center, University of North Carolina, Chapel Hill, NC 27599, USA
[9]Department of Molecular Genetics, University of Toronto, Toronto, ON M5S 1A8, Canada
[10]Co-first author
[11]Lead Contact
*Correspondence: panagis.filippakopoulos@sgc.ox.ac.uk
http://dx.doi.org/10.1016/j.celrep.2016.11.014

SUMMARY

Elucidation of interactions involving DNA and histone post-translational-modifications (PTMs) is essential for providing insights into complex biological functions. Reader assemblies connected by flexible linkages facilitate avidity and increase affinity; however, little is known about the contribution to the recognition process of multiple PTMs because of rigidity in the absence of conformational flexibility. Here, we resolve the crystal structure of the triple reader module (PHD-BRD-PWWP) of ZMYND8, which forms a stable unit capable of simultaneously recognizing multiple histone PTMs while presenting a charged platform for association with DNA. Single domain disruptions destroy the functional network of interactions initiated by ZMYND8, impairing recruitment to sites of DNA damage. Our data establish a proof of principle that rigidity can be compensated by concomitant DNA and histone PTM interactions, maintaining multivalent engagement of transient chromatin states. Thus, our findings demonstrate an important role for rigid multivalent reader modules in nucleosome binding and chromatin function.

INTRODUCTION

Post-translational modifications (PTMs) on histones and DNA are critical regulators of chromatin stability, structure, and gene expression (Schübeler, 2015; Tessarz and Kouzarides, 2014). Combinations of histone PTMs have been recognized to constitute a cellular language, involving deposition, interpretation, and removal of PTMs, referred to as the "histone code" (Strahl and Allis, 2000). Different classes of protein interaction modules ("readers") have evolved to recognize and bind to specific PTMs, including lysine methylation and acetylation, among many known PTMs (Choudhary et al., 2009; Tan et al., 2011). Acetylated lysines (Kac) are recognized by bromodomain modules (BRDs) with affinities ranging in the micromolar range (Filippakopoulos et al., 2012); a single BRD can also bind two adjacent acetylation marks (Filippakopoulos et al., 2012; Morinière et al., 2009), thus enhancing affinity. Recognition of multiple histone PTMs by employing several effector modules present within a given protein confers avidity and increases specificity/affinity as a result of multivalency (Ruthenburg et al., 2007).

Multivalency has been studied in BRD-containing proteins involving two effector modules, often another BRD or a plant homeodomain (PHD). For example, in the case of the transcription initiation factor TFIID subunit 1 (TAF1), two BRD modules exist in a rigidly confined topology, allowing for optimal recognition of multiple acetylated lysines on histone H4 with low micromolar affinity (Jacobson et al., 2000). Multi-domain or paired arrangements including a PHD/BRD cassette provide a platform for combinatorial recognition of lysine methylation and acetylation, introducing higher specificity. For example, the chromatin regulator tripartite motif 24 (TRIM24) binds to the N-terminal H3 tail, simultaneously engaging lysine 4 (H3K4), via a PHD finger, whereas an adjacent BRD module linked via a flexible loop binds to acetylated lysine 23 (H3K23ac) (Tsai et al., 2010). The relative topology between two effector modules affects the type of signals interpreted as a function of the linker connecting the modules that acts as a molecular ruler; for instance, the PHD/BRD

This is an open access article under the CC BY license (http://creativecommons.org/licenses/by/4.0/).

cassette found in BPTF can recognize and bind to histone 3 dimethyl-lysine 4 (H3K4me$_2$) and histone 3 trimethyl-lysine 4 (H3K4me$_3$) (Li et al., 2006) via the PHD domain, facilitating the adjacent BRD to gain specificity for histone 4 lysine 16 acetyl (H4K16ac) found in *trans* within a single nucleosome over other H4 acetylations (Ruthenburg et al., 2011). Therefore, combination of effector modules not only introduces conformational plasticity, leading to specificity, but also introduces avidity, resulting in higher affinities. However, it is not clear whether this is an additive effect so that introduction of additional modules will result in similar enhancements offering specificity toward developing landscapes of PTMs.

To better understand how multivalent interactions involving more than two reader domains impact recognition of PTMs, affecting protein function, we studied the topology of the N-terminal triple reader domain architecture found in the zinc-finger MYND domain-containing protein 8 (ZMYND8), which contains, in addition to a PHD/BRD arrangement, a Pro-Trp-Trp-Pro (PWWP) domain within a PHD/BRD/PWWP cassette. ZMYND8 has been previously found to participate in transcriptional regulation complexes (Eberl et al., 2013; Havugimana et al., 2012; Kloet et al., 2015; Malovannaya et al., 2011), is implicated in gene silencing (Poleshko et al., 2010), acts as a DNA damage response element (Gong et al., 2015), and has recently been proposed to control, together with KDM5C, enhancer activity (Shen et al., 2016). Here we describe the high-resolution crystal structure of the ZMYND8 N-terminal triple reader PHD/BRD/PWWP module and show how contributions from multivalent, simultaneous recognition of DNA and histone PTMs drive ZMYND8 function, affecting recruitment to DNA-damaged sites.

RESULTS

ZMYND8 Triple Reader PHD/BRD/PWWP Modules Form a Stable Unit

Guided by sequence similarity to the recently structurally characterized BRD/PWWP tandem modules of ZMYND11 (Wang et al., 2014; Wen et al., 2014) and by testing expression constructs of varying lengths, we expressed and crystallized the PHD/BRD/PWWP cassette of ZMYND8. The 1.7-Å crystal structure showed that the PHD domain packs next to the BRD domain, whereas a zinc-finger motif linked the PHD/BRD and PWWP modules (Figures 1A and 1B). ZMYND8 contains the conserved asparagine responsible for Kac peptide docking onto BRD modules (Filippakopoulos et al., 2012), a feature missing in the ZMYND11 BRD, which, instead, contains a tyrosine residue and was not found to recognize Kac (Wang et al., 2014; Wen et al., 2014; Figure S1A). The distribution of charge on the surface of the ZMYND8 structure revealed highly charged patches as well as a large hydrophobic area on the back of the PWWP domain (Figure 1C). A C-terminal extension necessary for expression of the three protein modules packed onto this hydrophobic area of the PWWP domain, inserting bulky hydrophobic residues (F360, Y362, F365, and Y369) into pockets formed on the PWWP surface (Figure 1D). The interactions of the C-terminal extension with the PWWP domain explain the necessity for its presence, conferring stability to the triple reader modules. Structural and sequence alignment with ZMYND11 did not reveal any similarities in this region of the protein, suggesting that the observed C-terminal interactions are unique to ZMYND8.

The ZMYND8 Reader Ensemble Provides Structural Clues for Histone Peptide Binding

The similar orientation between the BRD/PWWP modules of ZMYND8 and ZMYND11 as well as the existence of a number of similar residues previously associated with histone isoform-specific interactions of ZMYND11 (Figures S1B and S1C) prompted us to examine whether the reader modules of ZMYND8 provide a structural template for histone peptide recognition. PHD domains recognize and bind to Kme$_x$ sequences found on histones; in the presence of a full aromatic cage, H3K4me$_3$ inserts within the cage while a bulky residue separates the modified lysine from the adjacent arginine (R2), as exemplified in the case of the PHD finger of BPTF bound to a histone H3 K4me$_3$ peptide (Li et al., 2006; Figure S2A). A partial aromatic cage can also accommodate K4me$_3$, as exemplified in the ING2 PHD finger complex with a histone H3 K4me$_3$ peptide (Peña et al., 2006 Figure S2B). In the absence of such a cage, unmodified lysine is accommodated in a shallow groove of the PHD finger formed by a small hydrophobic core flanked by charged residues, as exemplified in the case of the TRIM24 complex with an H3K4 peptide (Tsai et al., 2010; Figure S2C). The architecture of the surface found on the ZMYND8 PHD finger resembles TRIM24, with a partial aromatic wall present (formed by F89) (Figures S2D and S2E). We therefore hypothesized that unmodified or, possibly, singly methylated lysines may be recognized by the ZMYND8 PHD module.

Structural alignment of the ZMYND8 PHD domain with the TRIM33 PHD/BRD tandem module in complex with a methylated histone H3 peptide (histone 3 trimethyl-lysine 3 [H3K9me$_3$]) (Xi et al., 2011) revealed a different orientation of the PHD/BRD modules, suggesting simultaneous engagement of histone peptides with both modules (Figure S2F). In addition, although the TRIM33 topology was shown to be responsible for concurrent engagement of K9me$_3$ and histone 3 lysine 18 acetyl (K18ac) on histone H3, the ZMYND8 topology orients the BRD module closer to histone 3 lysine 14 acetyl (H3K14ac). We therefore hypothesized that simultaneous engagement of histone peptides by the PHD and BRD modules may be possible and that histone 3 lysine 14 acetyl (K14ac) may be a target site of the ZMYND8 BRD module.

Structural overlay of the PWWP domain to the mouse ZMYND11 structure in complex with histone 3 isoform 3 trimethyl lysine 36 (H3.3K36me$_3$) (Wen et al., 2014) revealed a similar arrangement of the BRD/PWWP modules, which are linked by a zinc finger in both proteins (Figure S2G). In addition, an aromatic cage (residues F288, W291, and F307) similar to ZMYND11 was found to be present, suggesting potential binding to methylated peptides. Intriguingly, the C-terminal tail of a crystallographic neighbor was found to be inserted into the cavity of the PWWP cage (Figure S3A). Superimposition of the mouse ZMYND11/histone 3 trimethyl lysine 36 (H3K36me$_3$) complex established that the C-terminal tail residue K396 of ZMYND8 mimics H3K36me$_3$ insertion within the cage, suggesting that ZMYND8 may be able to bind to unmodified lysine residues (Figure S3A).

OPEN
ACCESS
CellPress

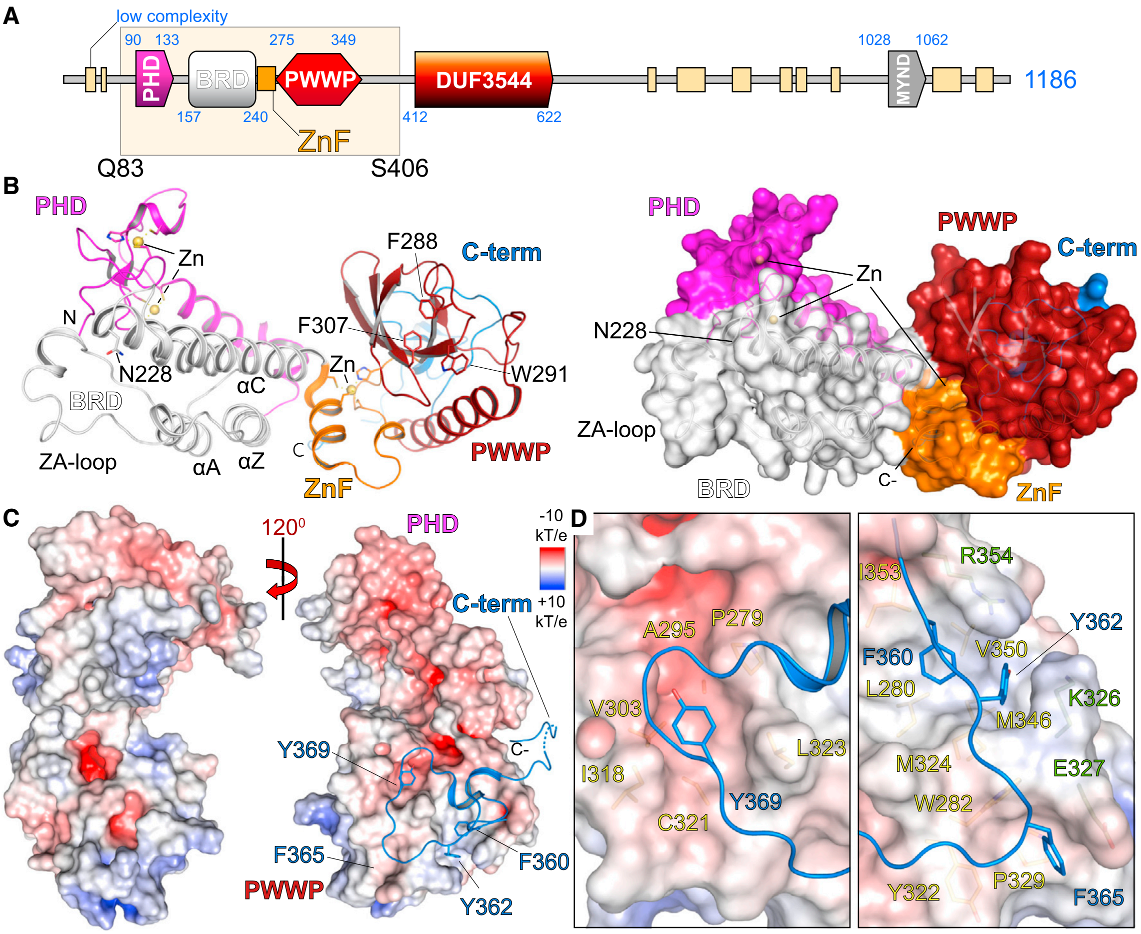

Figure 1. Structure of the ZMYND8 Triple Reader Module

(A) ZMYND8 has a modular architecture, including an N-terminal PHD/BRD/PWWP reader cassette and a C-terminal MYND interaction domain. A recombinant construct expressing residues Q83–S406 was structurally characterized.

(B) The crystal structure of the ZMYND8 reader modules is shown, with major secondary structural elements highlighted on the ribbon (left) and surface (right) models. The conserved Kac peptide docking residue (N228) of the BRD and the aromatic cage of the PWWP domain (F288, W291, and F307) are highlighted. The domain topology is highlighted on the right, with domains individually colored.

(C) Electrostatic distribution of charge displaying the highly charged surface of the protein. Rotation reveals the insertion of key aromatic residues (F360, Y362, F365, and Y369), found on the C-terminal portion of the construct, into the back of the PWWP domain, stabilizing this module.

(D) Detail of the C-terminal tail packing onto the surface of the PWWP domain, revealing three large hydrophobic pockets that accommodate the C-terminal peptide. The first pocket (P279, A285, V303, I318, C321, and L323) accommodates Y369 (left). The second pocket (I353, L280, V350, M324, and M346) is capped by R354 and K326 and accommodates F360 and Y362 (right). The third pocket (W282, Y322, and P329) is capped by E327 and accommodates F365 (right).

Our structural analysis supports the notion that the triple reader assembly of ZMYND8 may be able to engage histone peptides. A schematic of such an possible interaction is shown in Figure S3B.

ZMYND8 Binds to Histone Peptides In Vitro

To establish ZMYND8 interactions with histones, we next tested binding to histone peptides in vitro. Based on our structural analysis, we expected K14ac to engage the BRD module and K36 to engage the PWWP module. The positively charged surface lining the PWWP site toward the zinc finger (ZnF) and BRD modules was shown previously to bind to a phosphate ion in the ZMYND11 structure, in close proximity to T32 of histone H3 (Figure S3A). Threonine 32 has been found to be phosphorylated in a proteomic screen (Kettenbach et al., 2011); however, its biological role remains elusive. We interrogated binding by employing SPOT peptide arrays (Picaud and Filippakopoulos, 2015). Using 20-amino acid (aa)-long histone H3 peptides, we observed binding to most combinations containing a central K14ac modification (Figure 2A; Table S1). Histone H3 peptides spanning residues 22–42 also showed binding to histone H3.3K36 as well as K36me in the presence of pT32 (Figure 2B; Table S1), in agreement with our structural observations. We next systematically profiled histones H2A, H2B, H3, and H4, including their variant isoforms, employing 20-aa-long peptides carrying combinations of Kac, Kme$_x$ (x = 1,2,3), pS, and pT (Figure S4A; Table S2). We observed strong binding to most peptides in the presence of multiple Kac modifications. Histone H3 isoforms as well as H4

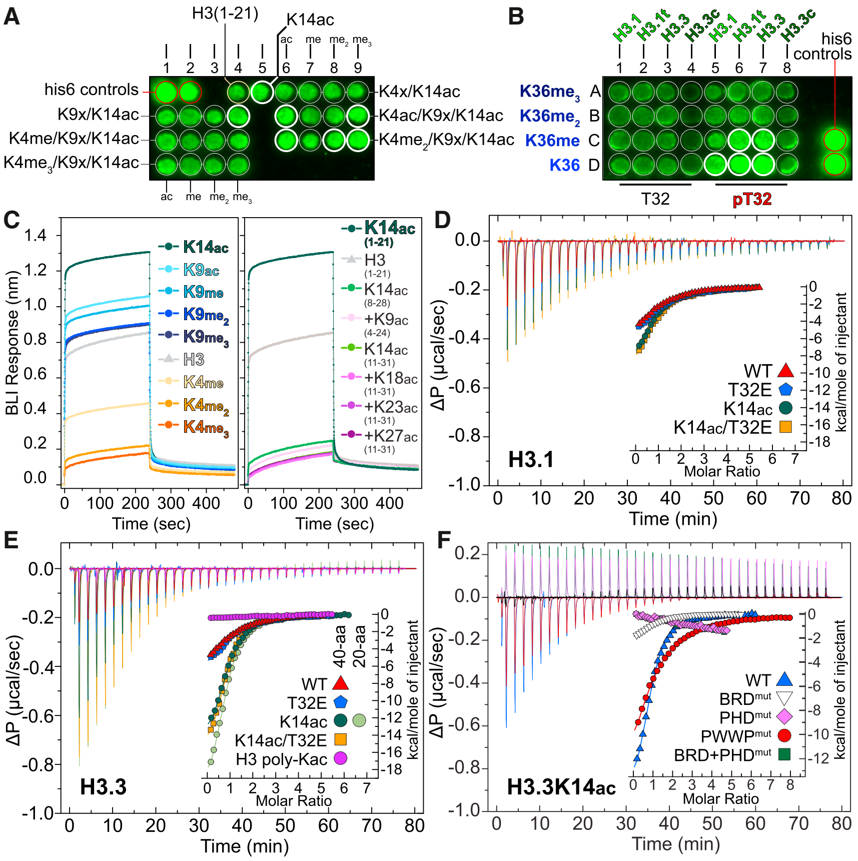

Figure 2. ZMYND8 Binds to Histone H3 In Vitro

(A) Focused peptide SPOT array spanning lysine acetylation/methylation combinations within the first 21 residues of N-terminal histone H3 peptides. The presence of K14ac increases binding intensity (thick white circles).

(B) Focused peptide SPOT array spanning residues 22–42 of all histone H3.x variants, carrying different methyl states of K36, in the absence or presence of pT32.

(C) BLI of the recombinant triple reader ensemble profiled against 20-aa-long N-terminal histone H3 peptides carrying single PTMs as indicated in the inset (left). Unmodified H3 peptides bind to the triple reader modules (shown in gray). K9 modifications are tolerated, whereas K4 methylations break the interaction, and K14 acetylation greatly enhances it. Removal of the N-terminal sequence from peptides results in loss of binding irrespective of additional modifications (K9ac, K18ac, K23ac, and K27ac) present together with the central K14ac mark (right), suggesting that the N-terminal portion of H3, which engages the PHD domain, is essential for binding to ZMYND8. Experiments were carried out twice (n = 2) for each condition/peptide.

(D) In-solution evaluation of histone H3.1 binding by ITC. Raw injection heats for titrations of modified peptides (carrying specific modifications as indicated in the inset) into a solution of ZMYND8 are shown. The inset shows the normalized binding enthalpies corrected for the heat of peptide dilution as a function of binding site saturation (symbols as indicated in the figure). Solid lines represent a nonlinear least-squares fit using a single-site binding model. Histone H3.1 lacking any modifications binds weakly (red triangle, K_D = 19.0 μM), whereas the addition of the phosphomimetic T32E has little effect (blue pentagon, K_D = 20.7 μM). Binding to K14ac is also weak (dark green circles, K_D = 15.1 μM), and the co-existence of the phosphomimetic T32E shows little effect (orange square, K_D = 17.5 μM). ITC titrations were carried out in triplicate (n = 3), and representative curves are shown.

(E) In solution evaluation of histone H3.3. Data are presented as in (D). Histone H3.3 peptides lacking any modifications bind weakly (red triangle, K_D = 19.6 μM), whereas the addition of the phosphomimetic T32E has little effect on binding (blue pentagon, K_D = 17.4 μM). Binding is mainly driven by K14ac (dark and light green circles, K_D = 6.8 μM or 6.6 μM 40-mer and 20-mer, respectively), and the co-existence of the phosphomimetic T32E shows little effect (orange square, K_D = 6.2 μM).

(F) In-solution evaluation of ZMYND8 carrying specific domain mutations to histone H3.3 binding by ITC. Data are presented as in (D). Mutation of the conserved asparagine (N228) on the bromodomain abolishes the interaction, as do mutations of conserved PHD residues (N87A, E104A, and D124A). Combination of PHD/BRD mutations has the same effect. Mutation of the conserved residues forming the PWWP cage (F288A and W291A) result in loss of affinity (WT ZMYND8, 6.8 μM; PWWP mutant, 23 μM).

exhibited higher binding intensities, suggesting stronger binding; however, we could not identify patterns suggestive of specificity toward certain combinations of PTMs.

To assess peptide binding in solution, we used biolayer interferometry (BLI) and profiled commercial libraries containing histone H2A, H2B, H3, and H4 N-terminally biotinylated peptides and found strong binding primarily to H3 and H4 peptides. Although H3K14ac exhibited the strongest signal, we also observed binding to unmodified H3 as well as to all methylation states of K9 (Figure 2C). Interestingly, although unmodified K4 peptides exhibited binding, K4 methylation decreased binding, in agreement with our structural observations (Figure S2A–S2E). Removal of the N-terminal amino acids of H3 resulted in complete loss of binding to all peptides tested irrespective of modifications incorporated (Figure 2C), suggesting that the PHD finger directly engages the N-terminal tail of the histone and is necessary for maintaining peptide interactions.

We next quantified peptide interactions employing BLI titrations and measured a dissociation constant of 27 μM against an H3K14ac peptide (Figure S4B); mutation of the conserved asparagine responsible for BRD/Kac histone interactions (Filippakopoulos et al., 2012) to a phenylalanine (N228F) weakened binding (Figure S4C). We systematically used BLI to interrogate binding to histone H3 modifications employing a set of custom peptides spanning 20 residues along the first 45 residues of each histone tail (Table S3). We observed binding in the presence of multiple Kacs, whereas combinations with Kme_x, other than $K4me_x$, systematically weakened binding (Figure S5). We also tested binding to H4 peptides by BLI and observed weak non-specific binding mainly driven by K12ac (Figure S6A–S6E). Isothermal titration calorimetry (ITC) helped establish precise thermodynamic parameters for peptide binding in solution. We prepared 40-aa-long recombinant H3.1, H3.3, and H4 peptides carrying site-specific acetylations deposited via amber codons

(Neumann et al., 2009) and measured low-micromolar binding for most H3 peptides carrying K14ac, with a small advantage toward H3.3 (6.8 μM) over H3.1 (15.1 μM) sequences (Figures 2D and 2E). Histone H4 peptides showed lower affinity, driven by H4K12ac (31.6 μM) as well as poly-acetylated H4 (37.7 μM) (Figure S6F).

Taken together, our data suggest that ZMYND8 binds via the BRD module to peptides carrying Kac in vitro, whereas the PHD domain is necessary for interactions with the N-terminal tail of these peptides. In addition, ZMYND8 exhibits a slight preference toward H3.3 K14ac-containing peptides over H3.1 or H4 peptides while tolerating a number of adjacent modifications (Tables S4 and S5).

Individual ZMYND8 Reader Domains Contribute to Histone Peptide Binding

Our structural analysis suggested that each of the three ZMYND8 reader modules may contribute to histone binding. Sequence alignments between different organisms showed high conservation of the N-terminal portion of ZMYND8 (Figure S7A). Structural comparison of each ZMYND8 domain to published peptide complexes (Peña et al., 2006; Wang et al., 2014) allowed identifying conserved residues within the PHD (N87, E104, and D124), BRD (N228), and PWWP (F288 and W291) domains, which should affect peptide binding (Figures S7A and S7B).

We tested binding to a 40-residue long histone H3 peptide carrying a K14ac epitope in solution by ITC using recombinant mutant proteins carrying the suggested mutations in each domain. Mutations in the PHD domain (Figures S7A and S7B) abolished peptide binding, as did mutation of the BRD domain (Figures S7A and S7B) or combination of PHD/BRD mutations (Figure 2F; Table S4). Interestingly, although mutation of the BRD resulted in loss of H4K12ac binding, mutation of the PHD had no effect, suggesting that engagement of the H4 tail is primarily initiated via the BRD module (Figures S6D and S6E; Table S5). Mutation of conserved aromatic residues found on the PWWP domain hydrophobic pocket (Figures S7A and S7B) resulted in a dramatic reduction in affinity toward the same peptide (Figure 2F; Table S4). Taken together, these data support a mode of ZMYND8 interaction with histone H3 peptides where all three domains simultaneously engage the first 40 N-terminal histone residues.

ZMYND8 Engages Histones via Its Reader Ensemble in Cells

Having established that ZMYND8 uses the three reader domains to engage histone H3 in vitro, we next asked whether the protein engages histone H3 in cells. We used full-length constructs of ZMYND8 WT or mutated on the BRD module (N228F), PWWP module (F288A/W291A), or all three reader modules (PHD, N87A/E104A/D124A; BRD, N228F; PWWP, F288A/W291A) (Figure 3A). We tested binding to histone H3 in HEK293 cells transfected with 3×FLAG-tagged variants (WT or mutant constructs) by performing immunoprecipitation with anti-FLAG followed by immunoblotting against specific H3 antibodies (Figure 3B). We were surprised to find all histone modifications tested to be enriched in the case of WT ZMYND8, whereas mutations in the BRD or PWWP domains strongly diminished histone binding. Importantly, when all three reader modules were mutated, binding to every histone H3 mark tested was lost. Quantification suggested that BRD mutations primarily affected K9ac/K14ac enrichment, whereas PWWP mutations affected and K36me$_x$ binding (Figures 3C and 3D); mutation of all three reader domains reduced binding by more than 80% toward any H3 mark. Similar results were obtained in the case of H4 binding, where all acetylation marks were bound by the WT protein. Mutations introduced in the BRD module completely abolished H4 interactions, in agreement with our in vitro data, suggesting that H4 binding relies solely on the BRD rather than on all three reader modules of ZMYND8 (Figure 3E).

Taken together, our data suggest that ZMYND8 relies on all three reader modules to engage histone H3 in cells, whereas binding to histone H4 mainly occurs through the BRD module.

ZMYND8 Engages DNA and Chromatin

We found the triple reader ensemble of ZMYND8 engaging histone peptides in vitro and in cells, suggesting that proximity to the nucleosome core might involve interactions with DNA. Previously, ZMYND11 structural features were linked to DNA interactions (Wang et al., 2014; Wen et al., 2014), prompting us to compare the ZMYND8 triple ensemble with the BRD/PWWP ZMYND11 structure (Wang et al., 2014). We found differences in the distribution of charge on the back side of these modules, opposite the aromatic cage responsible for Kme$_x$ binding (Figure 4A). Closer inspection of the two structures revealed only four positively charged patches on ZMYND8, as opposed to five on ZMYND11. A number of charged residues lining up the PWWP aromatic cage (K287, K289, K338, R339, K344, and K345) of ZMYND11 were previously implicated in nonspecific recognition of DNA (Wang et al., 2014). Although most of these residues are present in ZMYND8 (K284, K286, K334, and K336) and structurally align between proteins, the K344/K345 motif found in ZMYND11 is replaced by N340/S341 in ZMYND8 (Figure 4B; Figure S1A). In addition, the K338/R339 patch found in ZMYND11 is located in a loop region connecting β8 and α4 in the PWWP domain; however, the C-terminal PWWP loop extension of ZMYND8 forces this loop region to distort compared to ZMYND11, resulting in repositioning of K334/K336, thus forming a small extension of helix α4. This topology suggests that the α4 "face" of the human ZMYND8 PWWP domain may not be able to engage DNA. Repositioning of the loop between β8 and α4 results in K338 (ZMYND8) overlaying with K334 (ZMYND11), suggesting that this loop maintains contacts with DNA, as does the loop between β3 and β4 (K287/K289 in ZMYND8 and K284/K286 in ZMYND11).

In addition to the positively charged PWWP face, we identified positively charged patches on the surface of the BRD and PHD domains (Figure 4C). Systematic mutation of conserved residues on each interface (annotated in Figure S7A; PHD/BRD, R96A/K233A/K239A/K243A; PWWP, K284A/K286A/K334A) allowed testing the ability of recombinant constructs to interact with DNA in Electrophoretic Mobility Shift Assays (EMSA) (Garner and Revzin, 1981). Although the WT protein was able to shift AT-rich and GC-rich radioactive oligonucleotide probes, the two DNA face mutants lost their ability to interact with DNA

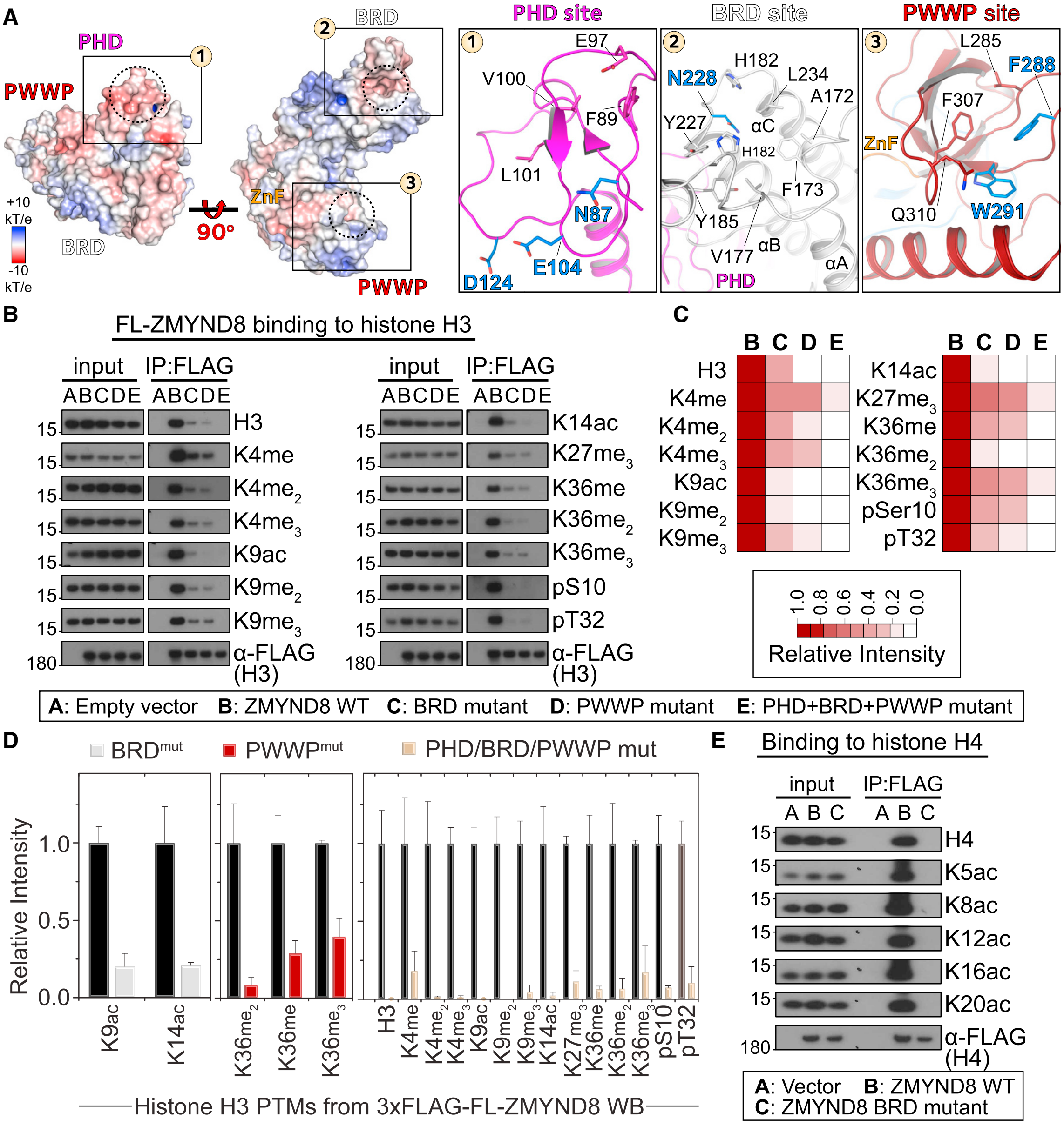

Figure 3. ZMYND8 Binds to Histone H3 in Cells through Its Reader Domains

(A) Surface representation of the electrostatic potential distribution onto the triple reader ZMYND8 ensemble (scale as indicated in the inset), highlighting potential docking sites of histone peptides spanning the PHD, BRD, and PWWP domains (see also Figures S7A and S7B). Dotted circles highlight the cavities where histone residues would insert. Details shown on the right highlight the residues implicated in peptide binding as well as critical residues (highlighted in blue) that were further mutated to probe ZMYND8-histone interactions.

(B) HEK293 cells transiently transfected with 3×FLAG-ZMYND8 WT or mutants (BRD mutant, N228F; PWWP mutant, F288A/W291A; PHD+BRD+PWWP mutant, N87A/E104A/D124A/N228F and F288A/W291A) were used to interrogate binding to histone H3. The WT protein recognizes all marks tested, whereas mutations on the interacting interface of the three modules result in gradual loss of binding. Mutated residues are annotated in (A).

(C) Western blot quantification demonstrating the effect of ZMYND8 mutations on recognition of histone H3 marks from (B). Relative intensity is as annotated in the inset.

(D) Quantification of the effect of BRD mutation (left), PWWP mutations (center), or PHD/BRD/PWWP mutations (right) on histone H3 marks. Although BRD mutations primarily affect recognition of acetylation at H3K9/K14ac and PWWP mutations affect H3K36me$_x$ states, simultaneous mutation of all three reader modules results in more than 85% loss of histone H3 binding for all marks tested. Data represent mean ± SEM from three biological replicates of the WBs shown in (B).

(E) HEK293 cells transiently transfected with 3×FLAG-ZMYND8 WT or BRD mutant (N228F) were used to interrogate binding to histone H4 marks. Acetylation of H4 can be recognized by the protein, but mutation of the BRD module results in total loss of the interaction.

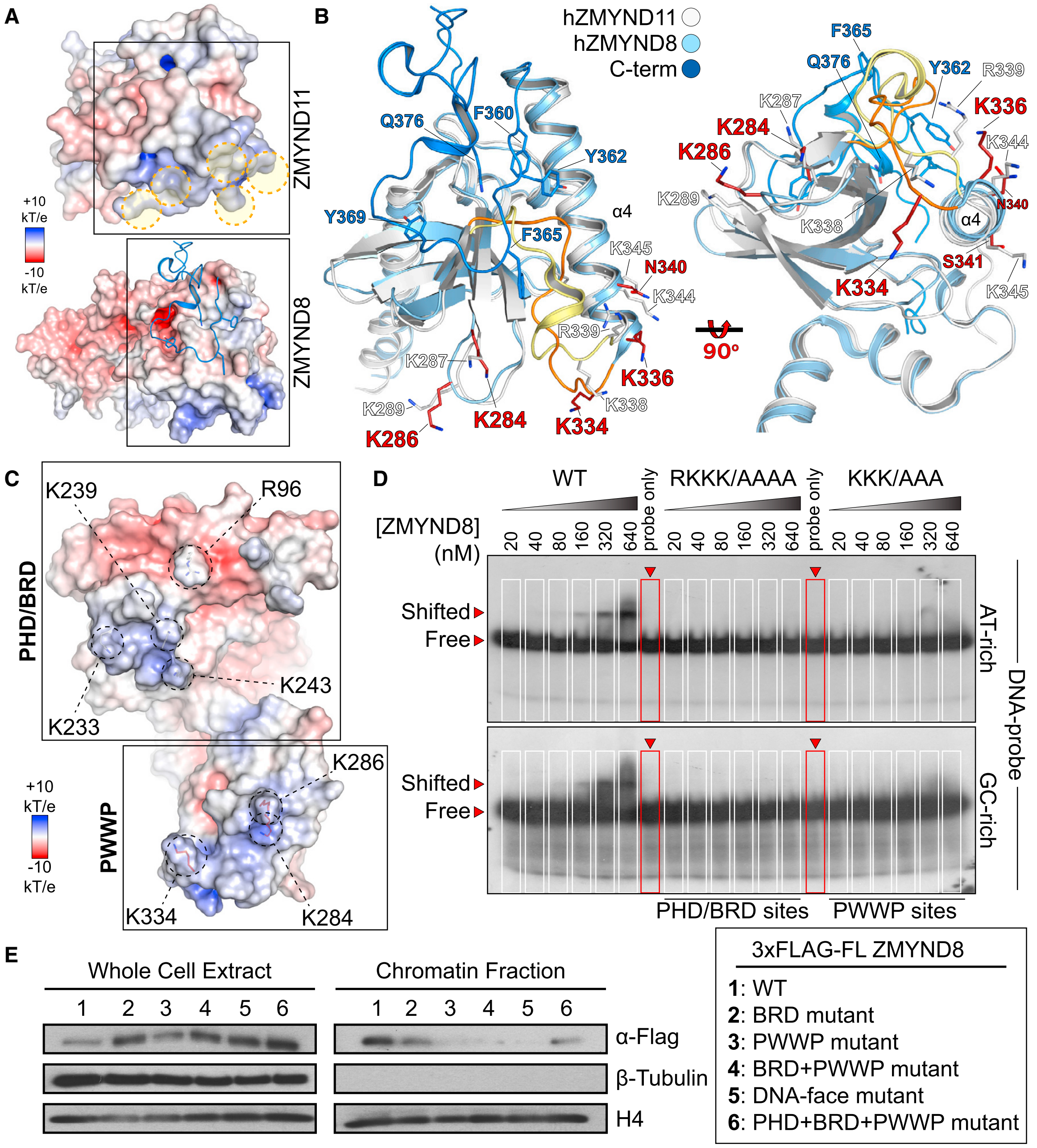

Figure 4. ZMYND8 Binds to DNA and Nucleosomes

(A) Detail of the PWWP domains of human ZMYND11 (PDB: 4NS5) and ZMYND8, showing positively charged areas (highlighted with orange dotted circles). Structures are oriented so that the bottom of the PWWP domain is visible, with residue electrostatic potential plotted on the surface of each protein, as indicated in the inset.

(B) Detail of the boxed regions shown in (A) in ribbon representation, as indicated in the inset, highlighting the structural implications of the C-terminal portion of the hZMYND8 structure. The loop region connecting β8 and α4 in the PWWP domain (hZMYND11, orange; hZMYND8, pale yellow) adopts a helical turn in hZMYND8 as a result of the C-terminus packing under the PWWP domain via aromatic residues (Y362, F365, and Y369 shown in blue), resulting in a small extension of helix α4. Repositioning of the loop between β8 and α4 results in K338 (in hZMYND8) overlaying with K334 (in hZMYND11), and the loop between β3 and β4 (K287/K289 in hZMYND8 and K284/K286 in hZMYND11) suggests a role in maintaining contacts with DNA. Rotation of the superimposed structures by 90° (right) reveals a different orientation of the positive residues in each structure, suggesting different topologies when interacting with nucleosomes.

(C) DNA binding interfaces identified on the PHD/BRD modules (R96, K233, K239, and K243) and PWWP module (K284, K286, and K334) mapped onto the charged surface of the reader ensemble (scale as indicated in the inset).

(legend continued on next page)

(Figure 4D). Importantly, mutation of the two DNA-face interfaces in the full-length protein abrogated interactions with all H3 and H4 PTMs in HEK293 cells (Figures S8A and S8B); however, mutation of the histone-binding interfaces within each domain did not affect binding to DNA in EMSA assays (Figure S8C).

To verify that ZMYND8 simultaneously interacts with histones and DNA, directly engaging nucleosomes, we employed a chromatin fractionation assay (Rothbart et al., 2012a) using HEK293 cells transfected with full-length 3×FLAG-tagged ZMYND8, WT, or mutants that abolish histone or DNA interactions. Western blot (WB) analysis of the chromatin-associated fraction by anti-FLAG revealed a strong signal for the WT protein; mutations of the reader domains affecting histone binding or of the DNA-binding face of the protein resulted in dramatic reduction or loss of ZMYND8 in the chromatin-associated fraction. Taken together, our data suggest that ZMYND8 is tethered to chromatin, initiating multivalent interactions resulting in combinatorial readout of histones and DNA.

ZMYND8 Co-localizes with H3K14ac at Transcriptional Start Sites and Enhancers

We next investigated the genome-wide distribution of ZMYND8 in HEK293 cells together with H3K14ac, which we found to be the most potent determinant of histone binding in vitro, employing chromatin immunoprecipitation followed by sequencing (ChIP-seq). We found ZMYND8 signals enriched around transcriptional start sites (TSSs) together with H3K14ac (Figures 5A and 5B), and tag density analysis identified 39.1% significant overlap with H3K14ac signals at enhancer elements (Figure 5C; Figures S9A and S9B). ZMYND8 signals also correlated well with a recently reported dataset in breast ZR-75-30 cells (Shen et al., 2016) at enhancers (Figures S9C and S9D), and ZMYND8 occupancy around TSSs was shown in Genome Browser Tracks (Figure 5D). Small interfering RNA (siRNA) depletion of histone acetyl-transferases controlling K14ac (P300, PCAF, and ADA2a) (Feller et al., 2015; Jin et al., 2011; Nagy et al., 2010) reduced protein levels of this mark (Figure S9E) and showed loss of occupancy at the TSS of genes, resulting in reduction of ZMYND8 at the same loci, albeit at variable levels (Figure S9F). However, small interference RNA targeting an histone acetyltransferase (siHAT) also resulted in changes of methylation states of H3, making it difficult to directly assign a role for K14ac-specific recruitment of ZMYND8.

To better understand K14ac contributions to ZMYND8 localization, we engineered a "K14ac-masking" system, employing the BRD of BAZ2B, which we found previously to preferentially recognize H3K14ac in vitro (Filippakopoulos et al., 2012). Using SPOT assays, we first confirmed binding only to K14ac and measured low micromolar affinity (Table S6) in solution by ITC (Figures S10A and S10B). Mutation of the BAZ2B conserved asparagine (N2140) resulted in complete loss of binding in solution (Figures S10B and S10C). Constructs carrying three BAZ2B BRD modules were able to pull H3K14ac from HEK293 cells, whereas introduction of the N2140F mutation abolished this interaction (Figure S10C). We employed a fluorescent recovery after photobleaching (FRAP) technique to disrupt the interaction of mCherry-tagged 3×BAZ2B (WT or N/F mutant) with H3K14ac and observed displacement of the mutant protein from chromatin, as evidenced by the fast recovery after bleaching (Figures S10D and S10E), whereas the WT protein did not accumulate at the laser-damaged site (Figure S10F). Taken together, these data established the BAZ2B 3×BRD system as a mask for H3K14ac in cells. We next used this K14ac-masking tool in a U2OS Flp-In/TRex system stably expressing GFP-ZMYND8 using a FRAP assay to displace ZMYND8 from chromatin. In the presence of BAZ2B^{WT}, we observed fast recovery after bleaching, suggesting removal of ZMYND8 from chromatin, whereas the mutant BAZ2B^{NF} masking did not have this effect (Figure S10G; Figure 5E), thus establishing that K14ac masking results in higher mobility of ZMYND8. We next performed ChIP assays at the loci shown previously to be affected by siHAT depletion of K14ac. In agreement with the FRAP data, BAZ2B^{WT} resulted in loss of the ZMYND8 ChIP signal, whereas BAZ2B^{NF} did not have an effect (Figure 5F). Taken together, our data suggest that H3K14ac is a key determinant for ZMYND8 recruitment to chromatin, in agreement with our in vitro and in-cell observations.

ZMYND8 Recruitment to DNA-Damaged Sites Is Controlled by Histone and DNA Interactions

ZMYND8 has been shown to act as a DNA damage response protein, recruited to DNA-damaged sites as a function of its BRD interaction with histone H4 (Gong et al., 2015). We next tested the contribution of each reader module of ZMYND8 to recruitment onto damaged DNA sites. We employed a FRAP assay in U2OS cells transiently transfected with full-length GFP-ZMYND8^{WT} or mutants. ZMYND8^{WT} was actively recruited to a bleached nuclear region after ~60 s; however, mutations systematically introduced to the reader ensemble gradually reduced recruitment to the DNA-damaged sites (Figures 6A and 6B), suggesting that each reader module contributes to the recruitment process. Introduction of mutations on the DNA-face sites of ZMYND8 (Figure 6C) resulted in a similar loss of recruitment onto damaged sites, suggesting that both histone- and DNA-initiated interactions are necessary for ZMYND8 interactions with these sites (Figures 6D and 6E).

Loss of Reader Function Perturbs the ZMYND8 Interactome

We next asked whether multivalent interactions are also responsible for ZMYND8 participation within protein interaction networks through chromatin associations. First, we established the broader ZMYND8 interactome using two orthogonal approaches, affinity purification (AP) and proximity biotinylation

(D) The recombinant WT reader ensemble can shift a radioactive AT-rich (top) or GC-rich (bottom) DNA probe. Alanine mutations (guided by the model shown in A) introduced on the PHD/BRD (RKKK/AAAA) or the PWWP (KKK/AAA) interface abolish DNA interactions in EMSAs.

(E) Full-length ZMYND8 associates with chromatin, whereas mutations of its readers or its DNA-binding face result in loss of binding to chromatin. Full-length 3×FLAG ZMYND8 WT or mutants (as indicated in the inset) were transiently transfected into HEK293 cells, and the whole-cell extract and chromatin-associated fraction were analyzed for 3×FLAG-ZMYND8 by western blot.

OPEN ACCESS
CellPress

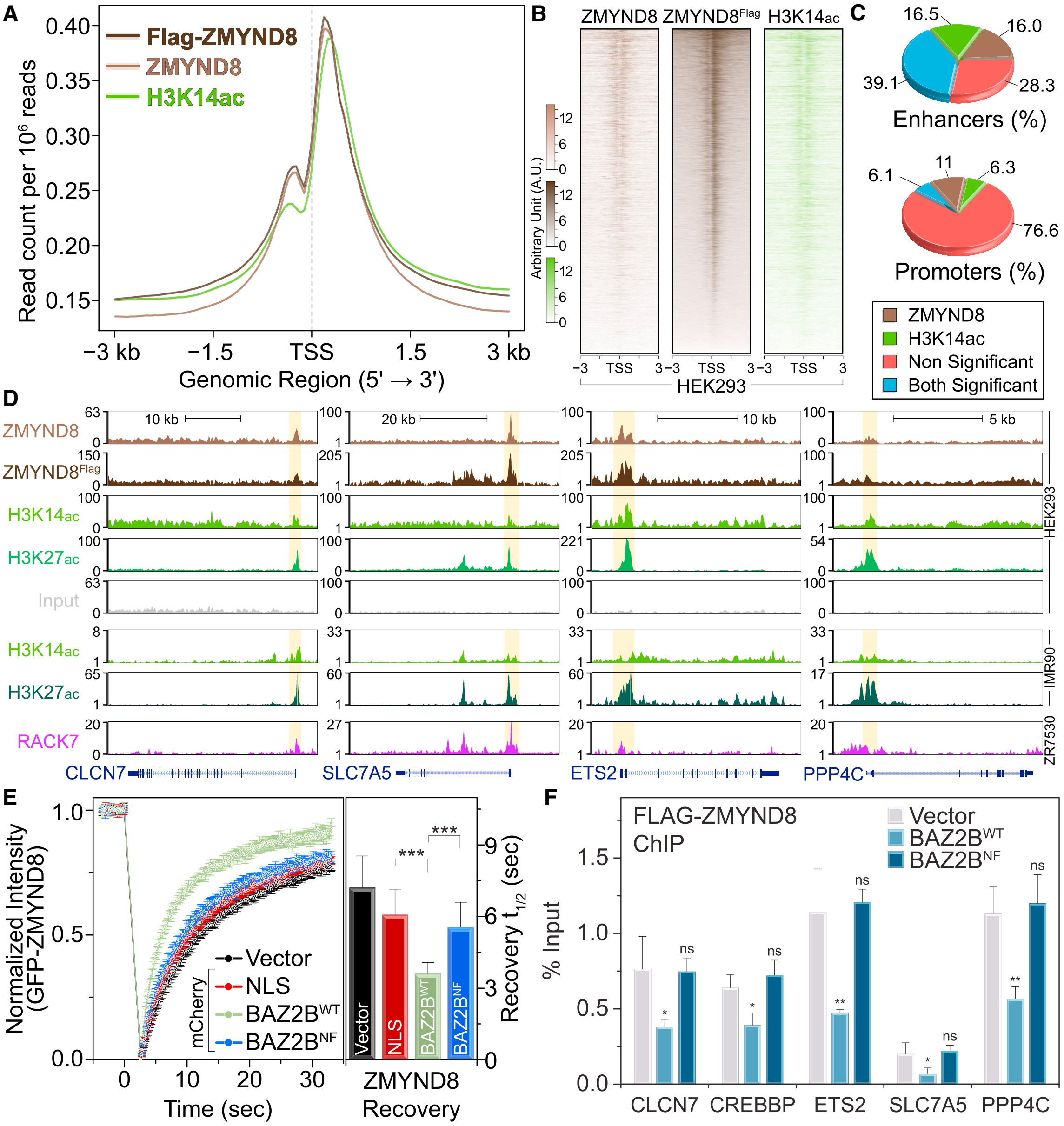

Figure 5. ZMYND8 Co-localizes with H3K14ac at Transcriptional Start Sites and Enhancers

(A) Average profile of ZMYND8, FLAG-ZMYND8, and H3K14ac ChIP-seq signals on ± 3 kb around transcriptional start sites in HEK293 cells. Representative distributions from biological replicates (n = 2) are shown.

(B) Heatmaps of ChIP-seq signals on ± 3 kb surrounding the TSS of genes bound to ZMYND8 or FLAG-ZMYND8 and H3K14ac in HEK293 cells.

(C) Distribution of tag densities for ZMYND8 and K14ac around enhancer (top) and promoter (bottom) regions showing significant overlap according to p values calculated relative to local background.

(D) ChIP-seq analysis of ZMYND8 and H3K14ac in HEK293 cells for selected genes. Signal enrichment is compared with the published enrichment signals for H3K14ac (GSM521883) and H3K27ac (GSM521887) in IMR90 cells as well as ZMYND8 in ZR-75-30 cells (GSE71323).

(E) FRAP experiments following masking of H3K14ac in U2OS cells by transfection with mCherry-3xBAZ2B (WT or N/F mutant) constructs. Competition for H3K14ac by BAZ2B^{WT} results in faster recovery of ZMYND8, whereas the inactive BAZ2B$^{N/F}$ mutant has no effect on ZMYND8 recovery, demonstrating direct competition for K14ac in cells. Data represent the mean ± SEM (n = 15). Right: quantitative comparison of time with half-maximal fluorescence recovery for GFP-ZMYND8. ***p < 0.005.

(F) ChIP-qPCR validation of FLAG-ZMYND8 over K14ac-bound loci in HEK293 cells stably expressing 3×FLAG-ZMYND8^{WT} following competition with HA-3xBAZ2B^{WT} or HA-3xBAZ2B^{NF}. Masking of H3K14ac by HA-3xBAZ2B^{WT} resulted in significant reduction of ZMYND8 occupancy at these loci. Data represent mean ± SD from biological triplicates (n = 3). **p < 0.01, *p < 0.05. ns, not significant.

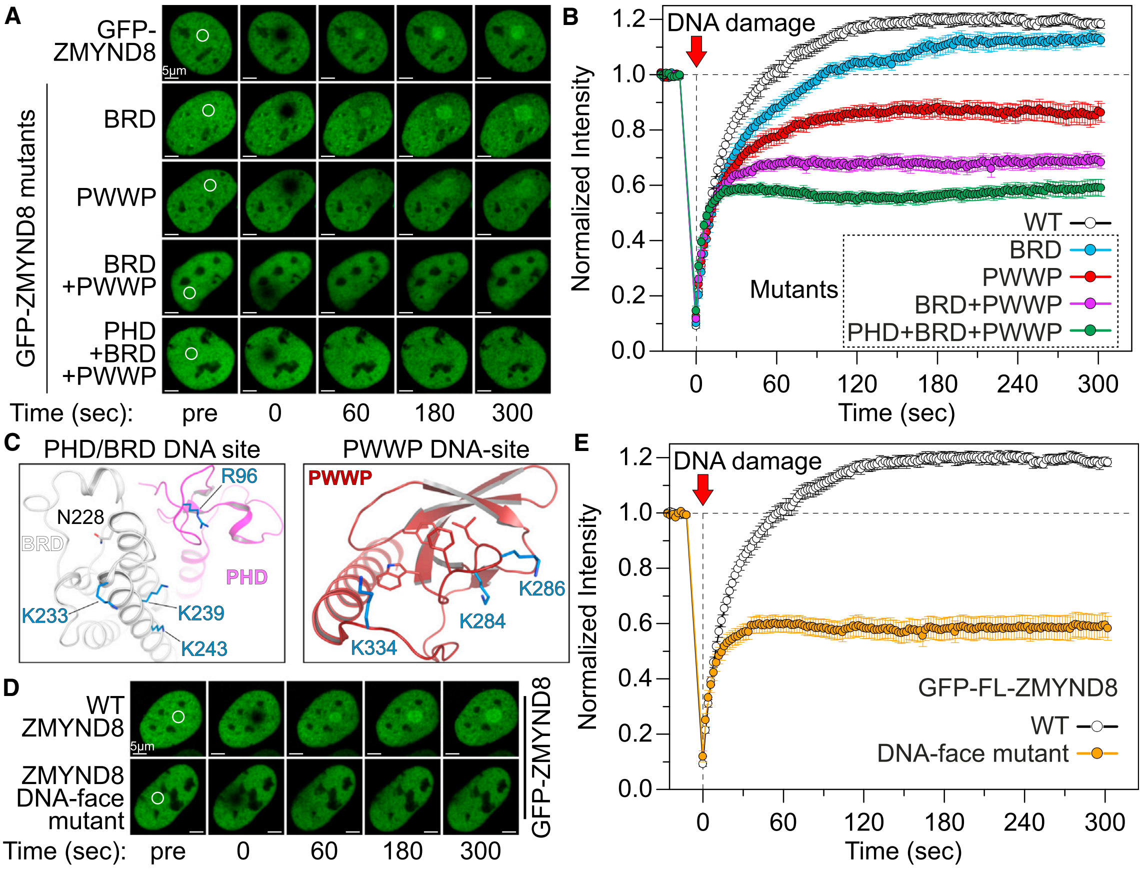

Figure 6. ZMYND8 Reader Interactions Control Recruitment to DNA Damage Sites

(A and B) U2OS cells transfected with GFP-tagged ZMYND8 WT or mutants were photobleached (small area annotated with a white circle) in FRAP experiments (A). The WT protein is rapidly recruited to the bleached site, whereas mutations of the reader modules result in gradual loss of the protein's ability to recruit onto the damaged site (B).

(C) Detail of the PHD/BRD (left) and PWWP (right) interfaces highlighting residues that can potentially bind to DNA.

(D and E) U2OS cells transfected with GFP-tagged ZMYND8 WT or DNA-face mutant (involving simultaneous mutation of the seven residues highlighted in C) were photobleached in FRAP experiments (D). The WT protein is rapidly recruited to the bleached site, whereas mutation of DNA-interacting residues results in significant loss of the proteins ability to recruit onto the damaged site (E).

The data in (B) and (E) represent the mean ± SEM from multiple experiments in different cells (n = 15).

(BioID) coupled to mass spectrometry (MS) (Lambert et al., 2015). Using both AP-MS (Figure 7A) and BioID-MS (Figure 7B), we found ZMYND8 to interact with components of transcriptional complexes, including the nucleosome remodeling and de-acetylase complex (NuRD), the co-repressor of the RE1-silencing transcription factor complex (CoREST), as well as the integrator complex. Mutation of the BRD module resulted in weaker interactions with these complexes, as exemplified by the lower number of peptides identified (Figures 7A and 7B). We also observed gain of interactions (particularly with BioID) with several new components that did not seem to be functionally linked.

Functional enrichment analysis followed by annotation and clustering of the AP-MS network components confirmed a systematic loss of interactions with chromatin-associated functions upon mutation of the BRD domain for all statistically significant biological processes (Figure S11A) and cellular components (Figure S11B). Similarly, loss of enrichment was observed in biological processes enriched by the BioID set of genes (Figure S11C), whereas there was gain of function toward non-chromatin-related cellular components, as exemplified by interactions with mitochondrial proteins (Figure S11D), suggesting that disruption of the chromatin association of ZMYND8 may be affecting its sub-cellular localization and its interaction network.

DISCUSSION

It has been proposed that multivalent binding results in higher specificity accompanied by affinity enhancement and loss of translational/rotation freedom to gain favorable entropy (Ruthenburg et al., 2007). Interactions with modified histone termini are usually weak (100–300 μM); however, reader modules have evolved to simultaneously engage multiple PTMs, resulting in single-digit micromolar interactions (Filippakopoulos et al., 2012; Morinière et al., 2009). Combination of multiple reader

OPEN
ACCESS
CellPress

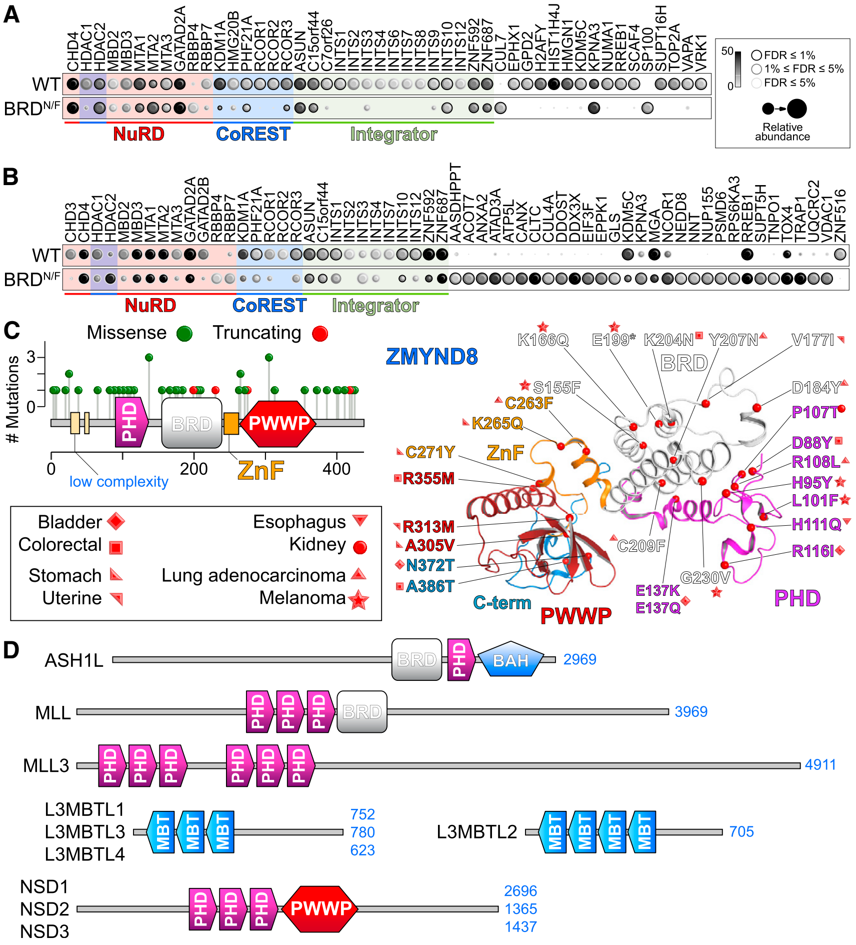

Figure 7. ZMYND8 Reader Interactions Control the Assembly of Transcriptional Complexes

(A) AP-MS of full-length 3×FLAG WT or BRD$^{N/F}$ ZMYND8 from HEK293 cells highlighting the changes in the ZMYND8 interactome following disruption of binding to acetylated histones by mutating the conserved BRD asparagine (N228) to a phenylalanine. The data represent the mean of biological replicate experiments (n = 2). Spectral counts of each prey proteins are displayed as a gray scale in the legend.

(B) Proximity biotinylation coupled to mass spectrometry of full-length BirA*-FLAG WT (top) or BRD mutant (bottom) ZMYND8 in HEK293 cells. Mutation of the BRD module results in partial loss of enrichment of many components found to interact by AP/MS as well as gain of interaction with several new proteins. The data represent the mean of biological replicate experiments (n = 2).

(C) Human ZMYND8 mutations and expression in cancer. Mutations annotated by the TCGA consortium found on the triple reader modules of ZMYND8 are annotated on the structure, colored according to the domain to which they belong, and classified by cancer type.

(D) Domain organization of proteins that contain multiple reader domains in serial arrangements, potentially adopting multivalent assemblies that can engage distinct histone or nucleosome states.

modules evolved to confer avidity and further specificity, resulting in single- to double-digit micromolar affinities, as exemplified by interactions of tandem BRDs in TAF1 (Jacobson et al., 2000) or tandem PHD/BRD modules in TRIMs (Ruthenburg et al., 2011; Tsai et al., 2010; Xi et al., 2011). Direct interactions with DNA have also evolved and are usually found in larger complexes, such as the regulator of chromosome condensation (RCC1), which directly binds nucleosomal DNA (Makde et al., 2010), the RPC1 ubiquitination module, which binds directly to the core nucleosome (McGinty et al., 2014), or LSD1-CoREST, which engages the nucleosome core between two turns of the DNA superhelix (Yang et al., 2006).

Here we report the structure of the triple N-terminal reader modules found in ZMYND8, which form a stable ensemble capable of simultaneously engaging histones and DNA. Binding is mainly driven by H3K14ac in vitro, and we find ZMYND8 to be associated with this mark in cells, whereas other histone modifications seem to be tolerated, with the exception of higher lysine methylation states. Two recent studies suggested that H4K16ac is a main point of histone H4 contact based on antibody evidence (Adhikary et al., 2016; Gong et al., 2015); however, our in vitro data clearly show that H4K12ac is the main driver of this interaction, which is much weaker than H3K14ac, whereas our antibody staining showed enrichment of all H4 acetyl states tested. Masking of H3K14ac using multiple bromodomains of BAZ2B destabilized the chromatin complex of ZMYND8, as evidenced by shorter recovery times in FRAP experiments, while removing the protein from K14ac-rich loci, as evidenced by ChIP-PCR assays.

Interaction with the entire N-terminal histone tail may result in "shielding," preventing remodeling, a topology that we find by molecular modeling to be possible (see Supplemental Information and Figure S12); we also find enzymes such as HDAC1, KDM1, and KDM5C participating in the ZMYND8 interaction network, which can potentially maintain the PTM landscape around ZMYND8 binding sites. Indeed, a recent study demonstrated that the demethylase KDM5C maintains a H3K4me1 state when recruited by ZMYND8 at its target enhancers (Shen et al., 2016). Perturbation of any of the interacting interfaces of the ZMYND8 reader ensemble does not completely dissociate the protein from chromatin, possibly allowing the system to return to an equilibrium state. However, simultaneous disruption of multiple points of contact (e.g., deacetylation of K14, methylation of K4) may result in dissociation from chromatin and destabilization of the ZMYND8 intricate network of interactions, possibly resulting in protein relocation. Indeed, in agreement with previous reports, we found ZMYND8 to be associated with tightly regulated machinery, including the CoREST and integrator complexes (Malovannaya et al., 2011) as well as the NuRD complex (Eberl et al., 2013; Havugimana

et al., 2012; Kloet et al., 2015). It was reported previously that ZMYND8 is recruited to DNA damage sites through the NuRD component CHD4 (Gong et al., 2015). Here we show that perturbation of the reader functions of ZMYND8 severely reduces its ability to recruit to DNA in response to DNA damage. Interestingly, we found that, although H4 interactions are abolished when the BRD is mutated, H3 interactions are only weakened but not lost, suggesting a different mode of histone tail engagement between histones.

Intriguingly, inspection of data reported by The Cancer Genome Atlas (TCGA) consortium (extracted from cBioPortal in November 2015; Gao et al., 2013; http://www.cbioportal.org/) revealed that 25% of ZMYND8 mutations occur within the reader ensemble, with enriched sites on the PHD and PWWP modules. Mapping of the mutated sites onto the crystal structure suggests that most, if not all, affect binding to histones either by disrupting the interaction interface or by destabilizing the domain topology (Figure 7C).

Our work provides a proof of concept linking structural rigidity with simultaneous DNA and histone interactions to maintain recognition of chromatin states in the absence of conformational flexibility. The relative weak binding exhibited by ZMYND8 could result in the maintenance of a transient binding state to chromatin, allowing for early association with establishing patterns of PTMs during chromatin landscape remodeling. The conformational rigidity present in the ZMYND8 triple reader ensemble could offer protection against random changes in the same PTM landscape, resulting in deposition or removal of marks that would destabilize or alter transcriptional programs. However, several other proteins contain multi-reader organizations, potentially offering topologies similar to the ZMYND8 triple reader ensemble or allowing reassembly in three dimensional clusters that offers similar characteristics (Figure 7D). More work is needed to define the characteristics of chromatin engagement as a function of plasticity involving multivalent protein assemblies, seeking to identify shared and distinct mechanisms that help relay integrated signaling cascades leading to specific transcriptional programs.

EXPERIMENTAL PROCEDURES

Cloning, Mutagenesis, and Stable Cell Lines

ZMYND8 (Uniprot: Q9ULU4, isoform 13) WT, or mutant was cloned into pDONR221, pDEST 5′ Triple-FLAG-pcDNA5-FRT-TO, or pDEST 5′ BirA*-FLAG-pcDNA5-FRT-TO vectors; the PHD/BRD/PWWP cassette was cloned into the pNIC-ZB vector (GenBank: GU452710). Proteins of interest were stably expressed in T-REx Flp-In HEK293 cells as described previously (Couzens et al., 2013; Supplemental Experimental Procedures).

Protein Expression and Purification

Recombinant proteins were expressed and purified by affinity and size exclusion chromatography as described previously (Savitsky et al., 2010).

Recombinant Histone Peptides

Recombinant histone peptides carrying specific acetylations were produced in vivo using the amber stop codon/suppressor tRNA technology (Neumann et al., 2009) with several modifications that increased overall yields from 10% to above 70% (Supplemental Experimental Procedures).

SPOT Peptide Assays

Human histone peptides were synthesized on cellulose arrays according to the SPOT synthesis described previously (Filippakopoulos et al., 2012).

BLI

Experiments were performed on an Octet RED384 system using commercial libraries (AltaBioSciences) or peptides synthesized as described previously (Rothbart et al., 2012b).

ITC

Experiments were carried out on an ITC200 titration microcalorimeter, and data analysis was performed using the MicroCal Origin software as described previously (Filippakopoulos et al., 2012); data are presented in the Supplemental Information.

Crystallization, Data Collection, and Structure Determination

Purified recombinant protein was crystallized at 4°C. Zinc single wavelength anomalous dispersion phasing (Zn-SAD) data were collected at Diamond Lightsource. Identification of three putative Zn sites in the SAD dataset allowed phasing, followed by manual model building and refinement as explained in the Supplemental Experimental Procedures.

Chromatin Fractionation Assay

Chromatin fractionation assays were performed with HEK293T cells transfected with ZMYND8 WT or mutant plasmids as described previously (Rothbart et al., 2012a).

Histone Immunoprecipitation

Cells were lysed, chromatin was digested, and co-immunoprecipitation was performed using antibodies described in the Supplemental Experimental Procedures.

FRAP

FRAP experiments were performed using a protocol modified from previous studies (Filippakopoulos et al., 2010) as described in the Supplemental Experimental Procedures.

EMSA

Oligonucleotide probes encoding AT-rich or GC-rich sequences were used together with recombinant ZMYND8 constructs (WT or mutant) to determine protein binding in electrophoresis experiments. For details, see the Supplemental Experimental Procedures.

Mass Spectrometry

Samples were analyzed by mass spectrometry in liquid chromatography-tandem MS experiments on a TripleTOF 5600 instrument. AP-MS as well as BioID-MS were performed as described previously (Lambert et al., 2014, 2015).

RNAi

Small RNAi was used to knock down ZMYND8 or HATs in HEK293 cells expressing inducible 3×FLAG-ZMYND8. For details, see the Supplemental Experimental Procedures.

ChIP and ChIP-Seq

Details regarding chromatin preparation, ChIP, and ChIP-PCR can be found in the Supplemental Experimental Procedures. Library construction and sequencing were performed at the Wellcome Trust Centre for Human Genetics using the PrepX Complete ILMN 32i DNA Library Kit (Illumina) and Illumina adapters. Purified DNA was analyzed by deep sequencing on the Illumina HiSeq 2500 platform.

ChIP-Seq Data Analysis

Reads were mapped to hg19 (NCBI build 37) using BWA-MEM, allowing up to two mismatches. Genome ChIP-seq profiles were generated using HOMER (Heinz et al., 2010), and enrichment profiles were visualized using ngs.plot (Shen et al., 2014) as described in the Supplemental Experimental Procedures.

Statistical Methods

For FRAP experiments, mean values and SEM were determined from 15 cells per condition tested over multiple experiments, and *P values* were calculated using two-tailed Student's t test or one-way ANOVA followed by Dunnett's multiple comparison test. ChIP-qPCR *P values* were determined using one-way ANOVA followed by Dunnett's multiple comparison test on biological replicates (n = 3). In AP-MS and BioID-MS experiments, data were analyzed with SAINTexpress to calculate the probability value of each potential protein-protein interaction from background contaminants using default parameters. Controls were compressed to six samples, and a false discovery rate (FDR) of 1% or lower was required for proteins to be classified as significant interaction partners of ZMYND8.

ACCESSION NUMBERS

The accession number for the model and structure factors of the ZMYND8 PHD/BRD/PWWP cassette reported in this paper is PDB: 4COS. The accession numbers for the mass spectrometry and high-throughput sequencing data reported in this paper are MassIVE: MSV000079336 and GEO: GSE81696, respectively.

SUPPLEMENTAL INFORMATION

Supplemental Information includes Supplemental Experimental Procedures, twelve figures, and nine tables and can be found with this article online at http://dx.doi.org/10.1016/j.celrep.2016.11.014.

AUTHOR CONTRIBUTIONS

P.S. prepared recombinant proteins, designed and cloned full-length ZMYND8 constructs and mutants, and performed crystallization trials. P.S. and S.P. designed, carried out, and analyzed biophysical experiments. T.K. performed crystallographic studies. T.F. carried out FRAP studies and immunoblotting. J.P.L established stable cell lines. J.P.L. and A.C.G. designed and performed proteomic experiments and analyzed data. E.S., B.S., and K.K. prepared recombinant peptides and performed chromatin fractionation assays. T.F., H.F., and C.G. designed and performed EMSA assays. C.Y.W. performed ChIP-seq assays. C.Y.W. and T.F. performed ChIP-qPCR assays. A.K., A.S., and P.F. analyzed ChIP-seq data. P.F. designed and supervised the study and prepared the manuscript. All authors read and commented on the manuscript.

ACKNOWLEDGMENTS

The authors are grateful for support received by the SGC, a registered charity (number 1097737) that receives funds from AbbVie, Bayer Pharma AG, Boehringer Ingelheim, Canada Foundation for Innovation, Eshelman Institute for Innovation, Genome Canada, Innovative Medicines Initiative (EU/EFPIA) (ULTRA-DD Grant 115766), Janssen, Merck & Co., Novartis Pharma AG, Ontario Ministry of Economic Development and Innovation, Pfizer, São Paulo Research Foundation-FAPESP, Takeda, and the Wellcome Trust (092809/Z/10/Z). P.F., S.P., and C.Y.W. are supported by a Wellcome Career Development Fellowship (095751/Z/11/Z). The work of P.F. is funded by Ludwig Cancer Research and the Medical Research Council (MR/N010051/1). T.F. is supported by an Uehara Memorial Foundation Fellowship (201330102). A.-C.G. is supported by the Government of Canada through Genome Canada and the Ontario Genomics Institute (OGI-088 and OGI-097) and the Canadian Institutes of Health Research (FDN 143301), and is the Canada Research Chair in Functional Proteomics and the Lea Reichmann Chair in Cancer Proteomics. J.P.L. was supported by a postdoctoral fellowship from CIHR and by a TD Bank Health Research Fellowship at the Lunenfeld-Tanenbaum Research Institute. B.D.S. is supported by a NIH grant (GM068088). E.S. was funded by a grant from the National Institute of General Medical Sciences Division of Training, Workforce Development, and Diversity under the Institutional Research and Academic Career Development Award (K12-GM000678). We thank Angie Green and the High-Throughput Genomics Group at the Wellcome Trust Centre for Human Genetics (funded by Wellcome Trust grant reference 090532/Z/09/Z) for the generation of sequencing data. We also thank Diamond Light Source for access to beamlines I03 and I04 (proposal number mx8421),which contributed to the results presented here. The mutation data used in this study are based on data generated by the TCGA Research Network (http://cancergenome.nih.gov/). We appreciate the ChIP-seq data of H3K14ac (GSM521881) and H3K27ac (GSM521887) from the NIH Roadmap Epigenomics Mapping Consortium (http://www.nihroadmap.nih.gov/epigenomics.org/).

Received: June 15, 2016
Revised: September 27, 2016
Accepted: October 31, 2016
Published: December 6, 2016

REFERENCES

Adhikary, S., Sanyal, S., Basu, M., Sengupta, I., Sen, S., Srivastava, D.K., Roy, S., and Das, C. (2016). Selective Recognition of H3.1K36 Dimethylation/H4K16 Acetylation Facilitates the Regulation of All-trans-retinoic Acid (ATRA)-responsive Genes by Putative Chromatin Reader ZMYND8. J. Biol. Chem. *291*, 2664–2681.

Choudhary, C., Kumar, C., Gnad, F., Nielsen, M.L., Rehman, M., Walther, T.C., Olsen, J.V., and Mann, M. (2009). Lysine acetylation targets protein complexes and co-regulates major cellular functions. Science *325*, 834–840.

Couzens, A.L., Knight, J.D., Kean, M.J., Teo, G., Weiss, A., Dunham, W.H., Lin, Z.Y., Bagshaw, R.D., Sicheri, F., Pawson, T., et al. (2013). Protein interaction network of the mammalian Hippo pathway reveals mechanisms of kinase-phosphatase interactions. Sci. Signal. *6*, rs15.

Eberl, H.C., Spruijt, C.G., Kelstrup, C.D., Vermeulen, M., and Mann, M. (2013). A map of general and specialized chromatin readers in mouse tissues generated by label-free interaction proteomics. Mol. Cell *49*, 368–378.

Feller, C., Forné, I., Imhof, A., and Becker, P.B. (2015). Global and specific responses of the histone acetylome to systematic perturbation. Mol. Cell *57*, 559–571.

Filippakopoulos, P., Qi, J., Picaud, S., Shen, Y., Smith, W.B., Fedorov, O., Morse, E.M., Keates, T., Hickman, T.T., Felletar, I., et al. (2010). Selective inhibition of BET bromodomains. Nature *468*, 1067–1073.

Filippakopoulos, P., Picaud, S., Mangos, M., Keates, T., Lambert, J.P., Barsyte-Lovejoy, D., Felletar, I., Volkmer, R., Müller, S., Pawson, T., et al. (2012). Histone recognition and large-scale structural analysis of the human bromodomain family. Cell *149*, 214–231.

Gao, J., Aksoy, B.A., Dogrusoz, U., Dresdner, G., Gross, B., Sumer, S.O., Sun, Y., Jacobsen, A., Sinha, R., Larsson, E., et al. (2013). Integrative analysis of complex cancer genomics and clinical profiles using the cBioPortal. Sci. Signal. *6*, pl1.

Garner, M.M., and Revzin, A. (1981). A gel electrophoresis method for quantifying the binding of proteins to specific DNA regions: application to components of the Escherichia coli lactose operon regulatory system. Nucleic Acids Res. *9*, 3047–3060.

Gong, F., Chiu, L.Y., Cox, B., Aymard, F., Clouaire, T., Leung, J.W., Cammarata, M., Perez, M., Agarwal, P., Brodbelt, J.S., et al. (2015). Screen identifies bromodomain protein ZMYND8 in chromatin recognition of transcription-associated DNA damage that promotes homologous recombination. Genes Dev. *29*, 197–211.

Havugimana, P.C., Hart, G.T., Nepusz, T., Yang, H., Turinsky, A.L., Li, Z., Wang, P.I., Boutz, D.R., Fong, V., Phanse, S., et al. (2012). A census of human soluble protein complexes. Cell *150*, 1068–1081.

Heinz, S., Benner, C., Spann, N., Bertolino, E., Lin, Y.C., Laslo, P., Cheng, J.X., Murre, C., Singh, H., and Glass, C.K. (2010). Simple combinations of lineage-determining transcription factors prime cis-regulatory elements required for macrophage and B cell identities. Mol. Cell *38*, 576–589.

Jacobson, R.H., Ladurner, A.G., King, D.S., and Tjian, R. (2000). Structure and function of a human TAFII250 double bromodomain module. Science *288*, 1422–1425.

Jin, Q., Yu, L.R., Wang, L., Zhang, Z., Kasper, L.H., Lee, J.E., Wang, C., Brindle, P.K., Dent, S.Y., and Ge, K. (2011). Distinct roles of GCN5/PCAF-mediated H3K9ac and CBP/p300-mediated H3K18/27ac in nuclear receptor transactivation. EMBO J. *30*, 249–262.

Kettenbach, A.N., Schweppe, D.K., Faherty, B.K., Pechenick, D., Pletnev, A.A., and Gerber, S.A. (2011). Quantitative phosphoproteomics identifies substrates and functional modules of Aurora and Polo-like kinase activities in mitotic cells. Sci. Signal. *4*, rs5.

Kloet, S.L., Baymaz, H.I., Makowski, M., Groenewold, V., Jansen, P.W., Berendsen, M., Niazi, H., Kops, G.J., and Vermeulen, M. (2015). Towards elucidating the stability, dynamics and architecture of the nucleosome remodeling and deacetylase complex by using quantitative interaction proteomics. FEBS J. *282*, 1774–1785.

Lambert, J.P., Tucholska, M., Pawson, T., and Gingras, A.C. (2014). Incorporating DNA shearing in standard affinity purification allows simultaneous identification of both soluble and chromatin-bound interaction partners. J. Proteomics *100*, 55–59.

Lambert, J.P., Tucholska, M., Go, C., Knight, J.D., and Gingras, A.C. (2015). Proximity biotinylation and affinity purification are complementary approaches for the interactome mapping of chromatin-associated protein complexes. J. Proteomics *118*, 81–94.

Li, H., Ilin, S., Wang, W., Duncan, E.M., Wysocka, J., Allis, C.D., and Patel, D.J. (2006). Molecular basis for site-specific read-out of histone H3K4me3 by the BPTF PHD finger of NURF. Nature *442*, 91–95.

Makde, R.D., England, J.R., Yennawar, H.P., and Tan, S. (2010). Structure of RCC1 chromatin factor bound to the nucleosome core particle. Nature *467*, 562–566.

Malovannaya, A., Lanz, R.B., Jung, S.Y., Bulynko, Y., Le, N.T., Chan, D.W., Ding, C., Shi, Y., Yucer, N., Krenciute, G., et al. (2011). Analysis of the human endogenous coregulator complexome. Cell *145*, 787–799.

McGinty, R.K., Henrici, R.C., and Tan, S. (2014). Crystal structure of the PRC1 ubiquitylation module bound to the nucleosome. Nature *514*, 591–596.

Morinière, J., Rousseaux, S., Steuerwald, U., Soler-López, M., Curtet, S., Vitte, A.L., Govin, J., Gaucher, J., Sadoul, K., Hart, D.J., et al. (2009). Cooperative binding of two acetylation marks on a histone tail by a single bromodomain. Nature *461*, 664–668.

Nagy, Z., Riss, A., Fujiyama, S., Krebs, A., Orpinell, M., Jansen, P., Cohen, A., Stunnenberg, H.G., Kato, S., and Tora, L. (2010). The metazoan ATAC and SAGA coactivator HAT complexes regulate different sets of inducible target genes. Cell. Mol. Life Sci. *67*, 611–628.

Neumann, H., Hancock, S.M., Buning, R., Routh, A., Chapman, L., Somers, J., Owen-Hughes, T., van Noort, J., Rhodes, D., and Chin, J.W. (2009). A method for genetically installing site-specific acetylation in recombinant histones defines the effects of H3 K56 acetylation. Mol. Cell *36*, 153–163.

Peña, P.V., Davrazou, F., Shi, X., Walter, K.L., Verkhusha, V.V., Gozani, O., Zhao, R., and Kutateladze, T.G. (2006). Molecular mechanism of histone H3K4me3 recognition by plant homeodomain of ING2. Nature *442*, 100–103.

Picaud, S., and Filippakopoulos, P. (2015). SPOTing Acetyl-Lysine Dependent Interactions. Microarrays (Basel) *4*, 370–388.

Poleshko, A., Einarson, M.B., Shalginskikh, N., Zhang, R., Adams, P.D., Skalka, A.M., and Katz, R.A. (2010). Identification of a functional network of human epigenetic silencing factors. J. Biol. Chem. *285*, 422–433.

Rothbart, S.B., Krajewski, K., Nady, N., Tempel, W., Xue, S., Badeaux, A.I., Barsyte-Lovejoy, D., Martinez, J.Y., Bedford, M.T., Fuchs, S.M., et al. (2012a). Association of UHRF1 with methylated H3K9 directs the maintenance of DNA methylation. Nat. Struct. Mol. Biol. *19*, 1155–1160.

Rothbart, S.B., Krajewski, K., Strahl, B.D., and Fuchs, S.M. (2012b). Peptide microarrays to interrogate the "histone code". Methods Enzymol. *512*, 107–135.

Ruthenburg, A.J., Li, H., Patel, D.J., and Allis, C.D. (2007). Multivalent engagement of chromatin modifications by linked binding modules. Nat. Rev. Mol. Cell Biol. *8*, 983–994.

Ruthenburg, A.J., Li, H., Milne, T.A., Dewell, S., McGinty, R.K., Yuen, M., Ueberheide, B., Dou, Y., Muir, T.W., Patel, D.J., and Allis, C.D. (2011). Recognition of a mononucleosomal histone modification pattern by BPTF via multivalent interactions. Cell *145*, 692–706.

Savitsky, P., Bray, J., Cooper, C.D., Marsden, B.D., Mahajan, P., Burgess-Brown, N.A., and Gileadi, O. (2010). High-throughput production of human proteins for crystallization: the SGC experience. J. Struct. Biol. *172*, 3–13.

Schübeler, D. (2015). Function and information content of DNA methylation. Nature *517*, 321–326.

Shen, L., Shao, N., Liu, X., and Nestler, E. (2014). ngs.plot: Quick mining and visualization of next-generation sequencing data by integrating genomic databases. BMC Genomics *15*, 284.

Shen, H., Xu, W., Guo, R., Rong, B., Gu, L., Wang, Z., He, C., Zheng, L., Hu, X., Hu, Z., et al. (2016). Suppression of Enhancer Overactivation by a RACK7-Histone Demethylase Complex. Cell *165*, 331–342.

Strahl, B.D., and Allis, C.D. (2000). The language of covalent histone modifications. Nature *403*, 41–45.

Tan, M., Luo, H., Lee, S., Jin, F., Yang, J.S., Montellier, E., Buchou, T., Cheng, Z., Rousseaux, S., Rajagopal, N., et al. (2011). Identification of 67 histone marks and histone lysine crotonylation as a new type of histone modification. Cell *146*, 1016–1028.

Tessarz, P., and Kouzarides, T. (2014). Histone core modifications regulating nucleosome structure and dynamics. Nat. Rev. Mol. Cell Biol. *15*, 703–708.

Tsai, W.W., Wang, Z., Yiu, T.T., Akdemir, K.C., Xia, W., Winter, S., Tsai, C.Y., Shi, X., Schwarzer, D., Plunkett, W., et al. (2010). TRIM24 links a non-canonical histone signature to breast cancer. Nature *468*, 927–932.

Wang, J., Qin, S., Li, F., Li, S., Zhang, W., Peng, J., Zhang, Z., Gong, Q., Wu, J., and Shi, Y. (2014). Crystal structure of human BS69 Bromo-ZnF-PWWP reveals its role in H3K36me3 nucleosome binding. Cell Res. *24*, 890–893.

Wen, H., Li, Y., Xi, Y., Jiang, S., Stratton, S., Peng, D., Tanaka, K., Ren, Y., Xia, Z., Wu, J., et al. (2014). ZMYND11 links histone H3.3K36me3 to transcription elongation and tumour suppression. Nature *508*, 263–268.

Xi, Q., Wang, Z., Zaromytidou, A.I., Zhang, X.H., Chow-Tsang, L.F., Liu, J.X., Kim, H., Barlas, A., Manova-Todorova, K., Kaartinen, V., et al. (2011). A poised chromatin platform for TGF-β access to master regulators. Cell *147*, 1511–1524.

Yang, M., Gocke, C.B., Luo, X., Borek, D., Tomchick, D.R., Machius, M., Otwinowski, Z., and Yu, H. (2006). Structural basis for CoREST-dependent demethylation of nucleosomes by the human LSD1 histone demethylase. Mol. Cell *23*, 377–387.

Cell Reports, Volume *17*

Supplemental Information

Multivalent Histone and DNA Engagement by a PHD/BRD/PWWP Triple Reader Cassette Recruits ZMYND8 to K14ac-Rich Chromatin

Pavel Savitsky, Tobias Krojer, Takao Fujisawa, Jean-Philippe Lambert, Sarah Picaud, Chen-Yi Wang, Erin K. Shanle, Krzysztof Krajewski, Hans Friedrichsen, Alexander Kanapin, Colin Goding, Matthieu Schapira, Anastasia Samsonova, Brian D. Strahl, Anne-Claude Gingras, and Panagis Filippakopoulos

Supplemental Figure S1

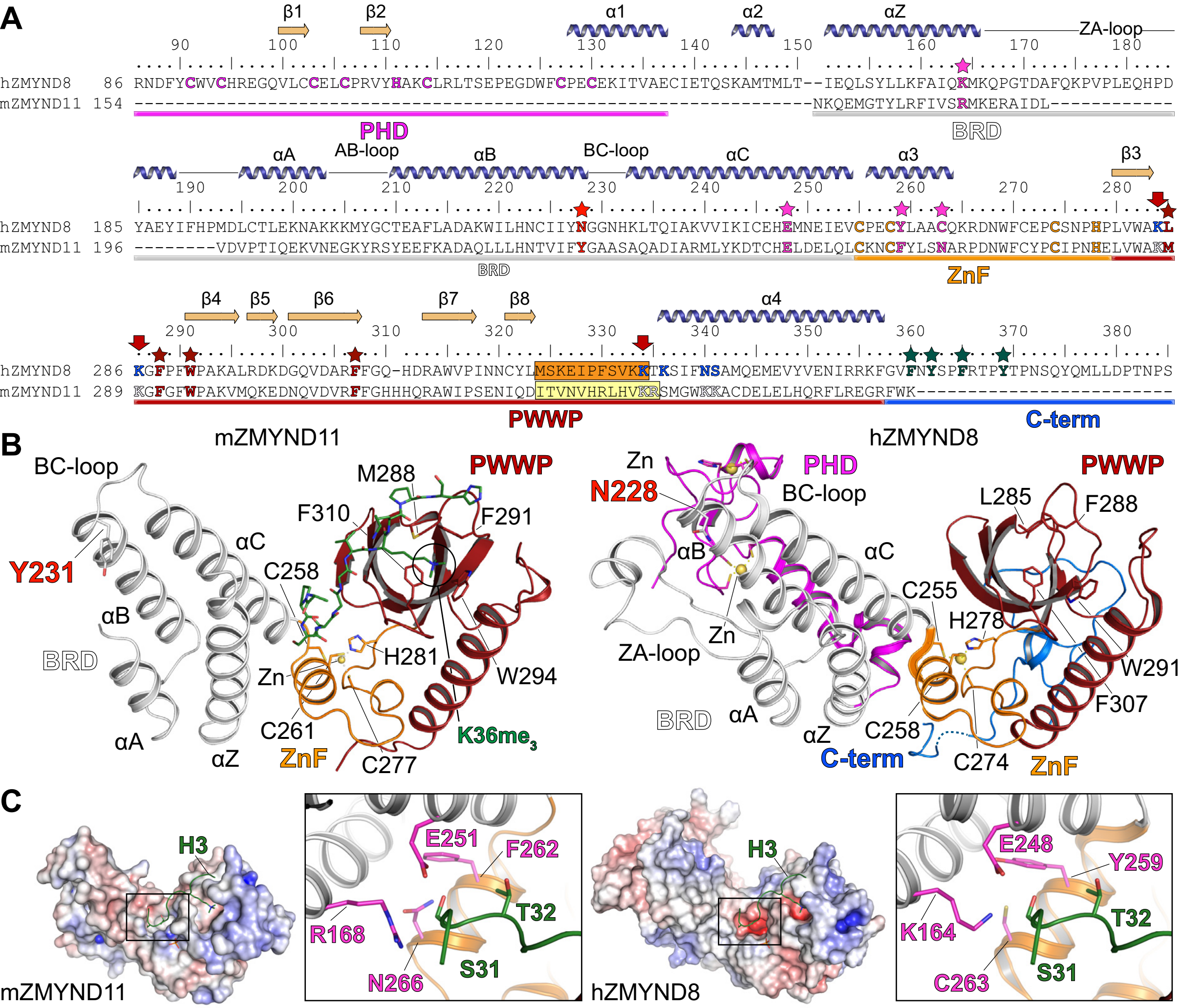

Supplemental Figure S1 – *Comparison of ZMYND8 and ZMYND11, related to Figure 1*. (**A**) Sequence alignment of human ZMYND8 and mouse ZMYND11. Both proteins share a common domain architecture, including three reader modules on their N-terminus, however ZMYND11 lacks the family-conserved asparagine (Y231 in mZMYND11, N228 in ZMYND8) which is responsible for binding to acetylated peptides (highlighted with a red * in the alignment). Secondary structural elements are highlighted and key residues are coloured in red or blue and highlighted with an arrow. The loop between the last β-sheet (β8) of the PWWP domain and the long helix (α4) before the C-terminal portion of the crystallized construct is highlighted (orange box for ZMYND11 and pale yellow for ZMYND8). C-terminal aromatic residues are highlighted in green and are annotated with a star. The PWWP aromatic/hydrophobic cage residues are also highlighted in dark red with a star. (**B**) Crystal structures of the BRD/PWWP domains of ZMYND11 in complex with an H3K36me$_3$ peptide (PDB: 4N4I) and the PHD/BRD/PWWP domains of ZMYND8 with structural elements annotated. Both proteins contain a zinc-finger insertion between the BRD and PWWP domains (shown in orange) which coordinates a zinc ion. The PHD domain of ZMYND8 packs behind the BRD module while the BRD/ZnF/PWWP arrangement is identical to that seen in ZMYND11. The C-terminal portion of the ZMYND8 crystallized construct is highlighted in blue and extends under the PWWP module towards the front of the protein, pointing towards the ZnF. Residues that form up the aromatic cage of the PWWP domains are highlighted (F291, W294, F310, capped by M288 in mZMYND11 and F288, W291, F307 capped by L285 in ZMYND8). (**C**) Residues responsible for S31 binding in mouse ZMYND11 (left) are mostly conserved in ZMYND8 with the exception of N266 which is replaced by C263. The H3.3 peptide found in ZMYND11 is overlaid on the ZMYND8 structure to highlight the position of S31.

Supplemental Figure S2

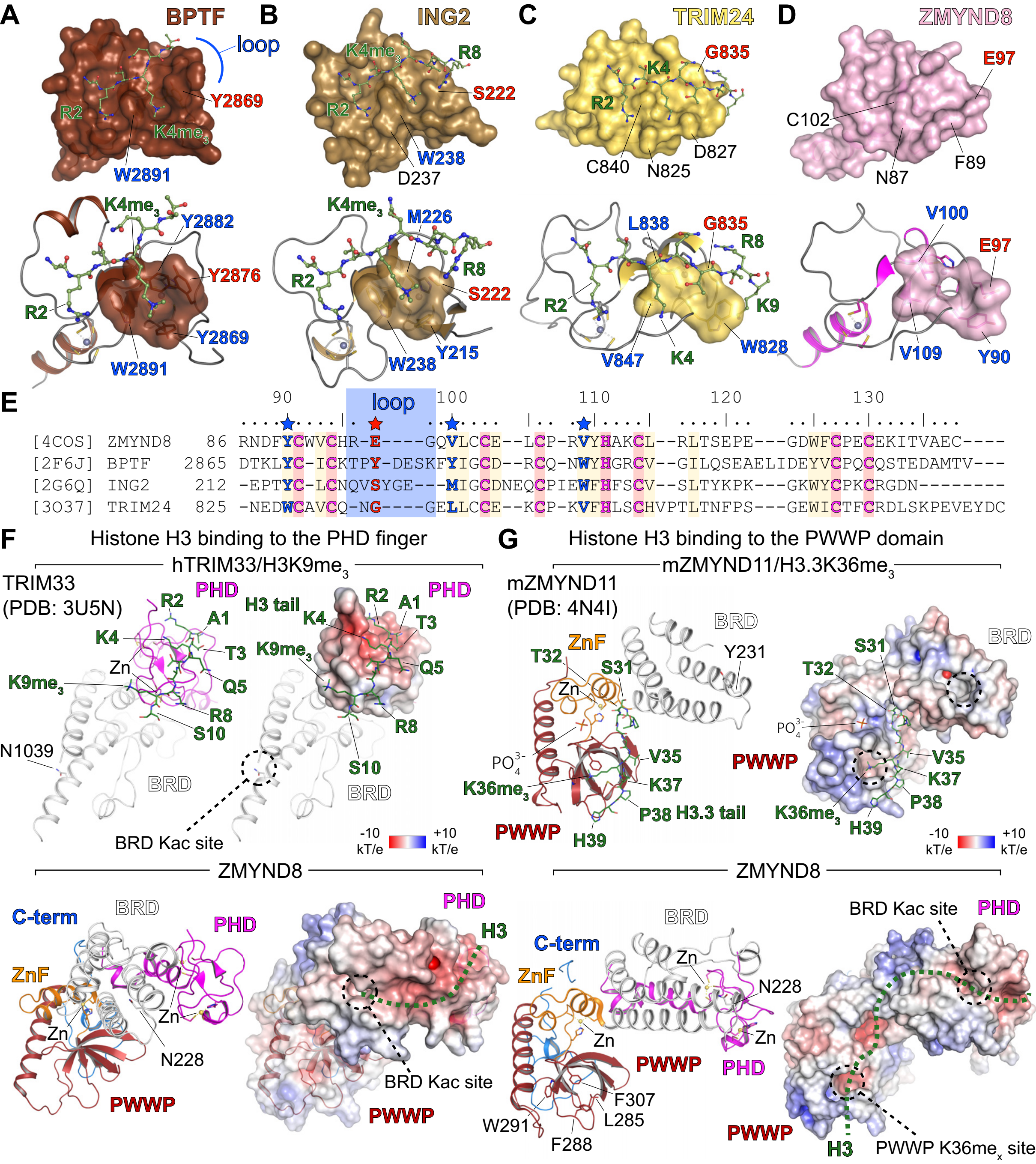

Supplemental Figure S2 – *Structural rational for ZMYND8 histone tail binding, related to Figures 1 & 2*. (**A**) K4me$_3$ binding to the PHD finger of BPTF (PDB: 2F6J). Two grooves formed around W2891 separate the histone R2 and the modified K4me$_3$ which are accommodated on the protein surface (top panel). A typical full aromatic cage, formed by Y2882, Y2876, Y2869 and W2891 surrounds the K4me$_3$ (bottom panel). (**B**) K4me$_3$ binding to the PHD finger of ING2 (PDB: 2G6Q). R2 and K4me$_3$ are separated by the bulky W238 and are accommodated in two grooves on the PHD surface. A partial hydrophobic groove formed by M296, Y215 and W238 accommodates K4me$_3$ which packs next to S222. (**C**) Binding of K4 to the PHD finger of TRIM24 (PDB: 3O37). K4 inserts in the groove between C840, N825 and D827 (top panel). In this case the aromatic cage is replaced by L838, W828 and V847 which present an open surface to the unmodified lysine, capped by G835 on the flexible loop to the right of the pocket. (**D**) The PHD finger of ZMYND8 resembles that of TRIM24, lacking an aromatic cage. A shallow groove formed by C102, N87 and F89 is present, with hydrophobic residues completing the surface (including V100 and V109) which is capped by E97 on the loop side. (**E**) Structural alignment of the four PHD finger shown in (a-d) highlighting residues forming the cage (shown in blue and annotated with a '*' as well as those found on a variable length loop region and cap the right side of the cage (shown in red and highlighted by a '*'). Conserved residues are also highlighted and annotated. (**F**) Comparison of the PHD finger (in magenta) from the PHD/BRD readers of human TRIM33 (top) and the PHD finger (in magenta) from the PHD/BRD/PWWP triple readers of ZMYND8 (bottom). Both structures have been superimposed based on the coordinates of the PHD domain. The TRIM33 structure (PDB: 3U5N) in complex with a modified histone H3 peptide (H3K9me$_3$, shown in green) demonstrates how the N-terminal portion of the peptide engages the entire PHD domain, eventually positioning the rest of the tail towards the BRD domain (the conserved asparagine, N1039 is highlighted on the structure). In the case of the ZMYND8 structure, the topology of the PHD and BRD modules is different, with the BRD cavity now much closer to the end of the PHD finger, suggesting that a histone H3 peptide can follow a similar path, positioning a histone tail towards the conserved asparagine of the BRD module (N228 in ZMYND8) which can accept an acetylated lysine (Kac site), as shown on the electrostatic surface of the protein (histone peptide path shown as a dotted thick green line). (**G**) Comparison of the PWWP domains (shown in red) of the mouse ZMYND11 (PDB: 4N4I shown on top) and human ZMYND8 (bottom panel). The mZMYND11 structure carries a histone H3.3 peptide modified at lysine K36 (H3.3K36me$_3$) which binds into the aromatic cage of the PWWP domain. In this particular structure S31 and T32 pack in a groove formed between the BRD and PWWP modules with the rest of the peptide extending on the PWWP surface. The BRD and PWWP cavities are highlighted with a dotted circle. Using a similar path, an H3 peptide that binds to the PHD

finger of ZMYND8 can extend to span the BRD cavity then the groove between the BRD and PWWP domains, potentially inserting K36 within the PWWP K36me$_x$ site formed by F288, W291 and F307.

Supplemental Figure S3

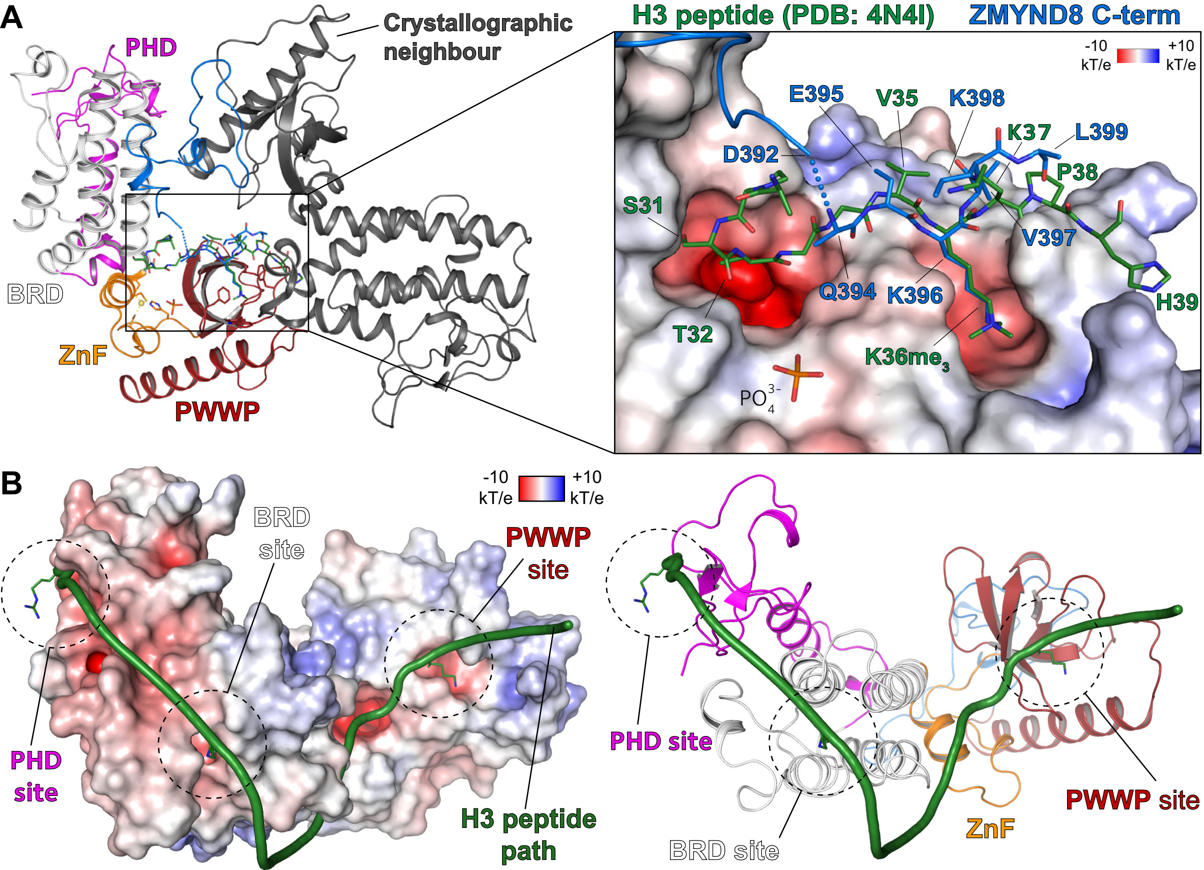

Supplemental Figure S3 – *Structural rational for ZMYND8 histone tail binding, related to Figures 1 & 2*. (**A**) Detail of the ZMYND8 structure highlighting packing of a symmetry equivalent (shown in gray) of the triple reader modules, inserting its C-terminal tail within the PWWP aromatic cage. Superimposition with the mouse ZMYND11 structure in complex with H3.3K36me$_3$ (PDB: 4N4I) shows good overlay of the ZMYND8 C-terminal K396 with the histone peptide's K36me$_3$ suggesting that the protein can bind to unmodified lysines. Key residues are highlighted coloured in green (histone H3.3 peptide) or blue (ZMYND8 crystallographic neighbour's C-terminal tail) on the electrostatic surface of the protein. A phosphate ion found on the mZMYND11 structure is positioned in a slightly positively charged pocket of the ZnF and PWWP domain surface, suggesting that small changes in peptide binding topology can be accommodated if T32 is phosphorylated. (**B**) Cartoon of hypothetical histone H3 peptide engagement by the triple reader modules of ZMYND8. The triple reader topology presents a charged surface that could potentially interact with a large portion of the histone N-terminal tail. Interfaces are presented in the PHD, BRD and PWWP sites, driving binding from all three reader domain sites.

Supplemental Figure S4

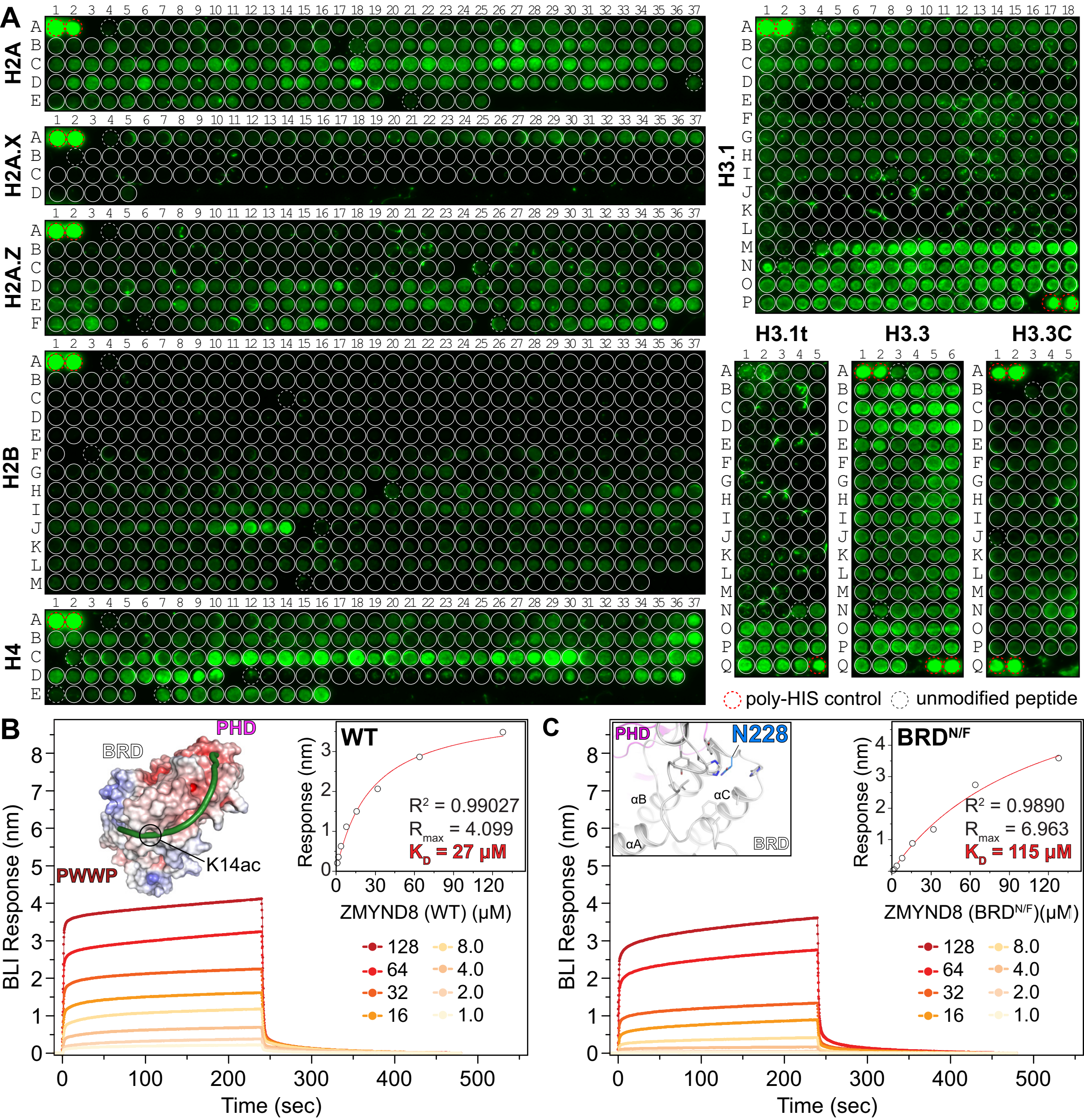

Supplemental Figure S4 – *ZMYND8 histone binding* in vitro*, related to Figure 2.* (**A**) Peptide SPOT arrays of human histones (H2, H3 and H4) carrying 20-aa long peptides with combinations of PTMs (including Kac, Kme_x (x =1,2,3), pS and pT). Histone isoforms (H2A, H2A.X, H2A.Z, H2B, H3.1t, H3.3 and H3.3C) were also tested to account for differences in sequence. Hexa-his-tagged recombinant ZMYND8 triple reader modules (PHD/PRD/PWWP) were incubated with the membranes and after washing were visualized with an anti-his antibody. The presence of multiple acetyl marks dominated the resulting binding that was observed. Peptide sequences are given in **Supplemental Table S2**. (**B**) Bio-layer interferometry (BLI) profiling of recombinant ZMYND8 (PHD/BRD/PWWP) binding to a synthetic H3K14ac biotinylated peptide (20-mer) immobilized on streptavidin sensors. The sensograms demonstrate binding and dissociation of the protein to the immobilized peptide at different concentrations. The inset shows the steady state response as a function of protein concentration calculated from the association and dissociation curves and fitted with a non-linear regression model. Fit parameters are given in the inset. A structural model highlighting the potential binding path of the H3 peptide crossing the BRD cavity where K14ac is predicted to bind is shown in the inset. Experiments were performed in 20 mM HEPES pH7.5, 200 mM NaCl, 20 mM TCEP. (**C**) Bio-layer inteferometry (BLI) evaluation of BRD mutant ($BRD^{N/F}$) ZMYND8 to an H3K14ac 40-mer peptide. Data and fitting is represented as in (B). The mutant protein exhibits weaker binding with slower and incomplete dissociation from the bound peptide suggesting non-specific binding.

Supplemental Figure S5

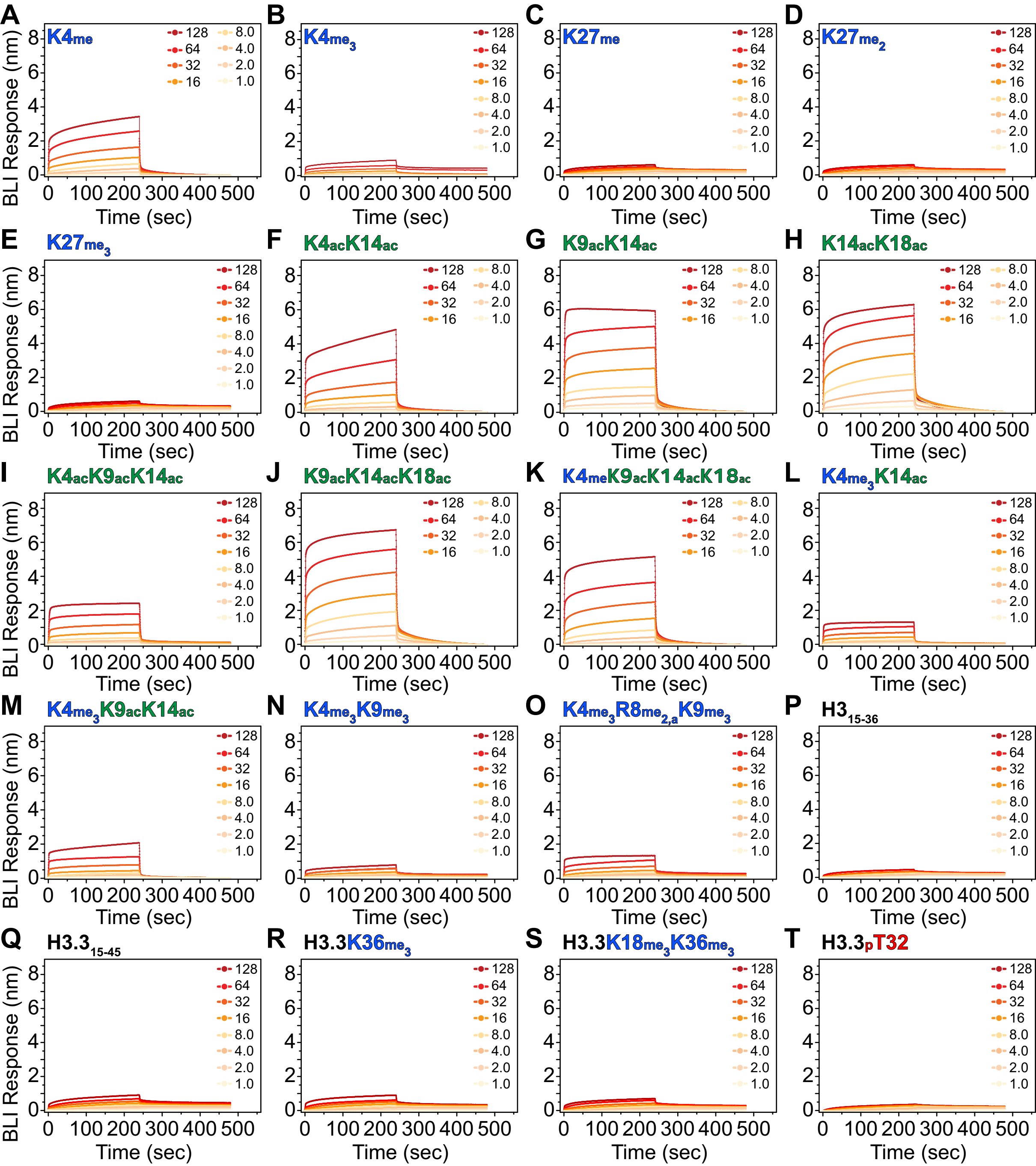

Supplemental Figure S5 – *In-solution validation of ZMYND8/histone H3 interactions employing biolayer interferometry (BLI), related to Figure 2*. Biotinylated histone H3 21-amino acid long peptides carrying different modification were immobilized on Super Streptavidin Biosensors and were profiled against a range of recombinant triple readers of ZMYND8. Association and dissociation measurements were performed in 20 mM HEPES pH7.5, 200 mM NaCl, 20 mM TCEP. Experiments were carried out at 25 °C with association and dissociation times of 240 sec using protein concentrations as indicated in the insets. Peptides tested (residues 1-21 unless otherwise stated) included: (**A**) K4me (**B**) K4me$_3$ (**C**) K27me (**D**) K27me$_2$ (**E**) K27me$_3$ (**F**) K4ac/K14ac (**G**) K9ac/K14ac (**H**) K14ac/K18ac (**I**) K4ac/K9ac/K14ac (**J**) K9ac/K14ac/K18ac (**K**) K4me/K9ac/K14ac/K18ac (**L**) K4me$_3$/K14ac (**M**) K4me$_3$/K9ac/K14ac (**N**) K4me$_3$/K9me$_3$ (**O**) K4me$_3$/R8me$_{2,a}$/K9me$_3$ (**P**) WT H3 (residues 15-36) (**Q**) WT H3.3 (residues 15-45) (**R**) H3.3 (residues 15-45) K36e$_3$ (**S**) H3.3 (residues 15-45) K18me$_3$/K36me$_3$ (**T**) H3.3 (residues 15-45) pT32.

Supplemental Figure S6

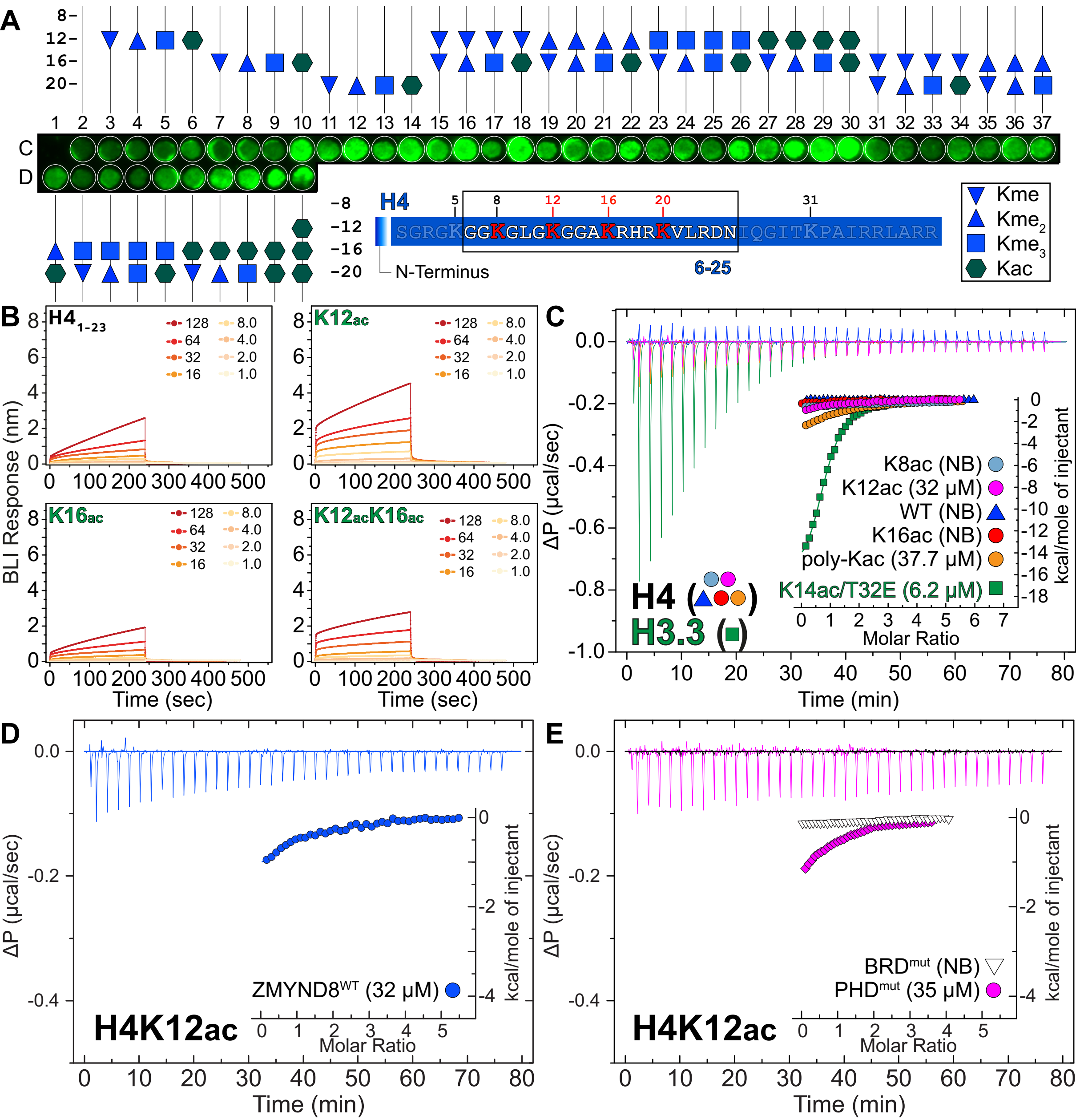

Supplemental Figure S6 – *ZMYND8 binding to histone H4, related to Figure 2.* (**A**) SPOT peptide array detail exploring binding of modified histone H4 peptides to the recombinant triple reader module of ZMYND8. Peptides span residues 6-24 of the human H4 tail and modified as indicated in the inset. (**B**) In-solution biolayer interferometry (BLI) validation of ZMYND8/histone H4 interactions. Biotinylated histone H4 21-amino acid long peptides carrying different modifications were immobilized on Super Streptavidin Biosensors and were profiled against a range of recombinant triple readers of ZMYND8. Association and dissociation measurements were performed in 20 mM HEPES pH7.5, 200 mM NaCl, 20 mM TCEP. Experiments were carried out at 25 °C with association and dissociation times of 240 sec using protein concentrations as indicated in the insets. Peptides tested (residues 1-21 unless otherwise stated) included: WT H4 N-terminal tail (1-23); K12ac; K16ac; K12ac/K16ac. (**C**) In solution evaluation of histone H4 binding by isothermal titration calorimetry (ITC). Raw injection heats for titrations of modified peptides (recombinantly produced 40-mers carrying specific modifications as indicated in the inset, deposited by an Amber codon system, or *in vitro* poly-acetylated using acetic anhydride) into a solution of ZMYND8 are shown in the main panel. The inset shows the normalized binding enthalpies corrected for the heat of peptide dilution as a function of binding site saturation (symbols as indicated in the figure). Solid lines represent a nonlinear least squares fit using a single-site binding model. Histone H4 peptide binding is driven by K12ac (K_D = 32 µM), an interaction which appears to be much weaker than the H3K14ac/T32E (K_D = 6 µM). (**D/E**) ITC validation of histone H4K12ac binding to ZMYND8 WT (D) or mutant (PHD or BRD, (E)) in solution. Data is presented as in (D). All ITC titrations were carried out in 20 mM HEPES pH 7.5 (at 25 °C), 200 mM NaCl and 15 °C while stirring at 1000 rpm.

Supplemental Figure S7

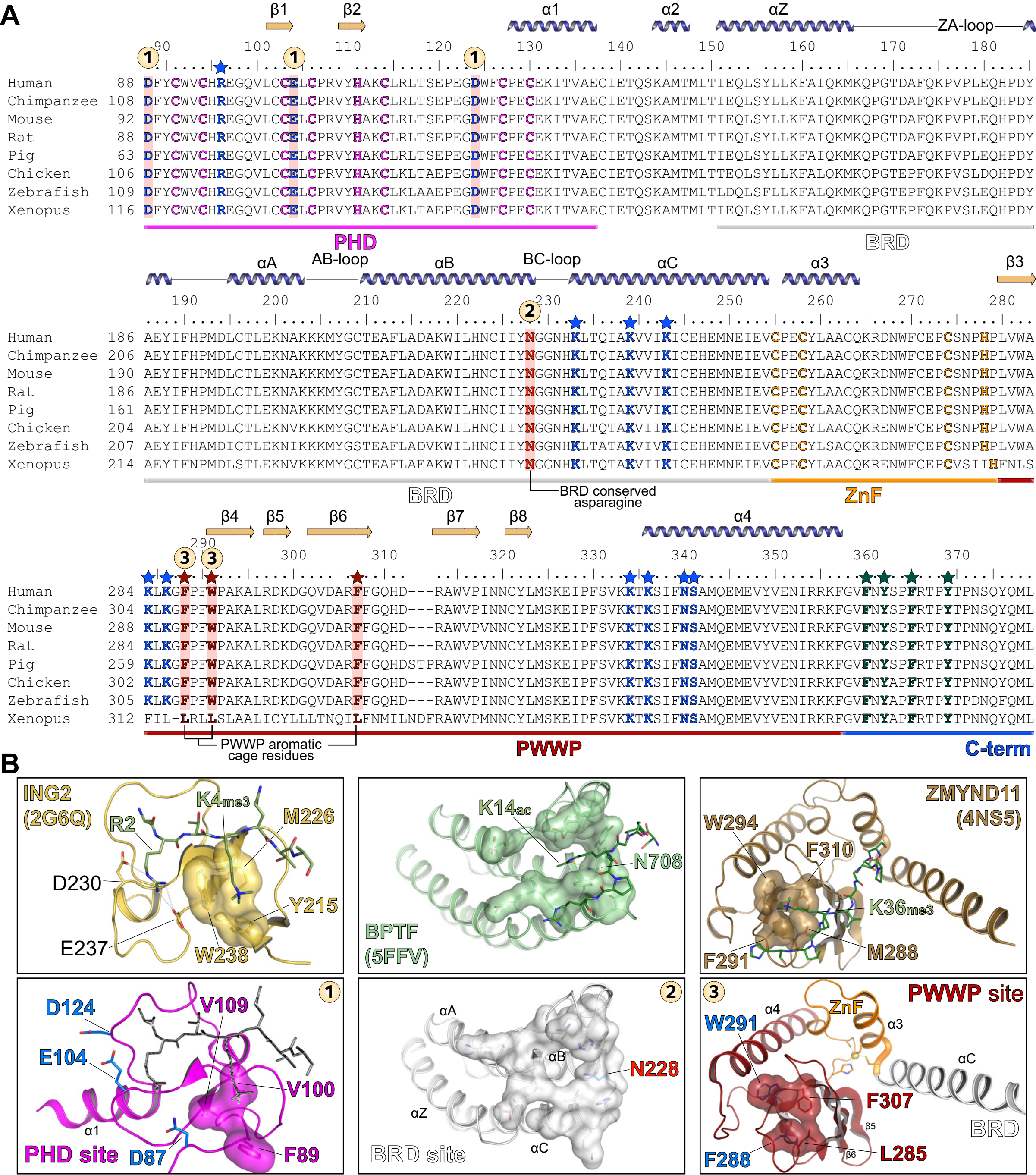

Supplemental Figure S7 – *Evolutionary conserved ZMYND8 residues and histone binding interfaces, related to Figure 2.* (**A**) Sequence alignment of human (ENSP00000418210), chimpanzee (ENSPTRP00000023370), mouse (ENSMUSP00000104892), rat (ENSRNOP00000025932), pig (ENSSSCP00000007942), chicken (ENSGALP00000007220), zebrafish (ENSDARP00000040234) and xenopus (ENSXETP00000063260) ZMYND8 triple reader modules, demonstrating high conservation of interaction interfaces. The secondary structure from the human ZMYND8 structure is displayed on top of the alignment and residues important for histone peptide binding are annotated. The three main sites (1: PHD, 2: BRD, 3: PWWP) are also highlighted in the inset. Residues implicated in DNA binding are highlighted in blue and annotated with a blue *. Residues implicated in C-terminal hydrophobic interactions are highlighted in green and annotated with a green *. The aromatic PWWP-cage residues are highlighted in dark red and indicated with a dark red *. (**B**) Structural overlays of histone peptide complexes with individual domains. Structural overlay of the ING2 PHD finger (top box) with the PHD domain of ZMYND8 (site '1'), highlighting residues important for K4me3 binding. Arg2 initiates interactions with the PHD loop region. The equivalent residues in ZMYND8 are annotated in blue (D87, E104 and D124), following the conservation highlighted in (a). A structural overlay of the BPTF bromodomain in complex with a K14ac peptide (top middle box) is shown compared to the ZMYND8 BRD module (middle lower box). The conserved asparagine (N228) is highlighted in blue. The PWWP domain of ZMYND11 (right top box) in complex with a K36me$_3$ peptide is shown in comparison to the ZMYND8 PWWP domain (right bottom box), highlighting the aromatic cage responsible for Kme$_x$ binding. Conserved residues are highlighted in blue (F288 and W291).

Supplemental Figure S8

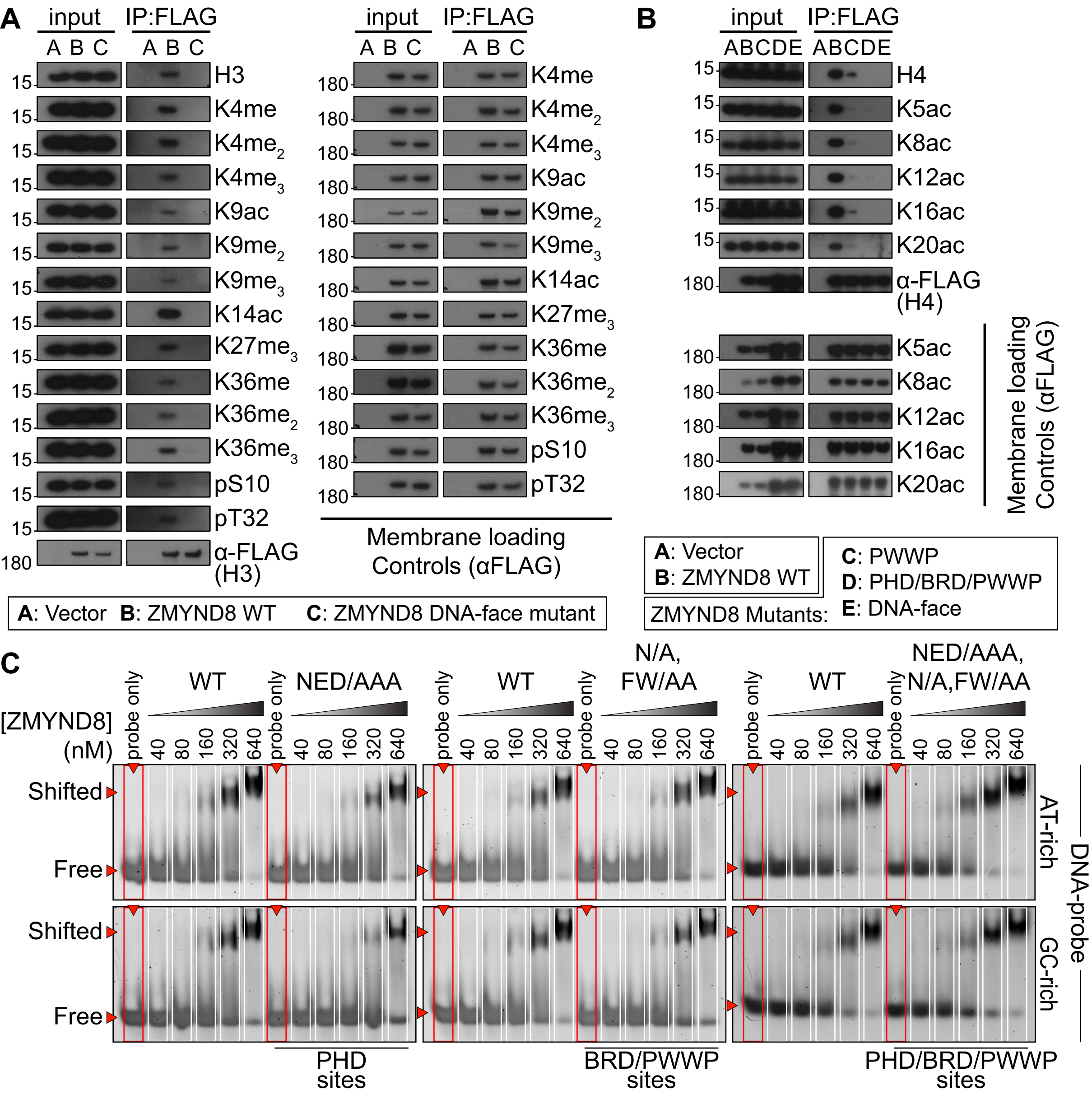

Supplemental Figure S8 – *ZMYND8 DNA binding is necessary for recruitment to DNA damaged sites and mutations of the triple reader ensemble do not affect recognition of DNA* in vitro*, related to Figures 3 & 4*. (**A**) Full-length ZMYND8 is unable to bind to histone H3 modifications in HEK293 cells when the DNA-binding sites identified in the structure are mutated to alanine (DNA-face mutant: R96A, K233A, K239A, K243A, K284A, K286A, K334A). HEK293 cells transiently transfected with full-length 3xFLAG ZMYND8 WT or DNA-face mutant and stained for different H3 modifications. Note that the DNA-face mutant of ZMYND8 shows much lower expression than the WT protein. (**B**) Effect of ZMYND8 mutations on histone H4 binding. HEK293 cells transiently transfected with full-length 3xFLAG ZMYND8 WT or PWWP (F288A/W291A), PHD/BRD/PWWP (N87A/E104A/D124A + N228F + F288A/W291A) or DNA-face (R96A, K233A, K239A, K243A, K284A, K286A, K334A) mutants, were stained for different H4 modifications. While mutations on the PWWP domain retained residual binding of all marks tested, mutation on all three domains completely abolished binding, as did mutations on the DNA-face of the protein. (**C**) Systematic mutation of domains within ZMYND8 did not have an effect in shifting a AT-rich (top) or GC-rich (bottom) DNA probe compared to the WT protein in non-radioactive EMSA assays. Mutations where introduced in the PHD domain (N87A/E104A/D124A), the BRD/PWWP domains (N228F; F288A/W291A), or all three reader domains (N87A/E104A/D124A/N228F/ F288A/W291A).

Supplemental Figure S9

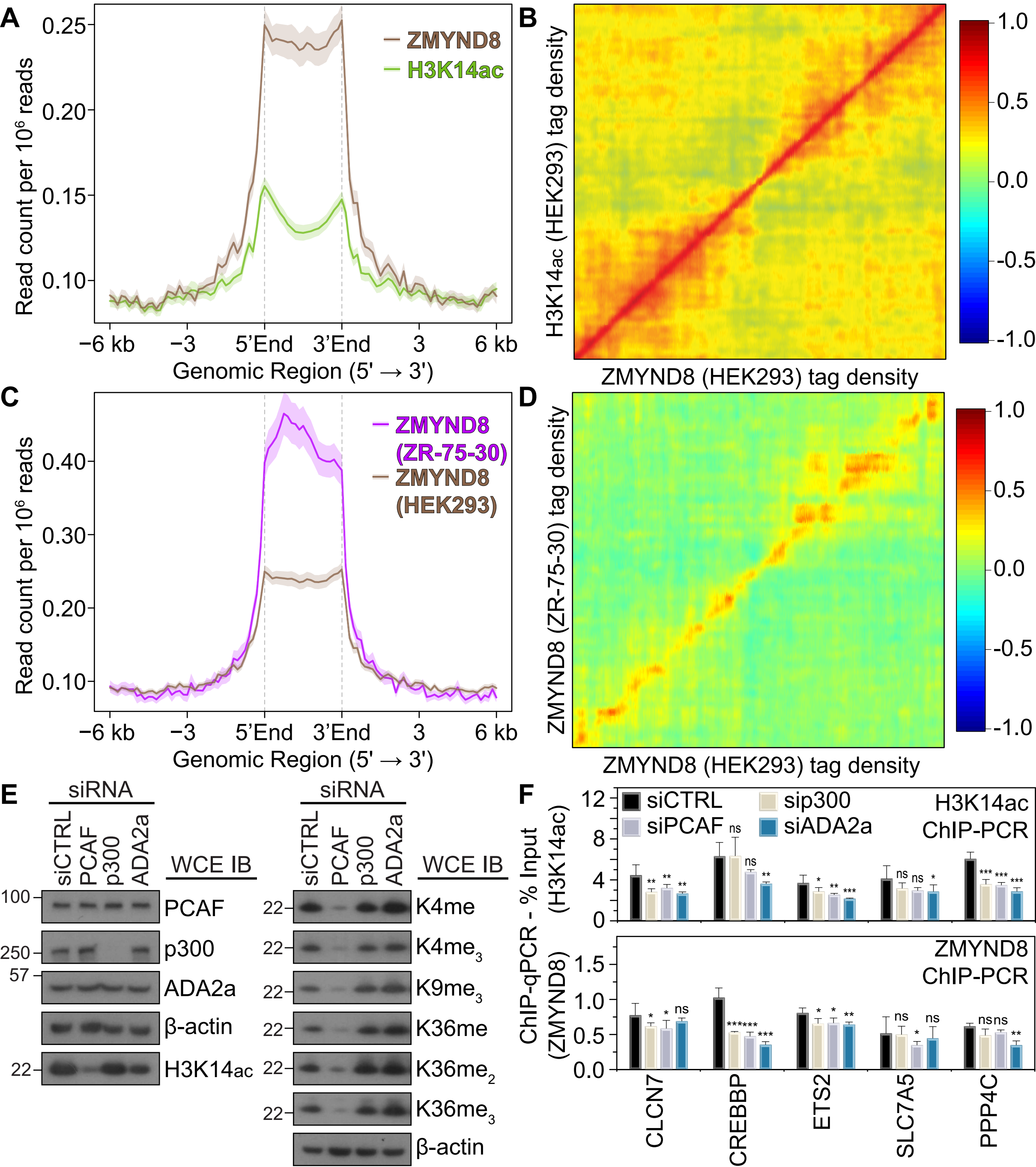

Supplemental Figure S9 – *ZMYND8 co-localizes with H3K14ac at Enhancers, related to Figure 5.* (**A**) Average profile of ZMYND8 and H3K14ac ChIP-seq signals on ±6 kb around enhancers (as defined by the FANTOM5 project) in HEK293 cells. (**B**) Canonical correlation matrix for ChIP-seq tag enrichment profiles observed in ±6 kb regions in ZMYND8-bound enhancers (defined by FANTOM5 annotations). High correlation (highlighted in red as indicated in the inset) was found between ZMYND8 and H3K14ac ChIP-seq signals in HEK293 cells. (**C**) Average profile of ZMYND8 ChIP-seq signals on ±6 kb around enhancers (as defined by the FANTOM5 project) in ZR-75-30 (data from GSE71323) and HEK293 cells. (**D**) Canonical correlation matrix for ZMYND8 ChIP-seq tag enrichment profiles observed in ± 6 kb regions in ZMYND8-bound enhancers (defined by FANTOM5 annotations), between HEK293 and ZR-75-30 (GSE71323) cells. Correlation is annotated as in (B). (**E**) siRNA depletion of HATs (PCAF, p300 and GCN5 via siRNA depletion of the ADA2a portion of the SAGA complex) resulted after 72h in reduction of H3K14ac (left panel). siHAT also (indirectly) resulted in variable attenuation of methylation marks found on histone H3 (right panel). (**F**) ChIP-qPCR enrichment of H3K14ac (top) and FLAG-ZMYND8 (bottom) signals following 72h siRNA depletion of HATs (siCTRL vs siPCAF, sip300, siADA2a). Signals are measured relative to IgG signal and presented as percentage of loaded input DNA. Data represent mean ± SEM from biological replicates (n=3). P values were calculated using Student’s t-test and are represented so that $P < 0.005$, “***”; $P < 0.01$, “**”; $P < 0.05$, “*”; Not-significant, “ns”.

Supplemental Figure S10

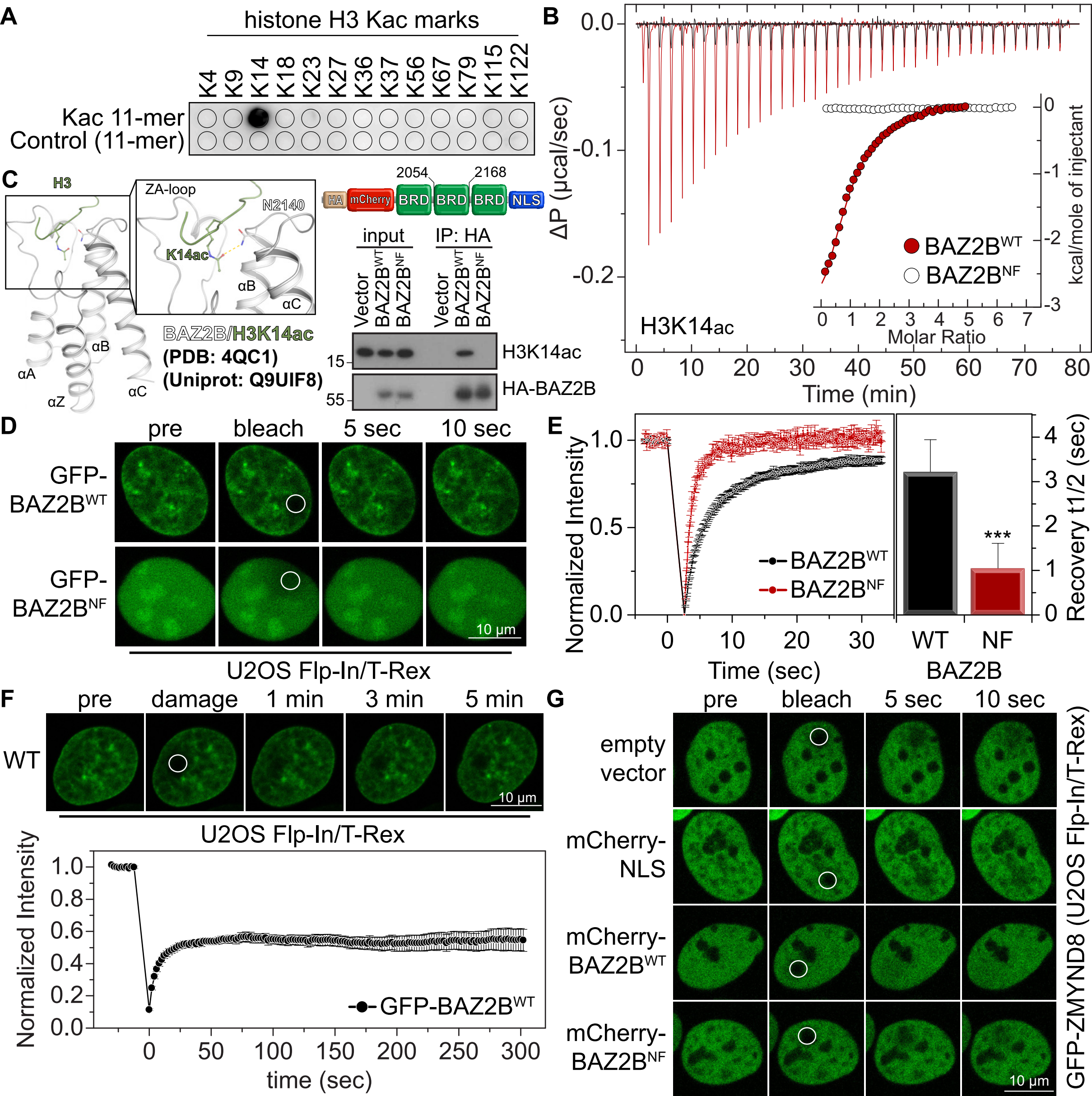

Supplemental Figure S10 – *Masking of H3K14ac using the bromodomain of BAZ2B prevents ZMYND8 binding, related to Figure 5.* (**A**) Cellulose-based SPOT validation of histone H3-peptide binding to recombinant BAZ2B bromodomain, showing high specificity for K14ac. (**B**) In solution evaluation of H3K14ac binding by ITC to recombinant BAZ2B WT or N2140F mutant. Raw injection heats for titrations of a 15-mer H3K14ac peptide into a solution of BAZ2B (WT or N2140F mutant) are shown in the main panel. The inset shows the normalized binding enthalpies corrected for the heat of peptide dilution as a function of binding site saturation (symbols as indicated in the figure). Solid lines represent a nonlinear least squares fit using a single-site binding model. While the WT proteins engages K14ac with low µM affinity the mutant shows no interaction at all. (**C**) Structural model of BAZ2B in complex with an H3K14ac peptide (PDB: 4QC1) showing the interaction between the conserved asparagine (N2140) and the acetylated lysine of histone H3. Mammalian constructs carrying 3x BRD modules of BAZ2B with N-terminal tags (HA/mCherry) and a C-terminal nuclear localization signal, bind by western blot to H3K14ac in HEK293 cells transiently transfected. The N2140F mutation abolishes the recognition of K14ac in cells. (**D**) Fluorescence recovery after photo-bleaching (FRAP) evaluation of GFP-3xBAZ2B (WT, top panels or N2140F mutant, bottom panels) dissociation from chromatin in U2OS cells transiently transfected with GFP-3xBAZ2B constructs. Target regions of photo-bleaching are indicated with a white circle. The scale bars correspond to a 10 µm region. (**E**) Quantitative comparison of time to half-maximal fluorescence recovery for GFP-3xBAZ2B. While the WT protein remains attached to chromatin, the mutant exhibits significant mobility as indicated by the faster recovery after bleaching. Data represent the mean ± SEM from multiple experiments in several cells (n = 15). (**F**) Data acquisition for longer times (5 min) did not show any accumulation of the GFP-BAZ2B protein onto the bleached site suggesting that there is no active recruitment to the damaged site. Data represent the mean ± SEM from multiple experiments in several cells (n = 15). (**G**) FRAP evaluation of full length GFP-tagged ZMYND8 dissociation from chromatin in U2OS Flp-In/TRex cells stably expressing GFP-ZMYND8. Nuclei of cells treated with vector, mCherry-NSL, mCherry-3xBAZ2B^{WT} or mCherry-3xBAZ2B^{NF} mutant.

Supplemental Figure S11

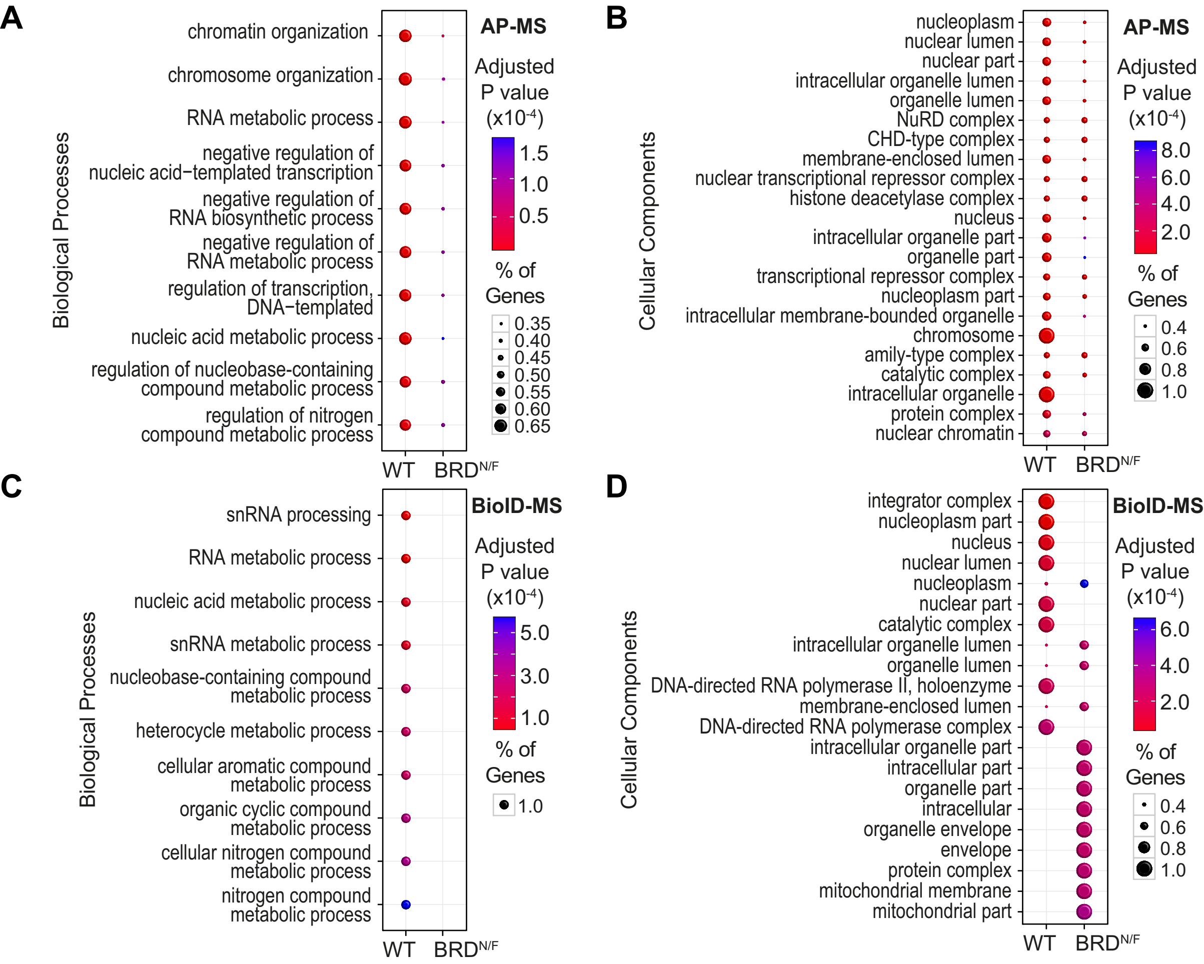

Supplemental Figure S11 – *Gene Ontology analysis of ZMYND8 (WT & BRD$^{N/F}$ mutant) proteomic networks, related to Figure 7.* (**A-D**) Enrichment of gene ontology terms following analysis of AP-MS or BioID data from full length 3x-FLAG ZMYND8 WT or BRD (BRD$^{N/F}$) mutant proteins. Spheres indicate percentage of genes participating in each enriched cluster, coloured by *P* value adjusted using the Benjamini-Hochberg false discovery rate (FDR) with a threshold of 0.05. Functional enrichment is shown for Biological Processes (AP-MS data in (A) and BioID data in (C)) as well as Cellular Components (AP-MS data in (B) and BioID data in (D)) Gene Ontology terms. The BRD mutant systematically shows reduced enrichment in several functions

Supplemental Figure S12

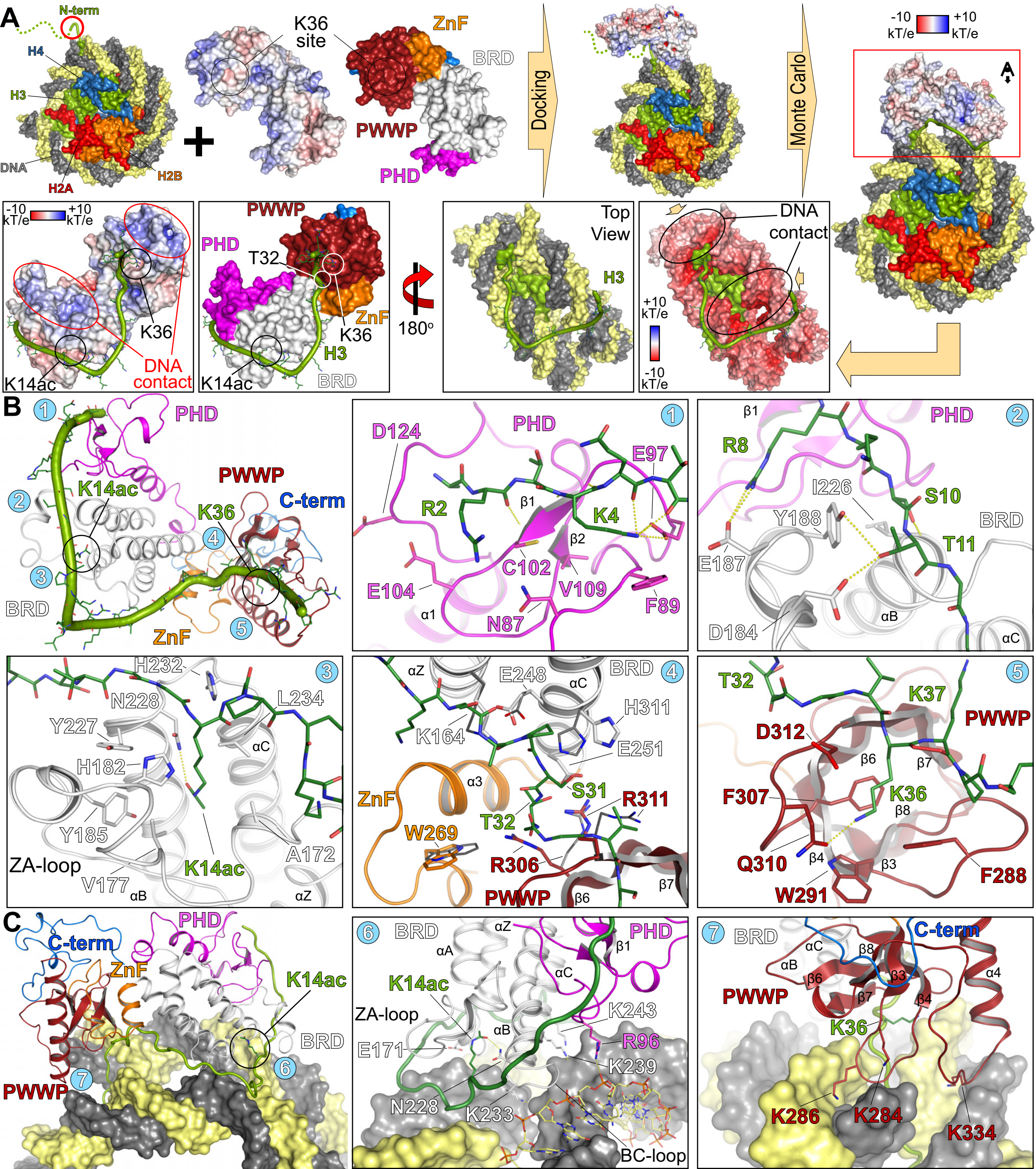

Supplemental Figure S12 – *Molecular modelling of the ZMYND8/nucleosome interaction*, *related to Figure* 7. (**A**) ZMYND8 was docked onto the core nucleosome particle (coloured as indicated in the inset – model taken from PDB: 3AFA) in a stepwise fashion, first by docking its reader ensemble onto the nucleosome while restraining K36 within the aromatic PWWP cage, then by building an H3 tail covering residues 1-36 on top of the reader ensemble by tethering and restraining its N-terminal part (ARTK) within the PHD finger cavity, K14ac within the BRD cavity, T32 within the cavity at the interface of the PWWP and BRD modules and K36 within the aromatic cage of the PWWP domain (top panel). Subsequent energy minimization by Monte Carlo simulation converged towards a low-energy docked structure whereby the reader ensemble was found positioned on the nucleosome core with the predicted DNA-face of the molecule directly initiating interactions with the nucleosomal DNA, while the histone H3 tail was bound on the PHD, BRD and PWWP face of the structure. A side view of this complex mode of interaction is shown on the left panel. A top-down view is split into two view-points rotated by 180˚ compared to each other (right panel) highlighting domain location (top) or electrostatic properties (bottom). The H3 tail is wrapped around the reader modules which in turn are initiating direct contacts with the DNA backbone, suggesting that ZMYND8 acts as both nucleosome and histone binder. (**B**) The N-terminal portion of the H3 peptide retained mainly backbone interactions with the PHD domain, while R2 was found in a charged environment (N87, E104 and D124) and K4 packed between C102, V109 and F89 while forming a salt bridge to E97 (Panel 1). R8 engaged directly E187 on the BRD site via a salt bridge, and S10/T11 were accommodated on top of the BRD surface, engaging the protein either via backbone contacts (S10 with I226) or directly via hydrogen bonds to Y188 and D184 (Panel 2). Acetylated K14 inserted into the BRD cavity and directly engaged the conserved asparagine (N228) while packing between H232, L234, H182, Y185 and V177 (Panel 3). S31 was found in proximity to H311, while T32 inserted in a cavity formed between the PWWP, BRD and ZnF domains directly engaging E251 (BRD), R306 and R313 (PWWP) (Panel 4). This pose was highlighted by substantial side chain rearrangement in the T32 immediate environment (R306, R313 on the PWWP domain; W269 on the ZnF; K164, H247, E248 on the BRD domain), and we wondered if this would provide additional space for accommodating a bulkier phosphorylation on T32. Unmodified K36 inserted into the hydrophobic pocket of the PWWP domain, following side chain rotations on F288 as well as D312, with the latter forming a salt bridge to K36 (Panel 5). (**c**) Binding to the entire histone tail was accompanied by close interactions of the reader ensemble with the DNA wrapped around the core histones both on the PHD/BRD as well as the PWWP sites R96 on the PHD site contacted one DNA strand while a combination of histone interactions and direct DNA contact with the second stand via D171 on the BRD site resulted in a deep insertion of the BRD BC-loop between the two DNA turns and K233 initiating backbone phosphate contacts, positioning at the same time a charged face of

the BRD C-helix (N236, K239 and K243) in direct contact with the DNA backbone (Panel 6). On the PWWP site, the loop between sheets β3 and β4 inserted K286 into the minor groove of one DNA turn with K286 close to the phosphate backbone, while the loop between sheet β8 and helix α4 inserts between two DNA turns, allowing K334 to direct contact with the phosphate backbone (Panel 7). ZMYND8 reader domains and their residues are coloured as follows: PHD domain, magenta; BRD domain, white; ZnF domain, orange; PWWP domain, dark red; C-terminal tail, blue. Nucleosome elements are also shown with the histone H3 N-terminal tail coloured in green and the DNA strands shown as surfaces (yellow and grey respectively).

Supplemental Tables

Supplemental Table S1 - Histone Arrays - Peptide Sequences (K14 and K36 SPOT arrays) used in the membranes of **Figure 2**

Histone H3<br>Peptide Length (1 - 21)	Histone	Array Position	Peptide Sequence
CTRL	H3	**A1**	HHHHHHHHHHH
CTRL	H4	**A2**	HHHHHHHHHHH
	H5	**A3**	
WT	H6	**A4**	ARTKQTARKSTGGKAPRKQLA
K14ac	H7	**A5**	ARTKQTARKSTGG**Kac**APRKQLA
K4ac/K14ac	H8	**A6**	ART**Kac**QTARKSTGG**Kac**APRKQLA
K4me/K14ac	H9	**A7**	ART**Kme**QTARKSTGG**Kac**APRKQLA
K4me2/K14ac	H10	**A8**	ART**Kme$_2$**QTARKSTGG**Kac**APRKQLA
K4me3/K14ac	H11	**A9**	ART**Kme$_3$**QTARKSTGG**Kac**APRKQLA
K9ac/K14ac	H12	**B1**	ARTKQTAR**Kac**STGG**Kac**APRKQLA
K9me/K14ac	H13	**B2**	ARTKQTAR**Kme**STGG**Kac**APRKQLA
K9me2/K14ac	H14	**B3**	ARTKQTAR**Kme$_2$**STGG**Kac**APRKQLA
K9me3/K14ac	H15	**B4**	ARTKQTAR**Kme$_3$**STGG**Kac**APRKQLA
	H16	**B5**	
K4ac/K9ac/K14ac	H17	**B6**	ART**Kac**QTAR**Kac**STGG**Kac**APRKQLA
K4ac/K9me/K14ac	H18	**B7**	ART**Kac**QTAR**Kme**STGG**Kac**APRKQLA
K4ac/K9me2/K14ac	H19	**B8**	ART**Kac**QTAR**Kme$_2$**STGG**Kac**APRKQLA
K4ac/K9me3/K14ac	H20	**B9**	ART**Kac**QTAR**Kme$_3$**STGG**Kac**APRKQLA
K4me/K9ac/K14ac	H21	**C1**	ART**Kme**QTAR**Kac**STGG**Kac**APRKQLA
K4me/K9me/K14ac	H22	**C2**	ART**Kme**QTAR**Kme**STGG**Kac**APRKQLA
K4me/K9me2/K14ac	H23	**C3**	ART**Kme**QTAR**Kme$_2$**STGG**Kac**APRKQLA
K4me/K9me3/K14ac	H24	**C4**	ART**Kme**QTAR**Kme$_3$**STGG**Kac**APRKQLA
	H25	**C5**	
K4me2/K9ac/K14ac	H26	**C6**	ART**Kme$_2$**QTAR**Kac**STGG**Kac**APRKQLA
K4me2/K9me/K14ac	H27	**C7**	ART**Kme$_2$**QTAR**Kme**STGG**Kac**APRKQLA
K4me2/K9me2/K14ac	H28	**C8**	ART**Kme$_2$**QTAR**Kme$_2$**STGG**Kac**APRKQLA
K4me2/K9me3/K14ac	H29	**C9**	ART**Kme$_2$**QTAR**Kme$_3$**STGG**Kac**APRKQLA
K4me3/K9ac/K14ac	H30	**D1**	ART**Kme$_3$**QTAR**Kac**STGG**Kac**APRKQLA
K4me3/K9me/K14ac	H31	**D2**	ART**Kme$_3$**QTAR**Kme**STGG**Kac**APRKQLA
K4me3/K9me2/K14ac	H32	**D3**	ART**Kme$_3$**QTAR**Kme$_2$**STGG**Kac**APRKQLA
K4me3/K9me3/K14ac	H33	**D4**	ART**Kme$_3$**QTAR**Kme$_3$**STGG**Kac**APRKQLA

Histone H3.X<br>Peptide Length (22 - 42)	Histone	Array Position	Peptide Sequence
K36me3	H3.1	**A1**	TKAARKSAPATGGV**Kme$_3$**KPHRYR
K36me3	H3.1t	**A2**	TKVARKSAPATGGV**Kme$_3$**KPHRYR
K36me3	H3.3	**A3**	TKAARKSAP**S**TGGV**Kme$_3$**KPHRYR
K36me3	H3.3C	**A4**	TKAARKSTP**S**T**C**GV**Kme$_3$**PHRYR

pT32/K36me3	H3.1	**A5**	TKAARKSAPA**pT**GGV**Kme$_3$**KPHRYR
pT32/K36me3	H3.1t	**A6**	TKVARKSAPA**pT**GGV**Kme$_3$**KPHRYR
pT32/K36me3	H3.3	**A7**	TKAARKSAP**SpT**GGV**Kme$_3$**KPHRYR
pT32/K36me3	H3.3C	**A8**	TKAARKSTP**SpTC**GV**Kme$_3$**PHRYR
K36me2	H3.1	**B1**	TKAARKSAPATGGV**Kme$_2$**KPHRYR
K36me2	H3.1t	**B2**	TKVARKSAPATGGV**Kme$_2$**KPHRYR
K36me2	H3.3	**B3**	TKAARKSAP**S**TGGV**Kme$_2$**KPHRYR
K36me2	H3.3C	**B4**	TKAARKSTP**S**T**C**GV**Kme$_2$**PHRYR
pT32/K36me2	H3.1	**B5**	TKAARKSAPA**pT**GGV**Kme$_2$**KPHRYR
pT32/K36me2	H3.1t	**B6**	TKVARKSAPA**pT**GGV**Kme$_2$**KPHRYR
pT32/K36me2	H3.3	**B7**	TKAARKSAP**SpT**GGV**Kme$_2$**KPHRYR
pT32/K36me2	H3.3C	**B8**	TKAARKSTP**SpTC**GV**Kme$_2$**PHRYR
K36me	H3.1	**C1**	TKAARKSAPATGGV**Kme**KPHRYR
K36me	H3.1t	**C2**	TKVARKSAPATGGV**Kme**KPHRYR
K36me	H3.3	**C3**	TKAARKSAP**S**TGGV**Kme**KPHRYR
K36me	H3.3C	**C4**	TKAARKSTP**S**T**C**GV**Kme**PHRYR
pT32/K36me	H3.1	**C5**	TKAARKSAPA**pT**GGV**Kme**KPHRYR
pT32/K36me	H3.1t	**C6**	TKVARKSAPA**pT**GGV**Kme**KPHRYR
pT32/K36me	H3.3	**C7**	TKAARKSAP**SpT**GGV**Kme**KPHRYR
pT32/K36me	H3.3C	**C8**	TKAARKSTP**SpTC**GV**Kme**PHRYR
		**C9**	
CTRL		**C10**	HHHHHHHHHHH
K36	H3.1	**D1**	TKAARKSAPATGGV**K**KPHRYR
K36	H3.1t	**D2**	TKVARKSAPATGGV**K**KPHRYR
K36	H3.3	**D3**	TKAARKSAP**S**TGGV**K**KPHRYR
K36	H3.3C	**D4**	TKAARKSTP**S**T**C**GV**K**PHRYR
pT32/K36	H3.1	**D5**	TKAARKSAPA**pT**GGV**K**KPHRYR
pT32/K36	H3.1t	**D6**	TKVARKSAPA**pT**GGV**K**KPHRYR
pT32/K36	H3.3	**D7**	TKAARKSAP**SpT**GGV**K**KPHRYR
pT32/K36	H3.3C	**D8**	TKAARKSTP**SpTC**GV**K**PHRYR
		**D9**	(blank)
CTRL		**D10**	HHHHHHHHHHH

Supplemental Table S2: Histone Arrays - Peptide Sequences used in the membranes of **Figure 4** (separate file).

Supplemental Table S3: Synthetic biotinylated histone peptides used to probe ZMYND8 histone binding preferences, related to **Figure 2**

#	Histone	Length	modifications	Sequence
1	H3	1-20	-	Ac-ARTKQTARKSTGGKAPRKQL-K(Biot)-NH2
2	H3	15-36	-	Ac-APRKQLATKAARKSAPSTGG-Peg-Biot
3	H3	1-20	K4me	ARTKmeQTARKSTGGKAPRKQL-K(Biot)-NH2
4	H3	1-20	K4me$_3$	ARTKme$_2$QTARKSTGGKAPRKQL-K(Biot)-NH2
5	H3	15-34	K27me	Ac-APRKQLATKAARKmeSAPSTGG-Peg-Biot
6	H3	15-34	K27me$_2$	Ac-APRKQLATKAARKme$_2$SAPSTGG-Peg-Biot
7	H3	15-34	K27me$_3$	Ac-APRKQLATKAARKme$_3$SAPSTGG-Peg-Biot
8	H3	1-20	K14ac	Ac-ARTKQTARKSTGGKacAPRKQL-K(Biot)-NH2
9	H3	1-20	K4acK14ac	Ac-ARTKacQTARKSTGGKacAPRKQL-K(Biot)-NH2
10	H3	1-20	K9acK14ac	Ac-ARTKQTARKacSTGGKacAPRKQL-K(Biot)-NH2
11	H3	1-20	K14acK18ac	Ac-ARTKQTARKSTGGKacAPRKacQL-K(Biot)-NH2
12	H3	1-20	K4acK9acK14ac	Ac-ARTKacQTARKacSTGGKacAPRKQL-K(Biot)-NH2
13	H3	1-20	K9acK14acK18ac	Ac-ARTKQTARKacSTGGKacAPRKacQL-K(Biot)-NH2
14	H3	1-20	K4meK9acK14acK18ac	Ac-ARTKmeQTARKacSTGGKacAPRKacQL-K(Biot)-NH2
15	H3	1-20	K4me$_3$K14ac	Ac-ARTKme$_3$QTARKSTGGKacAPRKQL-K(Biot)-NH2
16	H3	1-20	K4me$_3$K9acK14ac	Ac-ARTKme$_3$QTARKacSTGGKacAPRKQL-K(Biot)-NH2
17	H3	1-20	K4me$_3$K9me$_3$	Ac-ARTKme$_3$QTARKme$_3$STGGKAPRKQL-K(Biot)-NH2
18	H3	1-20	K4me$_3$R8me$_{2,a}$K9me$_3$	Ac-ARTKme$_3$QTARme$_{2,a}$Kme$_3$STGGKAPRKQL-K(Biot)-NH2
19	H3.3	15-43	-	Ac-APRKQLATKAARKSAPSTGGVKKPHRY-GG-K(Biot)-NH2
20	H3.3	15-43	K36me$_3$	Ac-APRKQLATKAARKSAPSTGGVKme$_3$KPHRY-GG-K(Biot)-NH2
21	H3.3	15-43	K18me$_3$K36me$_3$	Ac-APRKme$_3$QLATKAARKSAPSTGGVKme$_3$KPHRY-GG-K(Biot)-NH2
22	H3.3	27-45	pT32	Ac-KSAPSpTGGVKKPHRYRPGT-G-K(Biot)-NH2
23	H3.3	27-45	pT32/K36me	Ac-KSAPSpTGGVKmeKPHRYRPGT-G-K(Biot)-NH2
24	H4	1-23	-	Ac-SGRGKGGKGLGKGGAKRHRKVLR-K(Biot)-NH2
25	H4	1-23	K12ac	Ac-SGRGKGGKGLGKacGGAKRHRKVLR-K(Biot)-NH2
26	H4	1-23	K16ac	Ac-SGRGKGGKGLGKGGAKacRHRKVLR-K(Biot)-NH2
27	H4	1-23	K12acK16ac	Ac-SGRGKGGKGLGKacGGAKacRHRKVLR-K(Biot)-NH2

Supplemental Table S4 – Isothermal Titration Calorimetry of human ZMYND8 reader ensemble with recombinant 40-amino-acid long histone H3 peptides bearing AMBER codon deposited acetyl-lysine residues. Titrations were carried out in 20 mM HEPES pH 7.5 (at 25 °C), 200 mM NaCl and 15 °C while stirring at 1000 rpm. Peptides were titrated into protein solutions. Data related to **Figure 2**.

	Peptide	[P] (µM)	[Pep] (µM)	K_D (µM)	ΔH^{obs} (kcal/mol)	N	$T\Delta S$ (kcal/mol)	ΔG (kcal/mol)
**H3.1**	WT	30	777	19.0 ± 0.8	-7.42 ± 0.26	0.99 ± 0.028	-1.20	-6.22
	T32E	31	789	20.7 ± 0.9	-8.02 ± 0.26	1.05 ± 0.027	-1.84	-6.18
	K14ac	31	790	15.1 ± 0.6	-12.02 ± 0.37	0.79 ± 0.020	-5.64	-6.38
	K14ac/T32E	31	795	17.5 ± 0.8	-13.15 ± 0.47	0.81 ± 0.024	-6.88	-6.27
**H3.3**	WT	30	720	19.6 ± 0.8	-8.00 ± 0.26	1.03 ± 0.026	-1.79	-6.21
	T32E	29	761	17.4 ± 1.1	-8.62 ± 0.43	1.00 ± 0.040	-2.34	-6.28
	K14ac	27	800	6.8 ± 0.2	-16.49 ± 0.19	0.87 ± 0.079	-9.68	-6.81
	K14ac/T32E	32	779	6.2 ± 0.2	-17.26 ± 0.19	0.83 ± 0.069	-10.40	-6.86
	polyKac	29	471			NB		
	K14ac (20-mer)	29	742	6.6 ± 0.2	-22.88 ± 0.25	0.83 ± 0.070	-16.04	-6.84
	K14ac (mBRD)[a]	30	785	12.2 ± 0.3	-2.70 ± 0.05	0.95 ± 0.013	3.77	-6.47
	K14ac (mPHD)[b]	34.1	772			NB		
	K14ac (mPHD/BRD)[c]	35.4	772			NB		
	K14ac (mPWWP)[d]	27	761	8.6	-15.84 ± 0.72	0.49 ± 0.019	-9.16	-6.68
	K14ac/T32E (mPWWP)[d]	27	761	23.0	-22.61 ± 0.86	0.88 ± 0.029	-16.47	-6.14

[a] mBRD: BRD mutant (N228F); [c] mPHD/BRD: PHD+BRD mutant (N87A, E104A, D124A, N228F)
[b] mPHD: PHD mutant (N87A, E104A, D124A); [d] mPWWP: PWWP mutant (F288A, W291A)

Supplemental Table S5 – Isothermal Titration Calorimetry of human ZMYND8 reader ensemble with recombinant 40-amino-acid long histone H4 peptides bearing AMBER codon deposited acetyl-lysine residues. Titrations were carried out in 20 mM HEPES pH 7.5 (at 25 °C), 200 mM NaCl and 15 °C while stirring at 1000 rpm. Peptides were titrated into protein solutions. Data related to **Figure 2**.

	Peptide	[P] (μM)	[Pep] (μM)	K_D (μM)	ΔH^{obs} (kcal/mol)	N	TΔS (kcal/mol)	ΔG (kcal/mol)
**H4**	WT	30	1025			NB		
	K8ac	33	814			NB		
	K12ac	29	760	31.6 ± 5.0	-2.33 ± 0.59	0.83 ± 0.179	3.41	-5.74
	K16ac	30	736			NB		
	poly-Kac	32	805	37.7 ± 1.2	-5.65 ± 0.24	1.00 ± 0.036	0.18	-5.83
	K12ac (mBRD)[a]	37	727			NB		
	K12ac (mPHD)[b]	41	706	35.0 ± 2.2	-2.51 ± 0.20	0.75 ± 0.048	3.37	-5.88

[a] mBRD: BRD mutant (N228F); [b] mPHD: PHD mutant (N87A, E104A, D124A)

Supplemental Table S6 – Isothermal Titration Calorimetry of human BAZ2B (UniProt: Q9UIF8 residues 2054-2168 WT or N2140F mutant) with recombinant histone H3 peptides bearing K14ac. Titrations were carried out in 20 mM HEPES pH 7.5 (at 25 °C), 200 mM NaCl and 15 °C while stirring at 1000 rpm. Peptides were titrated into protein solutions. Data related to **Figure 5**.

BAZ2B	Peptide	[P] (μM)	[Pep] (μM)	K_D (μM)	ΔH^{obs} (kcal/mol)	N	$T\Delta S$ (kcal/mol)	ΔG (kcal/mol)
**WT**[¥]	$H3_{(9-19)}$K14ac	50	1550	7.6 ± 0.3	-8.91 ± 0.08	0.99 ± 0.007	-2.27	-6.64
**WT**[‖]	$H3_{(5-25)}$K14ac	40	1400	6.3 ± 0.2	-6.86 ± 0.06	1.00 ± 0.007	-0.12	-6.74
**WT**	$H3_{(1-20)}$(no Kac)	60	750			NB		
**WT**	$H3_{(1-20)}$K14ac	60	750	17.3 ± 0.9	-4.22 ± 0.15	0.94 ± 0.026	2.06	-6.28
**N2140F**	$H3_{(1-20)}$K14ac	60	750			NB		

[¥] data from (Filippakopoulos et al., 2012)
[‖] data from (Philpott et al., 2011)

Supplemental Table S7: Data collection and refinement statistics, related to the crystal structure shown in **Figure 1**.

Data Collection		
PDB	4COS	
Protein/Ligand	ZMYND8	ZMYND8 (Zn SAD)
Beamline	DLS I04	DLS I03
Wavelength	0.9796	1.2829
Space group	$P2_12_12_1$	$P2_12_12_1$
Cell dimensions: a, b, c (Å)<br>α, β, γ (deg)	66.96 68.83 70.35<br>90.00 90.00 90.00	66.06 69.01 70.34<br>90.00 90.00 90.00
Resolution* (Å)	1.67 (1.77-1.67)	1.78 (1.83-1.78)
Unique observations*		
Completeness* (%)	99.9 (99.7)	99.5 (98.8)
Redundancy*	6.0 (4.5)	12.5 (11.7)
R_{sym}*	0.077 (0.767)	0.115 (0.990)
I/ σI*	12.2 (2.0)	18.5 (3.6)
**Refinement**		
Resolution (Å)	1.67	
R_{work} / R_{free} (%)	17.2/21.0	
Number of atoms (protein/other/water)	2553/15/333	
B-factors ($Å^2$) (protein/other/water)	31.5/59.7/40.2	
r.m.s.d bonds (Å)<br>r.m.s.d angles (^{o})	0.015<br>1.661	
Ramachadran Favoured (%)<br>Allowed (%)<br>Disallowed (%)	97.09<br>2.59<br>0.32	

* Values in parentheses correspond to the highest resolution shell.

Rsym is the unweighted R-value on I between symmetry mates.

Rwork = Σhkl | |Fobs (hkl)| - k |Fcalc (hkl)| | / Σhkl |Fobs (hkl)| for the working set of reflections

Rfree is the R-value for 5% of the reflections excluded from refinement.

Supplemental Table S8: Primers used for EMSA assays related to **Figure 4**

DNA Probe	Primer	Sequence
AT-rich	fwd	CATGTTTTAATAGTATCTGCAATTTGTAATATGTCTA**c**atg
	rvs	gta**c**AAAATTATCATAGACGTTAAACATTATACAGATGTAC
GC-rich	fwd	CATGGCGGGGCCCCGCGACGCGGCTGCCTGCGGGGTG**c**atg
	rvs	gta**c**CGCCCCGGGGCGCTGCGCCGACGGACGCCCCACGTAC

fwd: forward primer; **rvs**: reverse primer;

Supplemental Table S9: Primers used ChIP-PCR experiments related to **Figure 5**.

Gene	NCBI		Sequence (5' →3')	Tm (˚C)	Location	Transcript Length
CLCN7	NM_001287	fwd	AAGAACAGAAGGCGAGT	50.3	chr16 1525587-1525658	72
		rvs	CAGTTTCCAGAGGCGAC	55.0		
CREBBP	NM_004380	fwd	GTTACAGACCGCCCTTG	54.9	chr16 3931258-3931342	85
		rvs	GACCACTTTGGAGCAGTTG	56.3		
ETS2	NM_001256295	fwd	CCTTATTACCCAAGCCCG	58.0	chr21 40177294-40177382	89
		rvs	CCCTAACGCCAAACCTG	57.6		
PPP4C	NM_002720	fwd	TGAAAGAGGGAGGCAGG	57.3	chr16 30087365-30087437	73
		rvs	TAGAGGAGTCTCGCACTATTAC	53.2		
SLC7A5	NR_002594	fwd	TATGTCGGGCATTCCTCT	56.5	chr16 87904472-87904572	101
		rvs	ACAAGTTAGTATCTGTGTGACC	51.0		

fwd: forward primer; **rvs**: reverse primer

Online Methods

Cloning and Mutagenesis

cDNA encoding the full length ZMYND8 (Uniprot: Q9ULU4, isoform 13) was reconstructed following the Gibson assembly protocol (Gibson et al., 2010; Gibson et al., 2009) from a PCR fragment spanning residues T95-D1188, amplified from the MGC cDNA clone (IMAGE 4844473) and oligonucleotides encoding the first 94 amino-acids that were missing in the MGC clone. The construct was subsequently cloned into a pDONR221 vector. Single point mutations or their combinations were introduced into the full length ZMYND8 Gateway entry clone using 15 cycles of the QuikChange II PCR protocol (Agilent Technologies). Full length ZMYND8 mammalian expression vectors encoding either an N-terminal 3x-FLAG, EGFP or BirA*-FLAG tags were constructed following the Gateway LR recombination reaction between relevant destination vectors previously described (Lambert et al., 2014) and wild type or mutated ZMYND8 Gateway entry clones. Wild type or mutated forms of the N-terminal reader modules of ZMYND8 (PHD, Bromo and PWWP domains, aa Q83-S406) for bacterial expression were amplified by PCR and sub-cloned into the pNIC-ZB vector (Genbank: GU452710) using the ligation-independent cloning (LIC) protocol (Aslanidis and de Jong, 1990).

Gateway entry clones containing three copies of either the wild type or N2140F substituted bromodomain of BAZ2B (S2054 – S2168 of Uniprot accession Q9UIF8) and C-terminal NLS sequence from the SV40 Large T-antigen (PKKKRKV) were constructed by multiple ligation independent cloning (LIC) protocol (Aslanidis and de Jong, 1990) in the pENTR-221 vector (Invitrogen). The GFP, mCherry or 3*HA tagged 3x-BAZ2B bromodomain constructs were constructed by Gateway LR recombination reaction between appropriate destination vector and an entry clone encoding for a wild type or mutated triple bromodomains.

Generation of Stable Cell Lines

Constructs for the genes of interest were generated via Gateway cloning into pDEST 5' Triple-FLAG-pcDNA5-FRT-TO or pDEST 5' BirA*-FLAG-pcDNA5-FRT-TO vectors. Proteins of interest were stably expressed in T-REx Flp-In HEK293 cells as previously described (Couzens et al., 2013).

Protein Expression and Purification

Recombinant wild type or mutated ZMYND8 (Q83-S406) proteins were expressed and purified as previously described (Savitsky et al., 2010). Briefly, E.coli BL21(DE3) cells containing a pRARE2 plasmid were transformed with a pNIC-ZB vector encoding the N-terminal triple reader domains of ZMYND8. Small (50 ml) cultures inoculated with several colonies were grown overnight at 37 ˚C in terrific broth (TB) media and were used to inoculate 2 l of TB with K/Na phosphates substituted with 5 g/l NaCl to prevent $ZnCl_2$ precipitation at 37 ˚C with aeration until the optical density (λ = 600 nm) reached a value of 3. The temperature was reduced to 18 ˚C and the cultures were allowed to cool down for 1 h before 100 µM of isopropyl β-D-1-thiogalactopyranoside (IPTG) and 1 mM of $ZnCl_2$ were added (final concentrations) to induce protein expression overnight. Cultures were harvested and cell pellets were resuspended in lysis buffer (50 mM HEPES, pH 7.5, 500 mM NaCl, 0.5 mM tris(2-carboxyethyl)phosphine (TCEP) and 5 % w/v glycerol) and mechanically lysed following 3 passes through an Avestin C5 high pressure homogeniser at 4 ˚C. The lysates were span down and supernatants were collected and loaded onto 5 ml gravity flow Ni-IDA columns. Each column was washed with at least 10 column volumes of lysis buffer containing 50 mM imidazole and eluted with elution buffer (20 mM HEPES, pH7.5, 250 mM NaCl, 300 mM imidazole, 5 % w/v glycerol, 0.5 mM TCEP) in 10 ml fractions. Fractions containing recombinant ZMYND8 protein were directly loaded onto an HP SP column on an AKTA Purifier, were eluted with a 0.25 – 1 M NaCl gradient and were combined. TCEP was added to a final concentration of 20 mM and the Z-Basic (ZB) tag was removed by overnight incubation at 4 ˚C with TEV protease (at 1:100 w/w). Following tag cleavage, the protein was re-purified through a Ni-IDA column in order to remove the ZB tag. The flow-through fraction was collected, concentrated and loaded onto a Superdex S75 16/60 column equilibrated with GF buffer (50 mM HEPES, pH 7.5, 200 mM NaCl, 2 % w/v glycerol, 20 mM TCEP). Recombinant ZMYND8 eluted as a single symmetrical monomeric peak, fractions were pooled and were either flash frozen in liquid nitrogen and stored in -80C freezer or used directly in crystallisation or biophysical experiments. All purification steps were performed on ice or in a cold room at 4 ˚C.

Custom Peptide Synthesis

Custom histone peptides carrying specific modifications were synthesised as previously described (Rothbart et al., 2012b). Sequences are tabulate in **Supplemental Table S3**.

Recombinant Histone Peptides

Histone H3 and H4 peptides encoding the N-terminal portion of the proteins (20 or 40 amino-acid long) were amplified in a bacterial expression system using a pCDFDuet™-1 (Novagen) plasmid carrying the ORF for the histone-tail fragments of interest, in order to yield sufficient amounts for biophysical characterization of histone interactions with ZMYND8. In brief, all peptides were expressed as his-tagged proteins and were purified by nickel affinity and size exclusion chromatography. Peptide integrity was assessed by SDS-PAGE and electro-spray mass spectrometry on an Agilent 6530 QTOF (Agilent Technologies Inc. Palo Alto, CA) mass spectrometer. Poly-acetylation of the peptides was performed essentially as previously described (Smith, 2005). In addition, histone peptides carrying specific acetylations (histone H3 at K14; histone H4 at K8 or K12 or K16) were produced *in vivo* using the amber stop codon/suppressor tRNA technology developed by Neumann and colleagues (Neumann et al., 2009) with several modifications that increased overall yields from 10% to above 70 %. In brief, ORF for the histone-tail fragments of interest presenting an amber codon (TAG) engineered at the desired sites were co-expressed in Tuner™ BL21 DE3 cells (Novagen, #70622) with an orthogonal N(epsilon)-acetyl-lysyl-tRNA synthetase/tRNA(CUA) pair, leading to a site specific incorporation of N(epsilon)-acetyl-lysine. Cells were grown at 37 ˚C in Terrific Broth (TB) medium previously inoculated with 10 ml of an overnight culture.

At OD_{600} of ~0.7 the culture was supplemented with 20 mM nicotinamide (NAM, Sigma, #N3376-100G) and 25 mM acetyl-lysine (H-Lys-Ac-OH, GL Biochem Shangai Ltd, #GLS140325). Protein expression was induced 30 min later by addition of 50 µM IPTG and cells were harvested by centrifugation (8,700 x *g*, 15 min, 4 ˚C; Beckman Coulter Avanti J-20 XP centrifuge) after 6 h at 37 ˚C. Peptides were then purified as described above.

SPOT Peptide Assays

Cellulose-bound peptide arrays were prepared employing Fmoc solid phase peptide synthesis using a MultiPep-RSi-Spotter (INTAVIS, Köln, Germany) according to the SPOT synthesis method

provided by the manufacturer as previously described (Picaud and Filippakopoulos, 2015). Human histone peptides (using UniProt accession codes Q6FI13 (histone H2A), P16104 (histone H2A.X), P0C0S5 (histone H2A.Z), P62807 (histone H2B), P68431 (histone H3.1), Q16695 (histone H3.1t), P84243 (histone H3.3), Q6NXT2 (histone H3.3C) and P62805(histone H4)) were synthesized on amino-functionalized cellulose membranes (Whatman™ Chromatography paper Grade 1CHR, GE Healthcare Life Sciences #3001-878) and the presence of SPOTed peptides was confirmed by ultraviolet light (UV, λ = 280 nM). The assay was performed as previously described (Filippakopoulos, 2012) using the his-6-tagged recombinant triple readers of ZMYND8 as bait protein. ZMYND8 protein bound to peptides was detected using antibody HPR conjugated (Novagene, # 71841) and the Pierce® ECL Western blotting Substrate (Thermo Scientific, # 32106). Chemiluminescence was detected with an image reader (Fujifilm LAS-4000 ver.2.0) typically using an incremental exposure time of 5 min for a total of 80 min (or until saturation was reached, in the case of very strong signal). Peptide locations on the arrays and their sequences are given in **Supplemental Tables S1 and S2**.

Biolayer Interferometry (BLI)

Experiments were performed on an Octet RED384 system (FortéBio) at 25 ˚C in 20 mM HEPES, pH 7.5, 200 mM NaCl and 20 mM TCEP using the FortéBio data acquisition software V.7.1.0.100. Biotinylated peptides were first immobilized onto Super Streptavidin biosensors (SuperStreptavidin (SSA) Dip and Read Biosensors for kinetic #18-0011, FortéBio), pre-equilibrated in the BLI buffer then quenched in a solution of 5 µM Biotin. (baseline equilibration 60 sec, peptide loading for 240 sec, quenching for 60 sec, 1000 x rpm shake speed, at 25 ˚C). The immobilized peptides were subsequently used in association and dissociation measurements performed within a time window of 600 sec (base line equilibration 120 sec, association for 240 sec, dissociation for 240 sec, 1000 x rpm shake speed, at 25 ˚C). Interference patterns from peptide-coated biosensors without protein were used as controls. After referencing corrections, the subtracted binding interference data were analyzed using the FortéBio analysis software (FortéBio data analysis software V.7.1.0.38) provided with the instrument following the manufacturer's protocols. Histone peptide binding to ZMYND8 was first explored against a set of biotinylated histone peptides commercially available (AltaBioSciences Histone array, Set 3 & Set 4 Histone Acetyl-Lysine library) carrying 21-mers with

single or multiple modifications, including lysine acetylation and methylation. Custom made histone H3 and H4 20-amino-acid long peptides with specific modification were then tested by employing a range of ZMYND8 concentrations (1, 2, 4, 8, 16, 32, 64 and 128 μM) in order to determine binding constants. Peptide sequences are given in **Supplementary Table S3**.

Isothermal Titration Calorimetry (ITC)

Experiments were carried out on an ITC200 titration microcalorimeter from MicroCal™, LLC (GE Healthcare) equipped with a Washing module, with a cell volume of 0.2003 ml and a 40 μl microsyringe. Experiments were carried out at 15 ˚C while stirring at 1000 rpm, in ITC buffer (20 mM HEPES pH 7.5 (at 25 °C), 200 mM NaCl). The microsyringe was loaded with a solution of peptide sample (470 - 1100 μM, in ITC buffer) and was carefully inserted into the calorimetric cell which was filled with an amount of the protein (0.2 ml, 27-33 μM in ITC buffer). Following baseline equilibration an additional delay of 60 sec was applied. All titrations were conducted using an initial control injection of 0.3 μl followed by 38 identical injections of 1 μl with a duration of 2 sec (per injection) and a spacing of 120 sec between injections. The titration experiments were designed as to ensure complete saturation of the proteins before the final injection. The heat of dilution for the peptides were independent of their concentration and corresponded to the heat observed from the last injection, following saturation of ligand binding, thus facilitating the estimation of the baseline of each titration from the last injection. The collected data were corrected for peptide heats of dilution (measured on separate experiments by titrating the proteins into ITC buffer) and deconvoluted using the MicroCal™ Origin software supplied with the instrument to yield enthalpies of binding (ΔH) and binding constants (K_B) in the same fashion to that previously described in detail by Wiseman and co-workers (Wiseman et al., 1989). Thermodynamic parameters were calculated using the basic equation of thermodynamics ($\Delta G = \Delta H - T\Delta S = -RT\ln K_B$, where ΔG, ΔH and ΔS are the changes in free energy, enthalpy and entropy of binding respectively). In all cases a single binding site model was employed, supplied with the MicroCal™ Origin software package. Dissociation constants and thermodynamic parameters are listed on **Supplementary Tables S4** and **S5**. Thermodynamic parameters for BAZ2B interactions with H3K14ac are listed in **Supplementary Table S6**.

Crystallization

Aliquots of the purified protein were set up for crystallization using a mosquito® crystallization robot (TTP Labtech, Royston UK). Coarse screens were typically setup onto Greiner 3-well plates using three different drop ratios of precipitant to protein per condition (100+50 nl, 75+75 nl and 50+100 nl). Initial hits were optimized further using Greiner 1-well plates and scaling up the drop sizes in steps. All crystallizations were carried out using the sitting drop vapour diffusion method at 4 °C. Crystals of ZMYND8 were grown by mixing 100 nl of the protein (11 mg/ml in 10 mM HEPES pH 7.5, 500 mM NaCl, 5 % Glycerol) with 50 nl of reservoir solution containing 1.8 M $(NH_4)_2SO_4$, 0.1 M MES pH 6.3 and 15 % (v/v) dioxane. Plate-like crystals appeared within several days from sitting drop plates at 4 ˚C. Prior to data collection, all crystals were transferred to a solution consisting of the precipitation buffer supplemented with 2M Li_2SO_4 and subsequently flash frozen in liquid nitrogen.

Data Collection and Structure Determination

Zn-SAD data were collected at Diamond Lightsource from one crystal close to the Zn K-edge at 1.2829 Å on beamline I03 and a second crystal was used to obtain higher resolution on beamline I04 at a wavelength of 0.9796 Å. Both datasets were integrated with XDS (Kabsch, 2010) and scaled with SCALA or AIMLESS (Evans and Murshudov, 2013). SHELXD (Sheldrick, 2010) was used to identify 3 putative Zn sites in the initial dataset. The electron density map after phase refinement with autoSHARP (Vonrhein et al., 2007) and subsequent density modification with SOLOMON (Abrahams and Leslie, 1996) was of excellent quality. Automated model building with BUCCANEER (Cowtan, 2006) resulted in a more than 90 % complete model, which was from then refined against the higher resolution dataset. Refinement was carried out with REFMAC (Murshudov et al., 2011) and after several rounds of manual rebuilding with COOT (Emsley et al., 2010), the model converged to a final Rcryst/Rfree of 17.2 % and 21.0 %, respectively. The quality of the final model was validated with MOLPROBITY (Chen et al., 2010). Thermal motions were analyzed using TLSMD (Painter and Merritt, 2006) and hydrogen atoms were included in late refinement cycles. Data collection and refinement statistics can be found in **Supplemental Table S7**. The model and structure factors have been deposited with PDB accession code: **4COS**.

Molecular Modelling

The ZMYND8 structure was manually positioned on the core nucleosome structure (PDB code 3AFA) (Tachiwana et al., 2010) so that the aromatic cage of the PWWP domain overlapped with the expected location of H3K36. While electron density for H3K37 is present in the experimental nucleosome structure, no density is available for H3K36. The manually positioned model was further optimized in the internal coordinates space with ICM (version 3.8-2) with loose distance restraints (10 Å < d < 20 Å) imposed between the side-chains of W291, Q310, F307 of ZMYND8 and the phosphate groups of T6, C84 (of strand i) and T292 (of strand j) on the nucleosome respectively, during two independent rigid docking Monte Carlo energy minimization cycles of 1.5 million steps each that converged towards the same low-energy docked conformation. Next, a histone H3K14ac peptide tail (residues 1 to 36) was docked with ICM to the nucleosome-ZMYND8 model with the following constraints: the epsilon nitrogen of H3K36 was restrained in the PWWP aromatic cage, the side-chain of T32 was restrained in the cavity at the interface of the PWWP and BRD domains (methyl group of T32 less than 5 Å from the A262 side-chain), the side-chain of K14ac was restrained in the bromodomain binding pocket (acetyl oxygen of K14 less than 5 Å from the amide nitrogen of N228) and the side-chain of A1 and K4 were restrained at the PHD finger (A1 side-chain less than 5 Å from P121 and K4 side-chain less than 5 Å from F89). A 36 million step Monte Carlo energy minimization simulation was conducted at this step with fully flexible H3 and flexible ZMYND8 side-chains.

In order to merge the histone H3 tail (docked to the nucleosome-ZMYND8 complex) with the experimental structure of the nucleosomal H3 core, a full-length H3 structure was built, residues 1 to 36 were tethered to the docking model, residues 39 to 135 were tethered to the H3 core (using PDB code 3AFA) and the energy of the system was again relaxed with ICM. Finally, a phosphate group was added to T32 and the energy of the system was minimized with the ICM "loop" algorithm where the loop was defined as residues 29 to 34 of H3.

Chromatin Fractionation Assay

Chromatin fractionation assays were performed with HEK293T cells transfected with 1 µg of the indicated plasmids as previously described (Rothbart et al., 2012a). Cells were collected 36 h post-transfection, and western blots were performed with 8 µg of protein. The following antibodies were

used to assess ZMYND8 chromatin association: Flag (Sigma), H4 (Abcam), and b-tubulin (Cell Signaling Technologies).

Cell Culture

Flp-In T-REx HEK293 cells, stably expressing full length 3xFLAG- or BirA*-FLAG-tagged ZMYND8 and 3xFLAG-ZMYND8 N228F mutant, were maintained in DMEM, GlutaMAX^{TM} supplement (Cat.#10566-016; Gibco), supplemented with 10 % heat-inactivated foetal bovine serum (FBS - Sigma), 100 U/ml penicillin (Gibco), 100 µg/ml streptomycin (Gibco), and 200 µg/ml Hygromycin (Invitrogen). Cells were grown at 37 °C in a humidified cabinet at 5 % CO_2. Protein expression was induced by treatment of cells with 1 µg/ml Tetracycline for 24 h.

Histone Immuno-precipitation

Cells were lysed on ice in a buffer containing 10 mM Tris-HCl pH 8.0, 150 mM NaCl, 1 mM EDTA, 0.5 % NP-40, 1 mM PMSF (P7626, Sigma) and protease inhibitor cocktail (#539134, Calbiochem). Lysates were treated with 100 U of TurboNuclease (T4330, Sigma) and 1 mM $MgCl_2$ for 1 h at 4 °C to further digest chromatin. Finally lysates were centrifuged at 21,130xg for 30 min at 4 °C, and the supernatant was collected. Co-immunoprecipitations were performed with anti-FLAG M2 antibody (F1804, Sigma) and Dynabeads Protein G (10003D, Life Technologies). Cell lysates were mixed with 1 µg of anti-FLAG M2 and 10 µl of Dynabeads Protein G, and incubated 2 h at 4 °C (constant rotation). The beads were washed with washing buffer 1 (20 mM Tris-HCl pH 7.5, 500 mM NaCl, 5 mM EGTA and 1 % Triton X-100) and washing buffer 2 (20 mM Tris-HCl pH 7.5, 150 mM NaCl and 5 mM EGTA). Samples were then dissolved for electrophoresis in Laemmli sample buffer (300 mM Tris-HCl pH6.8, 10 % SDS, 50 % glycerol and 0.05 % Bromophenol blue) and incubated at 98 °C for 3 min. Samples were fractionated by electrophoresis in a 4-12 % Bis-Tris Midi Protein Gels (WG1403, Life technologies) and transferred to a PVDF membrane (10600023, GE healthcare). The membrane was blocked for 1 h with 5 % semi-skimmed milk in T-TBS and was then immunoblotted with indicated primary antibodies overnight at 4 °C in 5 % BSA (A7906, Sigma) in T-TBS at a 1:2000 dilution. After 3 washes with T-TBS the membrane was incubated with anti-rabbit (7074, Cell Signalling) or anti-mouse (A4416, Sigma) HRP-linked antibodies at 1:5000 dilution in 5 % BSA in T-TBS for 2 h at room temperature. After 5-10 washes for 2 h with T-TBS the membrane was incubated with ECL (RPN2209, GE healthcare), and exposed to film.

Antibodies used for immunoblotting: Histone H3 (ab1791, Abcam); H3K4me (pAb-194-050, Diagenode); H3K4me2 (ab7766, Abcam); H3K4me3 (pAb-003-050, Diagenode); H3K9me2 (ab1220, Abcam); H3K9me3 (ab8898, Abcam); H3K9ac (07-352, Millipore); H3K14ac (ab52946, abcam); H3K27me3 (07-449, Millipore); H3K36me (ab9048, Abcam); H3K36me2 (07-369-I, Millipore); H3K36me3 (Lys36) (ab9050, Abcam); H3pT32 (ab4076, Abcam); H3pS10 (ab5176, Abcam); Histone H4 (05-858, Millipore); H4K5ac (07-327, Millipore); H4K8ac (ab15823, Abcam); H4K12ac (07-595, Millipore); H4K16ac (07-329, Millipore); H4K20ac (61531, Active Motif).

Fluorescent Recovery After Photo-bleaching (FRAP)

FRAP experiments were performed using a protocol modified from previous studies (Filippakopoulos et al., 2010). Briefly, U2OS Flp-In/T-REx cells were transfected (FuGENE6, Qiagen) with mammalian over-expression constructs encoding GFP chimeras with WT or mutant ZMYND8 (BRD mutant: N228F; PWWP mutant: F288A/W291A; BRD/PWWP mutant: N228F + F288A/W291A; PHD/BRD/PWWP mutant: E104A/N87A/D124A, N228F, F288A/W291A). Transfected cells were plated onto an 8-well imaging chamber (Miltenyi Biotec) and pre-sensitized with 10 µM 5-bromo-2'-deoxyuridine (BrdU) for 20-40 h. The FRAP and imaging system consisted of a Zeiss LSM 710 scan-head (Zeiss GmbH, Jena, Germany) coupled to an inverted Zeiss Axio Observer Z1 microscope equipped with a high-numerical-aperture (N. A. 1.40) 63x oil immersion objective (Zeiss GmbH, Jena, Germany) equipped with an incubator XLmulti S1 set to 37 °C and 5% CO_2. GFP fluorescence imaging was carried out with an argon-ion laser (λ = 488 nm) and with a piezomultiplier tube (PMT) detector set to detect fluorescence between 493-555 nm. A region of the nucleus was selected (3.09 μm^2) and after 10 pre-scans, the region was bleached (60 % laser power of 30 mW 405 nm diode laser; 12.61 µsec pixel dwell; 30 iterations). A time-lapse series was taken to record GFP recovery using 0.07-0.40 % of the power with an interval time of 2.0 sec. The image datasets and fluorescence recovery data were exported from the control software of the microscope (ZEN v.2.1) into Origin v.7 and analysed. The average intensity of pre-scans was normalized to 1. Mean signal values and SEM were determined from 15 cells per condition tested, over multiple experiments and *P* values were calculated using two-tailed Student's *t*-test or one-way analysis of variance followed by Dunnett's multiple comparison test.

Electrophoretic mobility shift assay (EMSA)

Oligonucleotide probes encoding AT-rich or GC-rich sequences (20 µg each – **Supplemental Table S8**) were annealed in 1 X React buffer 3 (Invitrogen) using the following PCR program: 94 ˚C, 2 min; 65 ˚C, 10 min; 37 ˚C, 10 min; 25 ˚C, 10 min. The probe (200 ng) was then labelled for 1 h at 25 ˚C in 1 X React buffer 1 (Invitrogen) supplemented with 200 µg/ml bovine serum albumin (BSA), 2 µM dithiothreitol (DTT), 200 µM each of dATP, dTTP, and dGTP, 1 µl Klenow polymerase (Invitrogen), and 1.5 µl α32-P-dCTP (15 µCi). Glycogen (20 µg) was then added, and the reaction mixture was brought up to 50 µl with deionised water. The DNA probe was extracted twice through 1 volume of phenol-chloroform and precipitated overnight in 1 volume ammonium acetate (5 M) and 6 volumes of ethanol at -20 ˚C. The probe was then recovered by centrifugation (16,000 x *g*; 30 min) and suspended in water. Purified recombinant protein encoding the three N-terminal reader domains of ZMYND8 (1 µl) was then added to 14 µl of band shift buffer (25 mM HEPES pH 7.4, 150 mM KCl, 200 µg/ml BSA, 5 µM DTT) on ice. The mixture was then incubated at 25 ˚C for 15 min with 1 µl of labelled probe and loaded onto a native gel containing 6 % acrylamide (44 % stock solution; 29:1 acrylamide:bis-acrylamide; Severn Biotech) in 0.5 X Tris/Borate/EDTA buffer (TBE, Gibco). Gels were polymerized by adding 0.09 % ammonium persulfate (APS) and N,N,N',N'-tetramethyl-ethylene diamine (TEMED). After running for 90 min at 180 V, gels were dried under vacuum for 1 h at 80 ˚C and were then exposed to autoradiography film, which was developed after overnight exposure at -20 ˚C.

Non radioactive EMSAs were run by annealing the DNA probes (20 µg each) in in oligo annealing buffer (1 M NaCl, 100 mM $MgCl_2$ and 500 mM Tris-HCl pH 8.0) using the following PCR program: 94 ˚C, 2 min; 65 ˚C, 10 min; 37 ˚C, 10 min; 25 ˚C, 10 min. The probe (500 nM) and purified recombinant protein encoding the three N-terminal reader domains of ZMYND8 (WT, PHD mutant, BRD/PWWP mutant or PHD/BRD/PWWP mutant) was then added to band shift buffer (25 mM HEPES pH 7.4, 150 mM KCl, 200 µg/ml BSA, 5 µM DTT) on ice. The mixture was incubated at 25 ˚C for 15 min and loaded onto a native gel containing 6 % acrylamide (44 % stock solution; 29:1 acrylamide:bis-acrylamide; Severn Biotech) in 0.5 X Tris/Borate/EDTA (TBE) buffer. Gels were polymerized by adding 0.09 % ammonium persulfate (APS) and N,N,N',N'-tetramethyl-ethylene diamine (TEMED). After running for 20 min at 150V, gels were stained with SYBR® Gold Nucleic

Acid Gel Stain (Thermo Scientific, S11494) for 1 h at room temperature. The images were acquired with ChemiDoc Imaging system (Bio-Rad).

Affinity Purification and Mass Spectrometry (AP-MS)

Stable cell lines (full length ZMYND8 WT or BRD mutant, N228F) were selectively grown in the presence of 200 µg/ml hygromycin up to 80 % confluence before expression was induced via 1 µg/ml tetracycline for 24 h and the cells were harvested. Two 150-mm plates were induced with tetracycline and treated with 50 µM biotin for 24 h before harvesting. Cells were pelleted at low speed, washed with ice-cold PBS and frozen at -80 °C until purification. Protein interactions were investigated following the FLAG AP-MS protocol previously described (Lambert et al., 2014). Briefly, cells from two 150-mm plates were pelleted, frozen down and lysed in 1.5 ml ice cold 50 mM HEPES-NaOH pH 8.0, 100 mM KCl, 2 mM EDTA, 0.1 % NP40, and 10 % glycerol with 1 mM PMSF, 1 mM DTT and Sigma protease inhibitor cocktail (P8340, 1:500) added immediately prior to processing. To aid with lysis, the cells were frozen on dry ice and thawed in a 37 ˚C water bath, and then put back on ice. Samples were sonicated at 4 ˚C using three 10 sec bursts with 2 sec pauses at 35 % amplitude. 100 U of benzonase were then added and the lysates were incubated at 4 ˚C for 1 h with rotation, then centrifuged (20,817 x *g* for 20 min at 4 ˚C) and the supernatant was added to tubes containing 25 µl of 50 % magnetic anti-FLAG M2 bead (Sigma, M8823) slurry prewashed in lysis buffer. FLAG immunoprecipitation was allowed to proceed at 4 ˚C for 2 h with rotation. Beads were pelleted by centrifugation (1000 rpm for 1 min) and magnetized, then the unbound lysate was aspirated. The beads were demagnetized, washed with 1 ml lysis buffer and magnetized again to aspirate off the wash buffer. The beads were then washed with 1 ml of 20 mM Tris-HCl (pH 8.0) containing 2 mM $CaCl_2$, then any excess wash buffer was removed by centrifugation, magnetizing and pipetting off the liquid. The now-dry magnetic beads were removed from the magnet and re-suspended in 7.5 µl of 20 mM Tris-HCl (pH 8.0) containing 750 ng of trypsin (Sigma, T7575) and the mixture was incubated at 37 ˚C with agitation overnight. After the initial incubation, beads were magnetized and the supernatant was transferred to a fresh tube. Another 250 ng of trypsin was added to the mixture followed by incubation, without agitation, for 3-4 h. The sample was acidified with formic acid to a final concentration of 2 % and the tryptic digests were stored at -40 ˚C until ready for mass spectrometry analysis. Parental Flp-In T-REx HEK293

cells and cells expressing NLS-BirA* fused to FLAG tag (not treated with excess biotin) were used as negative controls for AP-MS experiments and processed in parallel to the bait-expressing cell lines

Proximity biotinylation coupled to mass spectrometry (BioID-MS)

BioID was performed following the previously published protocol (Lambert et al., 2015). Briefly, cell pellets were thawed in 1.5 ml ice cold RIPA buffer containing 50 mM Tris-HCl (pH 7.5), 150 mM NaCl, 1% NP-40, 1 mM EDTA, 1 mM EGTA, 0.1 % SDS and 0.5 % sodium deoxycholate. PMSF (1 mM), DTT (1 mM) and Sigma protease inhibitor cocktail (P8340, 1:500) were added immediately before use. The lysates were sonicated, treated with benzonase and centrifuged as described in the FLAG AP-MS section. For each sample, 60 µl of streptavidin-sepharose bead slurry (GE Healthcare, Cat 17-5113-01) were pre-washed three times with 1 ml of lysis buffer by pelleting the beads with gentle centrifugation and aspirating off the supernatant before adding the next wash. Biotinylated proteins were captured on pre-washed streptavidin beads for 3 h at 4 ˚C with rotation. The beads were then gently pelleted and the unbound supernatant was saved for further analysis. The beads were then washed 2 x 1 ml with RIPA buffer and 3 x 1 ml with 50 mM ammonium bicarbonate (pH 8.0). Following the final wash, the beads were pelleted and any excess liquid was aspirated off. Beads were then re-suspended in 100 µl of 50 mM ammonium bicarbonate (pH 8.0), and 1 µg of trypsin solution was added. The samples were incubated overnight at 37 ˚C with rotation and an additional 1 µg of trypsin was then added, followed by a further incubation for 2-4 h. The beads were pelleted and the supernatant was transferred to a fresh tube. The beads were rinsed with 2 x 100 µl HPLC grade water and the wash fraction was combined with the supernatant. The peptide solution was acidified with 50 % formic acid to a final concentration of 2 % and the samples were placed in a speedvac to dry. Tryptic peptides were resuspended in 25 µl of 5 % formic acid and stored at -80 ˚C until analyzed by mass spectrometry. Parental Flp-In T-REx HEK293 cells, and stable cells expressing BirA*-FLAG fused either to a green fluorescent protein (GFP) or to a nuclear localization sequence (NLS) were used as negative controls for the BioID experiments and processed in parallel to the bait proteins.

Mass Spectrometry and Data Analysis

AP-MS samples, BioID samples and controls were analyzed by mass spectrometry in at least two biological replicates. 5 µl of each sample were directly loaded at 400 nl/min onto a 75 µm x 12 cm emitter packed with 3 µm ReproSil-Pur C_{18}-AQ (Dr.Maisch HPLC GmbH, Germany). Peptides were eluted from the column over a 90 min gradient generated by a NanoLC-Ultra 1D plus (Eksigent, Dublin CA) nano-pump and analyzed on a TripleTOF™ 5600 instrument (AB SCIEX, Concord, Ontario, Canada). The gradient was delivered at 200 nl/min, starting at 2 % acetonitrile with 0.1 % formic acid and ending at 35 % acetonitrile with 0.1 % formic acid over 90 min, followed by a 15 min clean-up at 80 % acetonitrile with 0.1 % formic acid, and a 15 min equilibration period back to 2 % acetonitrile with 0.1 % formic acid for a total of 120 min. To minimize carryover between each sample, the analytical column was washed for 3 h by running an alternating "saw-tooth" gradient from 35 % acetonitrile with 0.1 % formic acid to 80 % acetonitrile with 0.1 % formic acid, holding each gradient concentration for 5 min. Analytical column and instrument performance were verified after each sample by loading 30 fmol BSA tryptic peptide standard (Michrom Bioresources Inc. Fremont, CA) with 60 fmol α-Casein tryptic digest and running a short 30 min gradient. TOF MS calibration was performed on BSA reference ions before running the next sample in order to adjust for mass drift and verify peak intensity. The instrument method was set to a discovery or IDA mode which consisted of one 250 ms MS1 TOF survey scan from 400-1300 Da followed by twenty 100 ms MS2 candidate ion scans from 100-2000 Da in high sensitivity mode. Only ions with a charge of 2+ to 4+ which exceeded a threshold of 200 cps were selected for MS2, and former precursors were excluded for 10 sec after 1 occurrence.

Mass spectrometry data generated were stored, searched and analyzed using the *ProHits* laboratory information management system (LIMS) platform (Liu et al., 2010). Within *ProHits*, the resulting WIFF files were first converted to an MGF format using WIFF2MGF converter and to an mzML format using *ProteoWizard* (v3.0.4468) and the *AB SCIEX MS Data Converter* (V1.3 beta) and then searched using *Mascot* (v2.3.02) and *Comet* (v2012.02 rev.0). The spectra were searched with the RefSeq database (version 53, May 28th, 2014) acquired from NCBI against a total of 34374 human and adenovirus sequences supplemented with "common contaminants" from the Max Planck Institute (http://141.61.102.106:8080/share.cgi?ssid=0f2gfuB) and the Global

Proteome Machine (GPM; http://www.thegpm.org/crap/index.html). The database parameters were set to search for tryptic cleavages, allowing up to 2 missed cleavage sites per peptide with a mass tolerance of 40 ppm for precursors with charges of 2+ to 4+ and a tolerance of +/- 0.15 amu for fragment ions. Variable modifications were selected for deamidated asparagine and glutamine and oxidized methionine. The results from each search engine were analyzed through the *Trans-Proteomic Pipeline* (TPP v4.6 OCCUPY rev 3) (Deutsch et al., 2010) via the *iProphet* pipeline (Shteynberg et al., 2011). *SAINTexpress* (v3.3) (Teo et al., 2014) was used as a statistical tool to calculate the probability value of each potential protein-protein interaction from background contaminants using default parameters. Unless otherwise specified, controls were compressed to 6 samples and a FDR of 1 % or lower were required for proteins to be classified as significant interaction partners of ZMYND8.

AP-MS and BioID-MS Data Functional Enrichment, Visualization and Deposition

Functional enrichment analysis was performed using *DAVID Bioinformatics Resources* (v6.7) (Huang da et al., 2009). Functional Annotation Clustering (July 2015) was performed and Biological Process (BP FAT) are reported, with enrichment probabilities adjusted for GO terms with Benjamini-Hochberg FDR correction of 0.01 or smaller.

Dot plots of FL-ZMYND8 WT and FL-ZMYND8 N228F mutant interactomes obtained by BioID and AP-MS were generated using custombuilt proteomics data visualization tools (Knight et al., 2015). All MS files used in this study were deposited at MassIVE (http://massive.ucsd.edu) and assigned ID **MSV000079336**.

RNA Interference (ZMYND8, HATs) and ZMYND8 knock-down

The knock-down experiments of ZMYND8 or histone acetyl-transferases (HATs, including PCAF, p300, TADA2A) were performed by RNA interference using Lipofectamine RNAiMAX reagent (Cat.#13778-150). In the case of ZMYND8, HEK293 cells expressing inducible 3xFLAG-ZMYND8 were reverse transfected with 100 pmol Stealth siRNAs targeting ZMYND8 (Cat.#HSS119060; Invitrogen) or Stealth Negative Control for 48 h. Cells were harvested for RNA extraction, western blot or ChIP-qPCR analysis. In the case of HATs, cells were reverse transfected with 20 pmol siRNAs (Dharmacon ON TARGET SMARTpool; L005055-00 for PCAF; L003486-00 for p300, and L017516-00 for TADA2A) or Non-targeting siRNA (D001810-01-20) 72 h, as well as 10 nM

Doxycycline to induce 3xFLAG-ZMYND8 expression. Cells were harvested for western blot and ChIP-qPCR analysis

RNA extraction

Total RNA was extracted and prepared using the RNeasy Mini Kit (Cat.#74106; Qiagen) including an on-column DNase I digestion (Cat.#79254; Qiagen), according to the manufacturer's instructions. The resulting RNA was quantified and quality controlled using a Nanodrop spectrophotometer (model ND1000; Thermo Scientific) and the Agilent RNA 6000 Nano Kit (Cat.#5067-1511, Agilent Technologies).

Chromatin Immunoprecipitation (ChIP) and ChIP-seq

The ChIP DNA was collected using the SimpleChIP™ Enzymatic Chromatin IP Kit (Cell Signaling Technology #9005), according to the manufacturer's protocol. Briefly, HEK293 cells were collected from 15 cm plates and treated with 1 % formaldehyde (cross-linking) for 10 min and 125 mM Glycine for another 5 min (quenching) at room temperature. After washing three times with 1X PBS, cells were lysed in buffer A for 10 min on ice, then washed once in buffer B. Cells were re-suspended in buffer B, then treated with MNase (2000 gel units) at 37 ˚C for 30 min, before the digestion was stopped by adding EDTA (10 µl 0.5 mM EDTA per 100 µl reaction). Chromatin extracts were harvested following complete lysis of nuclei by sonication. 10 µg of chromatin samples were incubated with each antibody (2 µl anti-ZMYND8 (Bethyl Laboratory, A302-089A); 4 µl anti-H3K14ac (Millipore, 07-353); 4 µl anti-H3K27ac (Millipore, 07-595); 2 µl anti-Flag (Sigma, F1804)) for 12 h at 4 °C with rotation. Then 30 µl of ChIP-Grade ProteinG Magnetic Beads (#9006, Cell Signalling Technology) were added to each sample and incubated for 2 h at 4 °C with rotation. The beads were washed with low salt buffer and high salt buffer. Chromatin samples were eluted from antibody/beads in 150 µl of ChIP elution buffer for 30 min at 65 °C. The samples were reverse cross-linked by adding 6 µl 5M NaCl and 2 µl Proteinase K (20 mg/ml) for 2 h at 65 °C and then purified in DNA purification columns.

Library construction and sequencing were performed at the Wellcome Trust Centre for Human Genetics; Samples were quantified using the Quant-iT™ PicoGreen® dsDNA Kits (Invitrogen) and sample integrity was assessed using Agilent's Tapestation system both in accordance to manufacturer specifications. Libraries were constructed using PrepX Complete ILMN 32i DNA

Library Kit (#400076) with an input of 5 ng where available. Ligation of adapters was performed using Illumina Adapters within the Multiplexing Sample Preparation Oliogonucleotide Kit. Each library was PCR enriched with 25 µM each of the following custom primers: Multiplex PCR primer 1.0 5'-AATGATACGGCGACCACCGAGATCTACACTCTTTCCCTACACGACGCTCTTCCGATCT-3' and Index primer 5'-CAAGCAGAAGACGGCATACGAGAT[INDEX]CAGTGACTGGAGTTCAGACGTGTGCTCTTCCGATCT-3'. Indexes used were 8 bp long as previously described (Lamble et al., 2013). Enrichment and adapter extension of each preparation was obtained using 15 µl of size selected library in a 50 µl PCR reaction using NEBNext High-Fidelity 2X PCR Master Mix. After 18 cycles of amplification (one cycle at 98 ˚C for 30 sec followed by 18 cycles of the round (10 sec at 98 ˚C, 30 sec at 65 ˚C and then 30 sec at 72 ˚C) and then one cycle at 72 ˚C for 5 min. Hold at 4 ˚C), the PCR products were purified with Ampure beads (Agencourt/Beckman) on a Biomek NXp. The concentrations used to generate the multiplex pool were determined by Picogreen and pooling was performed using a Biomek 3000. The final size distribution of the pool was determined using a Tapestation 1DK system (Agilent), quantification for sequencing was determined using Library Quant Primers + KSF (Illumina #KK4923) and an MX3005P instrument (Agilent) before sequencing on an Illumina HiSeq 2500 as 51 single end. Base-calling was performed with bclToFastq (Illumina base-caller) version 1.8.4A.

Chromatin Immunoprecipitation & Quantitative PCR (ChIP-qPCR)

Eluted chromatin supernatants were purified using the QIAquick PCR Purification Kit (QIAGEN, #28106), and analysed by quantitative PCR. All reactions were run on a LightCycler 480 Instrument II (Roche) using LightCycler® 480 SYBR Green I Master (# 04887352001; Roche) according to the manufacturer's protocol. Samples were prepared on 384-well plates with a final reaction volume of 10 µl, containing 5 µl LightCycler® 480 SYBR Green I Master, 0.4 µM forward and reverse primers (**Supplemental Table S9**) and 2 µl of diluted samples. Data were normalized as % of input and are presented as mean ± SD from three biological replicates. Statistical significance was analysed using two-tailed Student's *t*-test or one-way analysis of variance followed by Dunnett's multiple comparison test. *P* values are presented such that: P<0.001 ****; P<0.005 ***; P<0.01 **; P<0.05, *; Not-significant, ns.

ChIP-seq Data Analysis

FASTQ files for ZMYND8, H3K14ac, H3K27ac were mapped to the human reference genome (hg19) by *BWA-MEM* (v.0.7.13) (Li and Durbin, 2009) using default parameters allowing up to two mismatches. SRA files were downloaded using the *SRA toolkit* (v.2.6.2) from NCBI's Gene Expression Omnibus for H3K14ac (GSM521881) and H3K27ac (GSM521887) in IMR90 cells; RACK7 and H3K27ac in ZR-75-30 cell lines (GSE71327); SRA files were processed with *bowtie2* (v.2.2.9) (Langmead and Salzberg, 2012) using default parameters. The genome ChIP-seq profiles for ZMYND8, H3K14ac, H3K27ac were generated using *HOMER* (v.4.8) (Heinz et al., 2010). The ChIP-seq profiles were normalized to GC content, 10^9 total tag numbers and peaks were called at FDR $\leq 10^{-3}$. Peaks were annotated by REFSEQ genes. Bigwig coverage profiles were generated with the UCSD toolbox. Enrichment profiles of DNA-interacting proteins at functionally important regions were visualized using *ngs.plot* (v.2.6.1) (Shen et al., 2014). Enhancer regions in HEK293 and computation of enrichment profiles in these loci were defined employing annotations from the FANTOM5 consortium (Andersson et al., 2014). Heatmaps of read coverage in ZMYND8-bound TSS and FANTOM5 enhancers were constructed with *deepTools* (v.2.2.4) (Ramirez et al., 2014). Canonical correlation analysis in ZMYND8-bound enhancers was performed with CCA (v.1.2) (Gonzalez et al., 2008).

ChIP-seq tags enrichment in promoter and enhancer regions relative to local background was computed with the *localEnrichmentBed* toolkit from https://github.com/dariober/bioinformatics-cafe/tree/master/localEnrichmentBed. The coordinates of the respective regulatory genomic elements were downloaded from the FANTOM5 data repository (http://fantom.gsc.riken.jp/5/data/). The *P*-value computed by the algorithm was filtered using a threshold of 0.01 to determine significantly enriched genomic loci. ChIP-sequencing data have been deposited in NCBI's Gene Expression Omnibus and are accessible through GEO accession number **GSE81696**.

Supplemental References

Abrahams, J.P., and Leslie, A.G.W. (1996). Methods used in the structure determination of bovine mitochondrial F-1 ATPase. Acta Crystallogr D *52*, 30-42.

Andersson, R., Gebhard, C., Miguel-Escalada, I., Hoof, I., Bornholdt, J., Boyd, M., Chen, Y., Zhao, X., Schmidl, C., Suzuki, T., *et al.* (2014). An atlas of active enhancers across human cell types and tissues. Nature *507*, 455-461.

Aslanidis, C., and de Jong, P.J. (1990). Ligation-independent cloning of PCR products (LIC-PCR). Nucleic Acids Res *18*, 6069-6074.

Chen, V.B., Arendall, W.B., Headd, J.J., Keedy, D.A., Immormino, R.M., Kapral, G.J., Murray, L.W., Richardson, J.S., and Richardson, D.C. (2010). MolProbity: all-atom structure validation for macromolecular crystallography. Acta Crystallogr D *66*, 12-21.

Couzens, A.L., Knight, J.D., Kean, M.J., Teo, G., Weiss, A., Dunham, W.H., Lin, Z.Y., Bagshaw, R.D., Sicheri, F., Pawson, T., *et al.* (2013). Protein interaction network of the mammalian Hippo pathway reveals mechanisms of kinase-phosphatase interactions. Sci Signal *6*, rs15.

Cowtan, K. (2006). The Buccaneer software for automated model building. 1. Tracing protein chains. Acta Crystallogr D *62*, 1002-1011.

Deutsch, E.W., Mendoza, L., Shteynberg, D., Farrah, T., Lam, H., Tasman, N., Sun, Z., Nilsson, E., Pratt, B., Prazen, B., *et al.* (2010). A guided tour of the Trans-Proteomic Pipeline. Proteomics *10*, 1150-1159.

Emsley, P., Lohkamp, B., Scott, W.G., and Cowtan, K. (2010). Features and development of Coot. Acta Crystallogr D *66*, 486-501.

Evans, P.R., and Murshudov, G.N. (2013). How good are my data and what is the resolution? Acta Crystallogr D *69*, 1204-1214.

Filippakopoulos, P., Picaud, S., Mangos, M., Keates, T., Lambert, J.P., Barsyte-Lovejoy, D., Felletar, I., Volkmer, R., Muller, S., Pawson, T., *et al.* (2012). Histone recognition and large-scale structural analysis of the human bromodomain family. Cell *149*, 214-231.

Filippakopoulos, P., Qi, J., Picaud, S., Shen, Y., Smith, W.B., Fedorov, O., Morse, E.M., Keates, T., Hickman, T.T., Felletar, I., *et al.* (2010). Selective inhibition of BET bromodomains. Nature *468*, 1067-1073.

Gibson, D.G., Smith, H.O., Hutchison, C.A., 3rd, Venter, J.C., and Merryman, C. (2010). Chemical synthesis of the mouse mitochondrial genome. Nat Methods *7*, 901-903.

Gibson, D.G., Young, L., Chuang, R.Y., Venter, J.C., Hutchison, C.A., 3rd, and Smith, H.O. (2009). Enzymatic assembly of DNA molecules up to several hundred kilobases. Nat Methods *6*, 343-345.

Gonzalez, I., Dejean, S., Martin, P.G.P., and Baccini, A. (2008). CCA: An R package to extend canonical correlation analysis. Journal of Statistical Software *23*, 1-14.

Heinz, S., Benner, C., Spann, N., Bertolino, E., Lin, Y.C., Laslo, P., Cheng, J.X., Murre, C., Singh, H., and Glass, C.K. (2010). Simple combinations of lineage-determining transcription factors prime cis-regulatory elements required for macrophage and B cell identities. Mol Cell *38*, 576-589.

Huang da, W., Sherman, B.T., and Lempicki, R.A. (2009). Systematic and integrative analysis of large gene lists using DAVID bioinformatics resources. Nat Protoc *4*, 44-57.

Kabsch, W. (2010). XDS. Acta Crystallogr D *66*, 125-132.

Knight, J.D., Liu, G., Zhang, J.P., Pasculescu, A., Choi, H., and Gingras, A.C. (2015). A web-tool for visualizing quantitative protein-protein interaction data. Proteomics *15*, 1432-1436.

Lambert, J.P., Tucholska, M., Go, C., Knight, J.D., and Gingras, A.C. (2015). Proximity biotinylation and affinity purification are complementary approaches for the interactome mapping of chromatin-associated protein complexes. J Proteomics *118*, 81-94.

Lambert, J.P., Tucholska, M., Pawson, T., and Gingras, A.C. (2014). Incorporating DNA shearing in standard affinity purification allows simultaneous identification of both soluble and chromatin-bound interaction partners. J Proteomics *100*, 55-59.

Lamble, S., Batty, E., Attar, M., Buck, D., Bowden, R., Lunter, G., Crook, D., El-Fahmawi, B., and Piazza, P. (2013). Improved workflows for high throughput library preparation using the transposome-based Nextera system. BMC Biotechnol *13*, 104.

Langmead, B., and Salzberg, S.L. (2012). Fast gapped-read alignment with Bowtie 2. Nat Methods *9*, 357-359.

Li, H., and Durbin, R. (2009). Fast and accurate short read alignment with Burrows-Wheeler transform. Bioinformatics *25*, 1754-1760.

Liu, G., Zhang, J., Larsen, B., Stark, C., Breitkreutz, A., Lin, Z.Y., Breitkreutz, B.J., Ding, Y., Colwill, K., Pasculescu, A.*, et al.* (2010). ProHits: integrated software for mass spectrometry-based interaction proteomics. Nat Biotechnol *28*, 1015-1017.

Murshudov, G.N., Skubak, P., Lebedev, A.A., Pannu, N.S., Steiner, R.A., Nicholls, R.A., Winn, M.D., Long, F., and Vagin, A.A. (2011). REFMAC5 for the refinement of macromolecular crystal structures. Acta Crystallogr D *67*, 355-367.

Neumann, H., Hancock, S.M., Buning, R., Routh, A., Chapman, L., Somers, J., Owen-Hughes, T., van Noort, J., Rhodes, D., and Chin, J.W. (2009). A method for genetically installing site-specific acetylation in recombinant histones defines the effects of H3 K56 acetylation. Mol Cell *36*, 153-163.

Painter, J., and Merritt, E.A. (2006). Optimal description of a protein structure in terms of multiple groups undergoing TLS motion. Acta Crystallogr D *62*, 439-450.

Philpott, M., Yang, J., Tumber, T., Fedorov, O., Uttarkar, S., Filippakopoulos, P., Picaud, S., Keates, T., Felletar, I., Ciulli, A.*, et al.* (2011). Bromodomain-peptide displacement assays for interactome mapping and inhibitor discovery. Mol Biosyst *7*, 2899-2908.

Picaud, S., and Filippakopoulos, P. (2015). SPOTing Acetyl-Lysine Dependent Interactions. Microarrays *4*, 370-388.

Ramirez, F., Dundar, F., Diehl, S., Gruning, B.A., and Manke, T. (2014). deepTools: a flexible platform for exploring deep-sequencing data. Nucleic Acids Res *42*, W187-191.

Rothbart, S.B., Krajewski, K., Nady, N., Tempel, W., Xue, S., Badeaux, A.I., Barsyte-Lovejoy, D., Martinez, J.Y., Bedford, M.T., Fuchs, S.M.*, et al.* (2012a). Association of UHRF1 with methylated H3K9 directs the maintenance of DNA methylation. Nat Struct Mol Biol *19*, 1155-1160.

Rothbart, S.B., Krajewski, K., Strahl, B.D., and Fuchs, S.M. (2012b). Peptide microarrays to interrogate the "histone code". Methods Enzymol *512*, 107-135.

Savitsky, P., Bray, J., Cooper, C.D., Marsden, B.D., Mahajan, P., Burgess-Brown, N.A., and Gileadi, O. (2010). High-throughput production of human proteins for crystallization: the SGC experience. J Struct Biol *172*, 3-13.

Sheldrick, G.M. (2010). Experimental phasing with SHELXC/D/E: combining chain tracing with density modification. Acta Crystallogr D *66*, 479-485.

Shen, L., Shao, N., Liu, X., and Nestler, E. (2014). ngs.plot: Quick mining and visualization of next-generation sequencing data by integrating genomic databases. BMC Genomics *15*, 284.

Shteynberg, D., Deutsch, E.W., Lam, H., Eng, J.K., Sun, Z., Tasman, N., Mendoza, L., Moritz, R.L., Aebersold, R., and Nesvizhskii, A.I. (2011). iProphet: multi-level integrative analysis of shotgun proteomic data improves peptide and protein identification rates and error estimates. Mol Cell Proteomics *10*, M111 007690.

Smith, C.M. (2005). Quantification of acetylation at proximal lysine residues using isotopic labeling and tandem mass spectrometry. Methods *36*, 395-403.

Tachiwana, H., Kagawa, W., Osakabe, A., Kawaguchi, K., Shiga, T., Hayashi-Takanaka, Y., Kimura, H., and Kurumizaka, H. (2010). Structural basis of instability of the nucleosome containing a testis-specific histone variant, human H3T. Proc Natl Acad Sci U S A *107*, 10454-10459.

Teo, G., Liu, G., Zhang, J., Nesvizhskii, A.I., Gingras, A.C., and Choi, H. (2014). SAINTexpress: improvements and additional features in Significance Analysis of INTeractome software. J Proteomics *100*, 37-43.

Vonrhein, C., Blanc, E., Roversi, P., and Bricogne, G. (2007). Automated structure solution with autoSHARP. Methods Mol Biol *364*, 215-230.

Wiseman, T., Williston, S., Brandts, J.F., and Lin, L.N. (1989). Rapid Measurement of Binding Constants and Heats of Binding Using a New Titration Calorimeter. Anal Biochem *179*, 131-137.
